# Reversible Assembly and Disassembly of σ‐Aromatic B_3_ Rings

**DOI:** 10.1002/chem.71615

**Published:** 2026-08-25

**Authors:** Julian Krauß, Frederick Koch, Niels Karsten Paul Hecht, Elisabeth Kaifer, Hans‐Jörg Himmel

**Affiliations:** ^1^ Anorganisch‐Chemisches Institut Ruprecht‐Karls‐Universität Heidelberg Heidelberg Germany

**Keywords:** adduct, borane, boranes, boron, chemistry, diborane

## Abstract

Electron‐deficient, planar oligoboron ring compounds fascinate due to their peculiar bonding properties (σ‐aromaticity) and reactivity; however, the directed synthesis of such compounds is challenging. In this work we report on the reaction of cationic diboranes with boranes to give cationic, σ‐aromatic B_3_ ring compounds, with three‐center‐two‐electron bond between the three boron atoms, being analogues of B_3_H_8_^−^, but with opposite charge. Base‐induced, reversible disassembly of the cationic B_3_ ring leads back to cationic diboranes and borane adducts. Quantum‐chemical calculations show that the thermodynamics of these reactions can be tuned by choice of the base. The reactions interconvert electron‐deficient boron three‐center bonds and electron‐precise two‐center bonds and disclose new synthetic access routes to cationic diboranes and σ‐aromatic B_3_ rings.

## Graphical abstract

B_3_ rings are the smallest σ‐aromatic boron compounds. Here, we show for the first time that cationic diboranes with an electron‐precise (2c,2e) bond can be reversibly converted to σ‐aromatic B_3_ rings with electron‐deficient (3c,2e) bonding. Base‐induced disassembly of the B_3_ ring leads back to cationic diboranes and monoborane adducts.

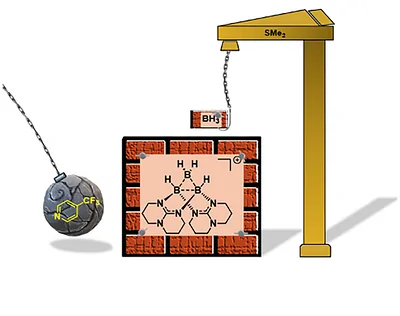

## 1 Introduction

Electron‐deficient oligoboron compounds were intensively studied in the past decades, in the context of basic concepts such as σ‐aromaticity and three‐dimensional aromaticity, as well as for a variety of applications [1, 2]. The electronic properties of these compounds can be described in terms of multicenter bonding (e.g., three‐center two‐electron (3c,2e)) bonding. Recently, σ‐bond resonance between (3c,2e) B_3_ rings was shown to be of importance for the understanding of the structure and properties of flat boron clusters and sheets (borophene), adding to the importance of these electron‐deficient multicenter bonds [3]. However, strategies for the directed construction of electron‐deficient oligoboron compounds are still very much underdeveloped.

B_3_ ring compounds are the smallest units that can show σ‐aromaticity. Among these compounds, salts of octahydrotriborate, (B_3_H_8_)^−^, and neutral B_3_H_7_L (with a donor L (e.g., an amine, see structures in Figure 1) are particularly well studied due to their relevance for applications ranging from hydrogen storage to ionic liquids and chemical vapor deposition (CVD) processes. Although several routes were developed for the synthesis of salts of B_3_H_8_^−^, some of them use relatively unstable reagents that are not easily accessible. Hence, already in 1961, NMe_4_B_3_H_8_ was prepared by stepwise degradation of decaborane, B_10_H_14_ [4]. Other routes start from NaBH_4_ and I_2_ [5], THF·BH_3_ and Na/Hg (synthesis of NaB_3_H_8_) [6], or NaBH_4_ and a metal halide (e.g., CuCl or BiCl_3_) [7]. Also, several neutral triboranes B_3_H_7_L were synthesized. Kodama and coworkers reported the synthesis of ammonia‐triborane, B_3_H_7_NH_3_, starting with tetraborane, B_4_H_10_ [8]. Later, B_3_H_7_NH_3_ was synthesized in a more convenient way from NaB_3_H_8_ [9]. Moreover, nucleophilic substitution reactions in the presence of a Lewis acid were developed, allowing to synthesize neutral triboron compounds such as B_3_H_7_PPh_3_ and B_3_H_7_NEt_3_ from B_3_H_8_^−^ [10]. Finally, several metallotriboranes were synthesized, in which (B_3_H_8_)^−^ interacts through B─H─M bonds with a metal (M). In some cases, tetraborane‐like structures are formed, with a metal fragment replacing a BH_2_ group in B_4_H_10_ [11, 12]. However, the variety of structures and properties of such compounds is still very much limited.

**FIGURE 1:**
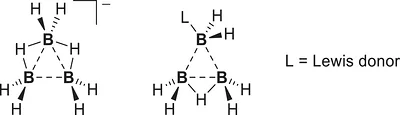
Structures of anionic (B_3_H_8_)^−^ and neutral B_3_H_7_L triboranes (L = Lewis base, e.g., THF or NH_3_) with 3c,2e bond between the three boron atoms.

In recent years, we developed the synthesis of electron‐deficient B_3_ and B_4_ ring compounds, starting from diboranes with electron‐donating bicyclic guanidinate substituents [13, 14]. We synthesized triboron compounds with a triangular electron‐deficient boron ring, being analogues to B_3_H_8_^−^ but with opposite charge, following the general strategy sketched in Figure 2a from the dihydridodiborane [HB(hpp)]_2_ (**DB1**, hpp = 1,3,4,6,7,8‐hexahydro‐2*H*‐pyrimido[1,2‐*a*]pyrimidine) and a source of a cationic borinium ion (BR_2_^+^). Hence, reaction of [HB(hpp)]_2_ with *N*‐borabicyclo[3.3.1]nonan‐9‐yl‐bis(trifluoromethanesulfonyl)imide or trifluoromethanesulfonate [15] led to the B_3_ ring **TB1** ^+^ (Figure 2b) [16]. Later, a quite special synthesis of another, related triborane ring, **TB2** ^+^, was developed [17], applying catecholatoborane as a source for the BH_2_^+^ ion (Figure 2b). However, this strategy is limited since it relies on a suitable source of an extremely reactive borinium ion as one of the starting reagents.

**FIGURE 2:**
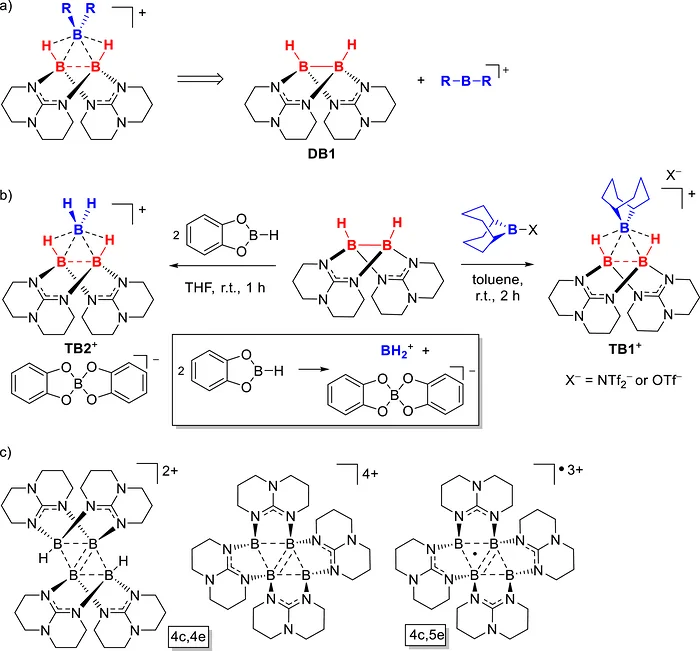
(a) Strategy for the synthesis of σ‐aromatic B_3_ rings from diborane [HB(hpp)]_2_ and a borinium ion (BR_2_^+^) source. (b) Synthesis of two different cationic B_3_ ring compounds using this approach. (c) Structures of the highly‐electron deficient tetraboron compounds [H_2_B_4_(hpp)_4_]^2+^,{[B(hpp)]_4_}^4+^, and {[B(hpp)]_4_}^·3+^.

Electron‐deficient, planar tetraboron compounds were also synthesized from simple diboranes such as **DB1**. The dihydridotetraboron compound [H_2_B_4_(hpp)_4_]^2+^ (Figure 2c) was obtained by hydride abstraction from **DB1** with B(C_6_F_5_)_3_ [18]. Double hydride abstraction from **DB1** and double triflate abstraction from the ditriflatodiborane [(TfO)B(hpp)]_2_ yielded [B_4_(hpp)_4_]**^·^** ^3+^ [19] and [B_4_(hpp)_4_]^4+^, respectively (Figure 2c) [20]. All three B_4_ rings exhibit planar, rhomboid B_4_ cores. The (4c,4e) bond in the B_4_ cores of [H_2_B_4_(hpp)_4_]^2+^ and [B_4_(hpp)_4_]^4+^ can be described as two B_3_ (3c,2e) bonds sharing the short diagonal. A (4c,5e) bond is present in the radical [B_4_(hpp)_4_]^3+^; the unpaired electron is delocalized in the σ‐bond scaffold. The ability of the σ‐aromatic [B_4_(hpp)_4_]^4+^ (a red and fluorescent compound) to take up an electron without loss of the planar, rhomboid B_4_ core adds to the interest in such compounds for optoelectronic applications.

Here, we develop a novel synthetic route to σ‐aromatic B_3_ rings. According to this route, a neutral monoborane is reacted with a precursor for a monocationic diborane (Scheme 1). Hence, the diborane and not the borane building block carries the positive charge. The tri‐coordinate boron atom in the diborane can be stabilized by a labile, neutral donor substituent that is readily eliminated. Compared with the previously followed strategy shown in Figure 2a, this approach has the advantage that it does not require a borinium cation source as a starting reagent and principally provides access to a variety of stable compounds with different substituents R. In this work, we also show that the B_3_ ring can be reversibly disassembled by reaction with a suitable Lewis base, giving back a neutral monoborane and a cationic diborane. The results presented herein provide the first example of the reversible assembly of σ‐aromatic electron deficient (3c,2e) bonds from diboranes with electron‐precise (2c,2e) bonds.

**SCHEME 1:**
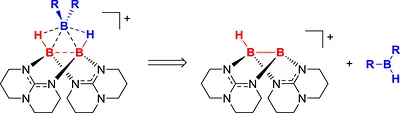
B_3_ ring synthesis from a cationic diborane and a neutral monoborane, reported in this work.

## 2 Results and Discussion

### 2.1 B_3_ Ring Assembly From Neutral Monoboranes and Cationic Diboranes

The first task was to identify a suitable cationic diborane source for the construction of B_3_ ring compounds according to the envisioned new strategy in Scheme 1. Obviously, a stabilizing ligand is necessary that protects the compound from dimerization to give [B_4_H_2_(hpp)_4_]^2+^ (Figure 2c) [18] but can nevertheless be readily substituted. We previously demonstrated that substitution reactions in diboranes with sp^3^‐hybridized boron atoms and bridging hpp substituents generally proceed via an elimination‐addition (S_N_1) pathway, obeying the octet rule [21]. We tested the addition of B(C_6_F_5_)_3_ to a solution of the dihydridodiborane [HB(hpp)]_2_ (**DB1**) and H_3_B·NMe_3_ as a way to obtain a B_3_ ring [15]. In the NMR spectra, the formation of the B_3_ ring was indeed observed, but there were additional signals due to [B_4_H_2_(hpp)_4_]^2+^ formed through dimerization of the intermediate [HB(*μ*‐hpp)B]^+^ [18]. Therefore, this protocol is not a practically useful route to B_3_ rings. We envisioned the cationic diboranylphosphonium ion [HB(hpp)_2_BPCy_3_]^+^ (**DB2** ^+^) as possible precursor [22], being the product of the reaction between **DB1** and B(C_6_F_5_)_3_ in the presence of PCy_3_ (Figure 3a). The compound exhibits a broad signal in the ^11^B NMR spectrum around *δ* = –2.26 ppm, to which both B atoms contribute.

**FIGURE 3:**
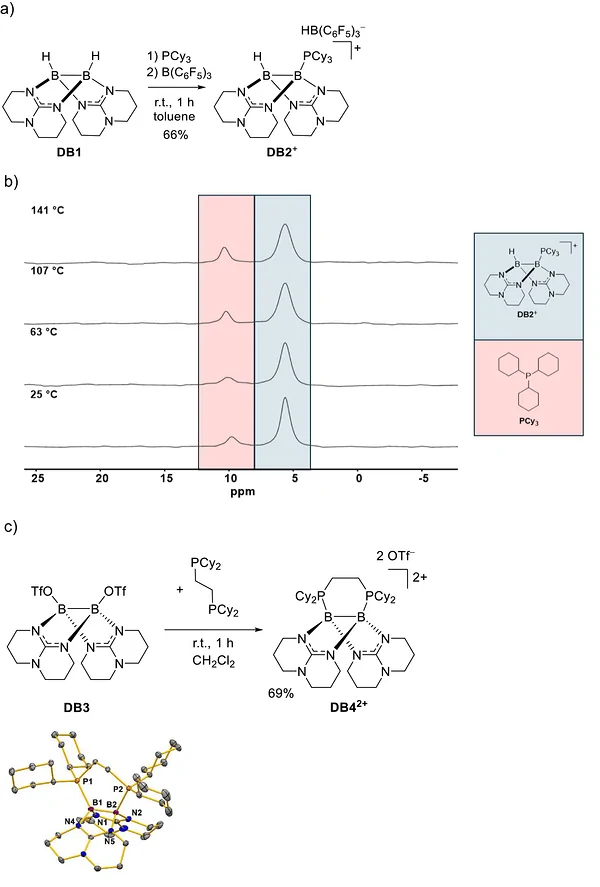
(a) Synthesis of **DB2**[HB(C_6_F_5_)_3_]. (b) Variable temperature ^31^P NMR spectra for **DB2**[HB(C_6_F_5_)_3_] in bromobenzene. (c) Synthesis of **DB4**[OTf]_2_ and illustration of the structure of **DB4** ^2+^. Displacement ellipsoids drawn at the 50% probability level. C−H hydrogens and counterions are omitted. Color code: B violet, C grey, N blue, P orange. Selected bond lengths (in Å) and angles (in °): B1−B2 1.753(3), B1−P1 2.020(2), B1−N1 1.539(3), B1−N4 1.539(3), B2−P2 2.002(2), B2−N2 1.537(3), B2−N5 1.548(3), N2−B1−N5 114.7(2).

Variable‐temperature NMR spectra were recorded to test the possibility of PCy_3_ elimination. Bromobenzene was chosen as solvent, since it allows measurements in a large temperature range, and the salt **DB2**[HB(C_6_F_5_)_3_] shows a good solubility in this solvent. Figure 3b shows the ^31^P NMR spectra at temperatures between 25°C and 141°C for **DB2**[HB(C_6_F_5_)_3_] in a bromobenzene solution. The NMR spectrum at room temperature contains a signal at *δ* = 6.40 ppm due to the boron‐bound phosphine in **DB2** ^+^, but in addition a weaker signal at the chemical shift value of free PCy_3_ (*δ* = 10.56 ppm). The ratio of uncoordinated to boron‐bound PCy_3_ can be estimated to be ca. 0.18:1 at 25°C from NMR signal integration. A weak signal of free PCy_3_ was previously observed in dichloromethane solution [22], but the ratio of free to coordinated phosphine was only 0.05:1 (see Supporting Information). The signal of free PCy_3_ increases with temperature, reaching 0.30:1 at 141°C in bromobenzene. The temperature induced changes are fully reversible. The signal in the ^11^B NMR spectrum is too broad to detect changes with increasing temperature, but the formation of a sp^2^‐hybridized boron atom can be excluded. Moreover, the signals due to [H_2_B_4_(hpp)_4_]^2+^ do not appear, showing that the cationic diborane does not dimerize upon phosphine elimination. From an Arrhenius analysis (see Supporting Information for details), one obtains Δ*G* = +17.3 ± 2.4 kJ mol^−1^, Δ*H* = +7.7 ± 1.9 kJ mol^−1^ and a minuscule entropy change, Δ*S* = –0.032 ± 0.0053 kJ mol^−1^ K^−1^, for the PCy_3_ elimination reaction. These values are significantly different to those derived from quantum‐chemical calculations (B3LYP+D3/def2‐TZVP) for the compound in the gas phase, yielding Δ*G* = –12 kJ mol^−1^, Δ*H* = +48 kJ mol^−1^ and Δ*S* = +0.203 kJ mol^−1^ K^−1^, and with inclusion of the solvent effect (Δ*G* = –46 kJ mol^−1^, Δ*H* = +15 kJ mol^−1^ in dichloromethane, and Δ*G* = –42 kJ mol^−1^, Δ*H* = +19 kJ mol^−1^ in bromobenzene). Most strikingly, the experimental analysis argues for almost no entropy change, although one might expect the elimination of phosphine to be associated with a significant entropy increase (in line with the calculations (gas phase and solvent). The results can be explained by a fast dissociation‐addition equilibrium. The eliminated PCy_3_ molecule remains close to the diboron compound within the same solvent cage, leading to a neglectable entropy change. The different signal ratios in bromobenzene and in dichloromethane suggest a solvent influence on this equilibrium. An alternative explanation is that the [HB(C_6_F_5_)_3_]^−^ anion interacts with the diboron cation formed upon PCy_3_ elimination (formation of a B–H–B bridge). Thereby, the anion binds in place for PCy_3_ to the cation, blocking its dimerization and leading to an entropy decrease that compensates the entropy gain from PCy_3_ release. This second possible explanation should lead to a change of the ^11^B NMR chemical shifts of the [HB(C_6_F_5_)_3_]^−^ anion. However, no changes were observed in the ^11^B NMR spectra. Therefore, this second explanation is less likely.

To obtain additional information, we treated the ditriflatodiborane precursor **DB3**, that is frequently used in our research group as a precursor to mono‐ and dicationic diborane compounds [21, 23, 24], with 1,2‐bis(dicyclohexylphosphino)ethane (dcpe) to obtain the diphosphino‐stabilized dicationic diborane **DB4**[OTf]_2_ (69% yield, Figure 3c). The ^31^P NMR spectrum in THF‐d_8_ solution contains a single signal at *δ* = –4.69 ppm, even at elevated temperature. Especially, no signal was observed at the chemical shift value of free dcpe (*δ* = 1.38 ppm), in contrast to the results obtained for **DB2**[HB(C_6_F_5_)_3_]. Obviously, the B–P bond is stronger in **DB4** ^2+^ than in **DB2** ^+^ due to the higher charge. Moreover, the chelate effect disfavors ligand dissociation. Hence, **DB3**[OTf]_2_ does not qualify as suitable precursor for the construction of B_3_ rings.

The weak B─P bond in **DB2**[HB(C_6_F_5_)_3_] allows substitution reactions leading to new cationic hydridodiboranes. Hence, reaction with pyridine or 4‐dimethylaminopyridine (DMAP) yielded the new cationic diboranes **DB5** ^+^ and **DB6** ^+^, with DMAP or pyridine in place of the PCy_3_ substituent (Scheme 2). The reactions proceed quantitatively, but purification to remove the PCy_3_ byproduct diminished the isolated yields. The two diboranes **DB5**[HB(C_6_F_5_)_3_] and **DB6**[HB(C_6_F_5_)_3_] were eventually isolated in pure form in a yield of 33% and 53%, respectively.

**SCHEME 2:**
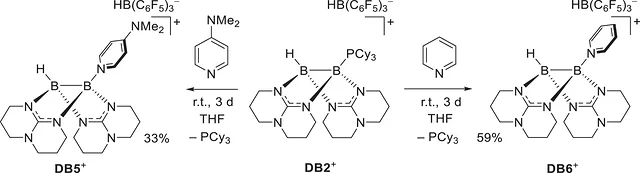
Substitution reactions starting with [HB(*μ*‐hpp)_2_BPCy_3_][HB(C_6_F_5_)_3_], in which the PCy_3_ substituent is replaced by DMAP (**DB5** ^+^) or pyridine (**DB6** ^+^).

These results demonstrate that the PCy_3_ substituent in **DB2** ^+^ can be replaced in substitution reactions, highlighting its potential as a versatile precursor for the synthesis of B_3_ rings. Therefore, we tested the suitability of this compound for the assembly of electron‐deficient B_3_ ring compounds according to the projected route sketched in Scheme 1. Indeed, reaction of **DB2**[HB(C_6_F_5_)_3_] with H_3_B·SMe_2_ in CH_2_Cl_2_ solution at room temperature yielded the B_3_ ring **TB2**[HB(C_6_F_5_)_3_] (Figure 4a). The NMR spectra indicated quantitative conversion, but the separation from the byproduct H_3_B·PCy_3_ required several washing cycles that greatly reduced the yield. The salt **TB2**[HB(C_6_F_5_)_3_] was eventually isolated in pure form in a yield of 19%.

**FIGURE 4:**
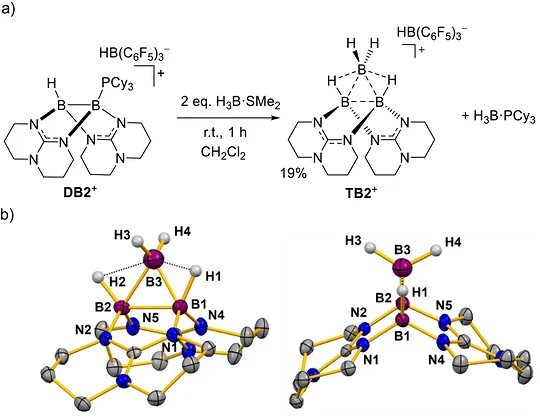
(a) Synthesis of **TB2**[HB(C_6_F_5_)_3_] from **DB2**[HB(C_6_F_5_)_3_] according to the strategy in Scheme 1. (b) Solid‐state structure of **TB2** ^+^ from two perspectives. Displacement ellipsoids drawn at the 50% probability level. C−H hydrogen atoms and counterion omitted. Color code: B violet, C grey, N blue. Selected bond lengths (in Å) and angles (in °): B1−B2 1.927(3), B1−B3 1.715(3), B2−B3 1.861(3), B1−N1 1.503(3), B1−N4 1.501(3), B2−N2 1.520(3), B2−N5 1.517(3), B1−B2−B3 53.8(1), B1−B3−B2 65.0(1), B2−B1−B3 61.1(1), N2−B2−N5 116.9(2).

### 2.2 On the Structure and Bonding in the B_3_ Rings

Crystals suitable for a structural analysis by SC‐XRD were grown from a toluene solution at –32°C. Figure 4b sketches the solid‐state structure of the cation **TB2** ^+^ in this new compound. The BH_2_ plane is approximately perpendicular to the B_3_ ring plane. This orientation allows for B–H···B interactions with the vacant orbital at the BH_2_ unit. The B_3_ ring forms a non‐isosceles triangle; the B1─B3 bond (1.715(3) Å) is significantly shorter than the B2─B3 bond (1.861(3) Å). Consequently, the B─H···B interactions are unsymmetric, too.

The BH_2_ unit most likely moves from its location close to one B atom in the [B_2_(hpp)_2_] unit to the energetically equal position closer to the other boron atom. Quantum‐chemical calculations at the B3LYP+D3/def2‐TZVP level of theory found a C_2_‐symmetric transition state with an isosceles B_3_ triangle for such a movement. Small positive and negative displacements along the imaginary vibrational mode lead to two energetically equivalent minima (see Figure 5a). The displacement of the apical BH_2_ unit from the position above the midpoint of the basal B─B bond amounts to 0.1 Å in these minima (Figure 5b). The energy‐minimum structures are only 0.9 kJ mol^−^^1^ lower in energy than the transition state, arguing for rapid movement of the apical BH_2_ unit and a dynamic interchange between two equivalent B─H···B interactions. A similar degree of distortion from an isosceles triangle is observed in the experimentally derived structures. The displacement of the apical boron atom from the position above the midpoint of the B─B bond amounts to 0.03 Å for **TB1**[OTf], 0.05 Å for **TB2**[B(cat)_2_], and 0.14 Å for **TB2**[HB(C_6_F_5_)_3_] (Figure 5c). The close agreement with the calculated value of 0.10 Å further emphasizes the flexible position of the apical BH_2_ unit.

**FIGURE 5:**
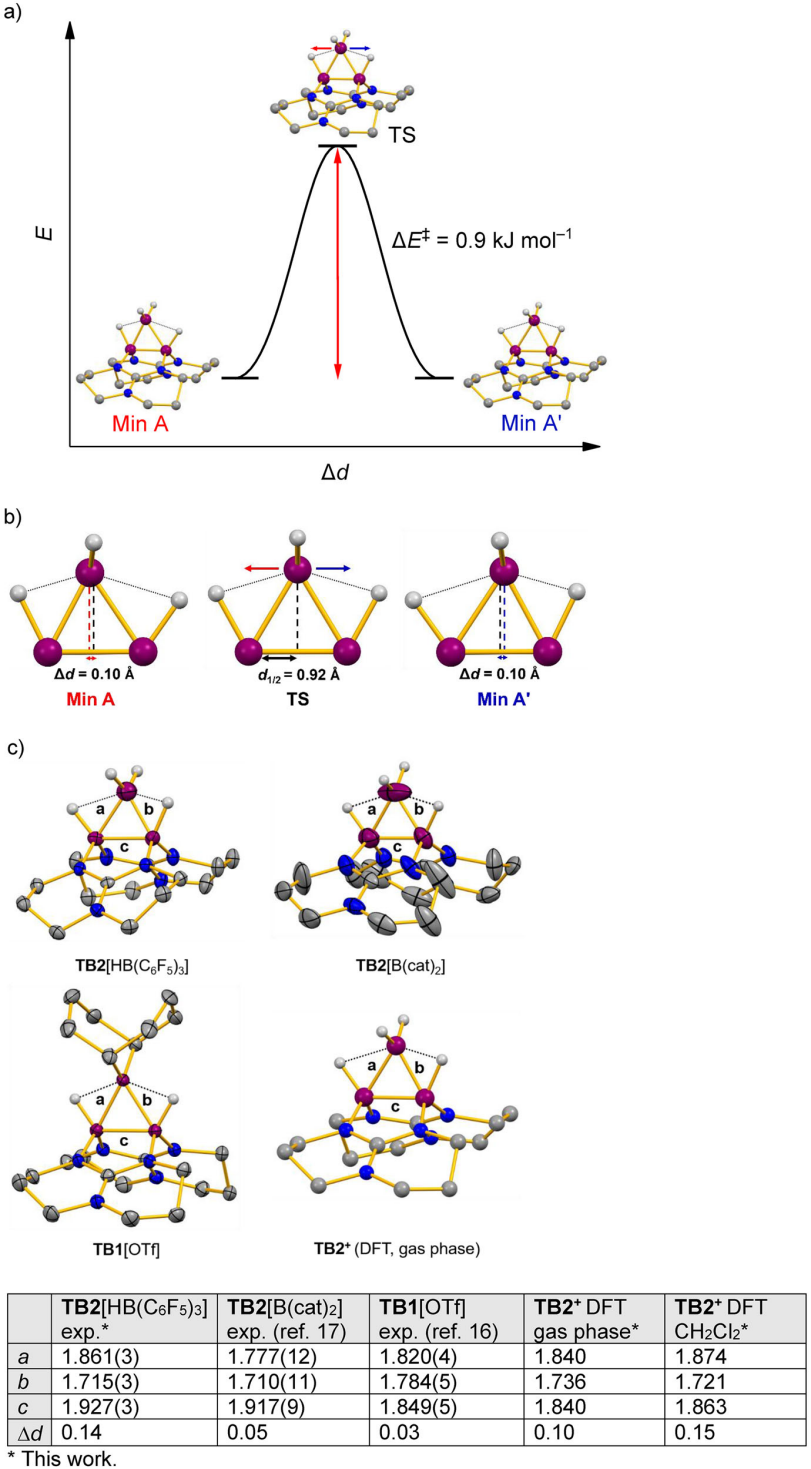
(a) Transition state (TS) and minima (Min A and Min A’) identified along the imaginary mode from B3LYP+D3/def2‐TZVP calculations (gas phase). (b) Visualization of the displacement of the apical boron atom from the position above the midpoint of the diborane B─B bond. (c) Comparison of selected structural parameters (in Å) for cationic σ‐aromatic B_3_ rings in the solid state (exp. values) and calculated with B3LYP+D3/def2‐TZVP.

The basal B─B bond between the hpp‐bridged boron atoms in **TB2**[HB(C_6_F_5_)_3_] measures 1.927(3) Å, being considerably elongated with respect to the B─B bond in [HB(hpp)]_2_ (1.772(3) Å) [23]. The SC‐XRD structures of **TB2**[HB(C_6_F_5_)_3_] and **TB2**[B(cat)_2_] exhibit basal B–B bond lengths of 1.927(3) and 1.917(9) Å, respectively. By contrast, the structure calculated with B3LYP+D3/def2‐TZVP for the isolated cation **TB2** ^+^ exhibits a shorter value of 1.840 Å. Notably, a similarly short basal B–B bond length of 1.820(4) Å was observed experimentally for the B_3_ ring in **TB1**[OTf] (Figure 5c). To obtain more information, quantum‐chemical calculations on **TB2** ^+^ were carried out in which the energy was followed as a function of the basal B–B bond length. These calculations found a remarkably shallow potential energy curve along this coordinate (Figure 6). Elongation of the basal B–B bond from 1.847 Å (calculated energy minimum value) to 1.927 Å (SC‐XRD value for new **TB2**[HB(C_6_F_5_)_3_]) resulted in an energy increase of only ca. 2.9 kJ mol^−^^1^. Inclusion of the solvent effect using the Conductor‐like Polarizable Continuum Model (CPCM, for dichloromethane) further reduced this energy difference to approximately 1.6 kJ mol^−^^1^ and shifted the minimum of the potential energy curve toward longer B–B distances (Figure 6). Consequently, variations in the basal B–B bond length lead only to small energy changes, in line with the delocalized, electron deficient bonding within the B_3_ ring.

**FIGURE 6:**
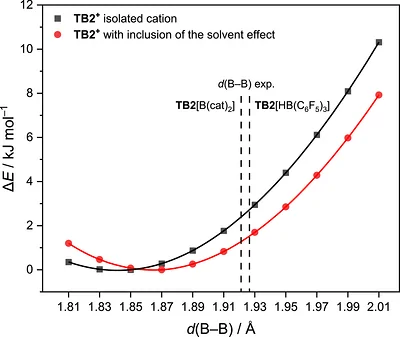
Relative energy Δ*E* as a function of the basal B─B bond length from B3LYP+D3/def2‐TZVP calculations (constrained geometry optimizations) without (gas phase, black curve) and with (red curve) inclusion of the solvent effect (CPCM, CH_2_Cl_2_).

The ^11^B NMR spectrum of **TB2**[HB(C_6_F_5_)_3_] showed a doublet at *δ* = −1.16 ppm (^1^ *J*_BH_ = 91 Hz), together with the characteristic signal of the [HB(C_6_F_5_)_3_]^−^ counterion at *δ* = −25.44 ppm (^1^ *J*_BH_ = 106 Hz) [25]. For comparison, a doublet at *δ* = −0.39 ppm (^1^ *J*_BH_ = 78 Hz) and a singlet at *δ* = 14.25 ppm due to the [B(cat)_2_]^−^ counterion appeared in the ^11^B NMR spectrum of **TB2**[B(cat)_2_] [17]. The low‐field doublet is assigned to the two basal boron atoms based on its 2:1 integral ratio relative to the counterion signal and its close agreement with the ^11^B NMR signal for the literature‐known B_3_ ring **TB1**[OTf] (doublet at *δ* = −1.73 ppm (^1^ *J*_BH_ = 80 Hz) together with an extremely broad resonance for the apical 9‐BBN boron atom at *δ* = 39.05 ppm) [16]. A signal from the apical BH_2_ unit is too broad to be clearly visible in the ^11^B NMR spectrum of **TB2** ^+^, due to the molecular dynamics (movement of the apical BH_2_ unit, Figure 5b) in combination with line broadening resulting from efficient quadrupolar relaxation of the ^11^B nucleus. Consistently, the ^1^H{^11^B} NMR spectra of both **TB2** ^+^ salts show only a single B−H resonance integrating to two hydrogen atoms (*δ* = 2.28–2.29 and 2.34–2.58 ppm), which is therefore assigned to the two basal B–H groups. ^11^B NMR measurements at high temperature to reduce the line broadening due to quadrupolar relaxation pathways (see the results obtained for **DB1** [26]) are not possible, since **TB2** ^+^ starts to decompose above temperatures of 60°C.

We also recorded IR spectra for solid **TB2**[B(cat)_2_] and **TB2**[HB(C_6_F_5_)_3_]. The IR spectrum of **TB2**[B(cat)_2_] shows four bands in the region characteristic for stretching modes ν(B–H) at 2560, 2486, 2429, and 2318 cm^−^^1^ (Figure 7). In good agreement with the experiments, quantum‐chemical calculations (B3LYP+D3/def2‐TZVP) predict the two B–H stretching modes of the apical BH_2_ unit at 2694 and 2604 cm^−^^1^, and the two modes of the basal B–H oscillators at 2437 and 2179 cm^−^^1^. For **TB2**[HB(C_6_F_5_)_3_], the observed B–H stretching bands overlap with the characteristic B–H stretching vibrations of the [HB(C_6_F_5_)_3_]^−^ anion [25, 27], prohibiting a clear assignment of the B–H stretching modes from the cationic B_3_ ring.

**FIGURE 7:**
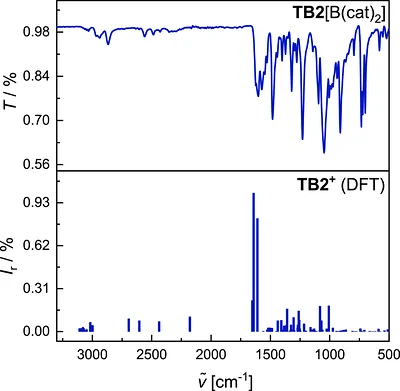
Experimental IR spectrum of **TB2**[B(cat)_2_] and illustration of the calculated vibrational properties (with normalized intensity) from B3LYP+D3/def2‐TZVP calculations on the isolated cation.

### 2.3 On the Scope of the New Synthetic Route to σ‐Aromatic B_3_ Rings

To demonstrate the scope of the new synthetic approach, **DB2**[HB(C_6_F_5_)_3_] was reacted with several other hydroboranes. The reactions were again carried out in dichloromethane at room temperature with two equivalents of the hydroborane. Reaction with HBCy_2_ (Cy = cyclohexyl) yielded the new σ‐aromatic B_3_ ring compound **TB3**[HB(C_6_F_5_)_3_]. A sharp doublet at *δ* = ─0.78 ppm (^1^J = 82 Hz) was assigned to the two B‐H boron nuclei, and a broad singlet around *δ* = 38 ppm to the boron of the apical BCy_2_ unit of **TB3** ^+^. Another doublet signal in the ^11^B NMR spectrum at *δ* = ─25.45 ppm (^1^J = 95 Hz) belongs to the [HB(C_6_F_5_)_3_]^−^ counterion. The product was isolated in pure form in a yield of 48% after a reaction time of 5 d and removal of the HBCy_2_·PCy_3_ by‐product (**Scheme** **3**). The NMR spectra also indicated the formation of B_3_ rings in the reactions of **DB2**[HB(C_6_F_5_)_3_] with catecholatoborane (HBcat) and pinacolatoborane (HBpin). In the ^11^B NMR spectra (see Supporting Information), a doublet signal at *δ* = ─1.07 ppm (d, ^1^J = 134 Hz) was assigned to the two B‐H boron atoms in the B_3_ ring with B(pin), and a doublet signal at *δ* = ─1.04 ppm (d, ^1^J = 128 Hz) to the two B‐H boron atoms in the B_3_ ring with B(cat)). However, the reactions with HB(pin) and HB(cat) proceeded slowly at room temperature, and the resulting B_3_ rings showed signs of decomposition during the workup procedure, hampering the isolation of the B_3_ rings in pure form. More work is needed to optimize the reaction conditions for their synthesis. The successful synthesis of **TB3**[HB(C_6_F_5_)_3_] demonstrates that the present synthetic strategy can be extended beyond H_3_B·SMe_2_, highlighting the suitability of **DB2^+^** as a precursor to σ‐aromatic B_3_ rings.

**SCHEME 3:**
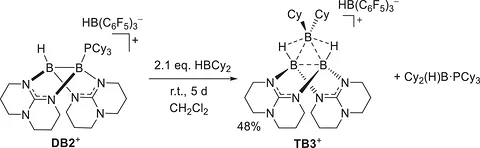
Reaction of **DB2**[HB(C_6_F_5_)_3_] with HBCy_2_ (2.1 eq.) to give **TB3**[HB(C_6_F_5_)_3_].

### 2.4 B_3_ Ring Disassembly to Give Cationic Diboranes and Neutral Monoboranes

Next, we tested the possibility to disassemble the B_3_ ring by reaction with a Lewis base. We first carried out some quantum‐chemical calculations to identify suitable bases to split the B_3_ ring to give a base‐stabilized cationic diborane and a base‐BH_3_ adduct. The calculations were carried out without (gas phase) and with inclusion of the solvent effect (dichloromethane). Some results of these calculations are illustrated in Figure 8 (see Supporting Information for further details). Reactions with simple phosphorus or nitrogen bases, PCy_3_ and NEt_3_, in the gas‐phase gave positive Δ*G* values of +16.6 and +18.2 kJ mol^−^^1^, respectively, indicating that B_3_ ring cleavage is thermodynamically unfavorable in these cases. The corresponding Gibbs free energies with consideration of the solvent effect are +34.4 and +15.2 kJ mol^−^^1^. On the other hand, reaction with pyridine bases led to negative Δ*G* values in the calculations (see Figure 8). The reaction Gibbs free energy varies with the substituent at the pyridine. Calculated Δ*G* values for the gas‐phase reactions amount to −39.3 kJ mol^−^^1^ for 4‐trifluoromethylpyridine, −62.5 kJ mol^−^^1^ for pyridine, and −105.2 kJ mol^−^^1^ for 4‐dimethylaminopyridine (DMAP), while the corresponding Δ*G* values with inclusion of the solvent effect are −44.7, −61.0, and −91.9 kJ mol^−^^1^. These results demonstrate a substantial influence of the pyridine substituent on the thermodynamics of the B_3_ ring cleavage. Obviously, electron‐withdrawing substituents, weakening the interaction with the cationic diborane and with the monoborane, lead to less negative Δ*G* values.

**FIGURE 8:**
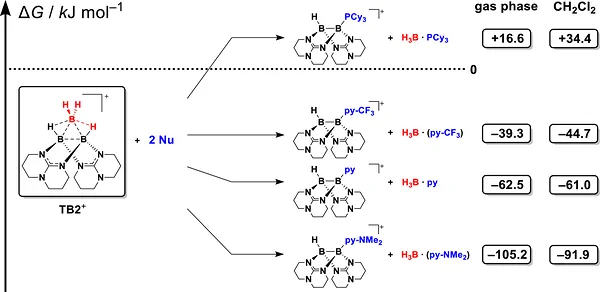
Gibbs free energy changes Δ*G* for the reaction of **TB2** ^+^ with two equivalents of a Lewis base (Nu) to give the base‐stabilized cationic diborane and the neutral base‐stabilized monoborane, calculated at the B3LYP+D3/def2‐TZVP level with and without inclusion of the solvent effect (CH_2_Cl_2_).

Based on these predictions from theory, we experimentally tested the base‐induced B_3_ ring disassembly with pyridine bases. For practical reasons, these experiments were carried out with **TB2**[B(cat)_2_], since the products with [B(cat)_2_]^−^ counterion generally crystallized better. Indeed, the experiments showed that the B_3_ ring can be selectively disassembled with pyridine, and the derivatives 4‐trifluoromethylpyridine and 4‐dimethylaminopyridine (DMAP) (Figure 9a). Electron‐withdrawing substituents at the pyridine increase the reaction time. Reaction of 4‐trifluoromethylpyridine with the B_3_ ring required ca. 3 d for completion, while reaction of DMAP was completed within 1 h, and reaction of pyridine within 2 h. The resulting DMAP‐ and 4‐trifluormethylpyridine‐substituted cationic diboranes were crystallized from dichloromethane layered with *n*‐pentane, and structurally characterized by SC‐XRD analysis. As expected, the structures of the two monocationic diboranes **DB5** ^+^ and **DB7** ^+^ are quite similar (Figure 9b). The compounds exhibit slightly shorter B─B bond lengths of 1.741(9) Å (for **DB5** ^+^) and 1.737(6) Å (for **DB7** ^+^), compared to the neutral dihydridodiborane **DB1** (1.773(4) Å) [23]. The B─N bond length amounts to 1.594(7) Å for **DB5** ^+^ and 1.606(5) Å for **DB7** ^+^, with the more electron‐deficient pyridine derivative exhibiting a slightly longer B─N bond. The angle between the pyridine plane and the BBN plane measures 95.4(4)° for **DB5** ^+^ and 95.9(5)° for **DB7** ^+^, while the dihedral angle defined by the BBH and BBN planes amounts to 2.0° and 4(3)°, respectively. In the IR spectra, characteristic B–H stretching vibrations are observed at 2293 cm^−^^1^ (**DB5** ^+^), 2323 cm^−^^1^ (**DB6** ^+^), and 2359 cm^−^^1^ (**DB7** ^+^). Although shifts of the ν(B–H) band are apparent, no clear correlation with the electronic properties of the coordinated pyridine ligand is evident. The ^11^B NMR spectra show two boron signals for the asymmetric cationic diborane species. For **DB5** ^+^, resonances are observed at *δ* = 4.86 and −2.99 ppm for the compound with [HB(C_6_F_5_)_3_]^−^ counterion, and at *δ* = 4.16 and −3.54 ppm for the compound with [B(cat)_2_]^−^ counterion. Likewise, **DB6** ^+^ exhibits resonances at *δ* = 5.36 and −2.77 ppm ([HB(C_6_F_5_)_3_]^−^) and *δ* = 5.22 and −2.60 ppm ([B(cat)_2_]^−^). Overall, only minor variations in the chemical shifts were observed.

**FIGURE 9:**
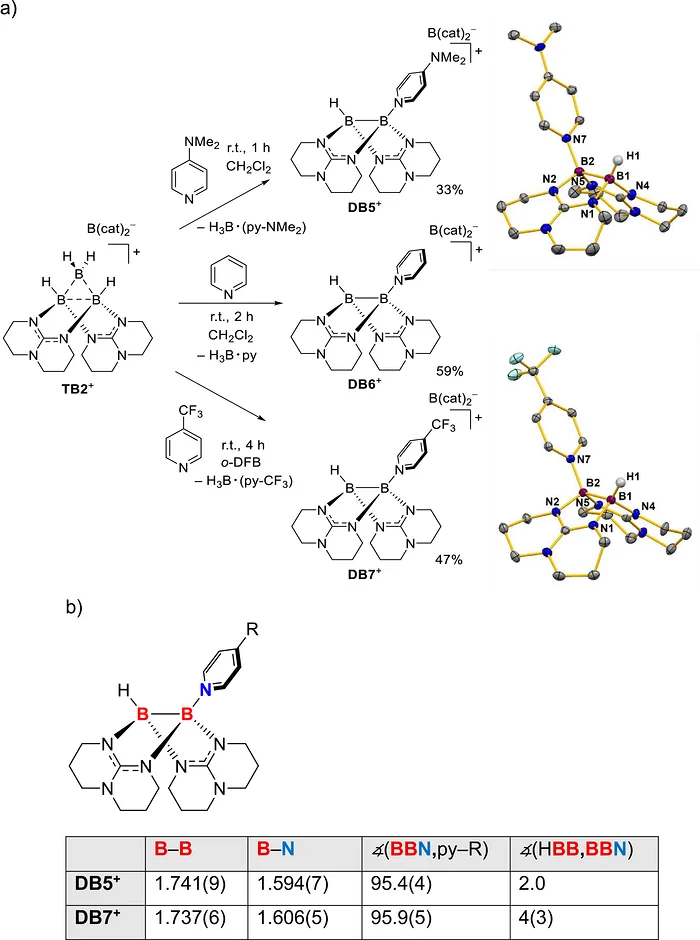
(a) Synthesis of the base‐stabilized cationic diboranes **DB5** ^+^, **DB6** ^+^, and **DB7** ^+^ through base‐induced disassembly of **TB2**[B(cat)_2_]. Illustration of the structures of **DB5** ^+^ and **DB7** ^+^ in the solid state are included. Displacement ellipsoids drawn at the 50% probability level. C─H hydrogens and counterions are omitted. Color code: B violet, C grey, N blue, F light blue. (b) Comparison of selected structural parameters (bond length in Å, angles in °) of **DB5** ^+^ (R = NMe_2_) and **DB7** ^+^ (R = CF_3_) in the solid state.

The three cations **DB5** ^+^, **DB6** ^+^, and **DB7** ^+^ differ in their color and UV/Vis spectra (Figure 10a). A systematic bathochromic shift of the lowest‐energy band in the UV/Vis spectra was observed across the series, with *λ*_max_ increasing from 315 nm (**DB5** ^+^) to 341 nm (**DB6** ^+^) and 384 nm (**DB7** ^+^). TD‐DFT calculations at the CAM‐B3LYP+D3/def2‐TZVP/CPCM(DCM) level of theory predict the lowest‐energy singlet excitations at 266, 294, and 323 nm for **DB5** ^+^, **DB6** ^+^, and **DB7** ^+^, respectively. In all three cases, the excitation into the corresponding *S*_1_ state can be described as HOMO→LUMO transition. Electron density difference plots reveal spatially separated regions of electron density depletion on the diborane fragment and electron density accumulation on the pyridine unit upon excitation (Figure 10b), in line with a pronounced charge‐transfer character. Hence, the lowest‐energy excitation (HOMO → LUMO) can be described as an excitation from the B‐B bonding orbital into the pyridine π* acceptor orbital. While the calculated excitation energies are systematically blue‐shifted relative to the experimental absorption maxima, the observed bathochromic shift with increasing electron‐withdrawing properties of the substituents at the pyridine, is well reproduced by the calculations.

**FIGURE 10:**
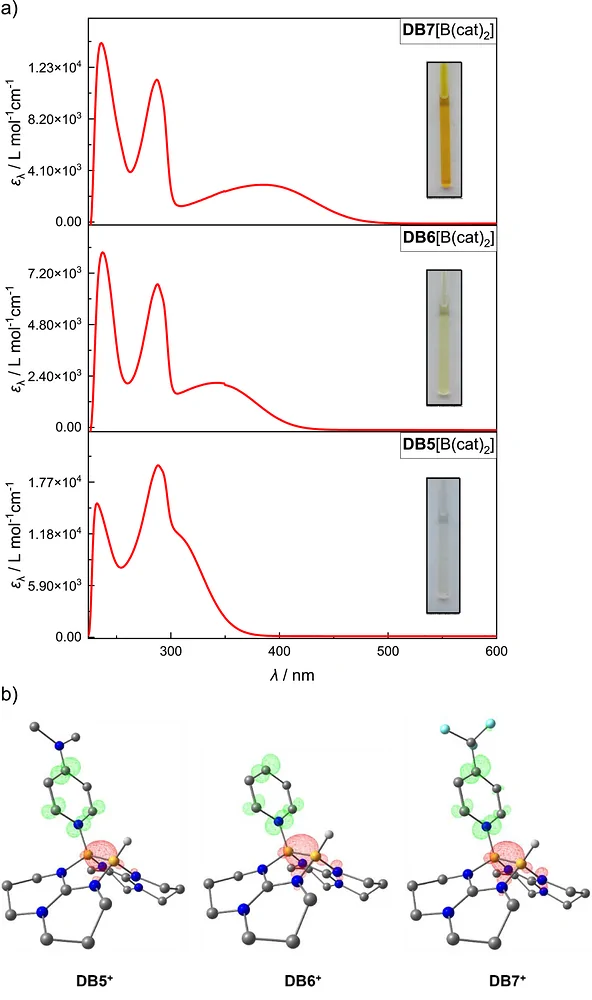
(a) Colors and UV/Vis spectra of the cationic diboranes **DB5** ^+^ (c = 4.62·10^−5^ mol L^−1^), **DB6** ^+^ (c = 6.76·10^−5^ mol L^−1^), and **DB7** ^+^ (c = 4.29·10^−5^ mol L^−1^) in CH_2_Cl_2_ solutions. (b) Electron density difference plots associated with the lowest‐energy singlet excitation (*S*_0_→S_1_) of compounds **DB5** ^+^, **DB6** ^+^, and **DB7** ^+^, calculated with CAM‐B3LYP+D3/def2‐TZVP/CPCM(CH_2_Cl_2_). Regions of electron density depletion in red, and regions of electron density accumulation in green.

Finally, we tested the reassembly of the B_3_ ring from the pyridine‐stabilized cationic diborane **DB7** ^+^ with 4‐trifluoromethylpyridine‐stabilized diborane. Indeed, reaction of **DB7** ^+^ with H_3_B·SMe_2_ yielded the B_3_ ring compound according to Figure 11a. The yield of the B_3_ ring compound was estimated to ca. 77% after 2 h reaction time at room temperature from ^11^B NMR signal integration. The formation of a significant amount of H_3_B·(py‐CF_3_) (characteristic signal at *δ* = 11.4 ppm in the ^11^B NMR spectrum) is not observed, most likely due to the low basicity of 4‐trifluoromethylpyridine. Only small signals from side‐products were detected, giving rise to a singlet at *δ* = 6.90 ppm and a doublet at *δ* = 28.88 ppm in the ^11^B NMR spectrum (Figure 11b). These minor signals may originate from decomposition processes. Nevertheless, the experiments clearly show that the pyridine‐stabilized cationic diborane can indeed be reassembled efficiently to the B_3_ ring.

**FIGURE 11:**
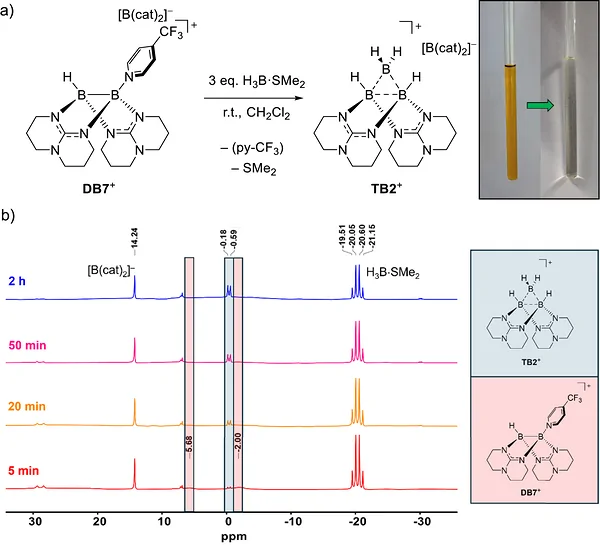
(a) Reaction of **DB7**[B(cat)_2_] with excess H_3_B·SMe_2_ (3.0 eq.) to give the cationic triboron compound **TB2**[B(cat)_2_]. (b) Stacked ^11^B NMR spectra showing the conversion to the triborirane **TB2**[B(cat)_2_] in CD_2_Cl_2_ solution.

## 3 Conclusions

In this work, electron‐deficient, σ‐aromatic cationic B_3_ rings are synthesized following a novel synthetic strategy by reacting cationic diboranes, stabilized by a weakly‐bound base, with a monoborane adduct. In addition, the first base‐induced disassembly reactions of cationic σ‐aromatic B_3_ rings are reported, leading back to cationic diboranes and monoboranes. In these reactions, electron‐precise (2c,2e) B─B bonds are reversibly interconverted into electron‐deficient (3c,2e) B_3_ bonds. The bond properties in the B_3_ ring compounds were analyzed by combining experimental results with quantum‐chemical calculations. Dynamic processes within the B_3_ ring were identified, leading to dynamic distortions that affect the NMR spectra.

This work provides first of all a novel basis for the directed assembly and disassembly of σ‐aromatic B_3_ ring compounds with (3c,2e) bonds from diboron and monoboron building blocks. Instead of the highly reactive borinium ion source used in previous work, a more readily available monoborane‐adduct is used here as starting compound. In addition, the base‐induced disassembly of the B_3_ rings can be used for the synthesis of new unsymmetric cationic hydridodiboranes. Finally, the results might lead the way to the use of σ‐aromatic B_3_ rings for the stabilization of monoboranes, that are otherwise prone to dismutation reactions. Base‐induced release of these monoboranes in situ upon base addition might open up new opportunities in hydroboration reactions. Research in this direction is currently on the way.

## Conflicts of Interest

The authors declare no conflicts of interest.

## Supporting information



The authors have cited additional references within the Supporting Information [28, 29, 30, 31, 32, 33, 34, 35, 36, 37, 38, 39, 40, 41, 42, 43, 44, 45, 46, 47, 48, 49, 50, 51, 52, 53, 54].
**Supporting File 1**: chem71615‐sup‐0001‐SuppMat.pdf

## Data Availability

The data that support the findings of this study are available in the supplementary material of this article.
