# Supplementary material for: Reversible Assembly and Disassembly of σ‐Aromatic B3 Rings
Source: Chemistry. 2026 Aug 25;32(42):e71615. doi: 10.1002/chem.71615 (PMC13755137; doi:10.1002/chem.71615)
Supplement: Supplementary file 1 [file CHEM-32-e71615-s001.pdf]

# Supporting Information

## Table of contents

1. General details of the synthetic work ........ 1

2. Experimental procedures and analytical data ........ 3

2.1 Phosphine substituted diboranes ........ 3

2.1.1 [$(PCy_3)(H)\{B(\mu$-hpp$)\}_2][HB(C_6F_5)_3]$ **DB2**$[HB(C_6F_5)_3]$ ........ 3

2.1.2 [(dcpe)$\{B(\mu$-hpp$)\}_2$][OTf]$_2$ **DB4**[OTf]$_2$ ........ 10

2.2 Triboriranes ........ 15

2.2.1 [$\{HB(\mu$-hpp$)\}_2(\mu$-$BH_2)$][B(cat)$_2$] **TB2**[B(cat)$_2$] ........ 15

2.2.2 [$\{HB(\mu$-hpp$)\}_2(\mu$-$BH_2)][HB(C_6F_5)_3]$ **TB2**$[HB(C_6F_5)_3]$ ........ 19

2.2.3 [$\{HB(\mu$-hpp$)\}_2(\mu$-$BCy_2)][HB(C_6F_5)_3]$ **TB3**$[HB(C_6F_5)_3]$ ........ 24

2.2.4 [$\{HB(\mu$-hpp$)\}_2(\mu$-BPin)]$[HB(C_6F_5)_3]$ **TB4**$[HB(C_6F_5)_3]$ ........ 28

2.2.5 [$\{HB(\mu$-hpp$)\}_2(\mu$-BCat)]$[HB(C_6F_5)_3]$ **TB5**$[HB(C_6F_5)_3]$ ........ 29

2.2.4 [$\{HB(\mu$-hpp$)\}_2(\mu$-$BH_2)$][B(cat)$_2$] **TB2**[B(cat)$_2$] ........ 30

2.3 *N*-heterocyclic ligand substituted diboranes ........ 32

2.3.1 [(DMAP)(H)$\{B(\mu$-hpp$)\}_2][HB(C_6F_5)_3]$ **DB5**$[HB(C_6F_5)_3]$ ........ 32

2.3.2 [(Py)(H)$\{B(\mu$-hpp$)\}_2][HB(C_6F_5)_3]$ **DB6**$[HB(C_6F_5)_3]$ ........ 37

2.3.3 [(DMAP)(H)$\{B(\mu$-hpp$)\}_2$][B(cat)$_2$] **DB5**[B(cat)$_2$] ........ 41

2.3.4 [(Py)(H)$\{B(\mu$-hpp$)\}_2$][B(cat)$_2$] **DB6**[B(cat)$_2$] ........ 45

2.3.5 [($CF_3$-Py)(H)$\{B(\mu$-hpp$)\}_2$][B(cat)$_2$] **DB7**[B(cat)$_2$] ........ 49

2.4 Reference Spectra ........ 55

2.4.1 Tricyclohexylphosphine $PCy_3$ ........ 55

2.4.2 1,2-bis(dicyclohexylphosphino)ethane (dcpe) ........ 55

3 Details of the crystal structure determination ........ 56

4 Details of the quantum-chemical calculations ........ 64

5 References ........ 95

# 1. General details of the synthetic work

All reactions were carried out under a dry argon atmosphere by using standard Schlenk technique. The compound **1** (hpp = 1,3,4,6,7,8-hexahydro-2*H*-pyrimido[1,2-*a*]pyrimidinate) was synthesized according to the literature.[1−6] All solvents were rigorously dried by the solvent purification system MB SPS-800 MBRAUN and stored over molecular sieves (3 Å and 4 Å) after being degassed by the freeze-pump-thaw method.

BRUKER Avance DPX 200, BRUKER Avance II 400 and BRUKER Avance III 600 devices were used for NMR spectroscopy. NMR spectra were recorded at 298 K unless otherwise stated and all chemical shifts are given in ppm relative to $Si(CH)_4$ ($^{1}H$, $^{13}C$), $BF_3{\cdot}Et_2O$ ($^{11}B$), $CFCl_3$ ($^{11}F$) and H3PO4 (85% in $H_2O$) ($^{31}P$). Infrared spectra were recorded as powder with a Bruker Alpha Platinum-ATR. Mass spectra were obtained with a JOEL AccuTOF GCx Instrument and a BRUKER ApexQe FT-ICR Instrument at the MS laboratory of the University of Heidelberg. Elemental analyses were performed at the Microanalytical Laboratory of the University of Heidelberg using the vario EL and vario MICRO cube devices from Elementar Analysensysteme GmbH.

Crystal data and details of the data collections, molecular structures, bond lengths and angles are given in section 3. "Crystal structure determination". Suitable crystals for single-crystal structure determination were taken directly from the mother liquor, taken up in perfluorinated polyether oil and fixed on a cryo loop. Full shells of intensity data were collected at low temperature with a Bruker D8 Venture, dual source (Mo- or Cu-$K_\alpha$ radiation, microfocus X-ray tube, Photon III Detector). Data were processed with the standard Bruker (SAINT, APEX3/4/6) software package.[7] Multiscan absorption correction was applied using the SADABS program.[8,9] The structures were solved by intrinsic phasing[10,11] and refined using the SHELXTL software package (Version 2018/3).[12-14] Graphical handling of the structural data during solution and refinement were performed with OLEX2.[15] All non-hydrogen atoms were given anisotropic displacement parameters. Hydrogen atoms bound to carbon were input at calculated positions and refined with a riding model. Hydrogen atoms bound to boron or nitrogen were located in difference Fourier syntheses and refined, either fully or with appropriate distance and/or symmetry. Split atom models were used to refine disordered groups and/or solvent molecules. When found necessary, suitable geometry and adp restraints were applied.[16-18] Due to severe disorder and fractional occupancy, electron density attributed to the solvent of crystallization was removed from some of the structures with the BYPASS procedure,[19] as implemented in PLATON (squeeze/hybrid). The visualization of the structures was performed using Mercury 4.2.0. Displacement ellipsoids are depicted with 50% probability.[20] CCDC 2566210, 2566211, 2566212 and 2566213 contain the supplementary

crystallographic data for this paper. These data can be obtained free of charge from the Cambridge Crystallographic Data Centre *via* www.ccdc.cam.ac.uk/data_request/cif.

# 2. Experimental procedures and analytical data

## 2.1 Phosphine substituted diboranes

### 2.1.1 [(PCy$_3$)(H){B($\mu$-hpp)}$_2$][HB(C$_6$F$_5$)$_3$] **DB2**[HB(C$_6$F$_5$)$_3$]

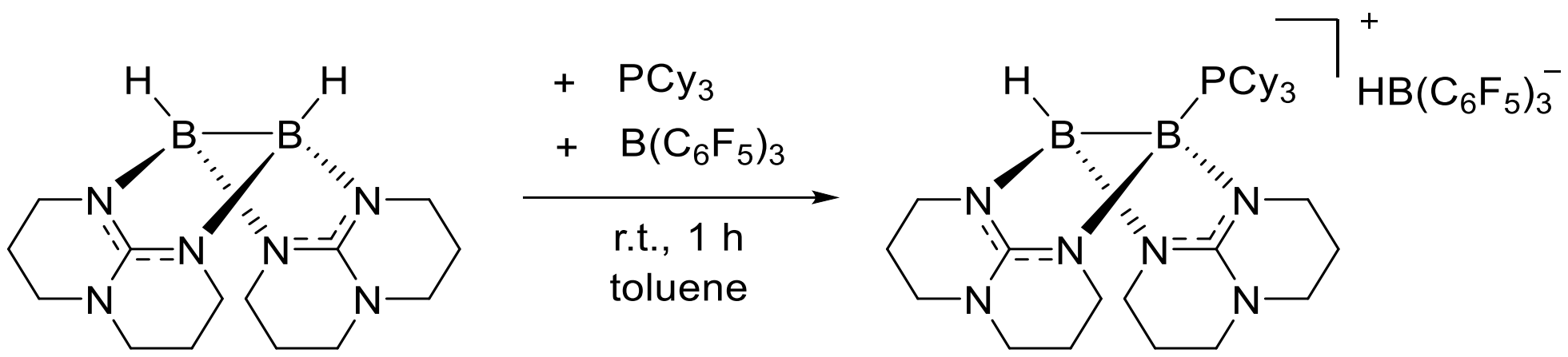

In a Schlenk flask, 300 mg of **DB1** (1.00 mmol, 1.00 eq.) and 280 mg of tricyclohexylphosphine (1.00 mmol, 1.00 eq.) were suspended in 5 mL toluene. In a second Schlenk flask, 512 mg of tris(pentafluorophenyl)borane (1.00 µmol, 1.00 eq.) was dissolved in 5 mL toluene. The tris(pentafluorophenyl)borane solution was added dropwise to the suspension while stirring vigorously. After one hour of stirring the toluene supernatant was removed *via* syringe and the oily residue was washed with toluene (3 x 3 mL) and *n*-pentane (3 x 3 mL). The solid residue was redissolved in 1 ml of dichloromethane and stored at –20 °C overnight. **DB2**[HB(C$_6$F$_5$)$_3$] was obtained in a yield of 721 mg (660 µmol, 66%) as a crystalline colorless solid.

**$^1$H NMR (600 MHz, CD$_2$Cl$_2$):**

$\delta$ = 3.40–3.04 (m, 16H, $H_{hpp}$), 2.09 (q, $^3J_{HH}$ = 12 Hz, 3H, $H_{Cy\text{-}CH}$), 1.93–1.73 (m, 24H, $H_{hpp}$), 1.40–1.22 (m, 14H, $H_{Cy\text{-}CH2}$) ppm.

**$^1$H{$^{11}$B} NMR (400 MHz, CD$_2$Cl$_2$):**

$\delta$ = 3.39 (bs, 2H, $H_{HBCF}$), 3.30–2.89 (m, 16H, $H_{hpp}$), 1.98 (q, $^3J_{HH}$ = 11 Hz, 3H, $H_{Cy\text{-}CH}$), 1.77–1.47 (m, 16H, $H_{hpp}$,$H_{Cy\text{-}CH2}$), 1.29–1.04 (m, 24H, $H_{Cy\text{-}CH2}$) ppm.

**$^{13}$C{$^1$H} NMR (151 MHz, CD$_2$Cl$_2$):**

$\delta$ = 158.0 ($C_{q\text{-}hpp}$), 149.3–147.8 (m, $C_{HBCF}$), 139.0–135.9 (m, $C_{HBCF}$), 47.6 ($C_{hpp}$), 47.4 ($C_{hpp}$), 45.9 ($C_{hpp}$), 45.0 ($C_{hpp}$), 32.8 (d, $^1J_{PC}$ = 28 Hz, $C_{Cy\text{-}CH}$), 28.8 (d, $^3J_{PC}$ = 2 Hz, $C_{Cy\text{-}CH2}$), 27.9 (d, $^2J_{PC}$ = 10 Hz, $C_{Cy\text{-}CH2}$), 26.6 (d, $^3J_{PC}$ = 1 Hz, $C_{Cy\text{-}CH2}$), 23.1 ($C_{hpp}$), 22.6 ($C_{hpp}$) ppm.

**$^{11}$B NMR (128 MHz, CD$_2$Cl$_2$):**

$\delta$ = –1.89 (bs, 2B), –25.46 (d, $^1J_{BH}$ = 92 Hz, 1B, $B_{HBCF}$) ppm.

**$^{11}$B{$^1$H} NMR (128 MHz, CD$_2$Cl$_2$):**

$\delta$ = –2.26 (bs, 2B), –25.46 (s, 1B, $B_{HBCF}$) ppm.

**$^{19}$F NMR (377 MHz, CD$_2$Cl$_2$):**

$\delta$ = –134.00 (d, $^3J_{FF}$ = 24 Hz, 6F, $F_{HBCF}$), –164.85 (t, $^3J_{FF}$ = 20 Hz, 3F, $F_{HBCF}$), –167.65–167.78 (m, 6F, $F_{HBCF}$) ppm.

**$^{31}$P NMR (162 MHz, $CD_2Cl_2$):**

$\delta$ = 5.63 (bs, 1P, $P_{PCy3}$) ppm.

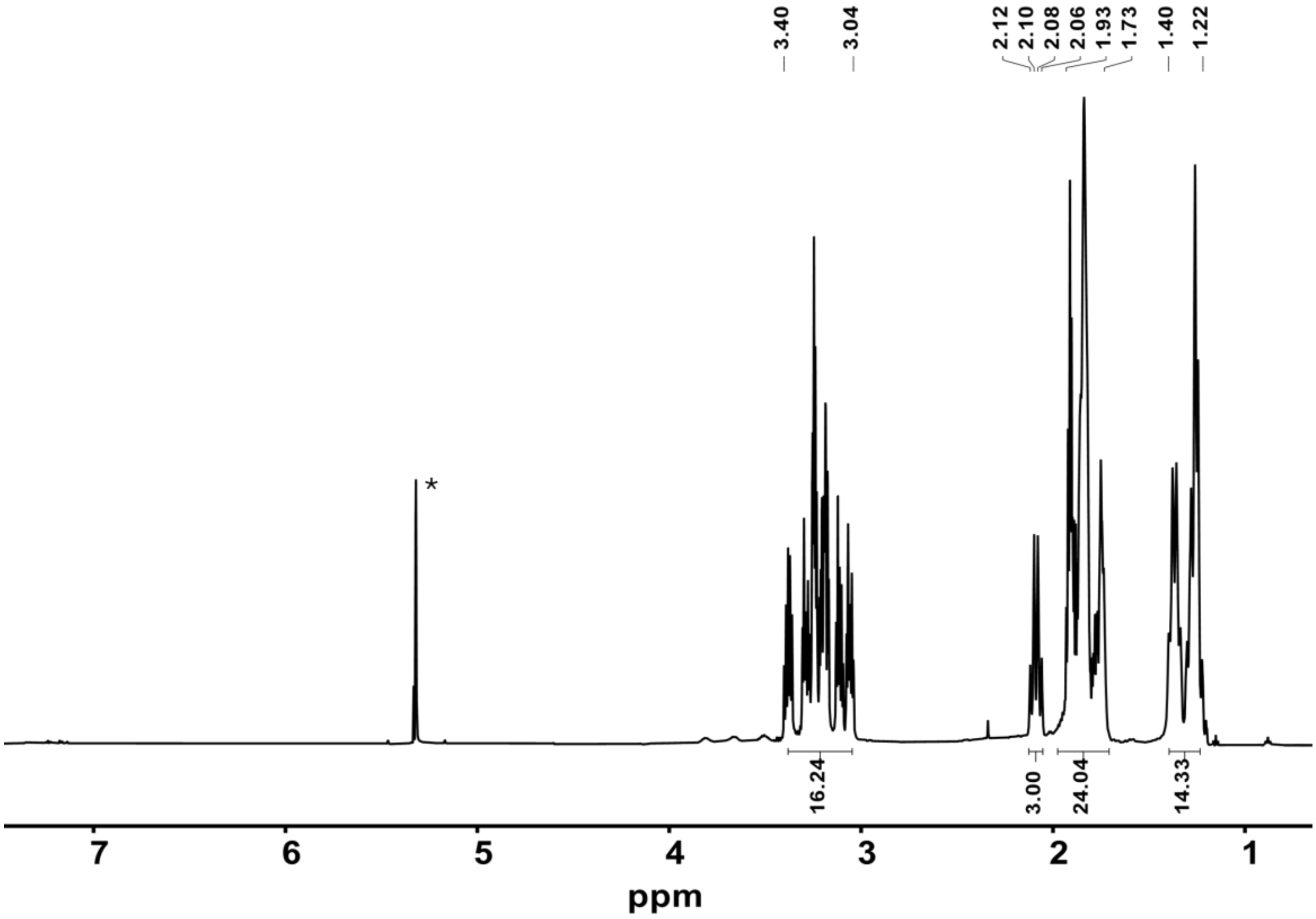


***Figure S1.*** *$^1$H NMR spectrum (600 MHz) of [($PCy_3$)(H){B(µ-hpp)}$_2$][HB($C_6F_5$)$_3$] in $CD_2Cl_2$.The asterisk * denotes a solvent signal.*

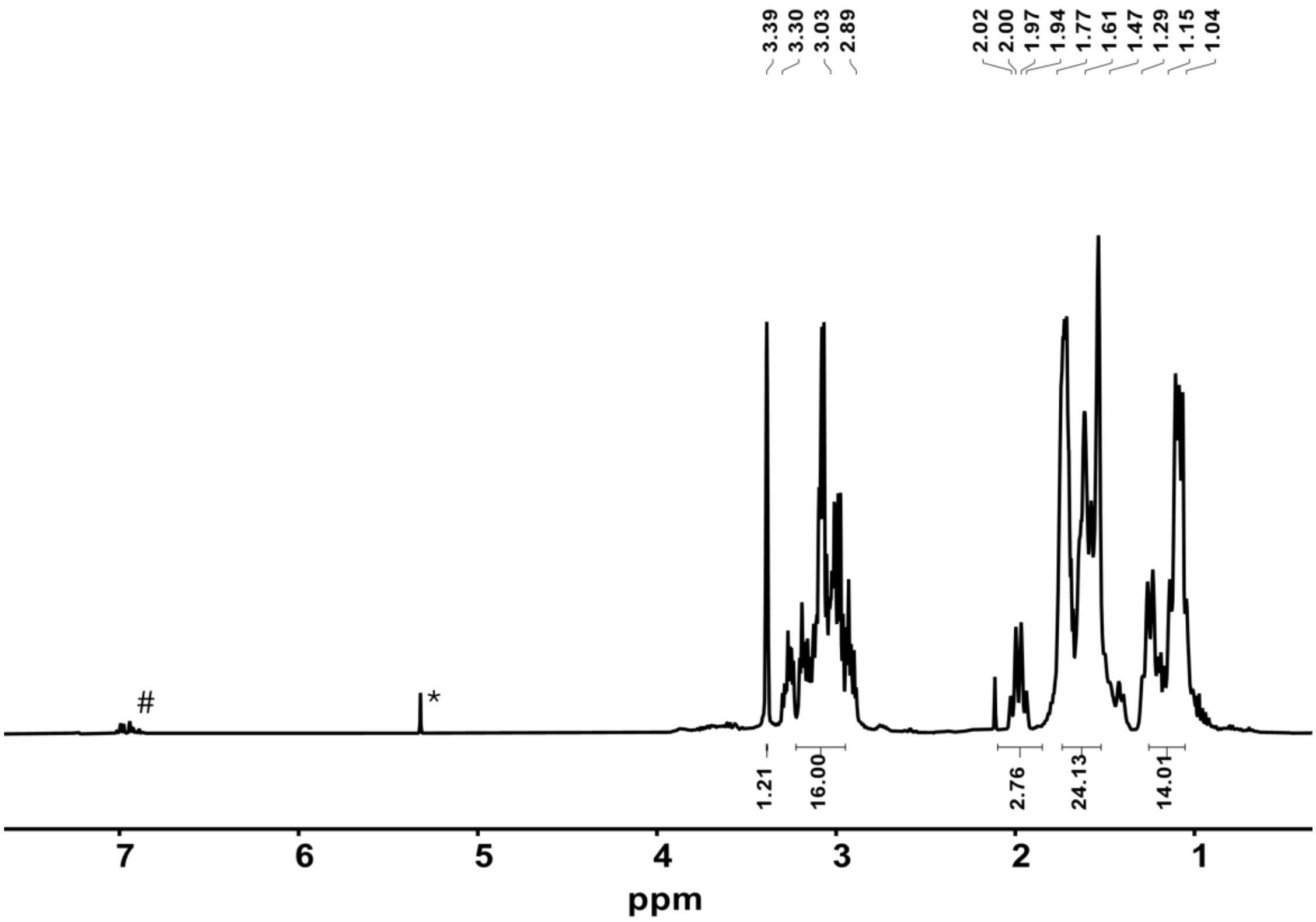


***Figure S2.*** *$^1$H{$^{11}$B} NMR spectrum (400 MHz) of [($PCy_3$)(H){B(µ-hpp)}$_2$][HB($C_6F_5$)$_3$] in $CD_2Cl_2$.The asterisk * denotes a solvent signal. # denotes residues of toluene.*

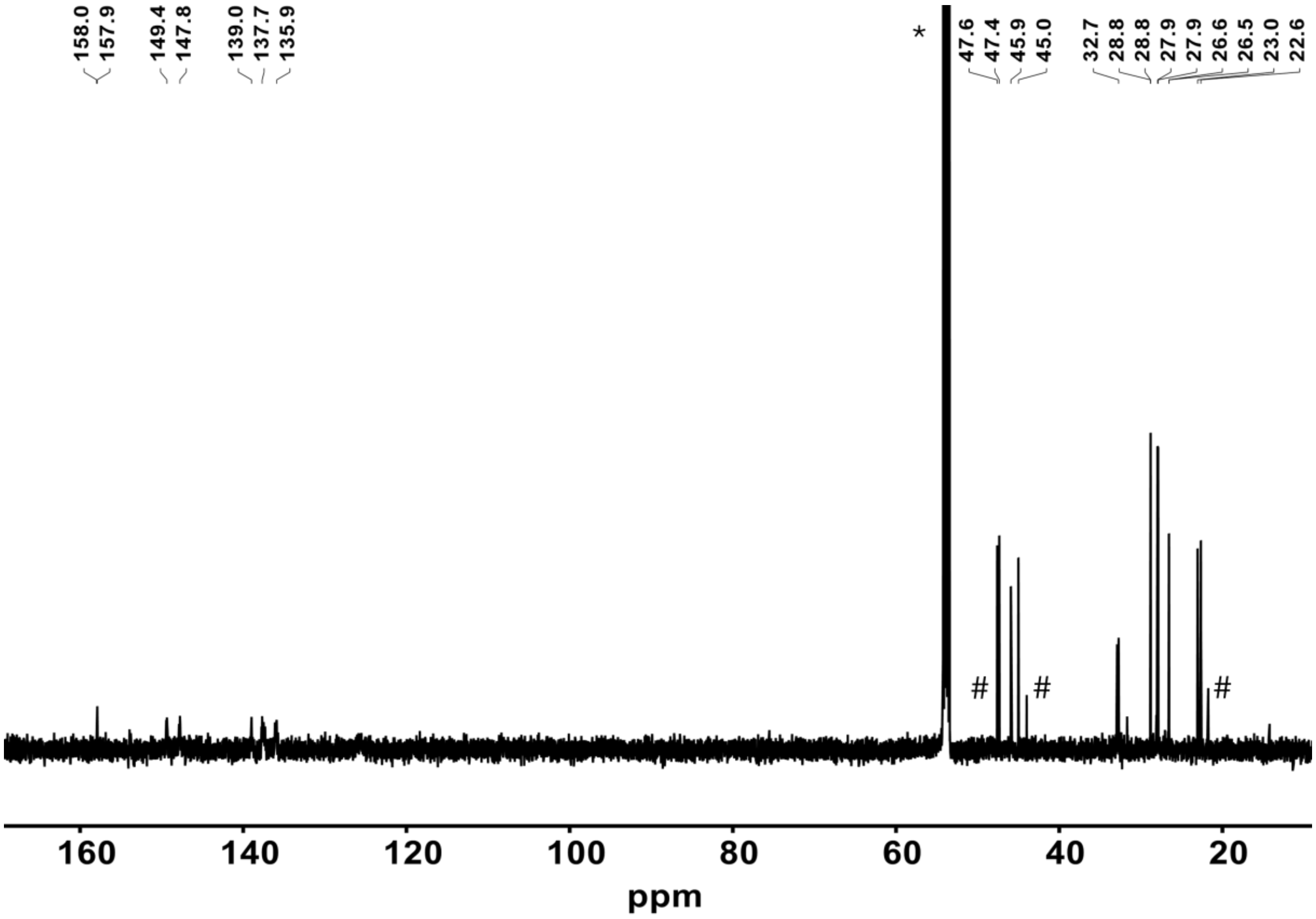


***Figure S3.*** *$^{13}C\{^1H\}$ NMR spectrum (100 MHz) of $[(PCy_3)(H)\{B(\mu\text{-hpp})\}_2][HB(C_6F_5)_3]$ in $CD_2Cl_2$. The asterisk * denotes a solvent signal. # denote free $hppH_2^+$ ligand.*

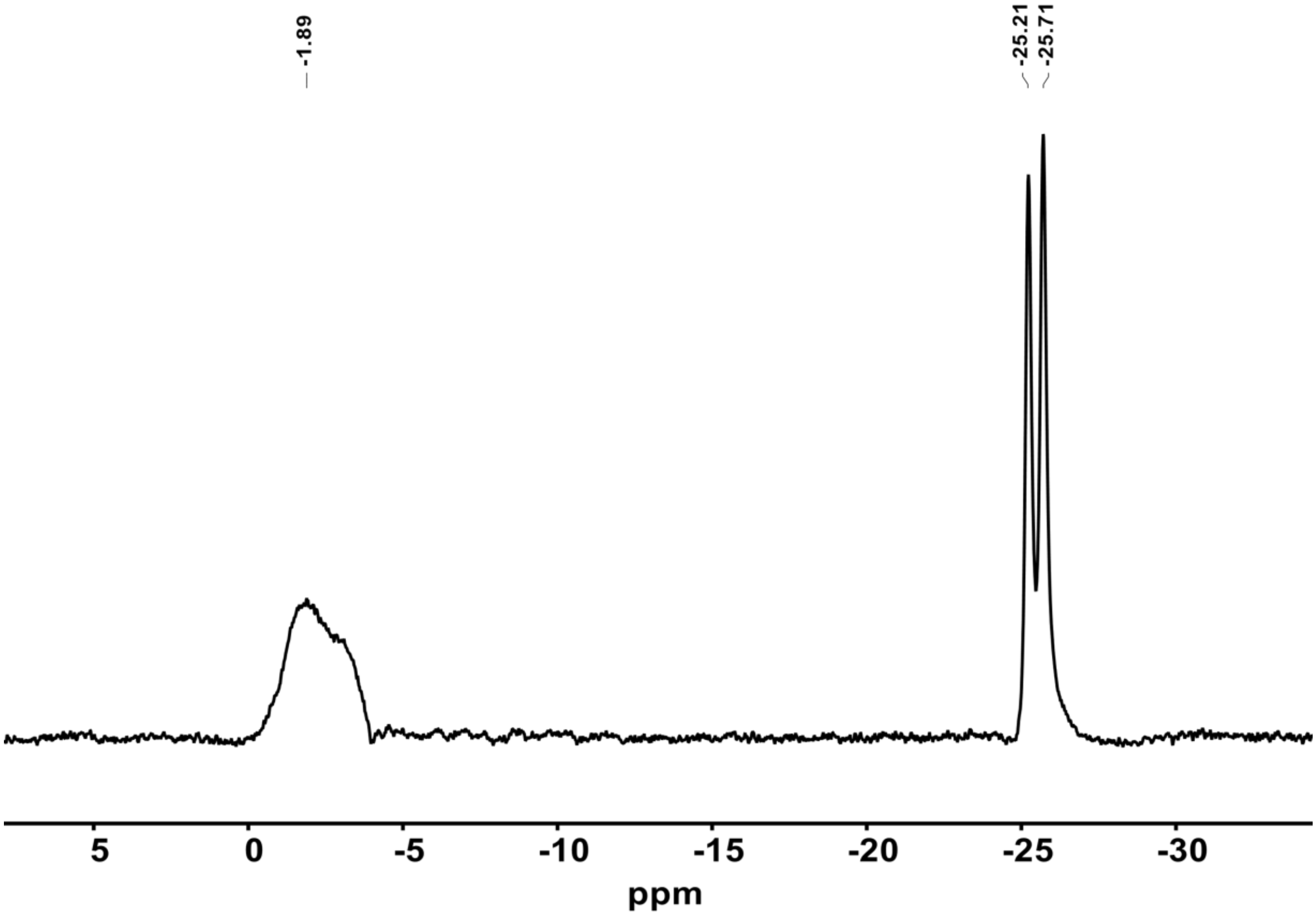


***Figure S4.*** *$^{11}B$ NMR spectrum (193 MHz) of $[(PCy_3)(H)\{B(\mu\text{-hpp})\}_2][HB(C_6F_5)_3]$ in $CD_2Cl_2$*

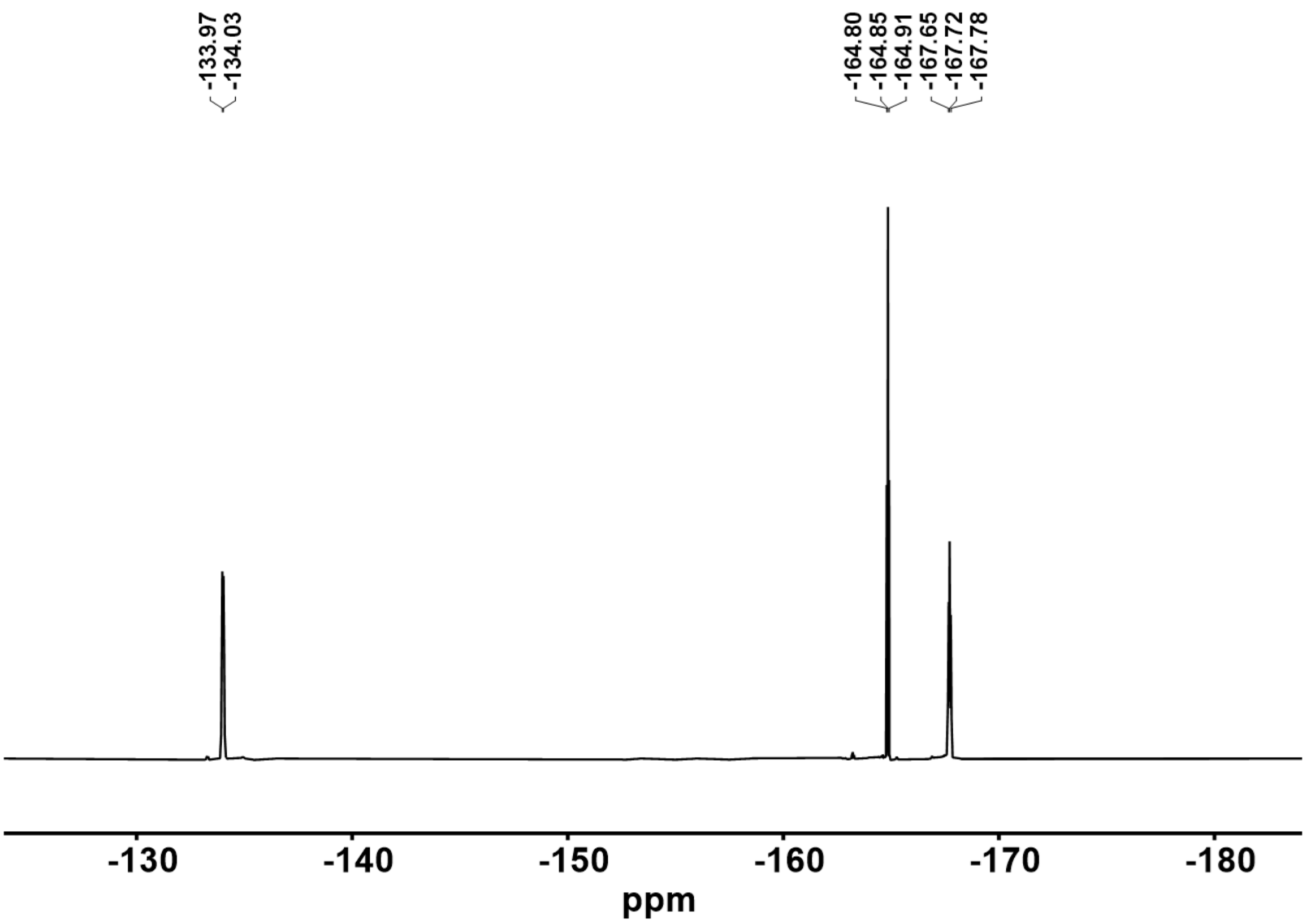


*Figure S5.* $^{19}F$ NMR spectrum (377 MHz) of $[(PCy_3)(H)\{B(\mu\text{-}hpp)\}_2][HB(C_6F_5)_3]$ in $CD_2Cl_2$.

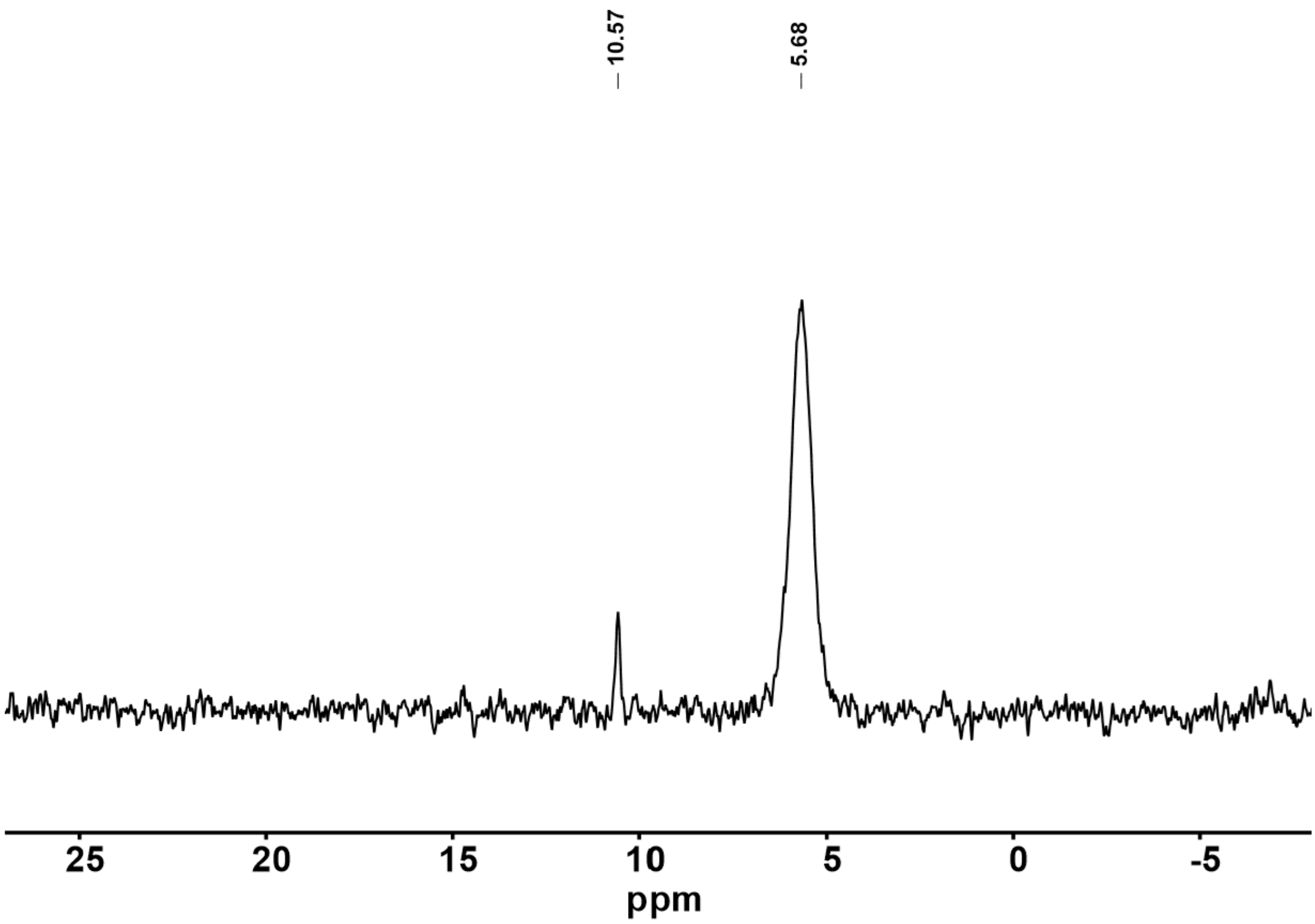


*Figure S6.* $^{31}P$ NMR spectrum (243 MHz) of $[(PCy_3)(H)\{B(\mu\text{-}hpp)\}_2][HB(C_6F_5)_3]$ in $CD_2Cl_2$.

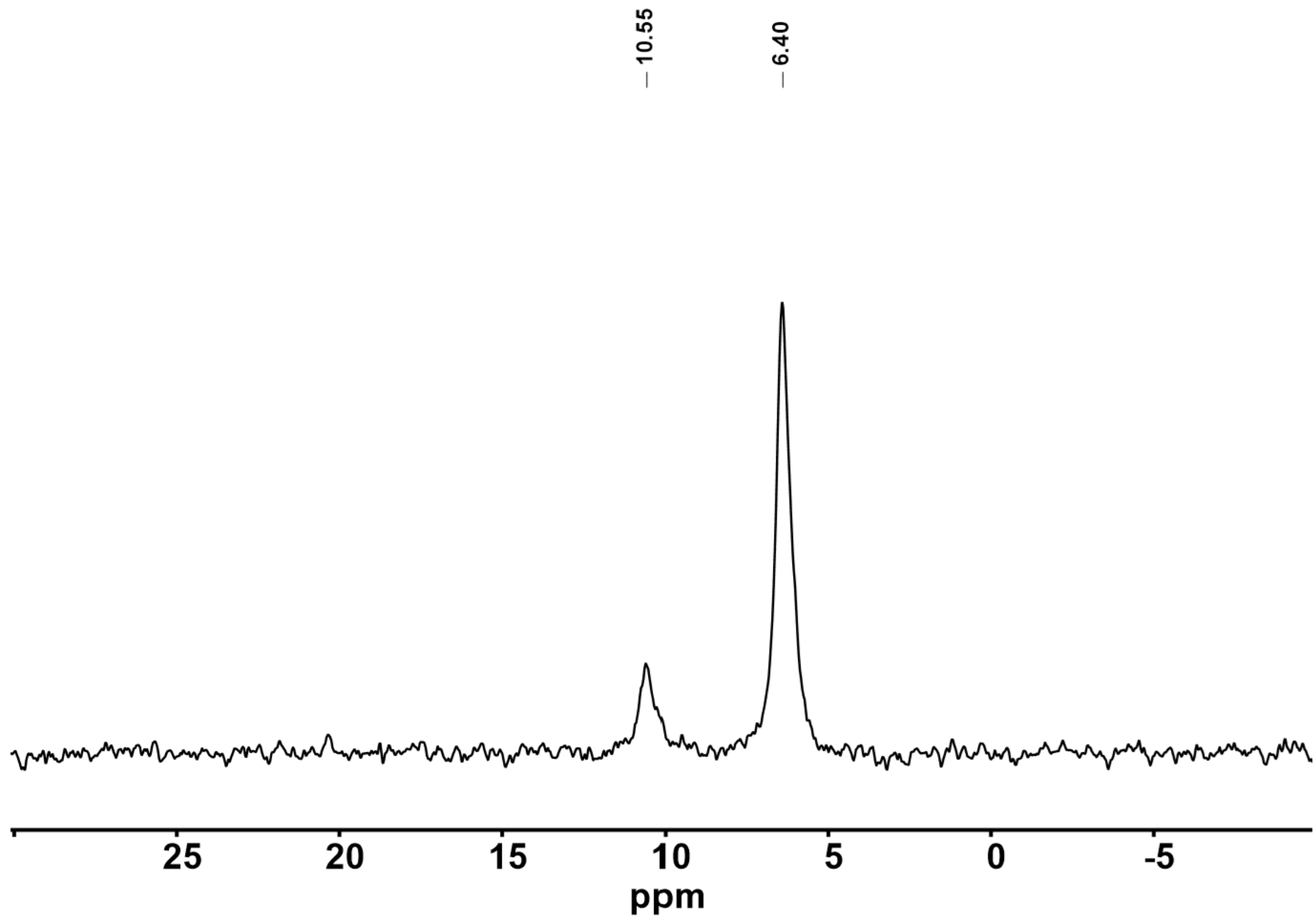


***Figure S7.*** *$^{31}$P NMR spectrum (243 MHz) of [(PCy$_3$)(H){B(μ-hpp)}$_2$][HB(C$_6$F$_5$)$_3$] in bromobenzene.*

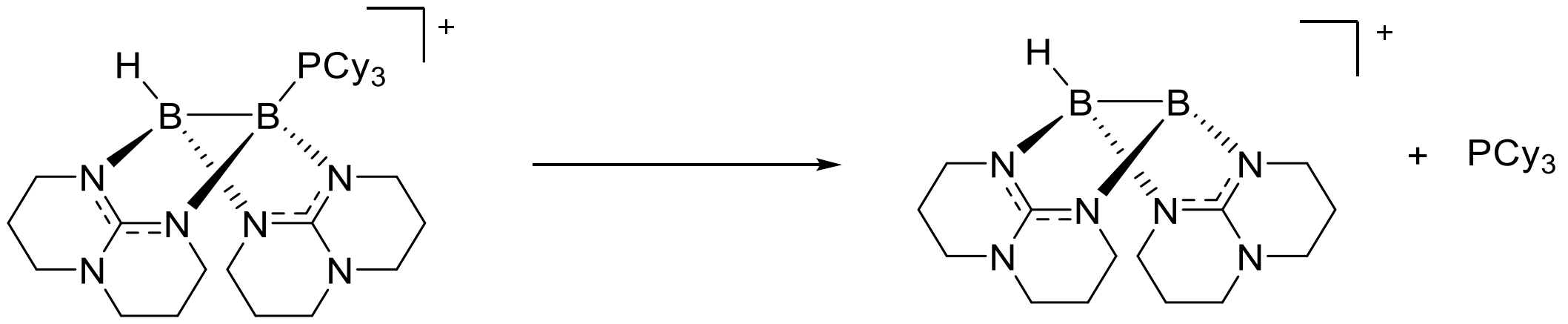


***Scheme S1.*** *Hypothetical dissociation reaction of [(PCy$_3$)(H){B(μ-hpp)}$_2$]$^+$* ***DB2$^+$*** *to PCy$_3$ and [HB(μ-hpp)$_2$B]$^+$.*

***Table S1.*** *Gibbs free energy, enthalpy and entropy change for the reaction shown in* ***Scheme S1*** *from B3LYP+D3/def2-TZVP calculations with and without solvent effects included using the COSMO continuum solvation model.*

| | Δ$G_R$ [kJ mol$^{-1}$] | Δ$H$ [kJ mol$^{-1}$] | Δ$S$ [J mol$^{-1}$ K$^{-1}$] |
|---|---|---|---|
| Gas phase | -12 | +48 | 203 |
| DCM (ε ≈ 8.9) | -46 | +15 | 203 |
| BrPh (ε ≈ 5.4) | -42 | +19 | 203 |

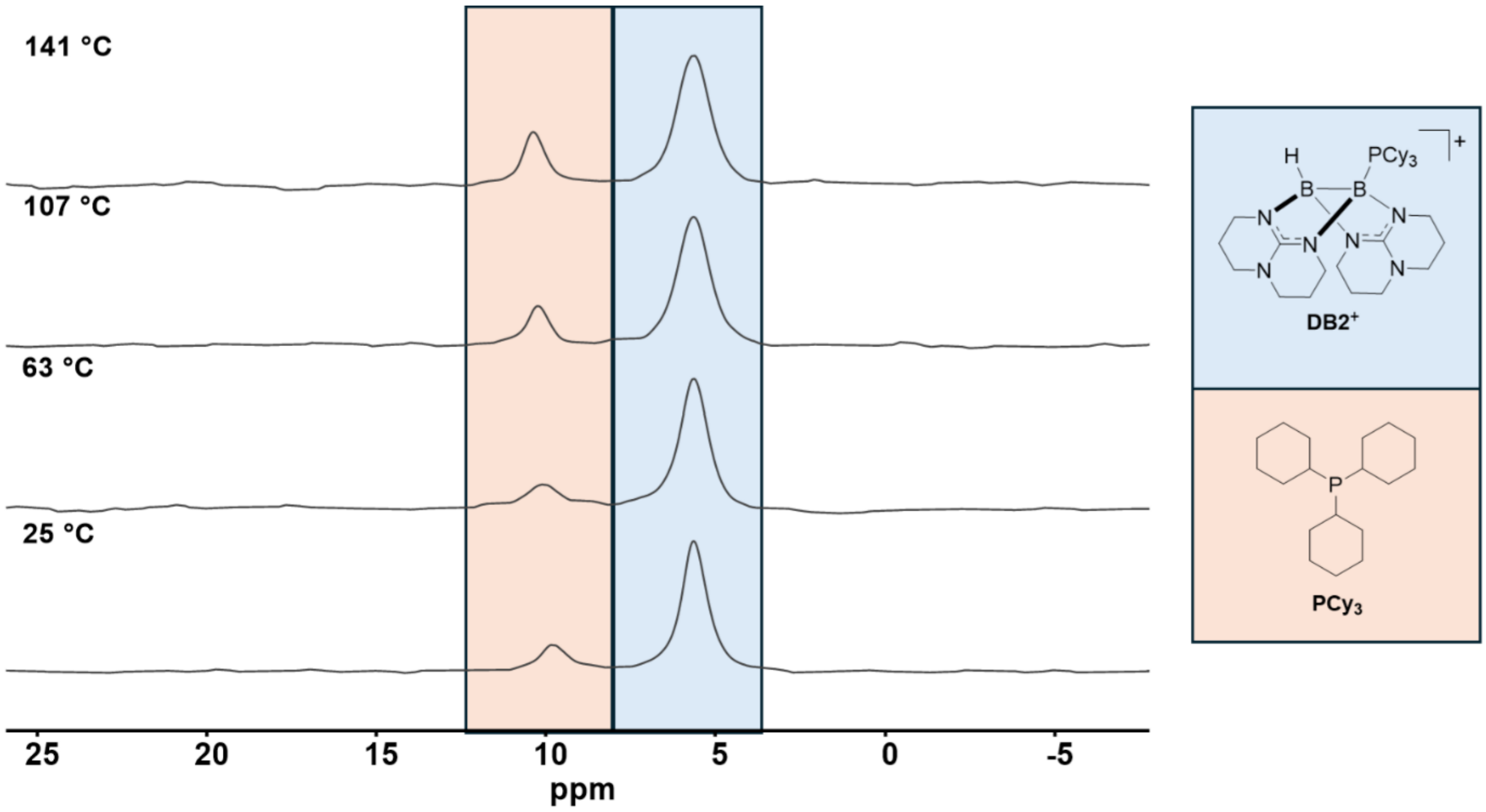


***Figure S8.*** *Variable temperature $^{31}P$ NMR spectra (243 MHz) of a c = 3.663·10$^{-3}$ mol L$^{-1}$ solution of [(PCy$_3$)(H){B(μ-hpp)}$_2$][HB(C$_6$F$_5$)$_3$] in bromobenzene at 25, 63, 107, and 141 °C.*

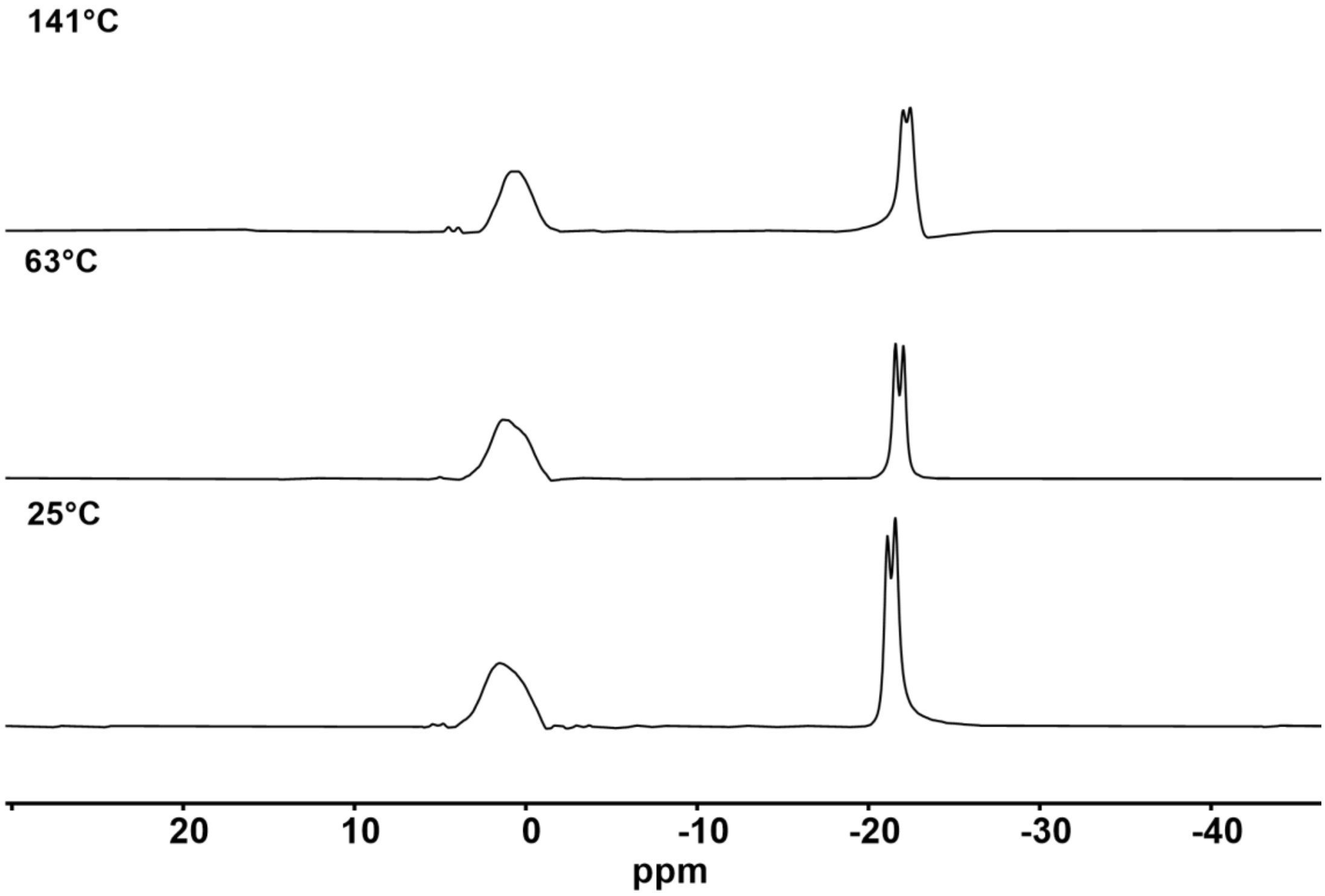


***Figure S9.*** *Variable temperature $^{11}B$ NMR spectra (193 MHz) of a c = 3.663×10$^{-3}$ mol L$^{-1}$ solution of [(PCy$_3$)(H){B(μ-hpp)}$_2$][HB(C$_6$F$_5$)$_3$] in bromobenzene at 25, 63 and 141 °C.*

***Equation S1:*** *Calculation of the equilibrium constant **K** for the dissociation of **DB2⁺***

$$\boldsymbol{K} = \frac{[PCy_3] * [(H)\{B(\mu - hpp)\}_2{}^{+}]}{[(PCy_3)(H)\{B(\mu - hpp)\}_2{}^{+}]} * \frac{1}{c^{\circ}}$$

$$[PCy_3] = [(H)\{B(\mu - hpp)\}_2{}^+]$$

$$\boldsymbol{K} = \frac{[PCy_3]^2}{[(PCy_3)(H)\{B(\mu - hpp)\}_2{}^+]} * \frac{1}{c^{\circ}}$$

$$[PCy_3] = c_0 * \alpha$$

$$\left[(PCy_3)(H)\{B(\mu - hpp)\}_2{}^+\right] = c_0 * (1 - \alpha)$$

$$\Delta G = -RTln(K)$$

***Table S2.*** *Degree of dissociation for **DB2**$^+$ obtained from the spectra in **Figure S7**.*

| *T* [K] | $PCy_{3,\ free}$ to $PCy_{3,\ bound}$ | Degree of dissociation *α* | *c*[$PCy_3$] [mol $L^{-1}$] | *c*[($PCy_3$)(H){B(*μ*-hpp)}$_2$] |
|---|---|---|---|---|
| 298.15 | 0.18:1 | 18/118 | 0.00558763 | 0.0310424 |
| 336.15 | 0.20:1 | 20/120 | 0.006105 | 0.030525 |
| 380.15 | 0.23:1 | 23/123 | 0.00684951 | 0.0297805 |
| 414.15 | 0.30:1 | 30/130 | 0.00845308 | 0.0281769 |

***Table S3.*** *Values of K and ΔG calculated from the data in **Table S2.***

| *T* [K] | K | Δ*G* |
|---|---|---|
| 298.15 | 0.0010058 | 17.1088069 |
| 336.15 | 0.001221 | 18.7474282 |
| 380.15 | 0.0015754 | 20.3959417 |
| 414.15 | 0.0025359 | 20.5809443 |

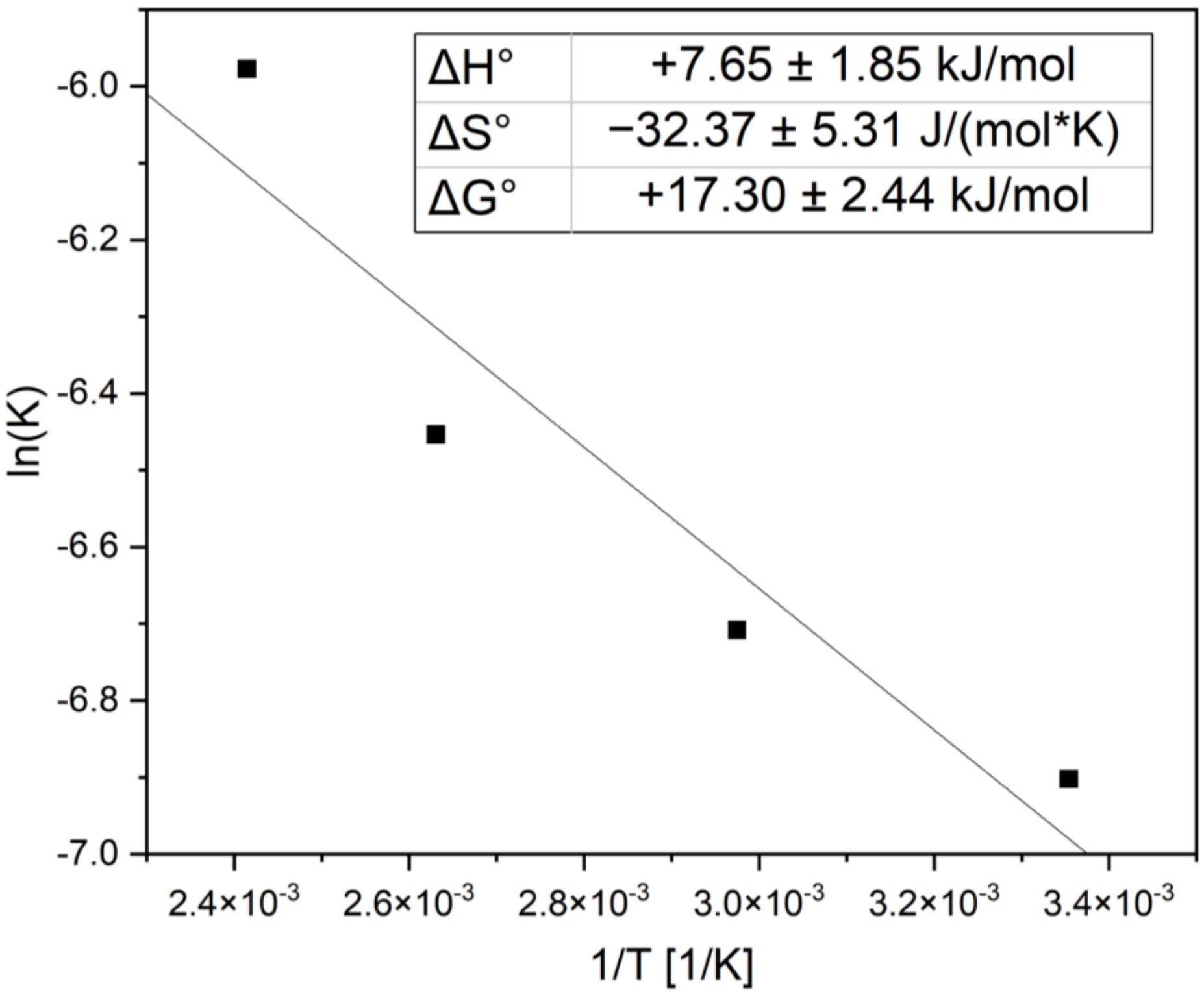


***Figure S10.*** *Arrhenius graph for the dissociation of **DB2**$^+$ plotted with data from **Table S3.***

## 2.1.2 [(dcpe){B($\mu$-hpp)}$_2$][OTf]$_2$ **DB4**[OTf]$_2$

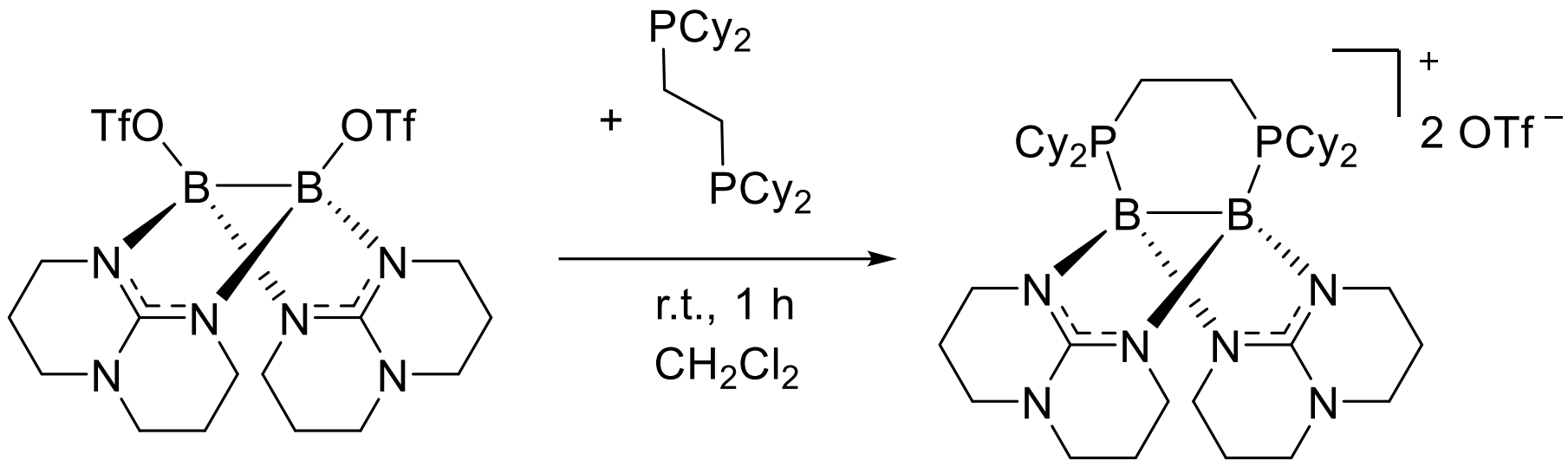

In a Schlenk flask, 61.4 mg of **DB3** (103 µmol, 1.00 eq.) and 43.5 mg of 1,2-bis(dicyclohexylphosphino)ethane (103 µmol, 1.00 eq.) were dissolved in 2 mL dichloromethane. After one hour of stirring at room temperature the solvent was removed under vacuum. The product was washed with toluene (3 x 3 mL and *n*-pentane (3 x 3 mL) and dried under vacuum. **DB4**[OTf]$_2$ was obtained in a yield of 72.4 mg (71.1 mol, 69%) as a colorless solid.

**$^{1}$H NMR (400 MHz, CD$_2$Cl$_2$):**

$\delta$ = 3.58–3.14 (m, 16H, $H_{hpp}$), 2.25–2.15 (m, 4H, $H_{Cy\text{-}CH}$), 2.07–1.79 (m, 32H, $H_{Cy\text{-}CH2}$,$H_{hpp}$), 1.57–1.26 (m, 20H, $H_{Et}$,$H_{Cy\text{-}CH2}$) ppm.

**$^{13}$C{$^{1}$H} NMR (151 MHz, CD$_2$Cl$_2$):**

$\delta$ = 160.7 ($C_{q\text{-}hpp}$), 121.4 (q, $^{1}J_{CF}$ = 321 Hz, $C_{OTf}$), 47.6 ($C_{hpp}$), 45.3 ($C_{hpp}$), 32.4 (d, $^{1}J_{PC}$ = 25 Hz, $C_{Cy\text{-}CH}$), 28.9 (d, $^{3}J_{PC}$ = 4 Hz, $C_{Cy\text{-}CH2}$), 28.4 (d, $^{3}J_{PC}$ = 4 Hz, $C_{Cy\text{-}CH2}$), 27.9 (d, $^{2}J_{PC}$ = 10 Hz, $C_{Cy\text{-}CH2}$), 27.3 (d, $^{2}J_{PC}$ = 10 Hz, $C_{Cy\text{-}CH2}$), 26.1 ($C_{Et}$), 22.1 ($C_{hpp}$) ppm.

**$^{11}$B NMR (128 MHz, CD$_2$Cl$_2$):**

$\delta$ = –4.85 (bs, 2B) ppm.

**$^{11}$B{$^{1}$H} NMR (128 MHz, CD$_2$Cl$_2$):**

$\delta$ = –4.02 (bs, 2B) ppm.

**$^{19}$F NMR (377 MHz, CD$_2$Cl$_2$):**

$\delta$ = –78.81 (s, 2F, $OTf^{-}_{free}$) ppm.

**$^{31}$P-NMR (162 MHz, CD$_2$Cl$_2$):**

$\delta$ = –4.69 (bs, 2P, $P_{PCy2}$) ppm.

**Mass spectrum (HR-TOF-MALDI$^{+}$):**

m/z = 869.5007 ([M+OTf$^{-}$]$^{+}$, 100%).

**IR** (ATR): 2928 (m), 2856 (m), 1601 (w), 1587 (m), 1542 (m), 1445 (m), 1395 (w), 1372 (w), 1322 (m), 1259 (s), 1220 (m), 1144 (m), 1097 (w), 1047 (w), 1028 (s), 1001 (w), 944 (w), 902 (w), 841 (w), 813 (w), 735 (w), 709 (w), 655 (w), 634 (w), 571 (w), 532 (w), 515 (w) cm$^{-1}$.

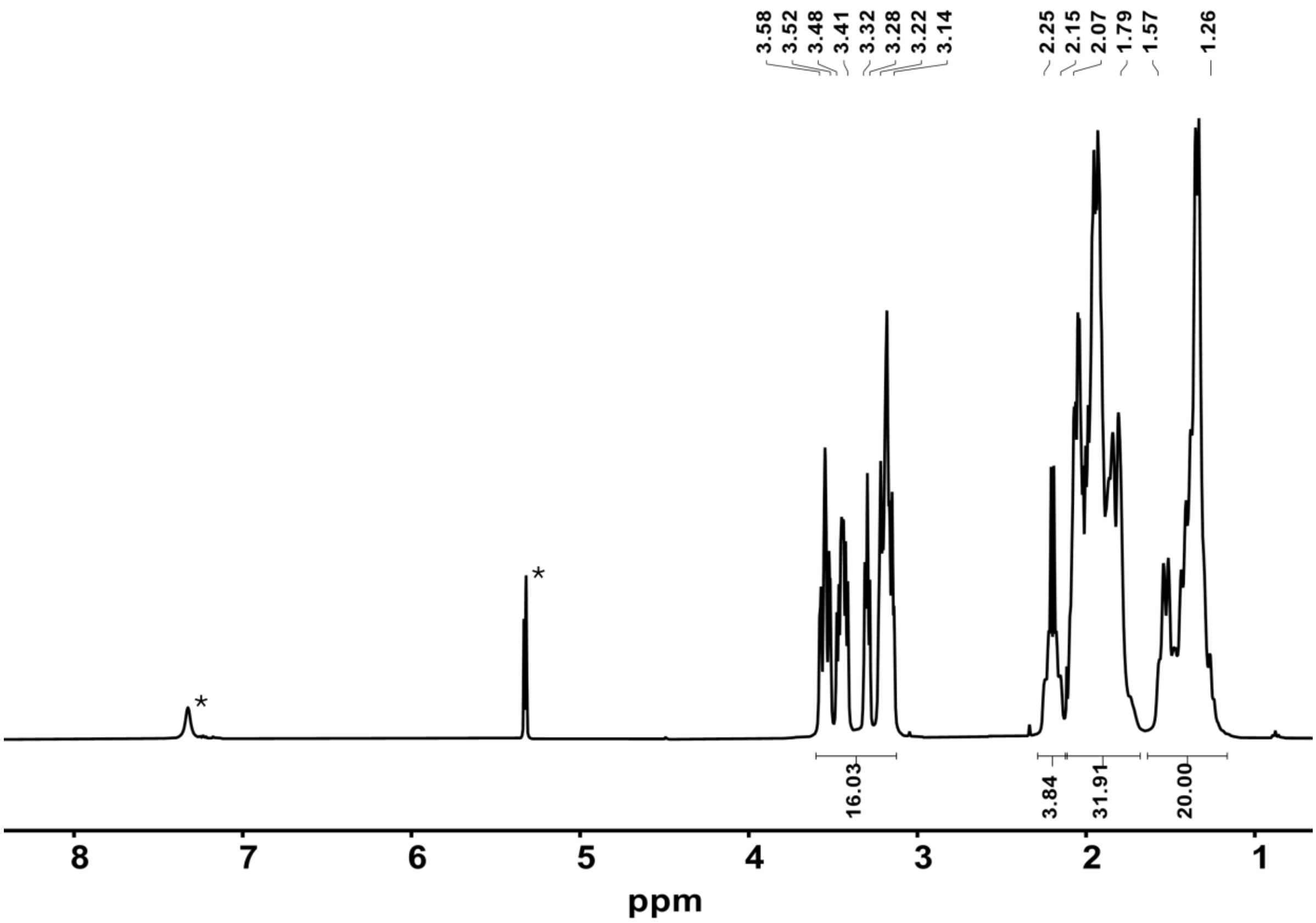


***Figure S11.*** *$^1$H NMR spectrum (400 MHz) of [(dcpe){B(μ-hpp)}$_2$][OTf]$_2$ in $CD_2Cl_2$. The asterisk * denotes a solvent signal.*

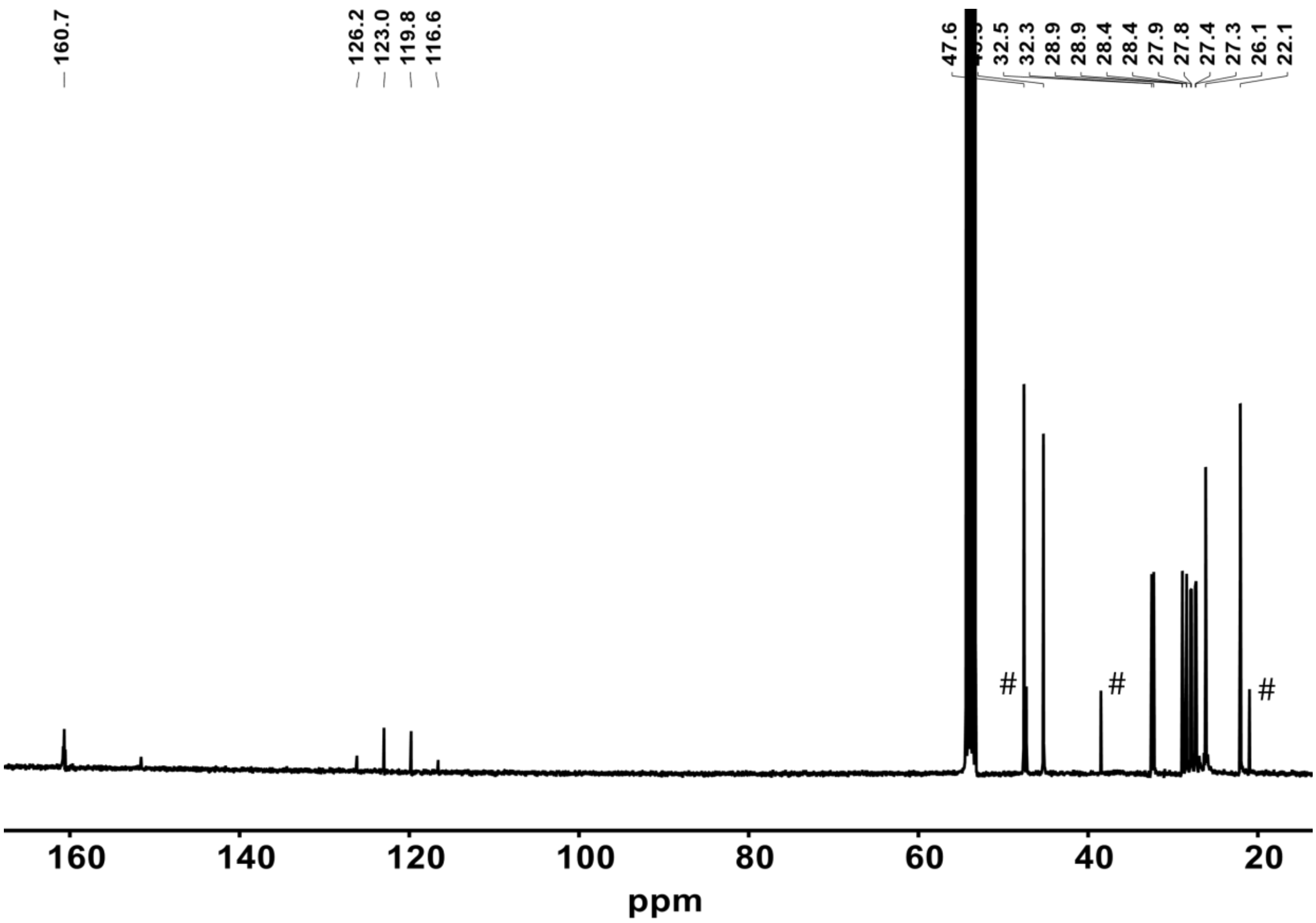


***Figure S12.*** *$^{13}C\{^1H\}$ NMR spectrum (100 MHz) of [(dcpe){B(μ-hpp)}$_2$][OTf]$_2$ in $CD_2Cl_2$. # denote free $hppH_2^+$ ligand.*

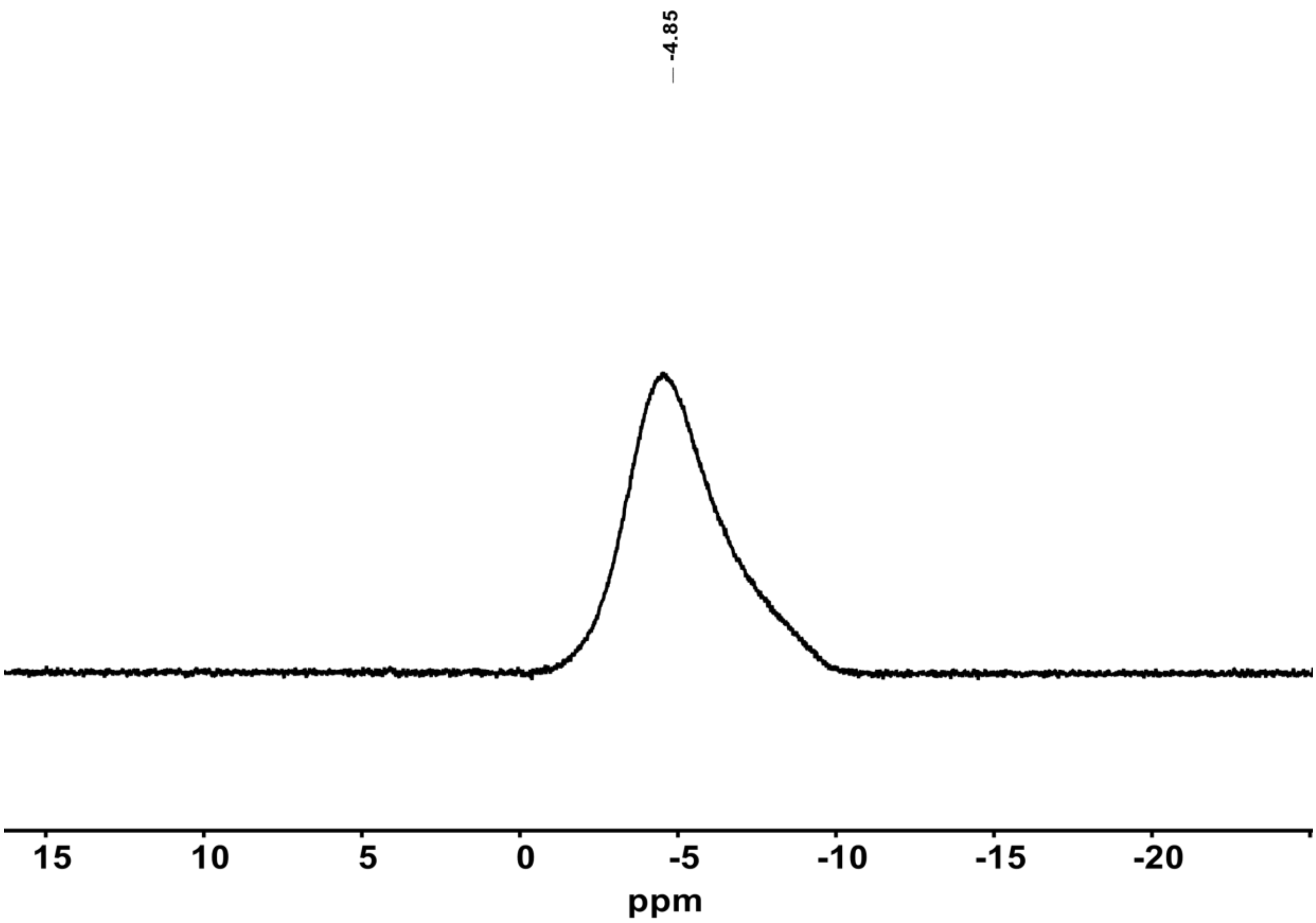


***Figure S13.*** *$^{11}$B NMR spectrum (128 MHz) of [(dcpe){B(μ-hpp)}$_2$][OTf]$_2$ in $CD_2Cl_2$.*

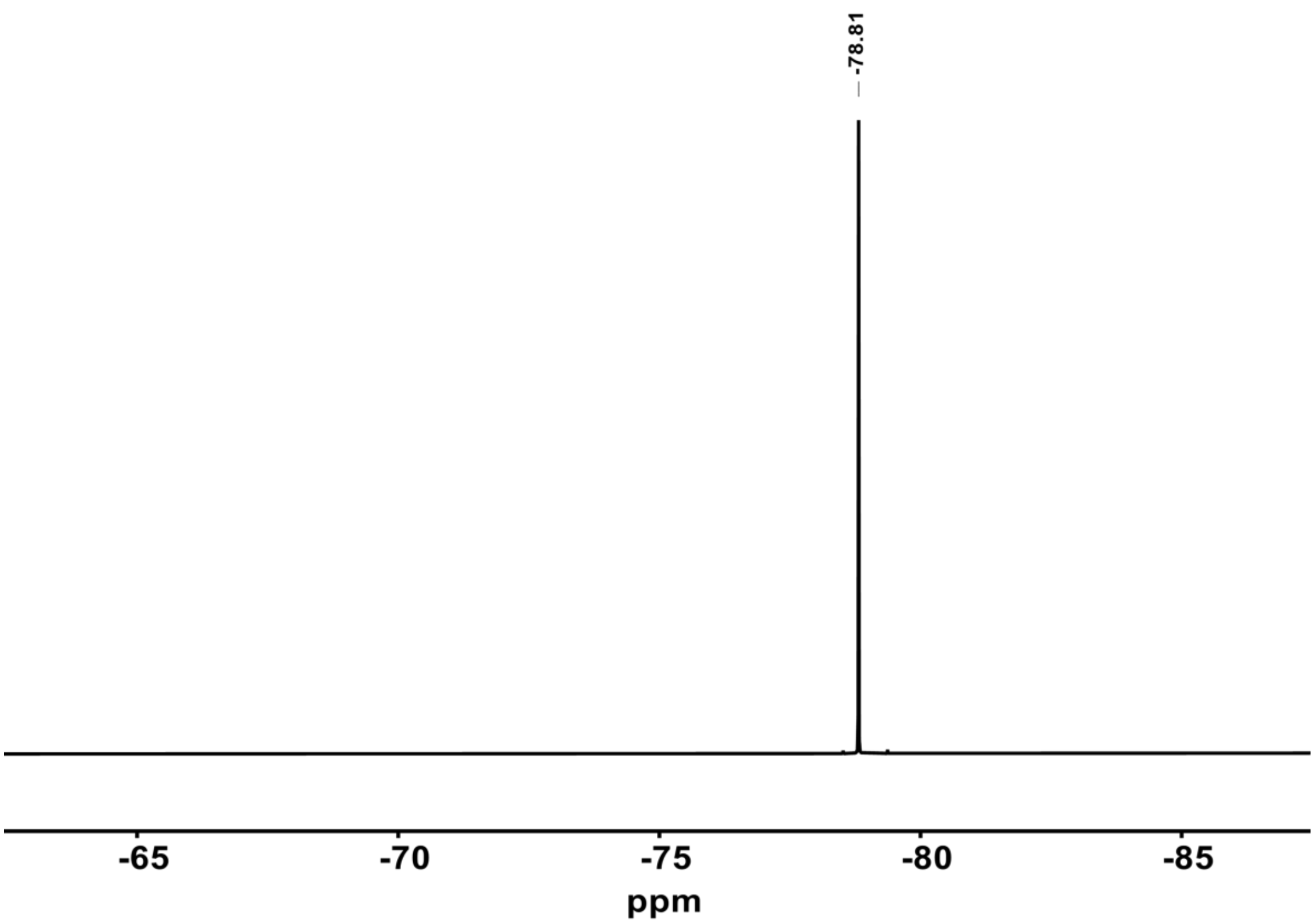


***Figure S14.*** *$^{19}$F NMR spectrum (377 MHz) of [(dcpe){B(μ-hpp)}$_2$][OTf]$_2$ in $CD_2Cl_2$.*

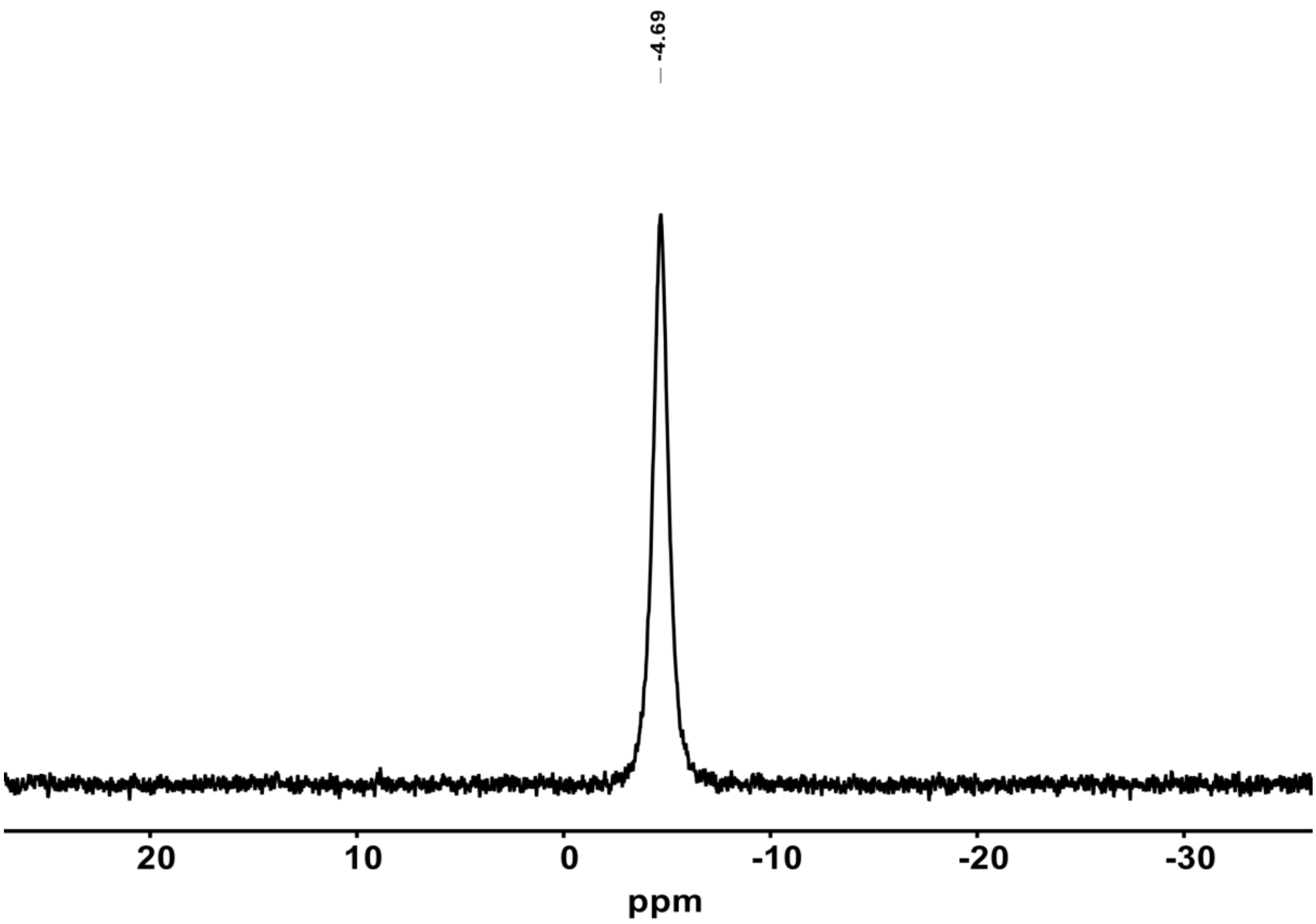


***Figure S15.*** *$^{31}P$ NMR spectrum (162 MHz) of [(dcpe){B(μ-hpp)}$_2$][OTf]$_2$ in $CD_2Cl_2$.*

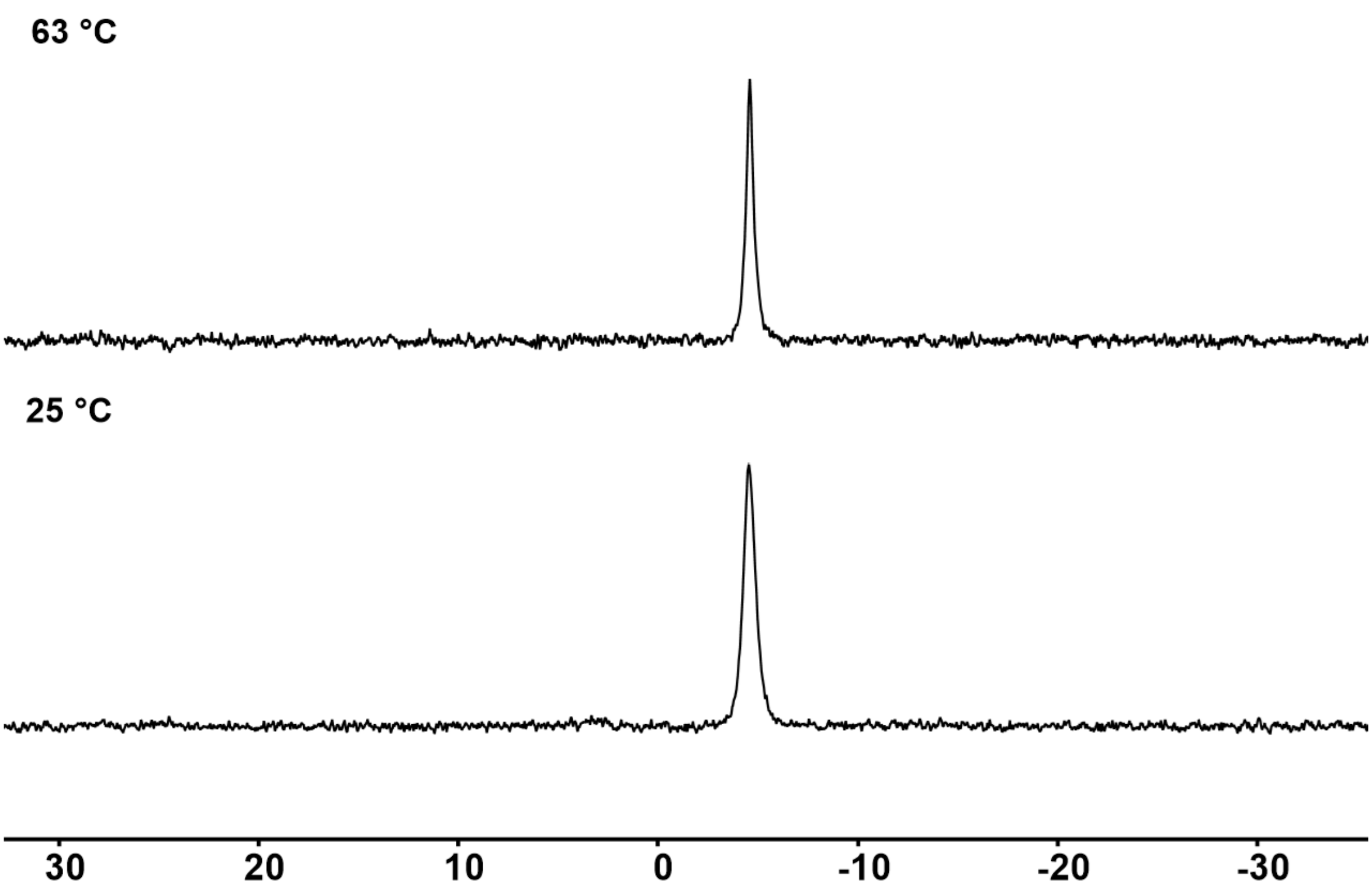


***Figure S16.*** *Variable temperature $^{31}P$ NMR spectrum (162 MHz) of [(dcpe){B(μ-hpp)}$_2$][OTf]$_2$ in THF-$d_8$ at 25 °C and 63 °C.*

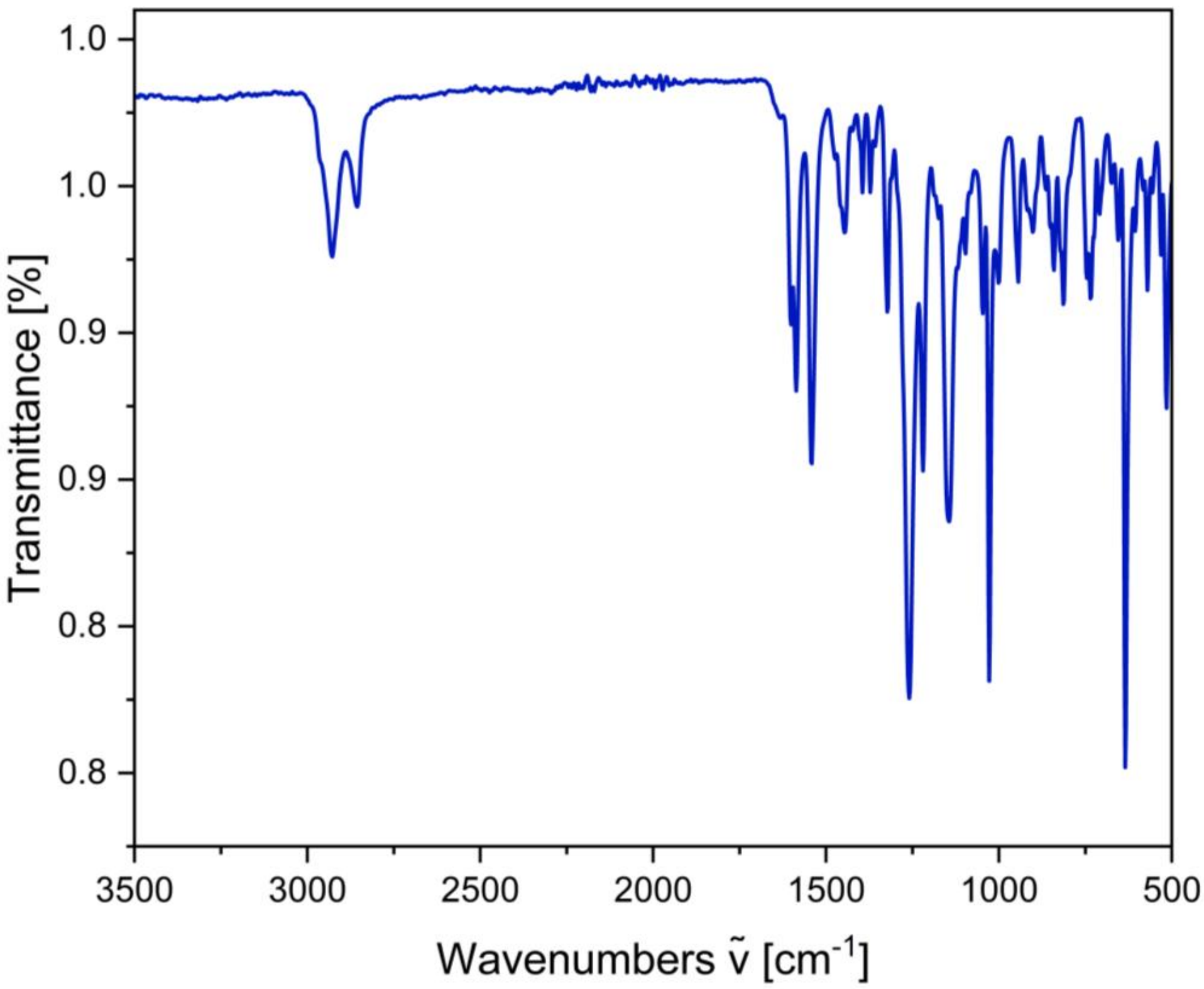


***Figure S17.*** *IR spectrum of [(dcpe){B(μ-hpp)}$_2$][OTf]$_2$ as powder.*

# 2.2 Triboriranes

## 2.2.1 [{HB($\mu$-hpp)}$_2$($\mu$-BH$_2$)][B(cat)$_2$] **TB2**[B(cat)$_2$]

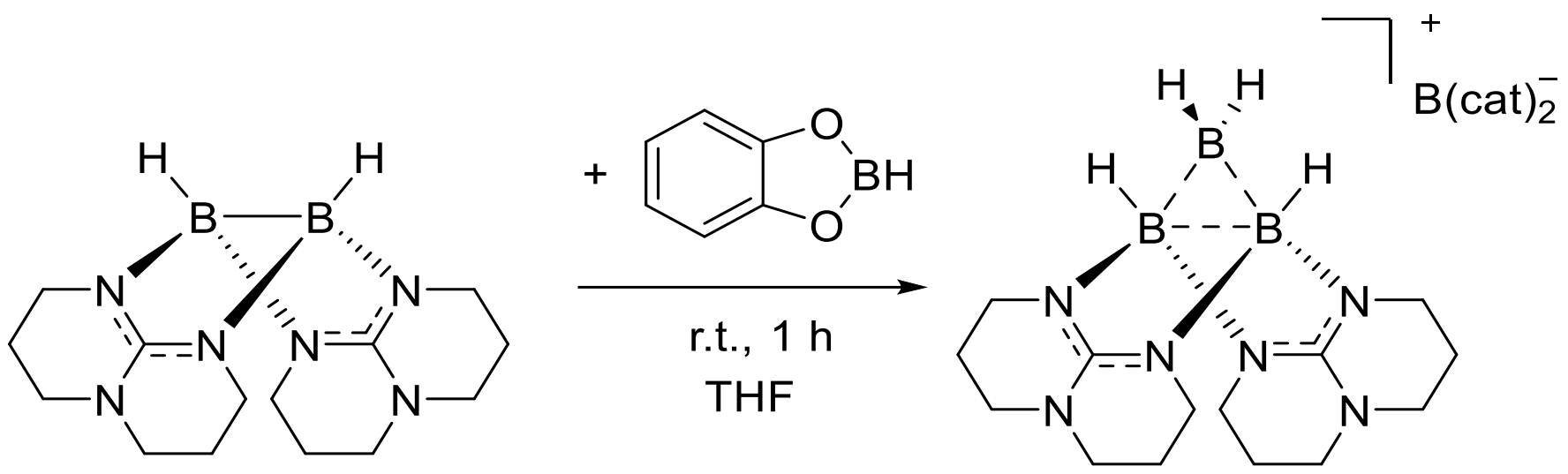


In a Schlenk flask, 160 mg of **DB1** (533 µmol, 1.00 eq.) was suspended in 2.5 mL tetrahydrofuran. In a second Schlenk flask, 141 mg of catecholborane (1.17 mmol, 2.20 eq.) was dissolved in 1.5 mL tetrahydrofuran. The catecholborane solution was added dropwise to the suspension immediately forming a colorless precipitate. After one hour of stirring the tetrahydrofuran supernatant was removed *via* syringe and the colorless residue was washed with tetrahydrofuran (3 x 2 mL) and dried under vacuum. **TB2**[B(cat)$_2$] was obtained in a yield of 186 mg (347 µmol, 65%) as a colorless powder.

**$^1$H NMR (600 MHz, CD$_2$Cl$_2$):**

$\delta$ = 6.58–6.52 (m, 8H, $H_{\mathrm{Cat}}$), 3.36–3.07 (m, 16H, $H_{\mathrm{hpp}}$), 1.97–1.88 (m, 8H, $H_{\mathrm{hpp}}$) ppm.

**$^1$H{$^{11}$B} NMR (600 MHz, CD$_2$Cl$_2$):**

$\delta$ = 6.58–6.52 (m, 8H, $H_{\mathrm{Cat}}$), 3.34–3.08 (m, 16H, $H_{\mathrm{hpp}}$), 2.29–2.28 (m, 2H, B$H$), 1.97–1.87 (m, 8H, $H_{\mathrm{hpp}}$) ppm.

**$^{13}$C{$^1$H} NMR (151 MHz, CD$_2$Cl$_2$):**

$\delta$ = 154.3 ($C_{\mathrm{q\text{-}hpp}}$), 152.5 ($C_{\mathrm{cat\text{-}O}}$), 117.8 ($C_{\mathrm{cat\text{-}H}}$), 108.4 ($C_{\mathrm{cat\text{-}H}}$), 46.4 ($C_{\mathrm{hpp}}$), 44.0 ($C_{\mathrm{hpp}}$), 21.8 ($C_{\mathrm{hpp}}$) ppm.

**$^{11}$B NMR (193 MHz, CD$_2$Cl$_2$):**

$\delta$ = 14.25 (s, 1B, $B_{\mathrm{cat}}$), –0.39 (d, $^1J_{\mathrm{BH}}$ = 78 Hz, 2B, $B$H) ppm.

**$^{11}$B{$^1$H} NMR (193 MHz, CD$_2$Cl$_2$):**

$\delta$ = 14.25 (s, 1B, $B_{\mathrm{cat}}$), –0.25 (s, 2B, $B$H) ppm.

**IR** (ATR): 2938 (w), 2867 (w), 2560 (w), 2486 (w), 2429 (vw), 1602 (m), 1572 (w), 1528 (w), 1482 (s), 1443 (w), 1401 (w), 1373 (w), 1320 (w), 1297 (w), 1277 (w), 1229 (s), 1142 (w), 1093 (w), 1047 (s), 1005 (w), 988 (w), 973 (w), 937 (w), 911 (w), 796 (w), 733 (m), 722 (w), 701 (m), 581 (w), 552 (w), 520 (w) cm$^{-1}$.

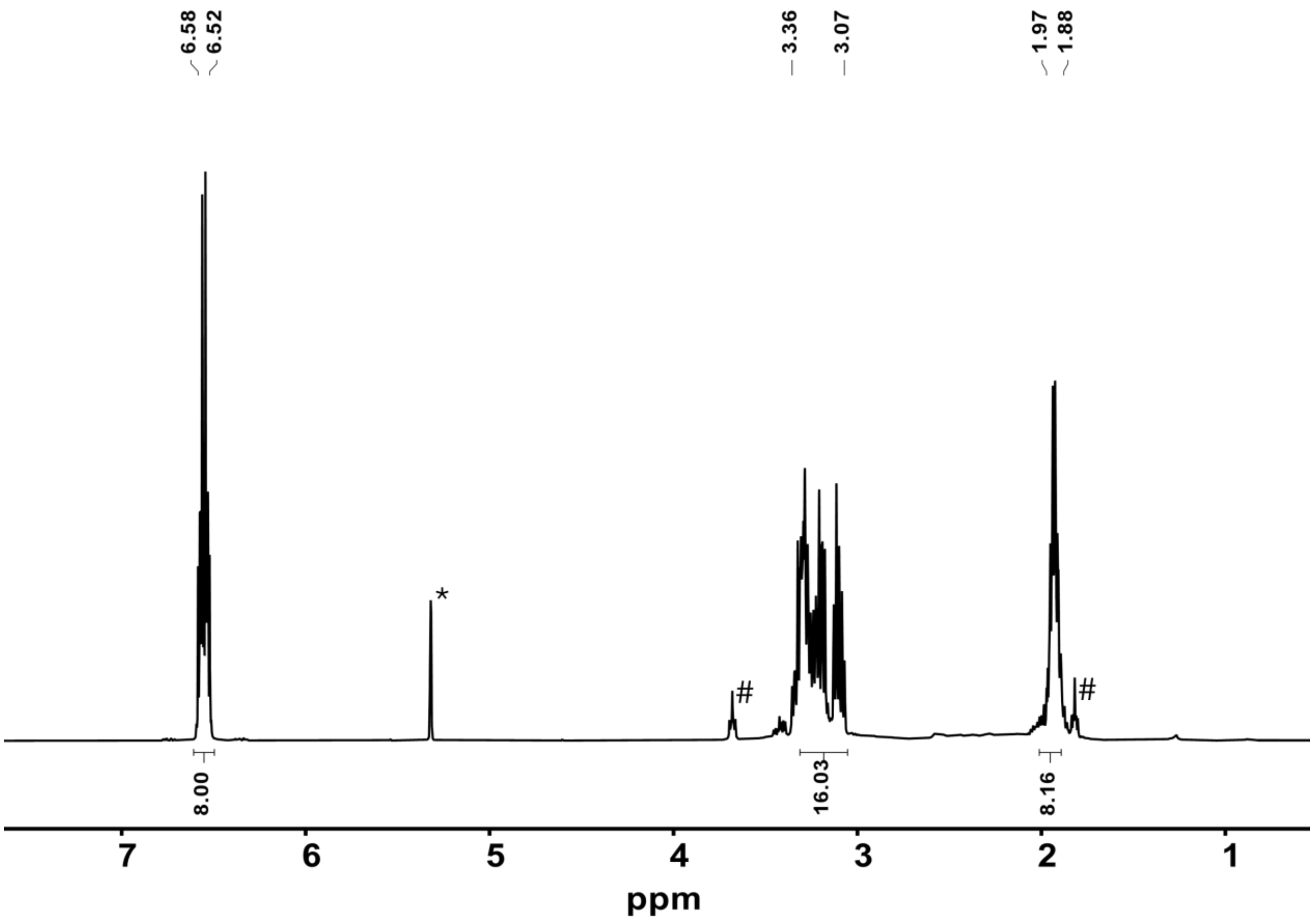


***Figure S18.*** *$^{1}H$ NMR spectrum (400 MHz) of [{HB(μ-hpp)}$_2$(μ-BH$_2$)][B(cat)$_2$] in $CD_2Cl_2$. The asterisk * denotes a solvent signal. # denotes residues of THF.*

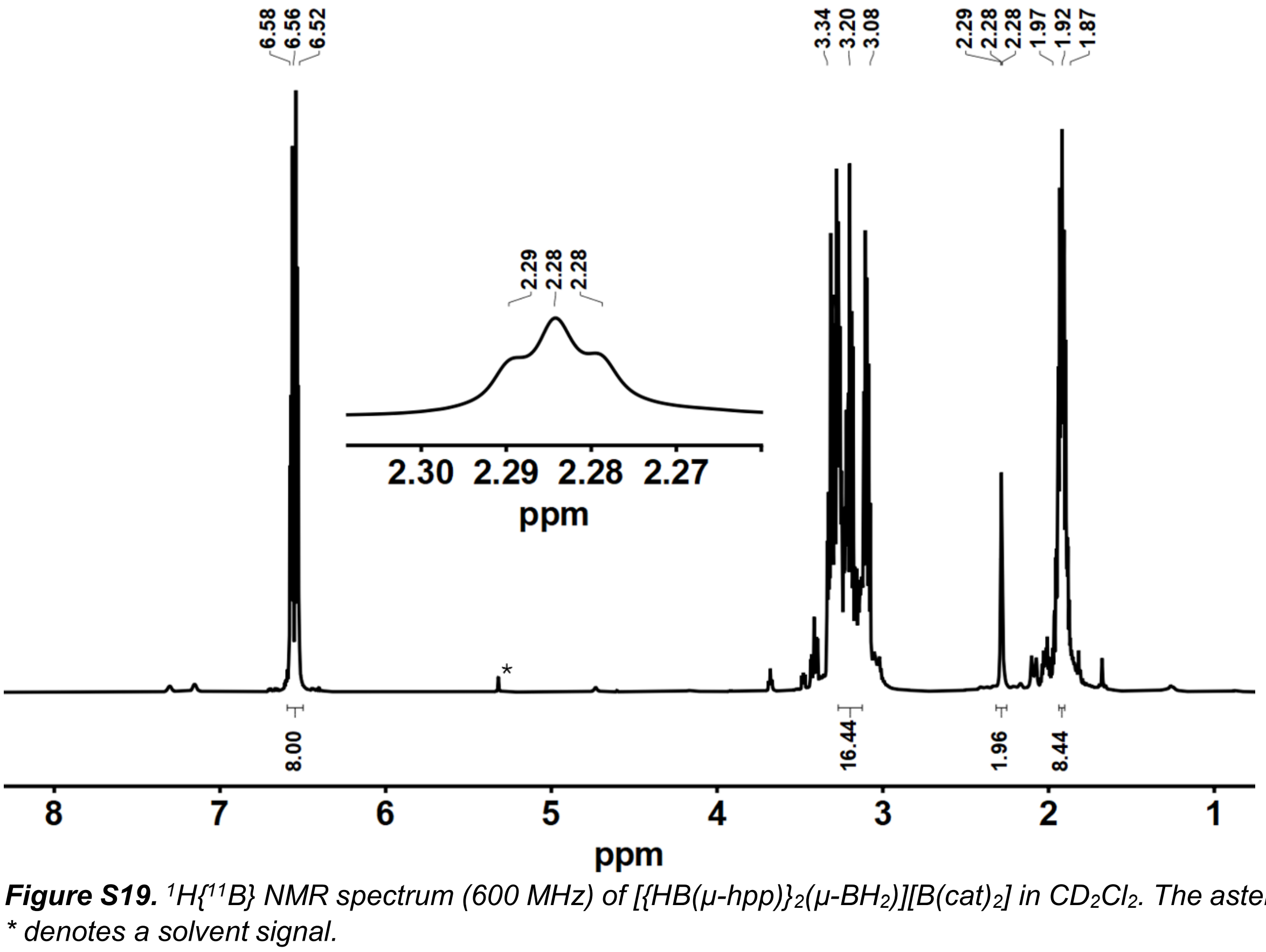


***Figure S19.*** *$^{1}H\{^{11}B\}$ NMR spectrum (600 MHz) of [{HB(μ-hpp)}$_2$(μ-BH$_2$)][B(cat)$_2$] in $CD_2Cl_2$. The asterisk * denotes a solvent signal.*

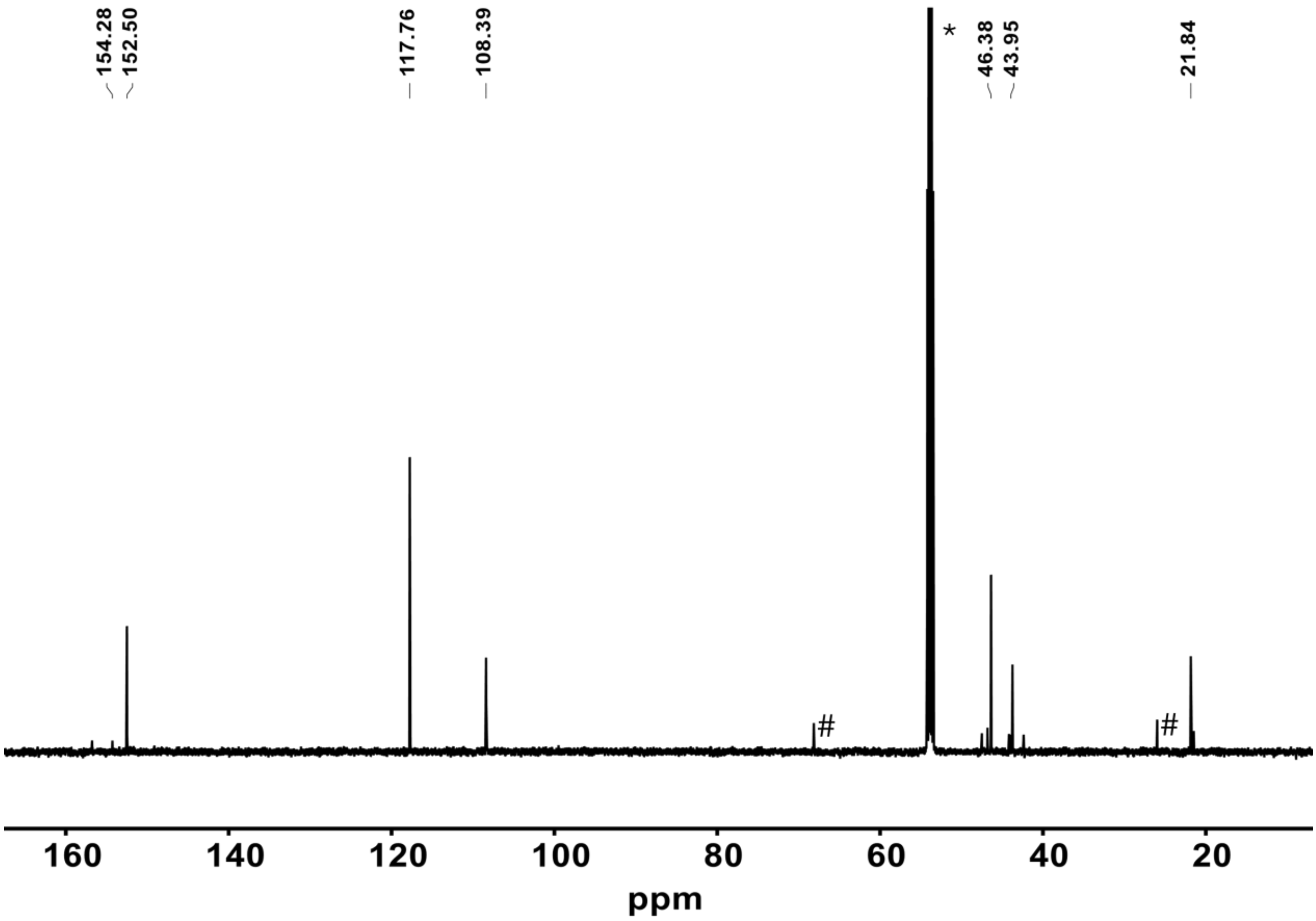


***Figure S20.*** *$^{13}C\{^1H\}$ NMR spectrum (100 MHz) of [{HB(μ-hpp)}$_2$(μ-$BH_2$)][B(cat)$_2$] in $CD_2Cl_2$. The asterisk * denotes a solvent signal. # denotes residues of THF.*

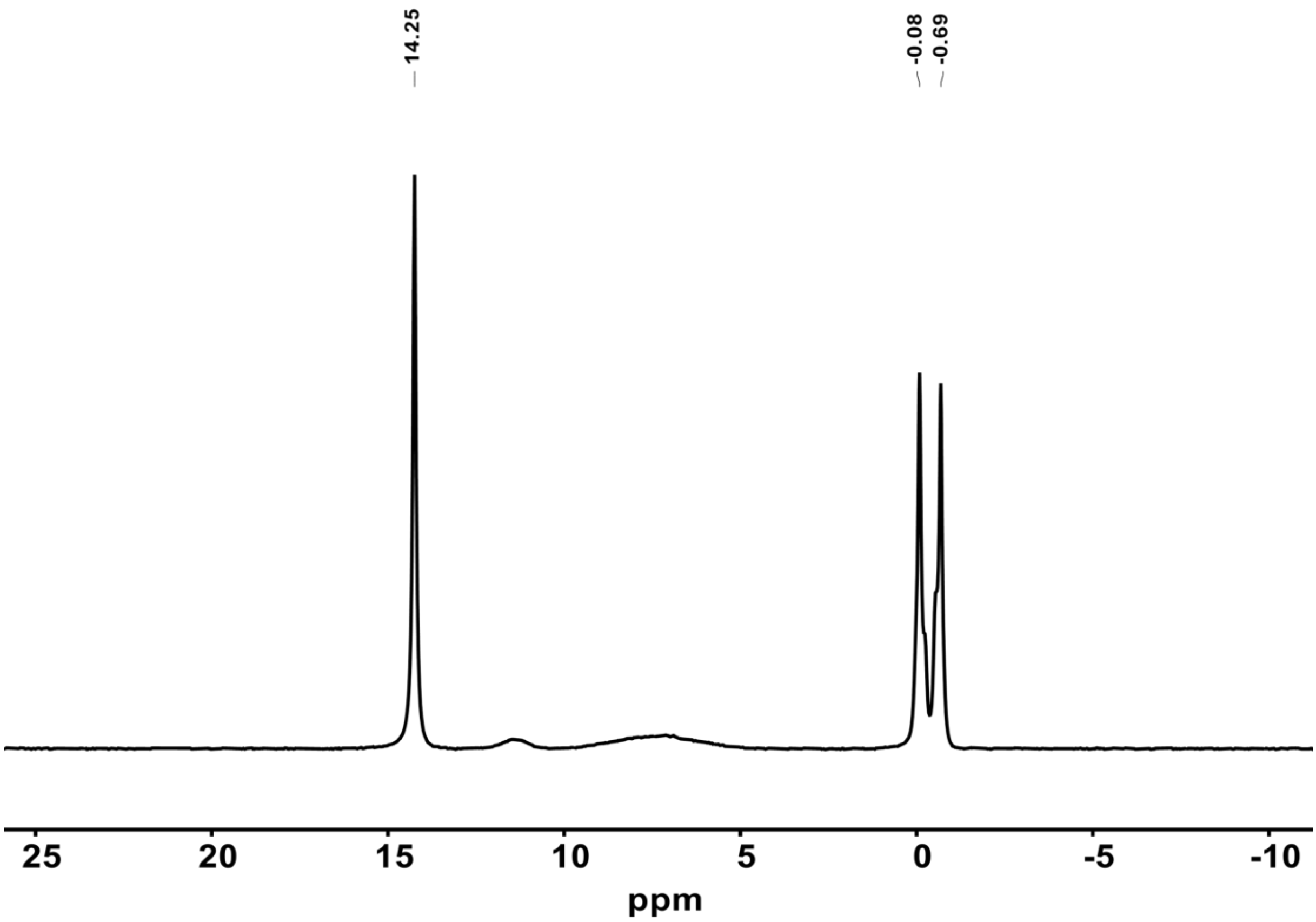


***Figure S21.*** *$^{11}B$ NMR spectrum (128 MHz) of [{HB(μ-hpp)}$_2$(μ-$BH_2$)][B(cat)$_2$] in $CD_2Cl_2$.*

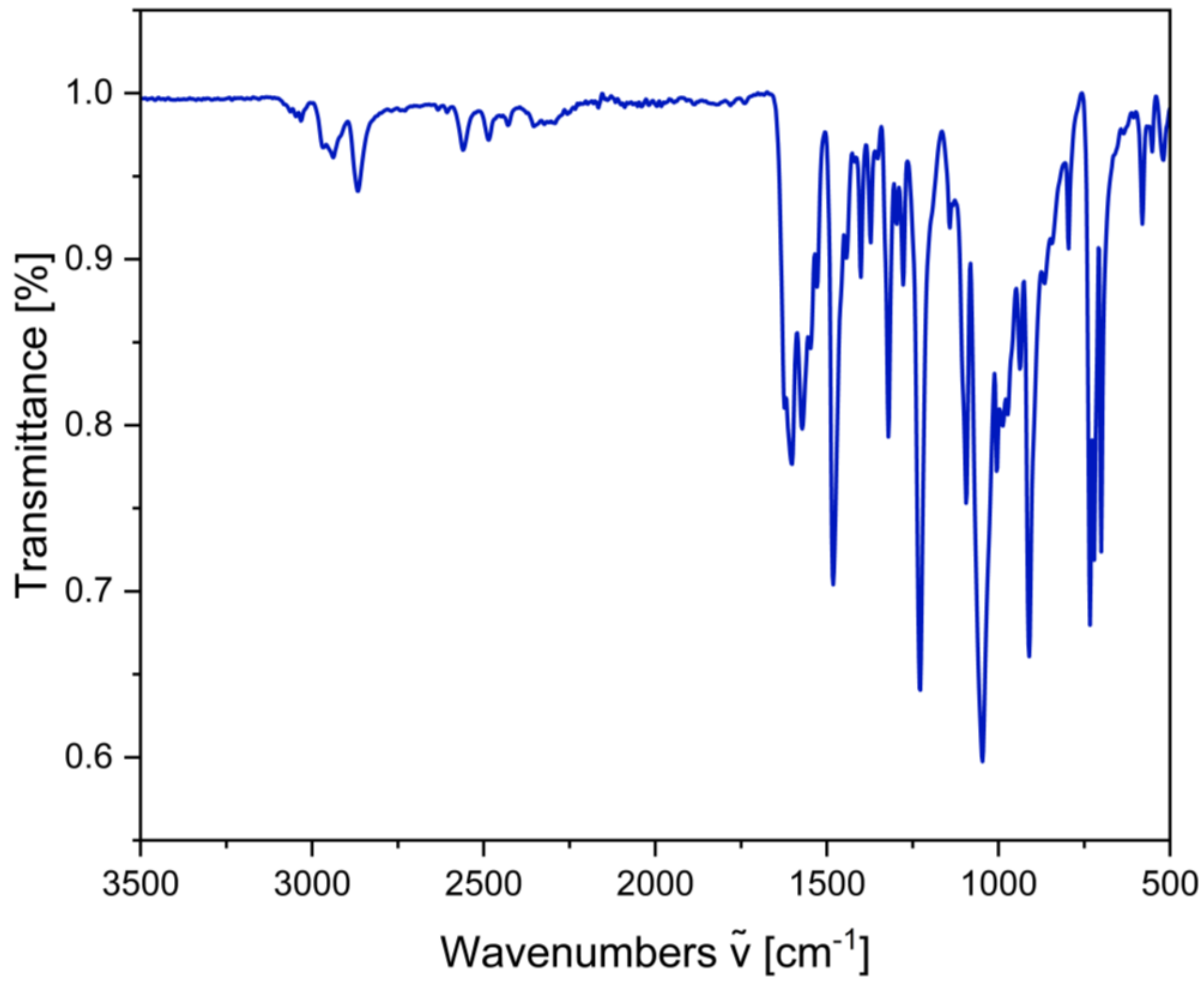


***Figure S22.*** *IR spectrum of [{HB(μ-hpp)}$_2$(μ-BH$_2$)][B(cat)$_2$] as powder.*

## 2.2.2 [{HB($\mu$-hpp)}$_2$($\mu$-BH$_2$)][HB($C_6F_5$)$_3$] **TB2**[HB($C_6F_5$)$_3$]

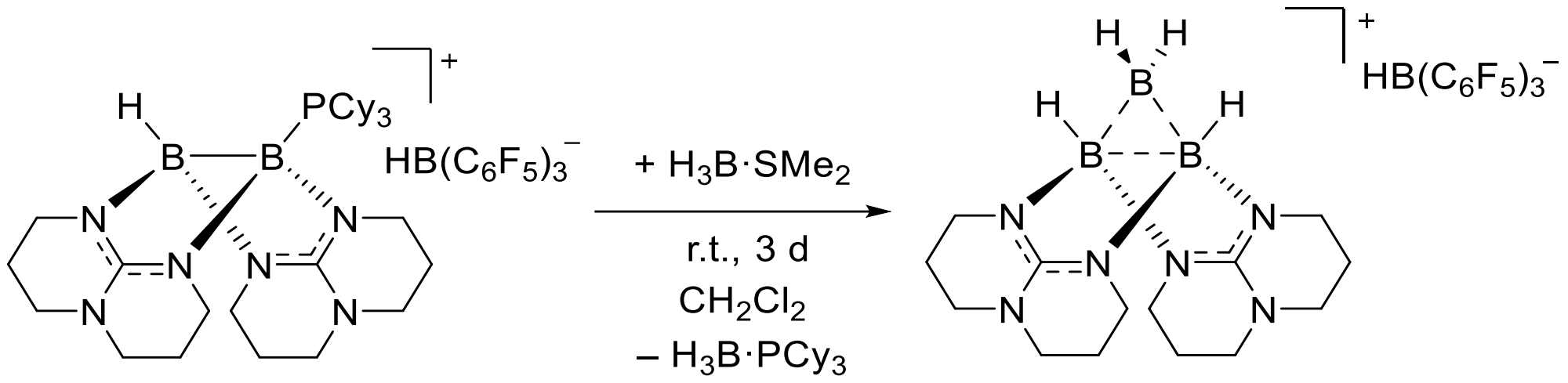


In a Schlenk flask, 300.0 mg of **DB2**[HB($C_6F_5$)$_3$] (274.6 µmol, 1.00 eq.) was dissolved in 4 mL dichloromethane. 52 µL (549.2 µmol, 2.00 eq.) of borane dimethylsulfide was added. After three days of stirring the volatile compounds were removed under reduced pressure. The reaction mixture washed four times with 2 x 5 mL of a 1:1 mixture of diethyl ether and *n*-pentane. The solid was redissolved in 2 mL of dichloromethane and layered with 5 mL of *n*-pentane. The supernatant was removed *via* syringe and the remaining colorless precipitate was dried under vacuum to obtain the product **TB2**[HB($C_6F_5$)$_3$] in a yield of 42.5 mg (52.5 µmol, 19%) as a colorless powder.

**$^{1}$H NMR (600 MHz, $CD_2Cl_2$):**

$\delta$ = 3.33–3.10 (m, 16H, $H_{\text{hpp}}$), 2.00–1.87 (m, 8H, $H_{\text{hpp}}$) ppm.

**$^{1}$H{$^{11}$B} NMR (600 MHz, $CD_2Cl_2$):**

$\delta$ = 3.58–3.53 (m, 1H, $H_{HBCF}$), 3.32–3.10 (m, 16H, $H_{\text{hpp}}$), 2.58–2.34 (m, 2H, B$H$), 2.01–1.87 (m, 8H, $H_{\text{hpp}}$) ppm.

**$^{13}$C{$^{1}$H} NMR (151 MHz, $CD_2Cl_2$):**

$\delta$ = 47.2 ($C_{\text{hpp}}$), 43.6 ($C_{\text{hpp}}$), 21.3 ($C_{\text{hpp}}$) ppm.

The $^{13}$C signal of $C_{\text{q-hpp}}$ could not be observed due to insufficient intensity.

**$^{11}$B NMR (193 MHz, $CD_2Cl_2$):**

$\delta$ = –1.16 (d, $^{1}J_{\text{BH}}$ = 91 Hz, 2B, B$H$), –25.44 (d, $^{1}J_{\text{BH}}$ = 106 Hz, 1B, $B_{\text{HBCF}}$) ppm.

**$^{11}$B{$^{1}$H} NMR (193 MHz, $CD_2Cl_2$):**

$\delta$ = –0.99 (s, 2B, $B$H), –25.33 (s, 1B, $B_{\text{HBCF}}$) ppm.

**$^{19}$F NMR (377 MHz, $CD_2Cl_2$):**

$\delta$ = –134.00 (d, $^{3}J_{\text{FF}}$ = 27 Hz, 6F, $F_{\text{HBCF}}$), –164.65 (t, $^{3}J_{\text{FF}}$ = 20 Hz, 3F, $F_{\text{HBCF}}$), –167.52–167.69 (m, 6F, $F_{\text{HBCF}}$) ppm.

**Mass spectrum (HR-TOF-MALDI$^{+}$):**

m/z = 313.2656 ([M]$^{+}$, 100%).

**IR** (ATR): 2960 (w), 2923 (s), 2873 (m), 2375 (w), 2364 (m), 2340 (w), 1640 (w), 1601 (w), 1574 (w), 1509 (w), 1462 (m), 1326 (w), 1260 (m), 1182 (w), 1066 (s), 1047 (w), 1006 (s), 968 (w), 920 (w), 899 (w), 851 (m), 796 (s), 753 (w), 705 (w), 662 (w), 615 (w), 517 (w) $cm^{-1}$.

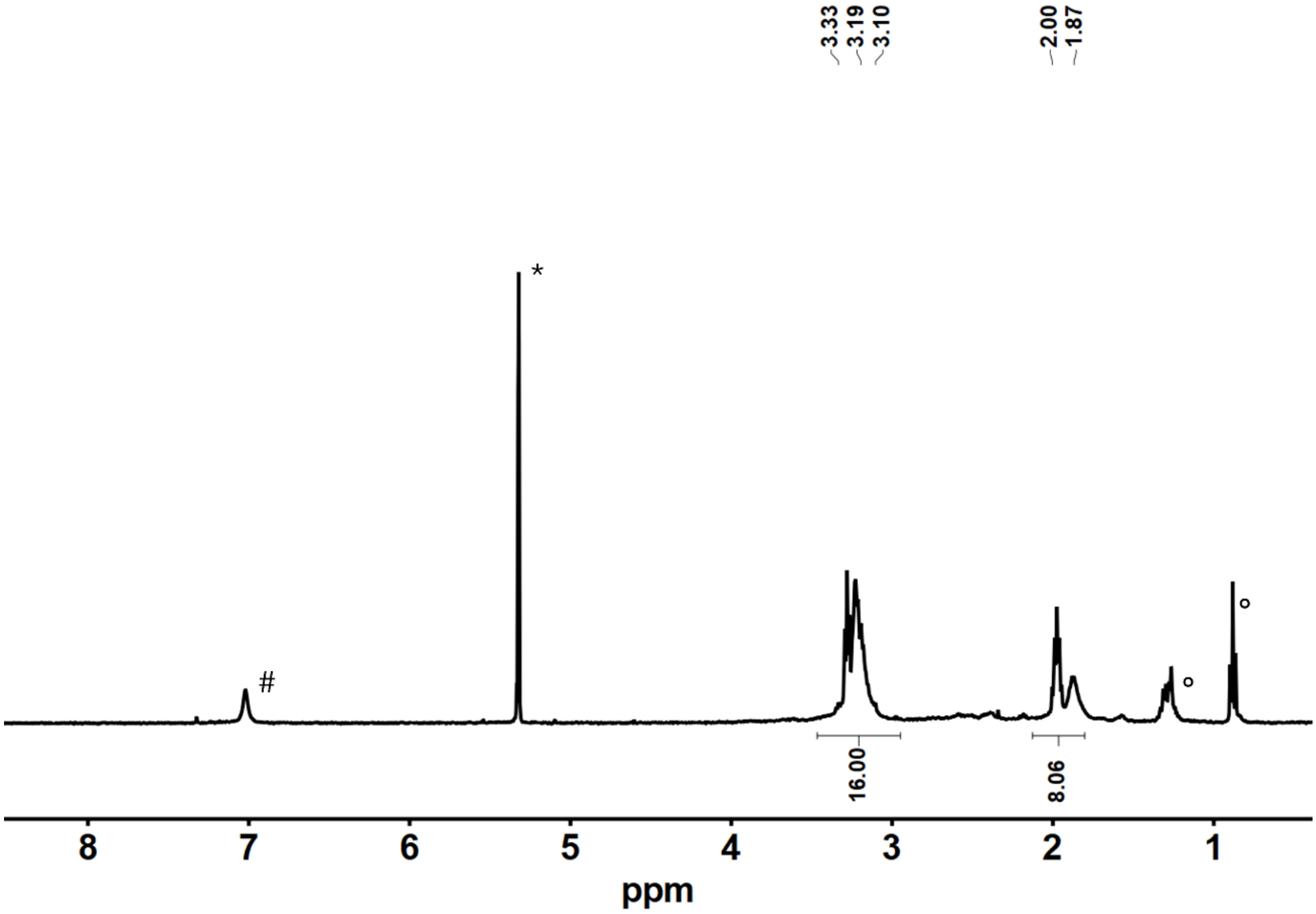


***Figure S23.*** *$^{1}H$ NMR spectrum (600 MHz) of [{HB(μ-hpp)}$_2$(μ-BH$_2$)][HB(C$_6$F$_5$)$_3$] in CD$_2$Cl$_2$. The asterisk * denotes a solvent signal. ° denotes residues of n-hexane. # denotes residues of toluene.*

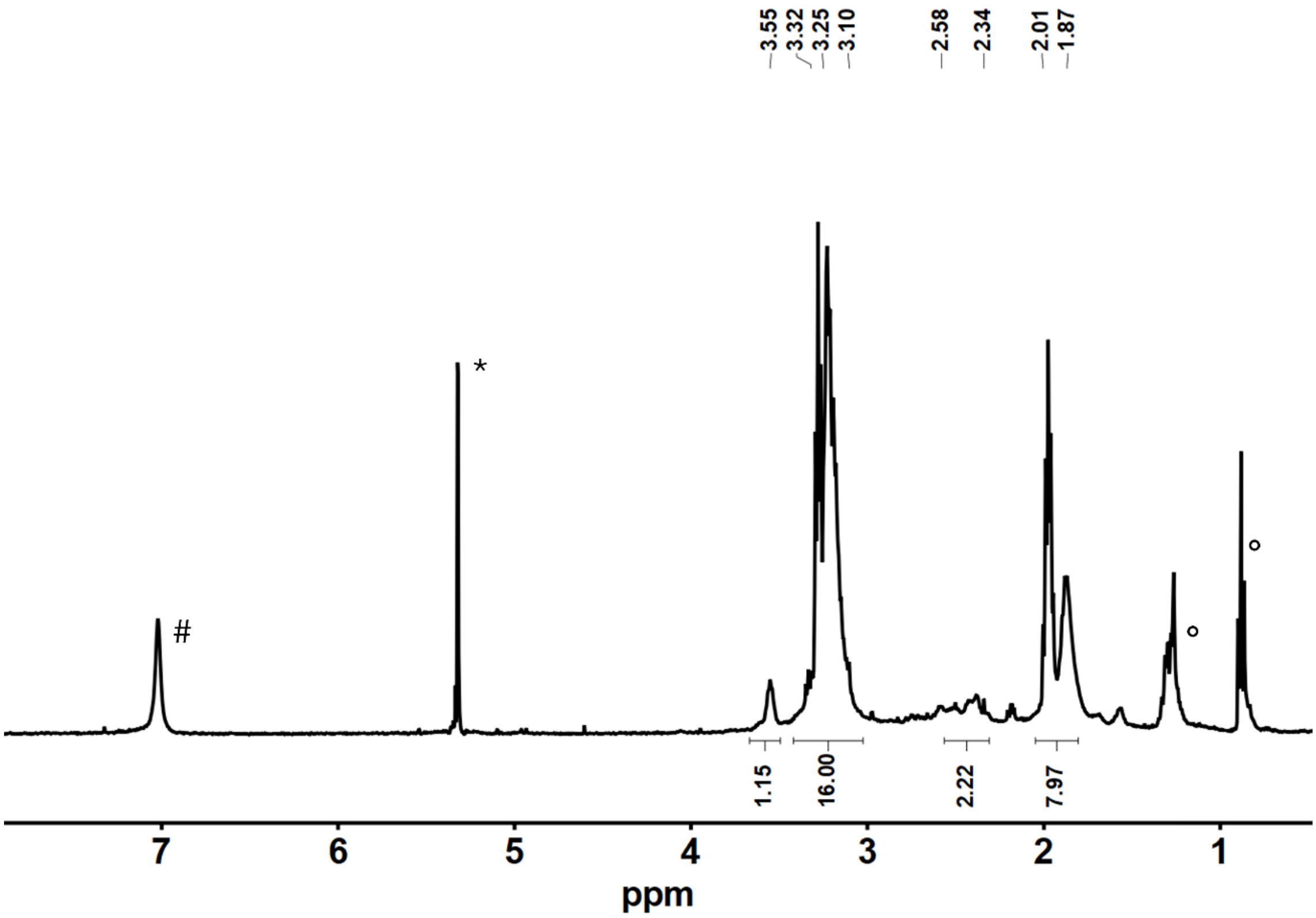


***Figure S24.*** *$^{1}H\{^{11}B\}$ NMR spectrum (400 MHz) of [{HB(μ-hpp)}$_2$(μ-BH$_2$)][HB(C$_6$F$_5$)$_3$] in CD$_2$Cl$_2$. The asterisk * denotes a solvent signal. ° denotes residues of n-hexane. # denotes residues of toluene.*

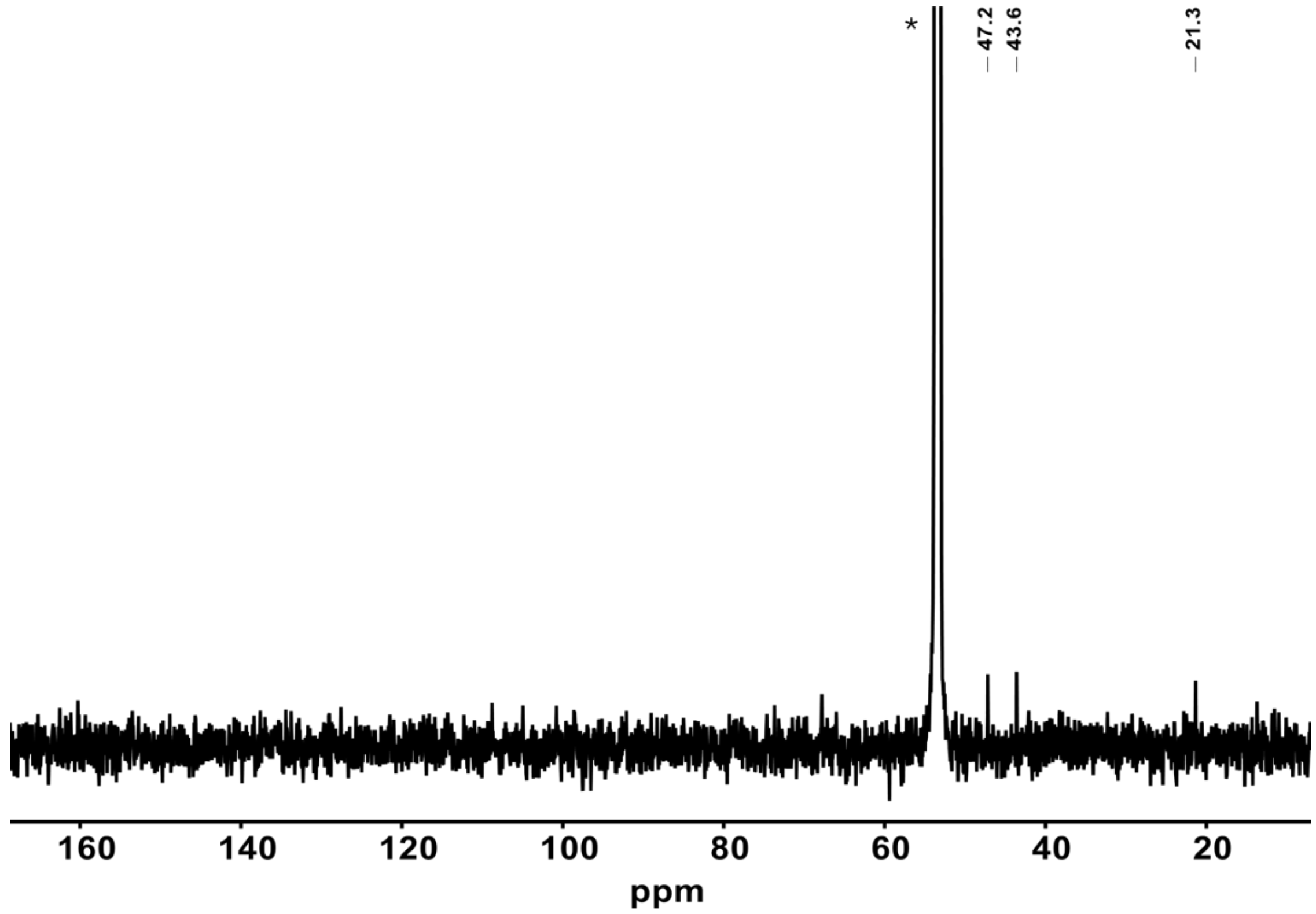


***Figure S25.*** *$^{13}C\{^{1}H\}$ NMR spectrum (100 MHz) of $[\{HB(\mu\text{-hpp})\}_2(\mu\text{-}BH_2)][HB(C_6F_5)_3]]$ in $CD_2Cl_2$. The asterisk * denotes a solvent signal.*

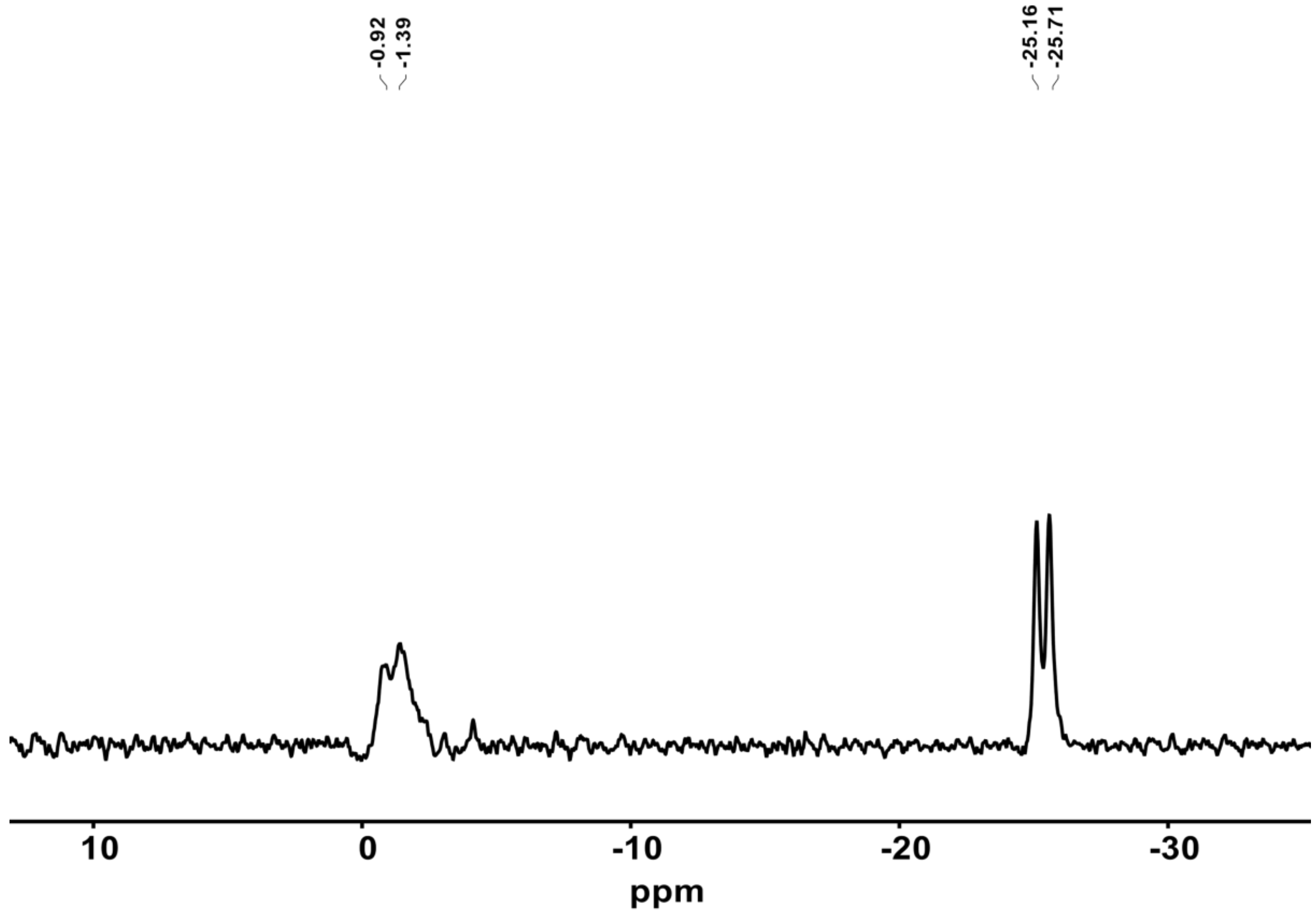


***Figure S26.*** *$^{11}B$ NMR spectrum (128 MHz) of $[\{HB(\mu\text{-hpp})\}_2(\mu\text{-}BH_2)][HB(C_6F_5)_3]$ in $CD_2Cl_2$.*

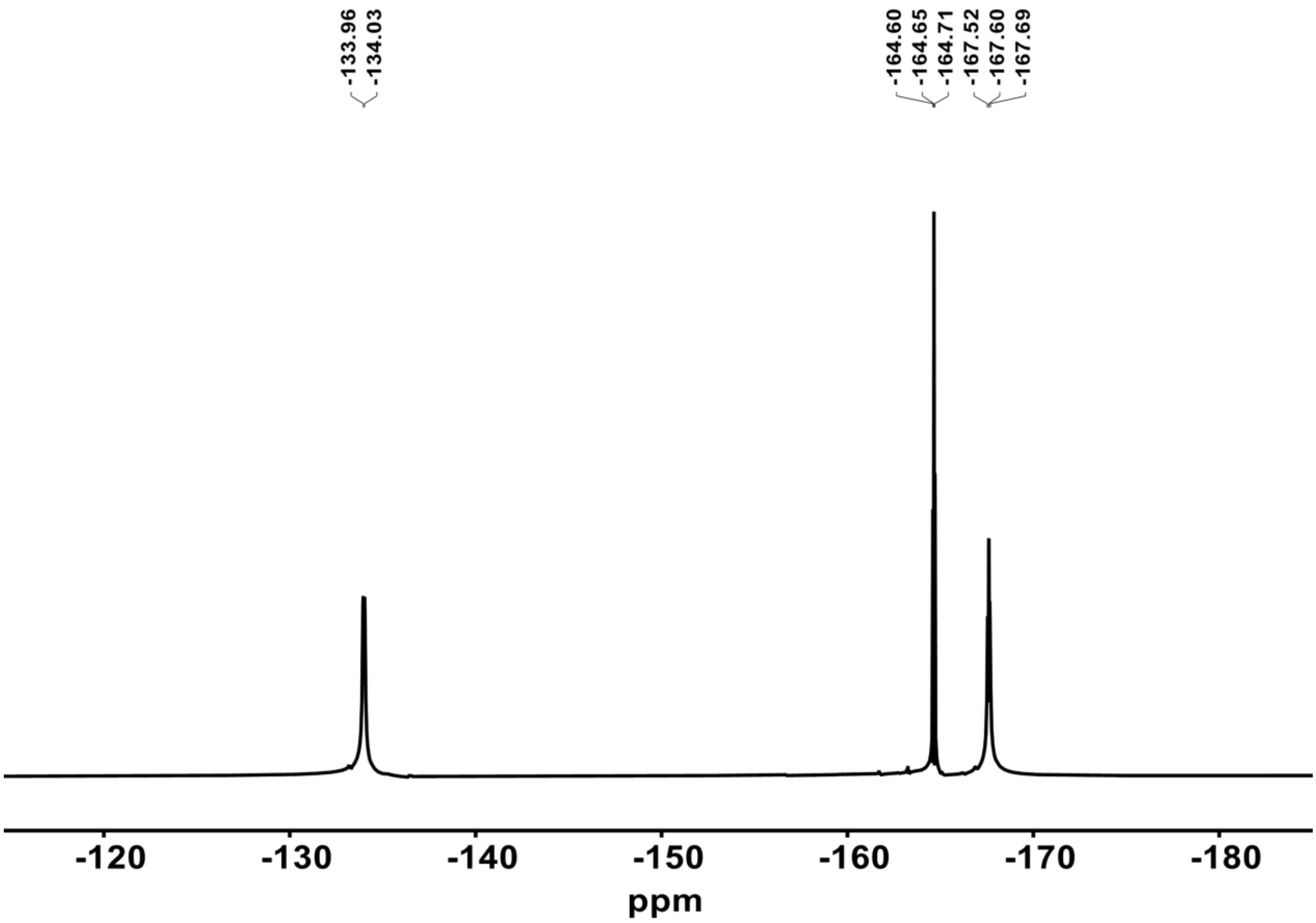


***Figure S27.*** *$^{19}$F NMR spectrum (377 MHz) of [{HB(μ-hpp)}$_2$(μ-BH$_2$)][HB(C$_6$F$_5$)$_3$] in CD$_2$Cl$_2$.*

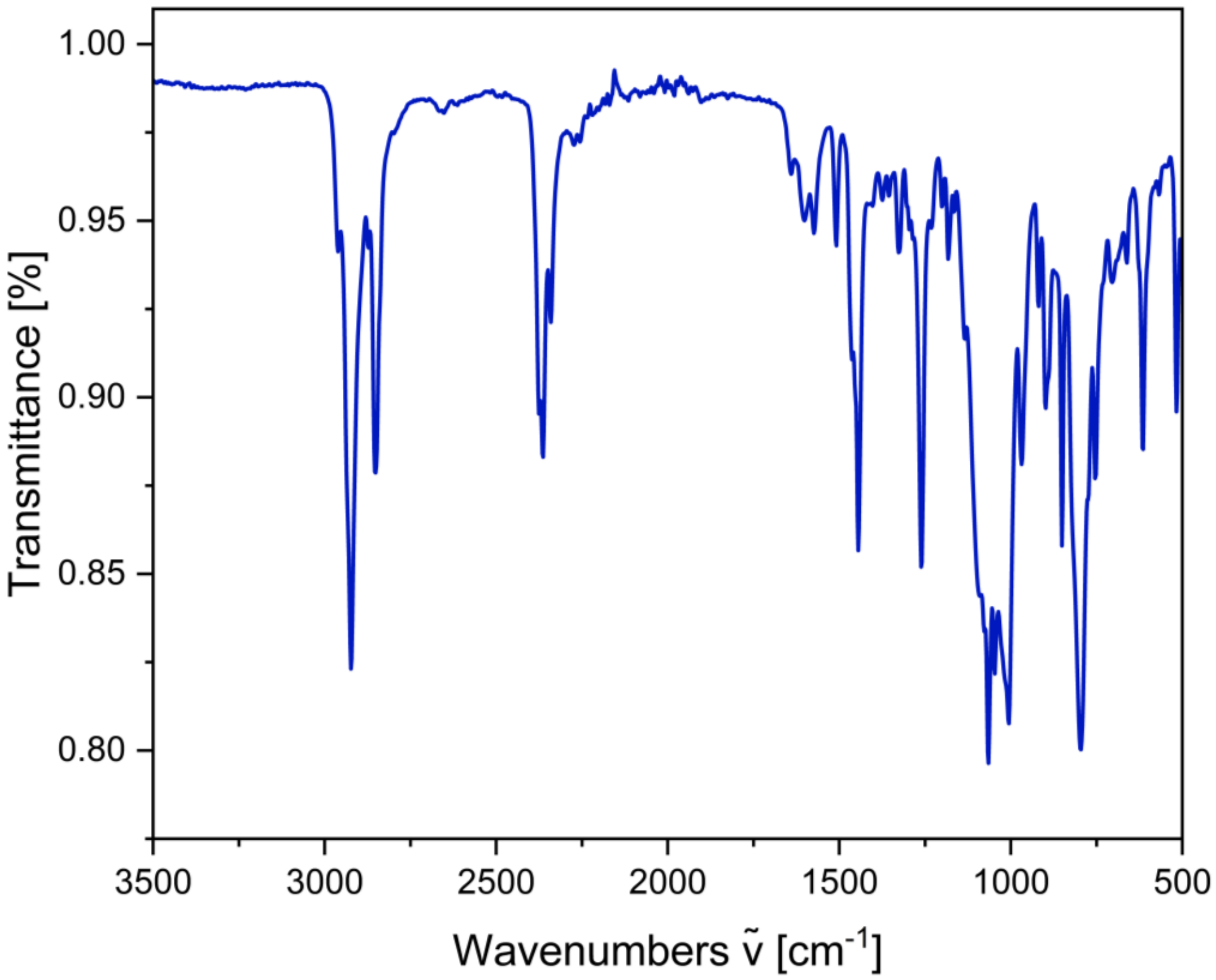


***Figure S28.*** *IR spectrum of [{HB(μ-hpp)}$_2$(μ-BH$_2$)][HB(C$_6$F$_5$)$_3$] as powder.*

## 2.2.3 [{HB(*μ*-hpp)}$_2$(*μ*-BCy$_2$)][HB(C$_6$F$_5$)$_3$] **TB3**[HB(C$_6$F$_5$)$_3$]

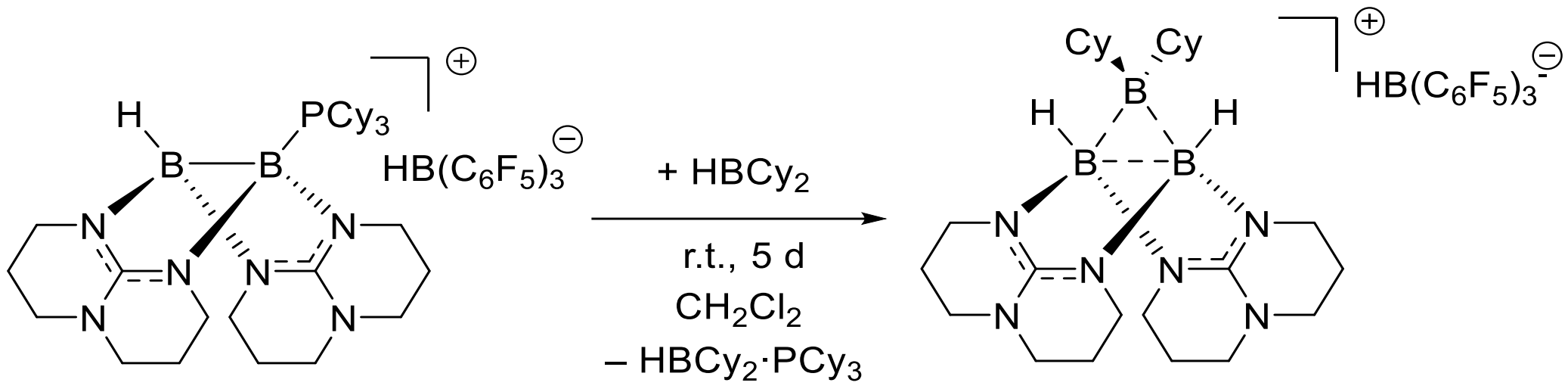


In a Schlenk flask, 68.6 mg of **DB2**[HB(C$_6$F$_5$)$_3$] (62.8 µmol, 1.00 eq.) and 23.5 mg of dicyclohexylborane (131.9 µmol, 2.10 eq.) were dissolved in 4 mL dichloromethane. After five days of stirring the volatile compounds were removed under reduced pressure. The resulting residue was washed with *n*-hexane (9 x 2 mL) and was dried under vacuum to obtain the product **TB3**(HB(C$_6$F$_5$)$_3$) in a yield of 29.8 mg (30.1 µmol, 48%) as a colorless solid.

**$^1$H NMR (600 MHz, CD$_2$Cl$_2$):**

$\delta$ = 3.36–3.04 (m, 16H, $H_{hpp}$), 2.00–1.12 (m, 30H, $H_{hpp,Cy}$) ppm.

**$^{13}$C{$^1$H} NMR (151 MHz, CD$_2$Cl$_2$):**

$\delta$ = 156.5 ($C_{q\text{-}hpp}$), 46.7 ($C_{hpp}$), 44.0 ($C_{hpp}$), 33.5 ($C_{Cy}$), 29.1 ($C_{Cy}$), 28.6 ($C_{Cy}$), 27.5 ($C_{Cy}$), 22.0 ($C_{hpp}$) ppm.

**$^{11}$B NMR (193 MHz, CD$_2$Cl$_2$):**

$\delta$ = 37.92 (br. s, 1B, $B_{BCy2}$), –0.78 (d, $^1J_{BH}$ = 82 Hz, 2B), –25.45 (d, $^1J_{BH}$ = 95 Hz, 1B, $B_{BCF}$) ppm.

**$^{11}$B{$^1$H} NMR (193 MHz, CD$_2$Cl$_2$):**

$\delta$ = 38.02 (br. s, 1B, $B_{BCy2}$), –0.77 (s, 2B), –25.45 (s, 1B, $B_{BCF}$) ppm.

**$^{19}$F NMR (377 MHz, CD$_2$Cl$_2$):**

$\delta$ = –134.00 (d, $^3J_{FF}$ = 23 Hz, 6F, $F_{BCF}$), –164.81 (t, $^3J_{FF}$ = 20 Hz, 3F, $F_{BCF}$), –167.63–167.78 (m, 6F, $F_{BCF}$) ppm.

**Mass spectrum (HR-TOF-MALDI$^+$):**

m/z = 477.4214 ([M–HB(C$_6$F$_5$)$_3$]$^+$, 72.6 %).

**IR** (ATR): 2922 (w), 2852 (s), 2363 (w), 1639 (w), 1600 (w), 1573 (m), 1507 (m), 1458 (s), 1401 (w), 1372 (w), 1321 (m), 1273 (m), 1232 (m), 1189 (w), 1094 (m), 1074 (w), 1047 (w), 965 (s), 907 (w), 857 (w), 820 (w), 758 (w), 727 (w), 659 (w), 590 (w), 567 (w), 509 (w) cm$^{-1}$.

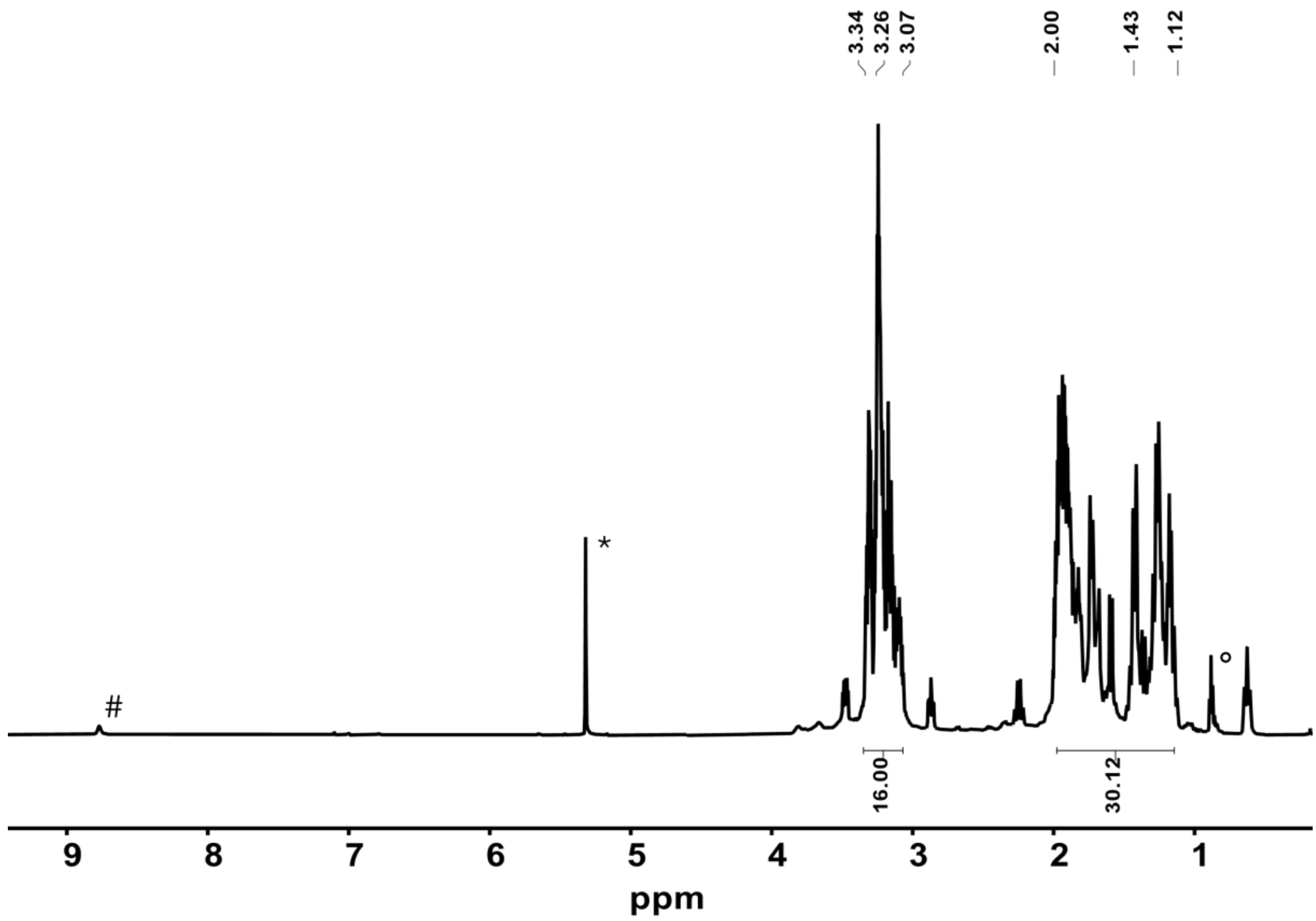


***Figure S29.*** *$^{1}$H NMR spectrum (600 MHz) of [{HB(μ-hpp)}$_2$(μ-BCy$_2$)][HB(C$_6$F$_5$)$_3$] in CD$_2$Cl$_2$. The asterisk * denotes a solvent signal. # denote traces of free hppH$_2^+$ ligand. ° denotes residues of n-hexane.*

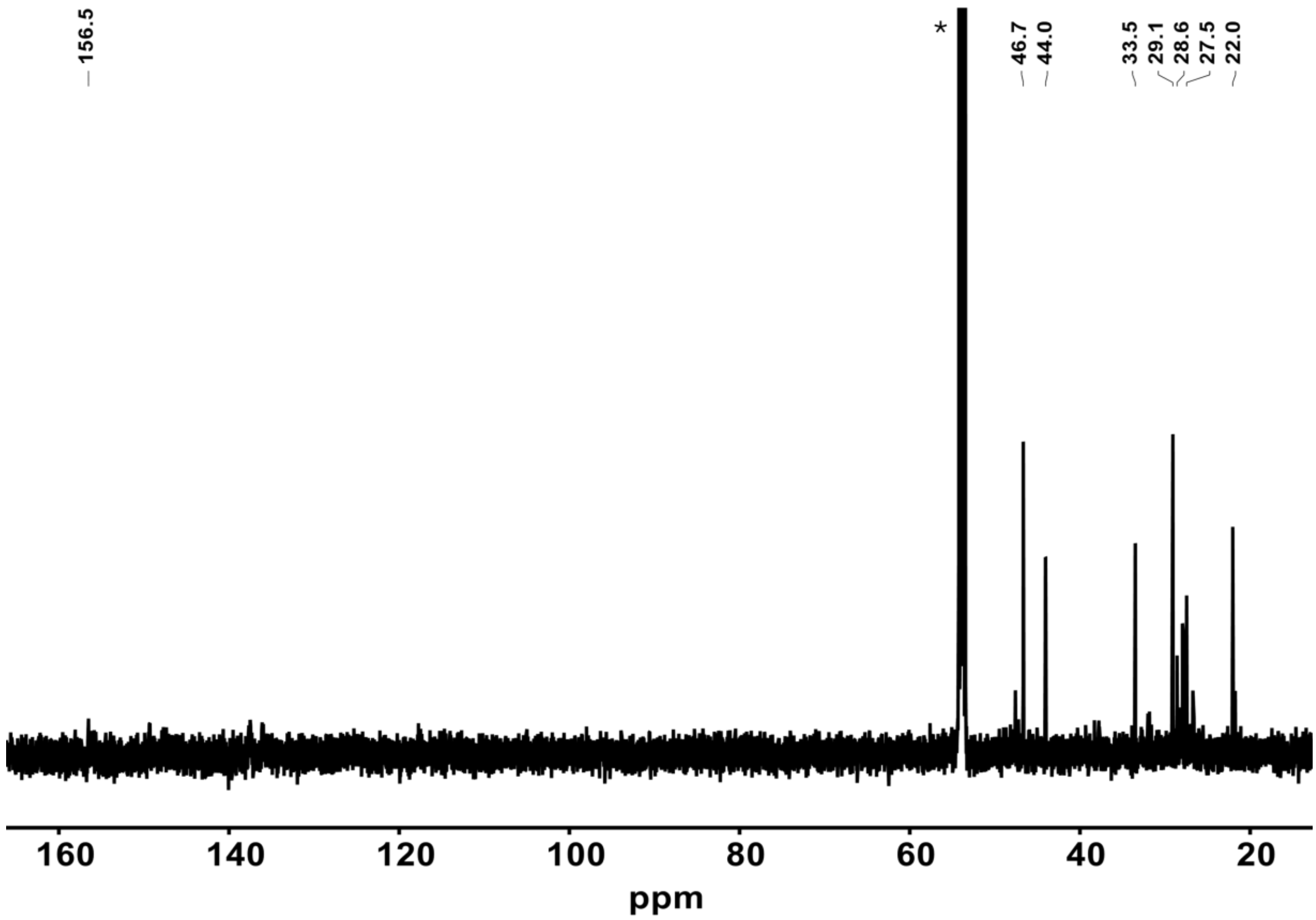


***Figure S30.*** *$^{13}$C{$^{1}$H} NMR spectrum (100 MHz) of [{HB(μ-hpp)}$_2$(μ-BCy$_2$)][HB(C$_6$F$_5$)$_3$]] in CD$_2$Cl$_2$. The asterisk * denotes a solvent signal.*

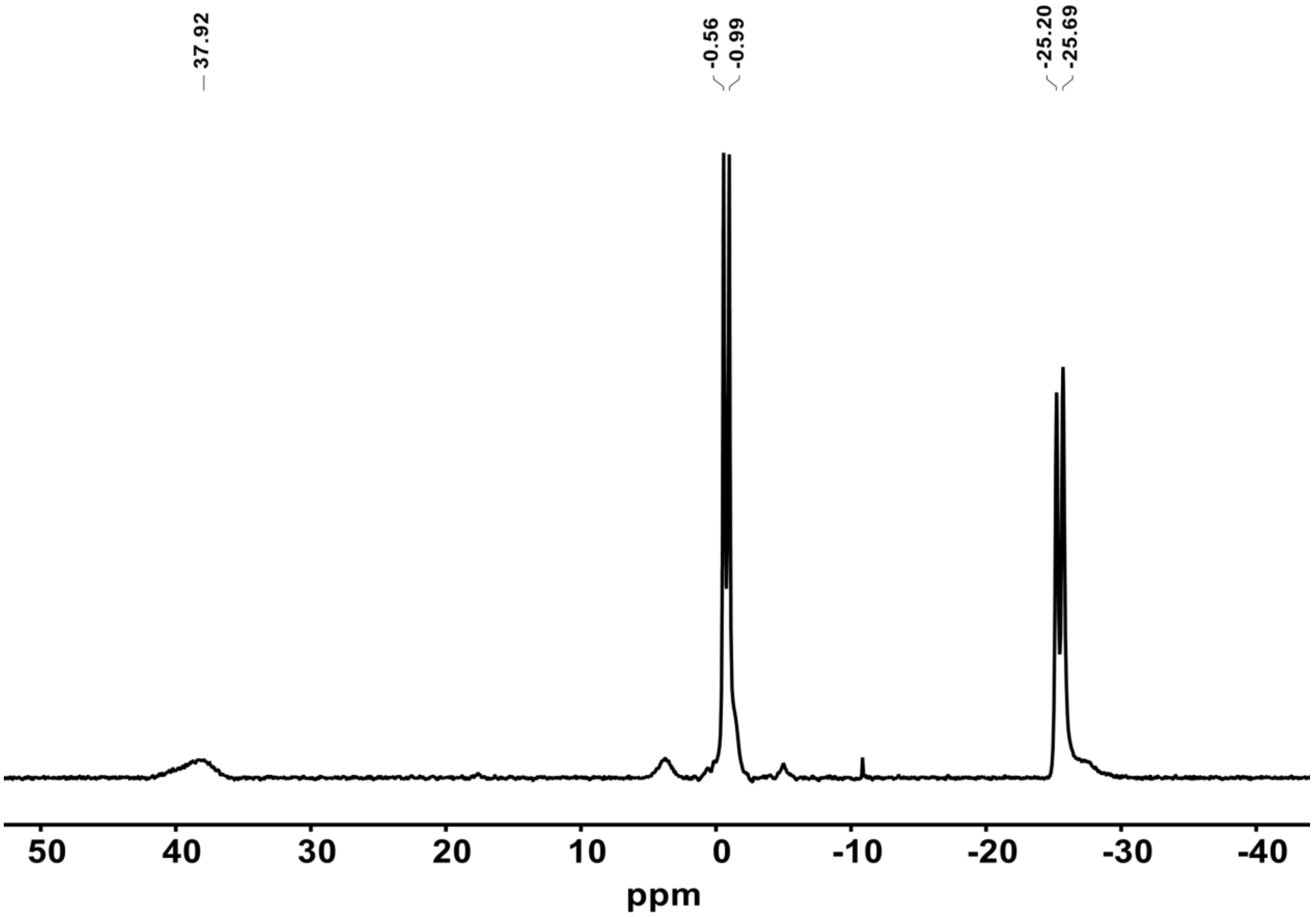


***Figure S31.*** *$^{11}B$ NMR spectrum (128 MHz) of [{HB(μ-hpp)}$_2$(μ-BCy$_2$)][HB($C_6F_5$)$_3$] in $CD_2Cl_2$.*

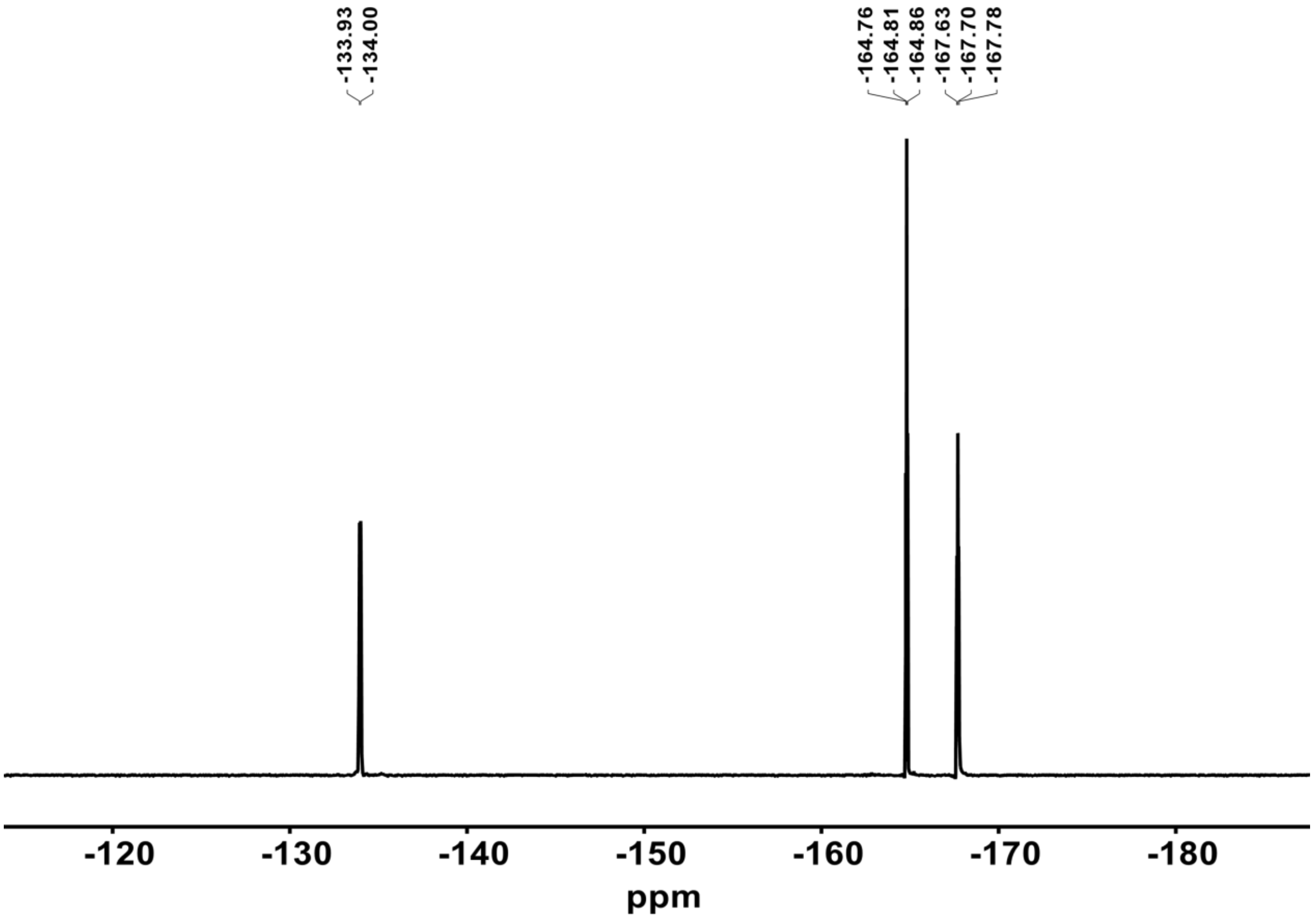


***Figure S32.*** *$^{19}F$ NMR spectrum (377 MHz) of [{HB(μ-hpp)}$_2$(μ-BCy$_2$)][HB($C_6F_5$)$_3$] in $CD_2Cl_2$.*

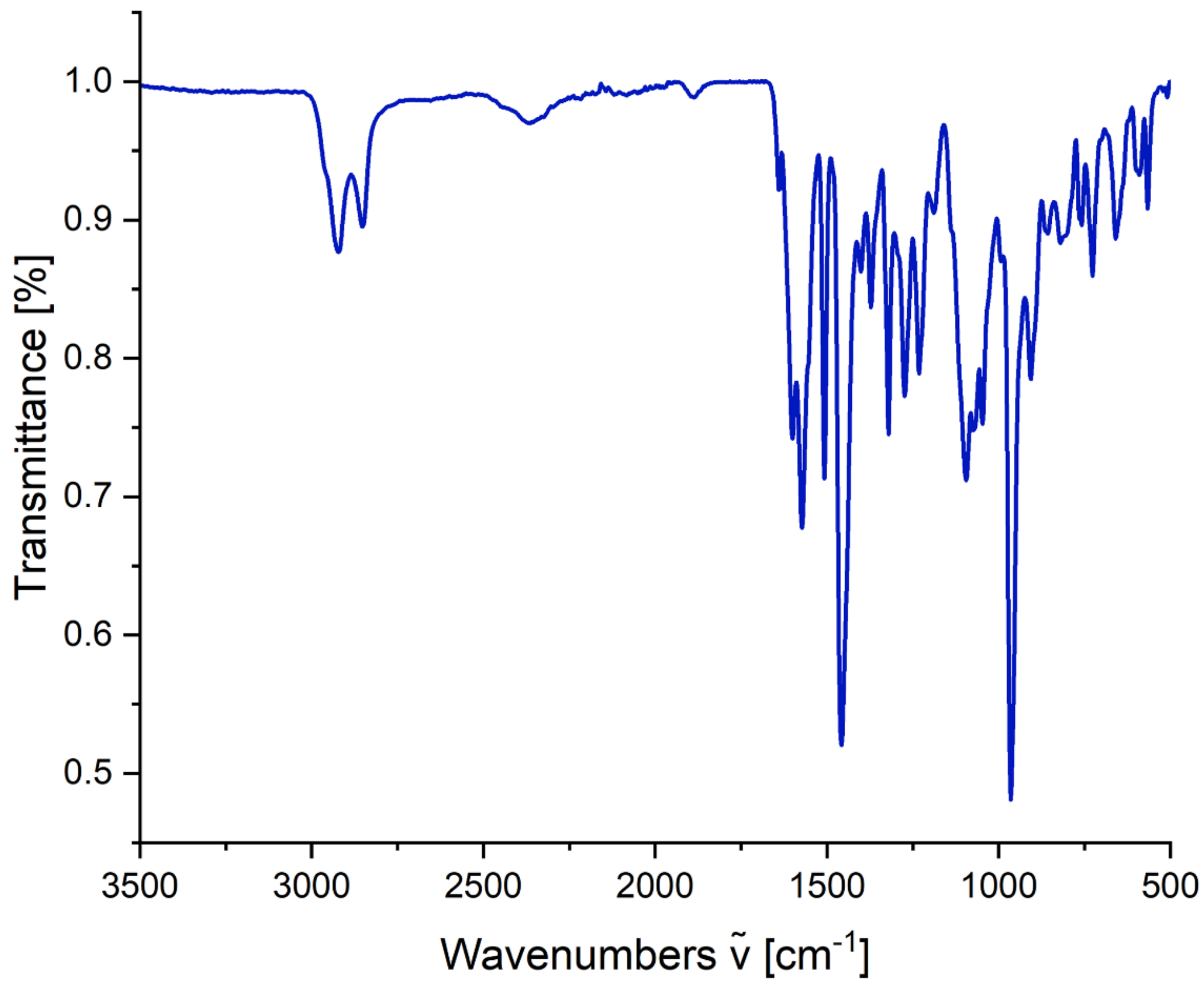


***Figure S33.*** *IR spectrum of [{HB(μ-hpp)}$_2$(μ-BCy$_2$)][HB(C$_6$F$_5$)$_3$] as powder.*

## 2.2.4 [{HB($\mu$-hpp)}$_2$($\mu$-BPin)][HB($C_6F_5$)$_3$] **TB4**[HB($C_6F_5$)$_3$]

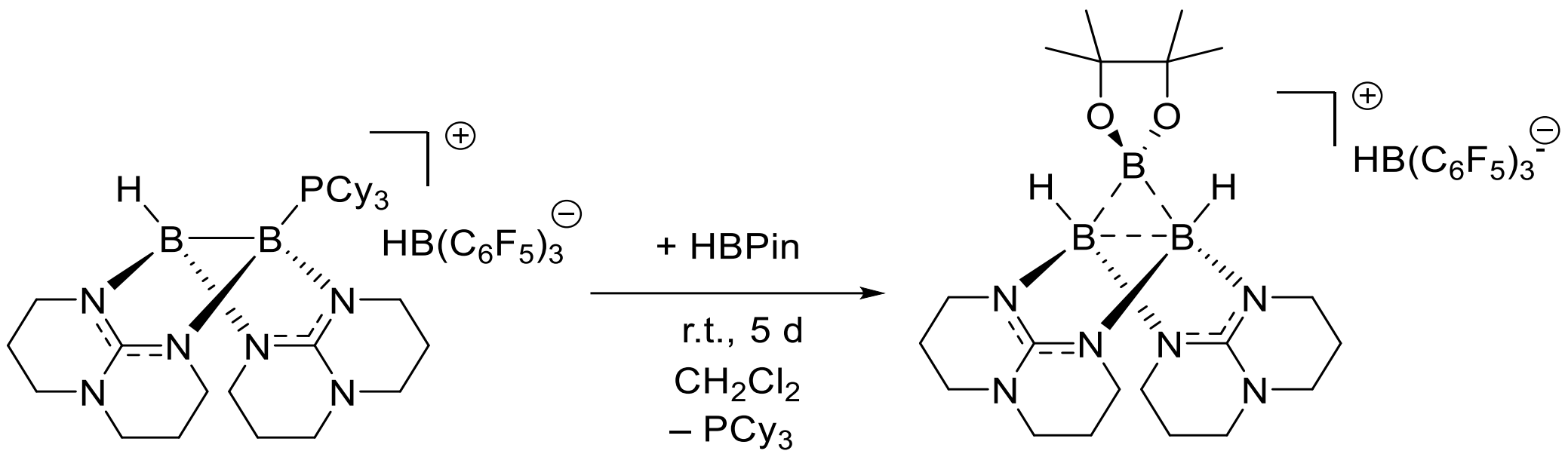


In a Schlenk flask, 170.0 mg of **DB2**[HB($C_6F_5$)$_3$] (155.6 µmol, 1.00 eq.) and 45.2 µL of pinacolborane (311.2 µmol, 2.00 eq.) were dissolved in 2 mL dichloromethane. After five days of stirring the completion of the reaction could be confirmed by $^{11}$B and $^{31}$P NMR spectroscopy. The volatile compounds were removed under reduced pressure. The resulting residue was washed four times with 2 mL of *n*-hexane and was dried under vacuum. **TB4**[HB($C_6F_5$)$_3$] was found to be unstable, undergoing spontaneous decomposition over time, as monitored by $^{11}$B NMR spectroscopy. Attempts to isolate the product in analytically pure form were therefore unsuccessful.

**$^{11}$B NMR (193 MHz, $CD_2Cl_2$):**

$\delta$ = –1.07 (d, $^1J_{BH}$ = 134 Hz, 2B), –25.45 (d, $^1J_{BH}$ = 93 Hz, 1B, $B_{BCF}$) ppm.

**$^{11}$B{$^1$H} NMR (193 MHz, $CD_2Cl_2$):**

$\delta$ = –1.05 (s, 2B), –25.45 (s, 1B, $B_{BCF}$) ppm.

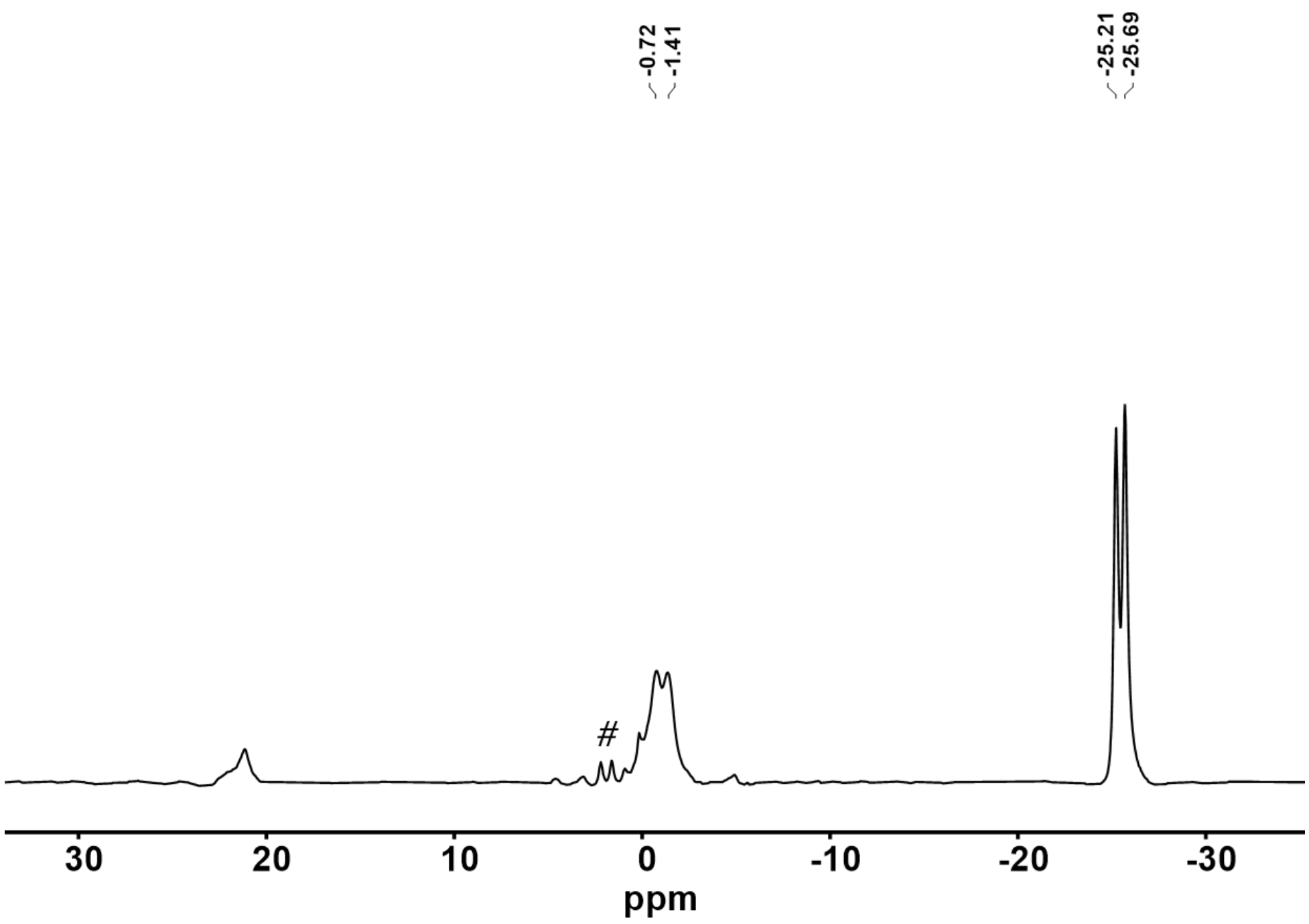


***Figure S34.*** *$^{11}$B NMR spectrum (193 MHz) of [{HB(µ-hpp)}$_2$(µ-BPin)][HB($C_6F_5$)$_3$] in $CD_2Cl_2$. # denotes decomposition product.*

## 2.2.5 [{HB($\mu$-hpp)}$_2$($\mu$-BCat)][HB($C_6F_5$)$_3$] **TB5**[HB($C_6F_5$)$_3$]

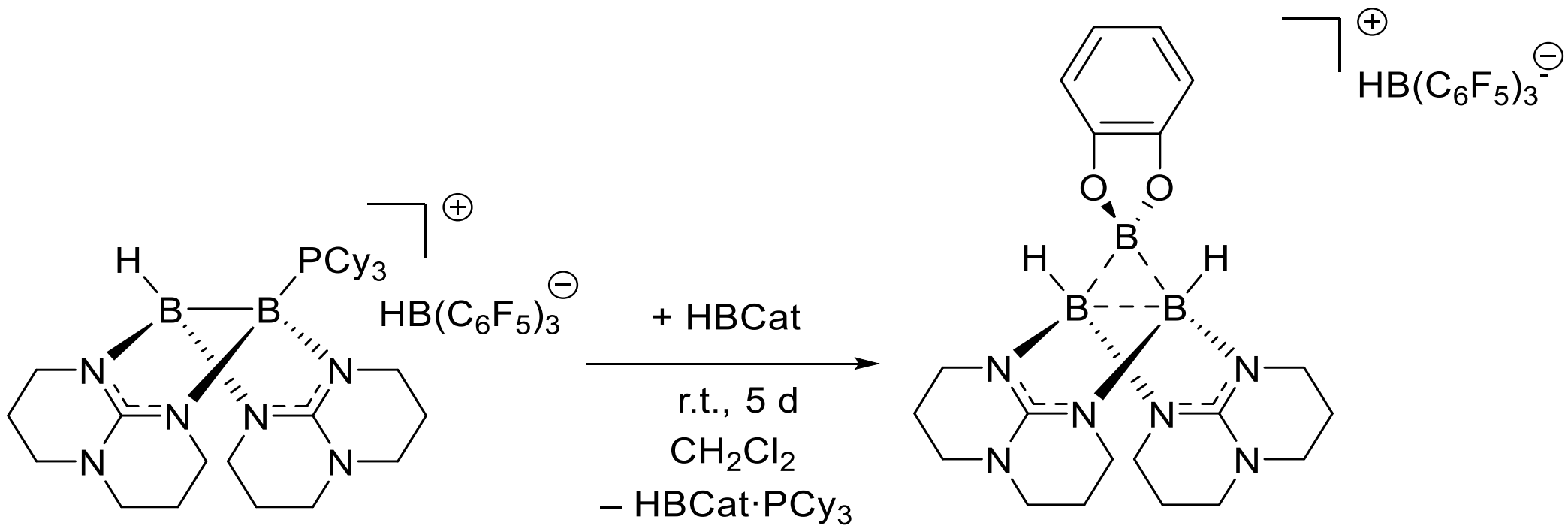


In a Schlenk flask, 55.0 mg of **DB2**[HB($C_6F_5$)$_3$] (50.4 µmol, 1.00 eq.) and 10.7 µL of catecholborane (100.7 µmol, 2.00 eq.) were dissolved in 2 mL dichloromethane. After five days of stirring the completion of the reaction could be confirmed by $^{11}$B and $^{31}$P NMR spectroscopy The volatile compounds were removed under reduced pressure. The resulting residue was washed four times with 2 mL of *n*-hexane and was dried under vacuum. **TB5**[HB($C_6F_5$)$_3$] was found to be unstable, undergoing spontaneous decomposition over time, as monitored by $^{11}$B NMR spectroscopy. Attempts to isolate the product in analytically pure form were therefore unsuccessful.

**$^{11}$B NMR (193 MHz, $CD_2Cl_2$):**
$\delta$ = –1.04 (d, $^1J_{BH}$ = 128 Hz, 2B), –25.42 (d, $^1J_{BH}$ = 92 Hz, 1B, $B_{BCF}$) ppm.

**$^{11}$B{$^1$H} NMR (193 MHz, $CD_2Cl_2$):**
$\delta$ = –1.06 (s, 2B), –25.42 (s, 1B, $B_{BCF}$) ppm.

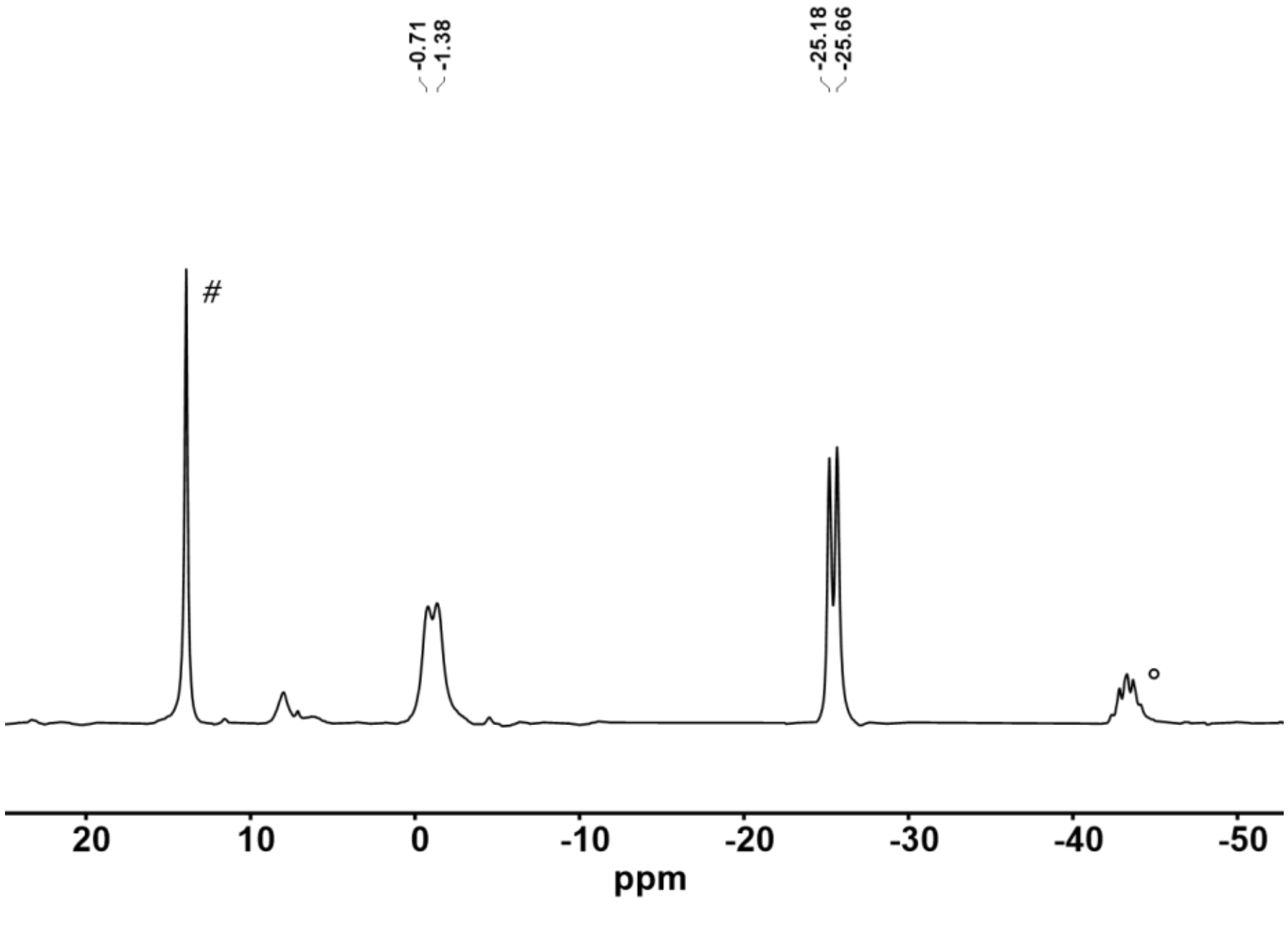


***Figure S35.*** *$^{11}$B NMR spectrum (193 MHz) of [{HB(µ-hpp)}$_2$(µ-BCat$_2$)][HB($C_6F_5$)$_3$] in $CD_2Cl_2$. . # denotes decomposition product. ° denote traces of HBCat-phosphine adduct.*

## 2.2.4 [{HB(μ-hpp)}$_2$(μ-BH$_2$)][B(cat)$_2$] **TB2**[B(cat)$_2$]

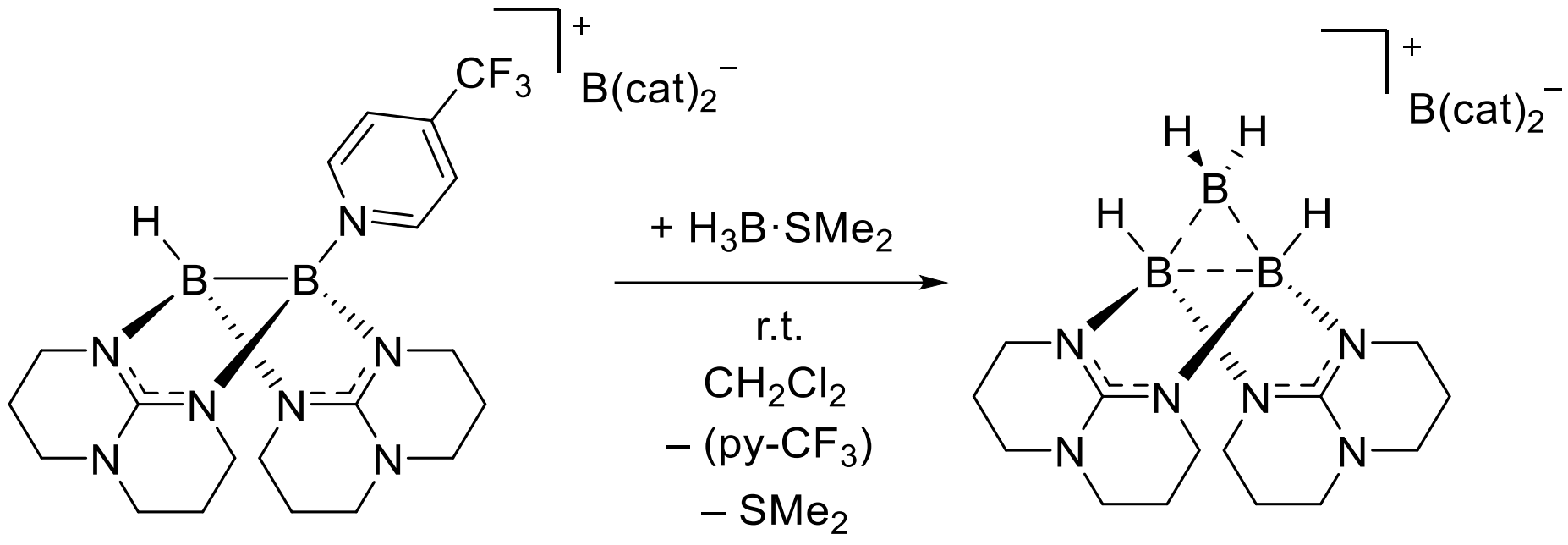


In a dry *J. Young* NMR tube, 10.0 mg of **DB7**[B(cat)$_2$] (13.5 μmol, 1.00 eq.) was dissolved in 0.6 mL deuterated dichloromethane. 3.8 μL (40.4 μmol, 3.00 eq.) of borane dimethylsulfide was added, resulting in a color change from orange to colorless during the course of the reaction. The formation of **TB2**[B(cat)$_2$] was monitored *via* NMR spectroscopy at room temperature over different reaction times.

**$^{11}$B NMR (193 MHz, CD$_2$Cl$_2$):**

$\delta$ = 14.24 (s, 1B, $B_{cat}$), –0.39 (d, $^1J_{BH}$ = 78 Hz, 2B, *B*H) ppm.

**$^{11}$B{$^1$H} NMR (193 MHz, CD$_2$Cl$_2$):**

$\delta$ = 14.24 (s, 1B, $B_{cat}$), –0.38 (s, 2B, *B*H) ppm.

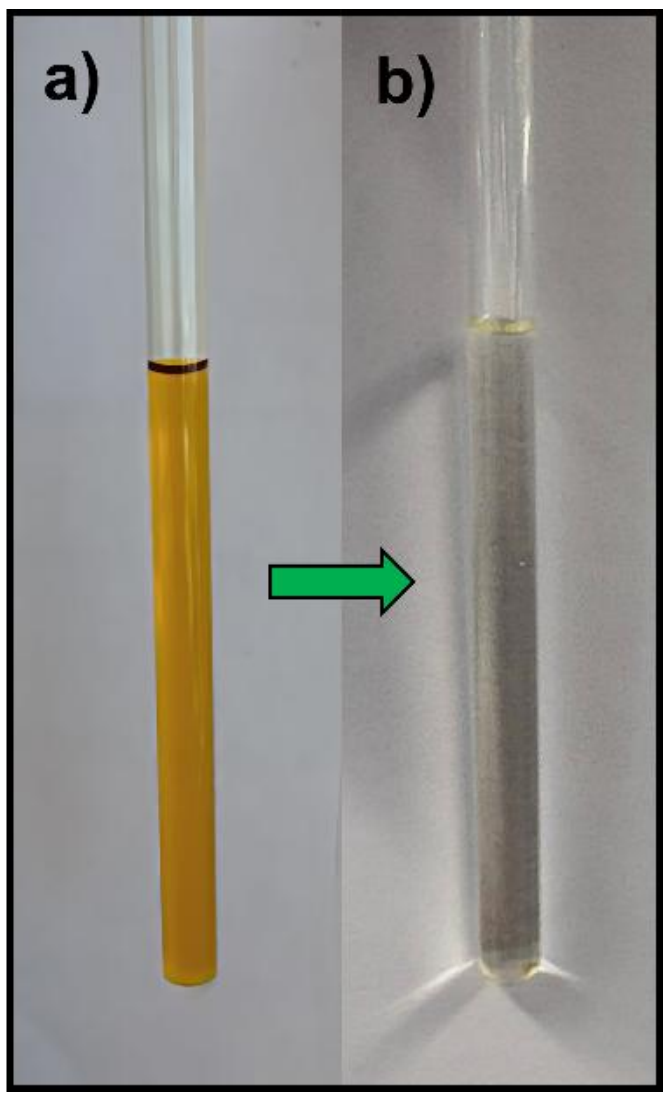


***Figure S36**. Color comparison of the conversion of monoadduct **DB7**[B(cat)$_2$] with borane dimethylsulfide to triborirane **TB2**[B(cat)$_2$] in CD$_2$Cl$_2$ after a) 1 min and b) 1 h.*

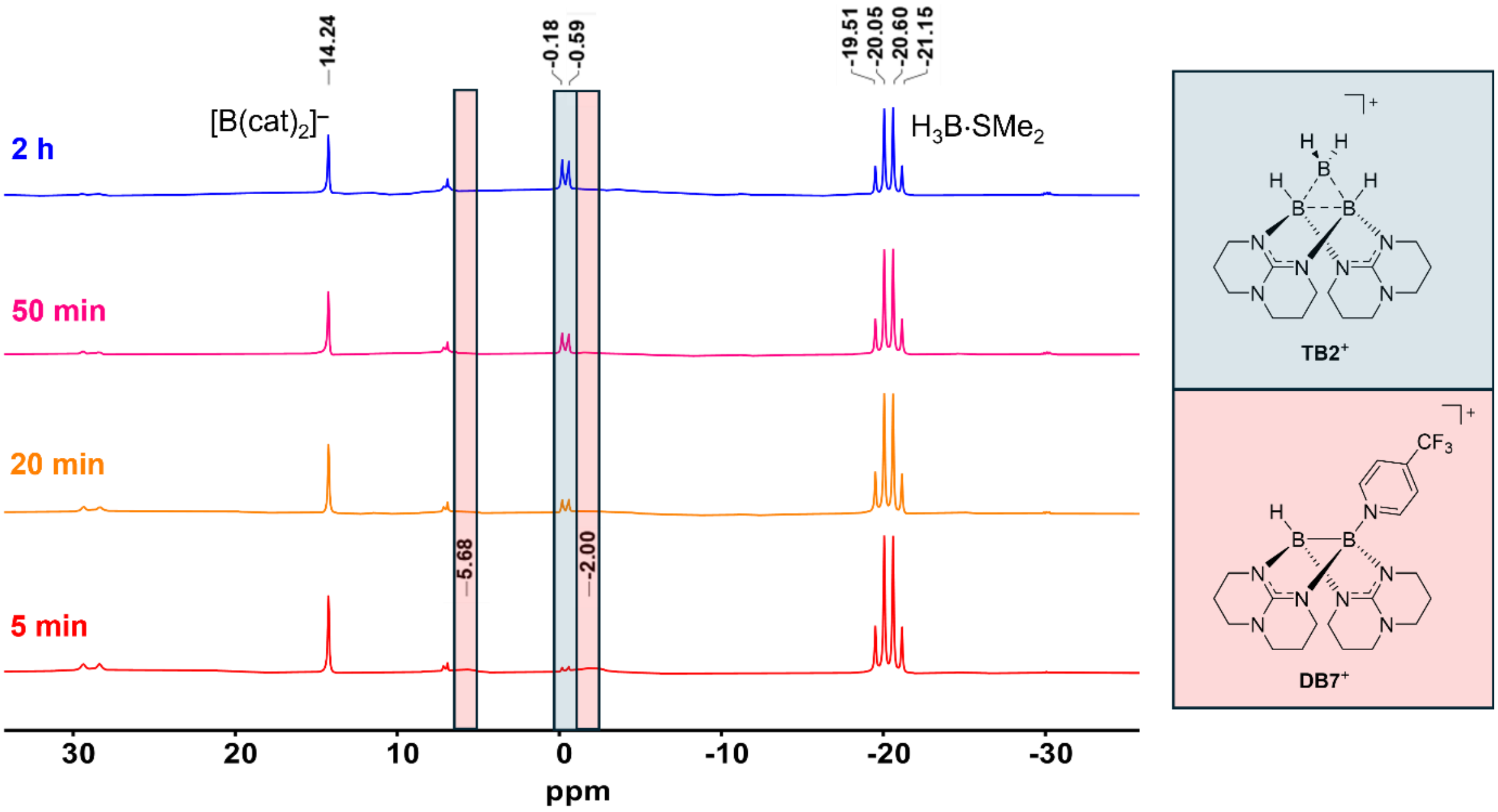


***Figure S37.*** Reaction of the monocationic diborane **DB7**[B(cat)$_2$] with $H_3B\cdot SMe_2$ to give the cationic triboron compound **TB2**[B(cat)$_2$].

# 2.3 *N*-heterocyclic ligand substituted diboranes

## 2.3.1 [(DMAP)(H){B(*μ*-hpp)}$_2$][HB($C_6F_5$)$_3$] **DB5**[HB($C_6F_5$)$_3$]

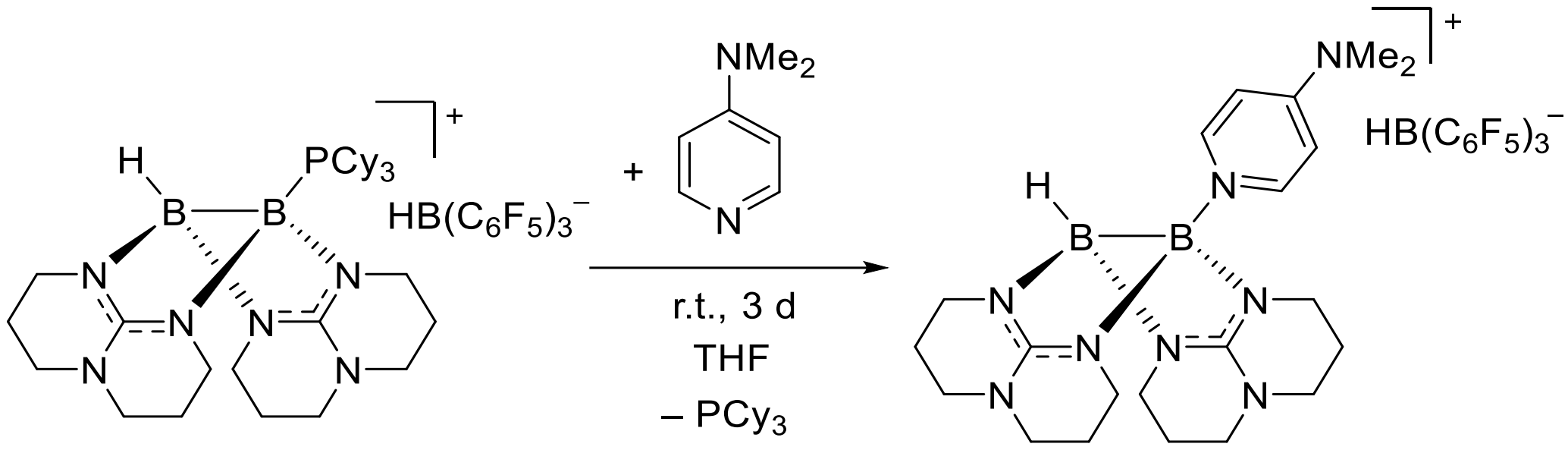


In a Schlenk flask, 104 mg of **DB2**[HB($C_6F_5$)$_3$] (95.4 μmol, 1.00 eq.) and 4-dimethylaminopyridine (114 μmol, 1.20 eq.) were dissolved in 5 mL tetrahydrofuran. After three days of stirring at room temperature the solvent was removed under reduced pressure. The resulting colorless residue was extracted with diethyl ether (3 x 1 mL). The combined extracts were layered with *n*-pentane and stored at –30 °C overnight during which a colorless precipitate formed. The solvent was removed *via* syringe and the crude product was washed with *n*-pentane (3 x 1 mL) and dried under vacuum to obtain **DB5**[HB($C_6F_5$)$_3$] in a yield of 31.0 mg (31.7 μmol, 33%) as a colorless solid.

**$^{1}$H NMR (600 MHz, $CD_2Cl_2$):**

$\delta$ = 7.72 (d, $^{3}J_{HH}$ = 8 Hz, 2H, $H_{DMAP}$), 6.65 (d, $^{3}J_{HH}$ = 8 Hz, 2H, $H_{DMAP}$), 3.11 (s, 12H, $H_{DMAP\text{-}Me}$), 3.29–3.00 (m, 16H, $H_{hpp}$), 2.00–1.80 (m, 8H, $H_{hpp}$) ppm.

**$^{13}$C{$^{1}$H} NMR (151 MHz, $CD_2Cl_2$):**

$\delta$ = 158.6 ($C_{q\text{-}hpp}$), 155.4 ($C_{q\text{-}DMAP}$), 142.5 ($C_{DMAP}$), 107.8 ($C_{DMAP}$), 47.6 ($C_{hpp}$), 47.4 ($C_{hpp}$), 44.7 ($C_{hpp}$), 40.3 ($C_{hpp}$), 39.8 ($C_{DMAP\text{-}Me}$), 23.1 ($C_{hpp}$), 22.6 ($C_{hpp}$) ppm.

**$^{11}$B NMR (193 MHz, $CD_2Cl_2$):**

$\delta$ = 4.86 (s, 1B), –2.99 (s, 1B), –25.45 (d, $^{1}J_{BH}$ = 91 Hz, 1B, $B_{HBCF}$) ppm.

**$^{11}$B{$^{1}$H} NMR (193 MHz, $CD_2Cl_2$):**

$\delta$ = 4.62 (s, 1B), –3.05 (s, 1B), –25.43 (s, 1B, $B_{HBCF}$) ppm.

**$^{19}$F NMR (377 MHz, $CD_2Cl_2$):**

$\delta$ = –133.96 (d, $^{3}J_{FF}$ = 27 Hz, 6F, $F_{HBCF}$), –164.78 (t, $^{3}J_{FF}$ = 20 Hz, 3F, $F_{HBCF}$), –167.57–167.73 (m, 6F, $F_{HBCF}$) ppm.

**Mass spectrum (HR-TOF-MALDI$^{+}$):**

m/z = 421.3183 ([M]$^{+}$, 100%).

**IR** (ATR): 2962 (w), 2860 (w), 2326 (w), 1638 (m), 1581 (w), 1558 (w), 1508 (m), 1457 (s), 1394 (w), 1370 (w), 1320 (w), 1260 (m), 1221 (w), 1181 (w), 1088 (w), 1041 (w), 964 (s), 854 (w), 796 (w), 740 (s), 661 (w), 600 (w), 567 (w), 520 (w) $cm^{-1}$.

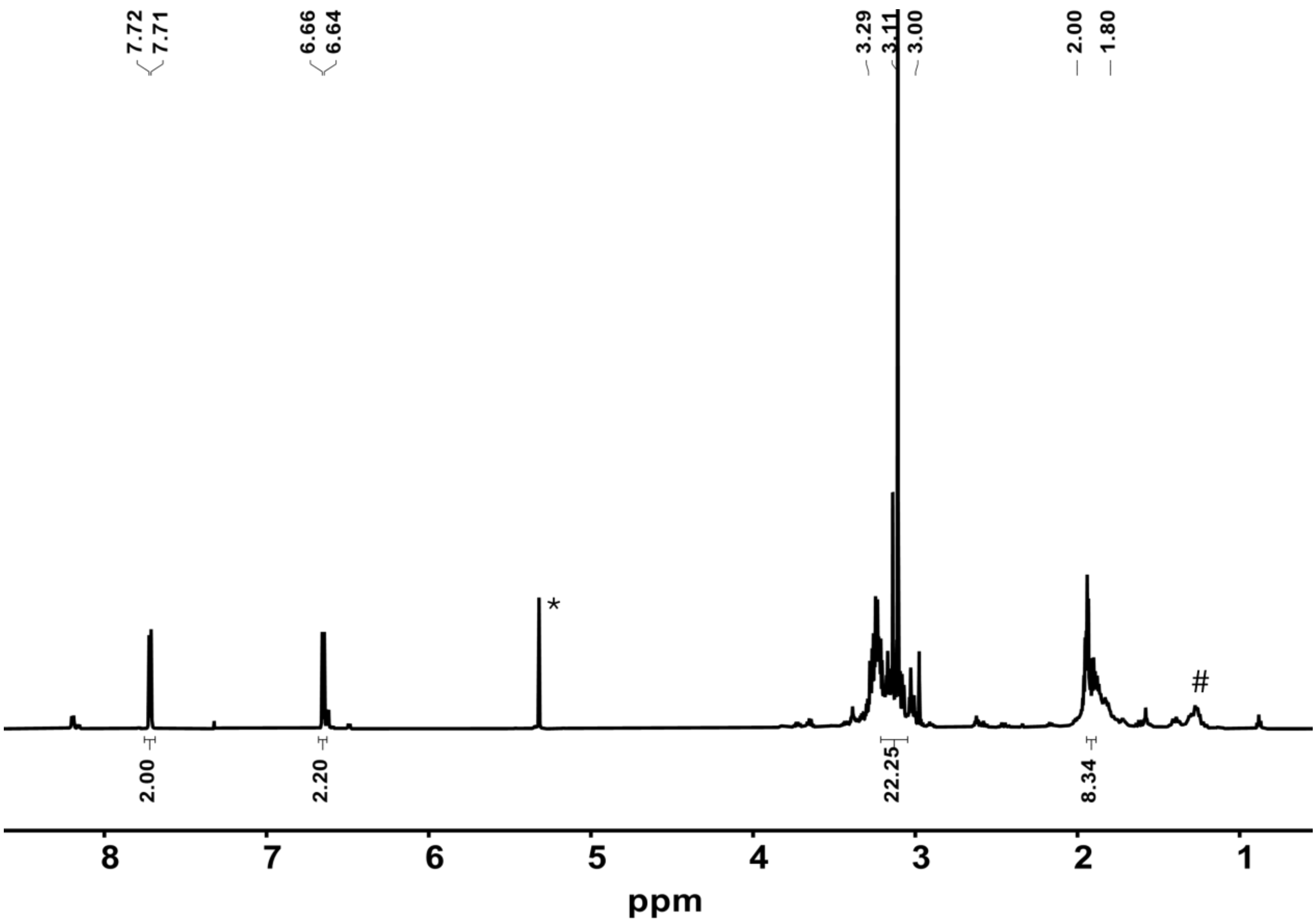


***Figure S38.*** *$^{1}$H NMR spectrum (600 MHz) of [(DMAP)(H){B(μ-hpp)}$_2$][HB($C_6F_5$)$_3$] in $CD_2Cl_2$. The asterisk * denotes a solvent signal. # denote residues of $PCy_3$.*

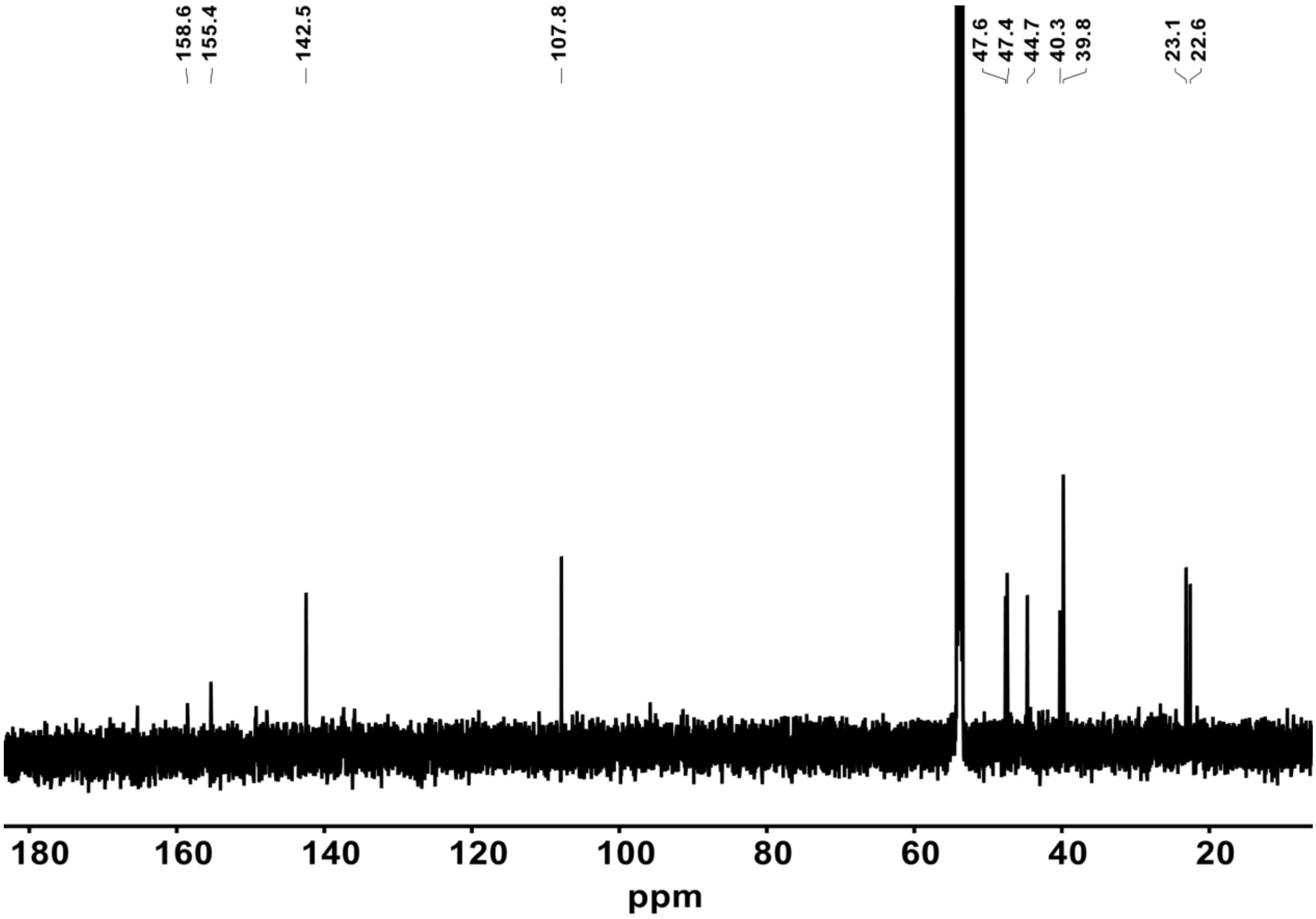


***Figure S39.*** *$^{13}$C{$^{1}$H} NMR spectrum (151 MHz) of [(DMAP)(H){B(μ-hpp)}$_2$][HB($C_6F_5$)$_3$] in $CD_2Cl_2$. The asterisk * denotes a solvent signal.*

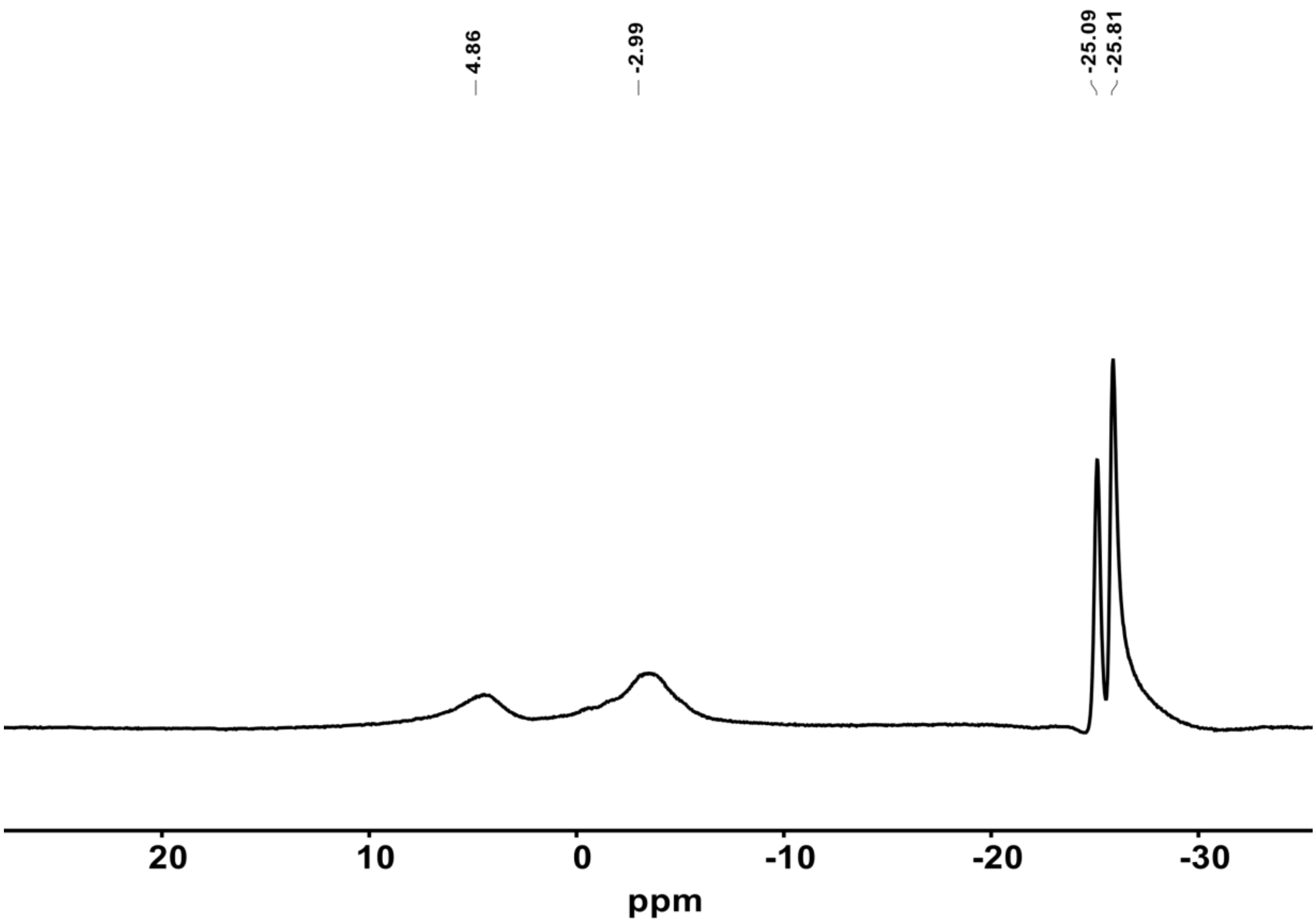


***Figure S40.*** *$^{11}B$ NMR spectrum (193 MHz) of [(DMAP)(H){B(μ-hpp)}$_2$][HB($C_6F_5$)$_3$] in $CD_2Cl_2$.*

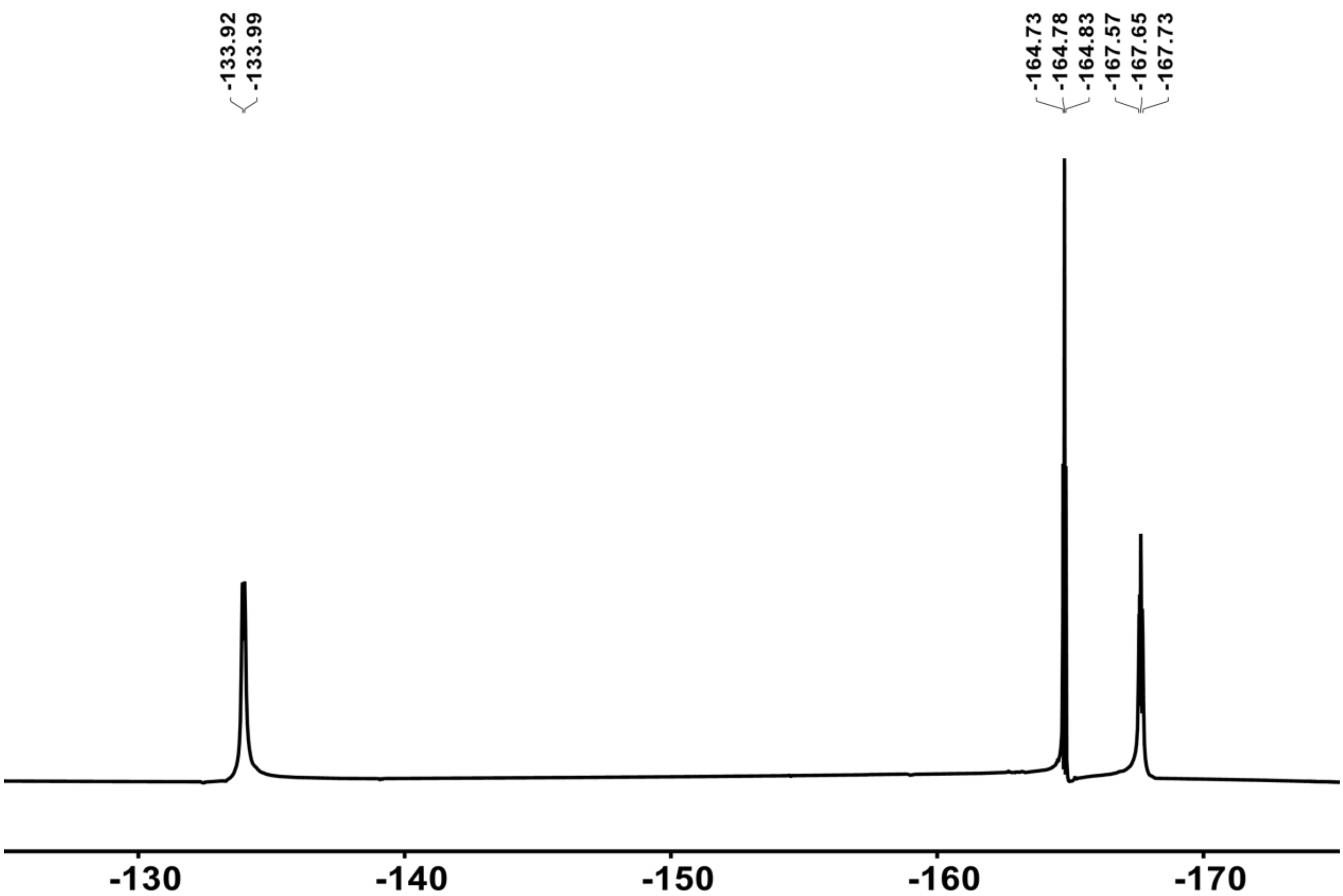


***Figure S41.*** *$^{19}F$ NMR spectrum (377 MHz) of [(DMAP)(H){B(μ-hpp)}$_2$][HB($C_6F_5$)$_3$] in $CD_2Cl_2$.*

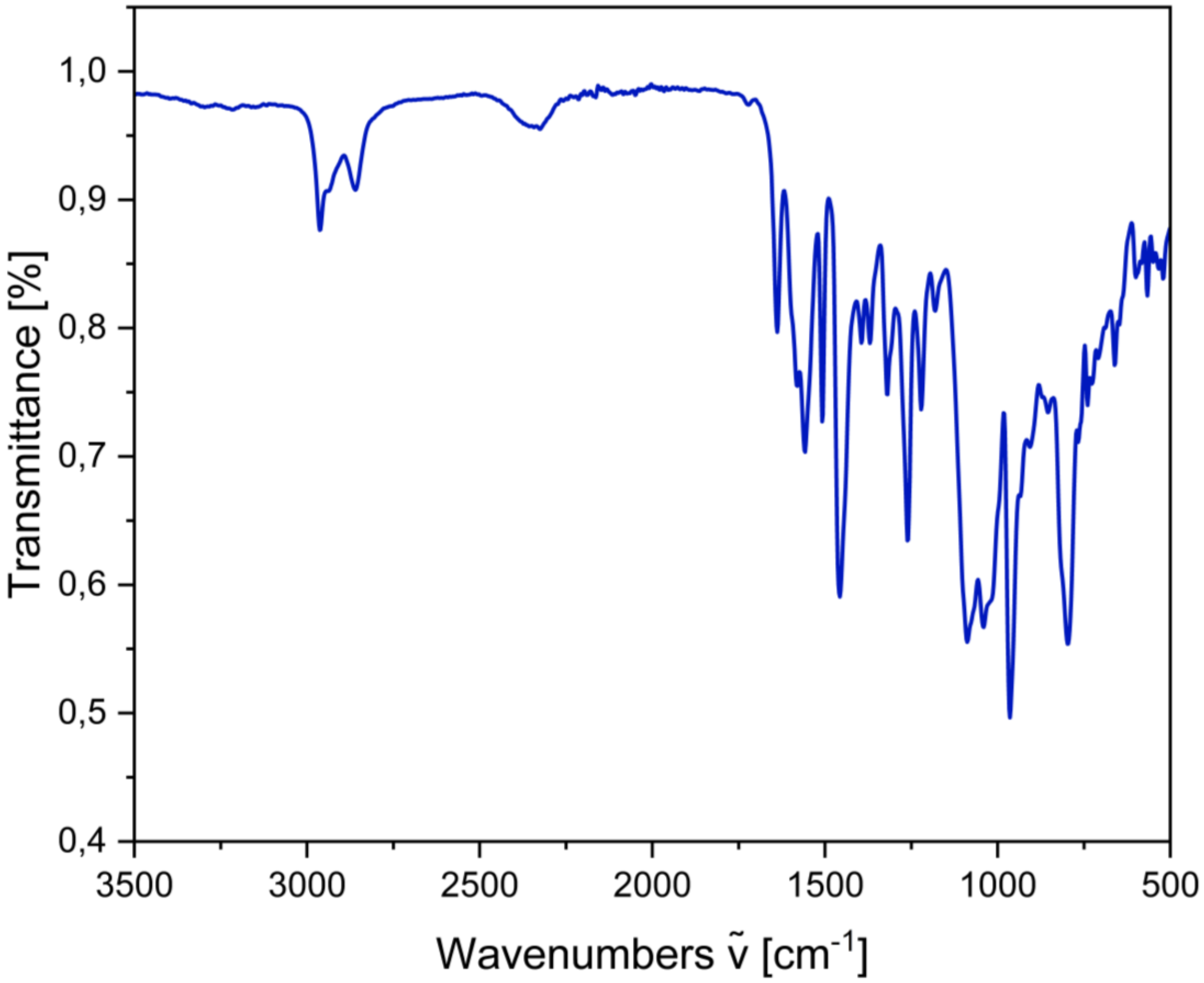


***Figure S42.*** *IR spectrum of [$HB_2$(hpp)$_2$(DMAP)][$HB(C_6F_5)_3$] as powder.*

## 2.3.2 [(Py)(H){B($\mu$-hpp)}$_2$][HB($C_6F_5$)$_3$] **DB6**[HB($C_6F_5$)$_3$]

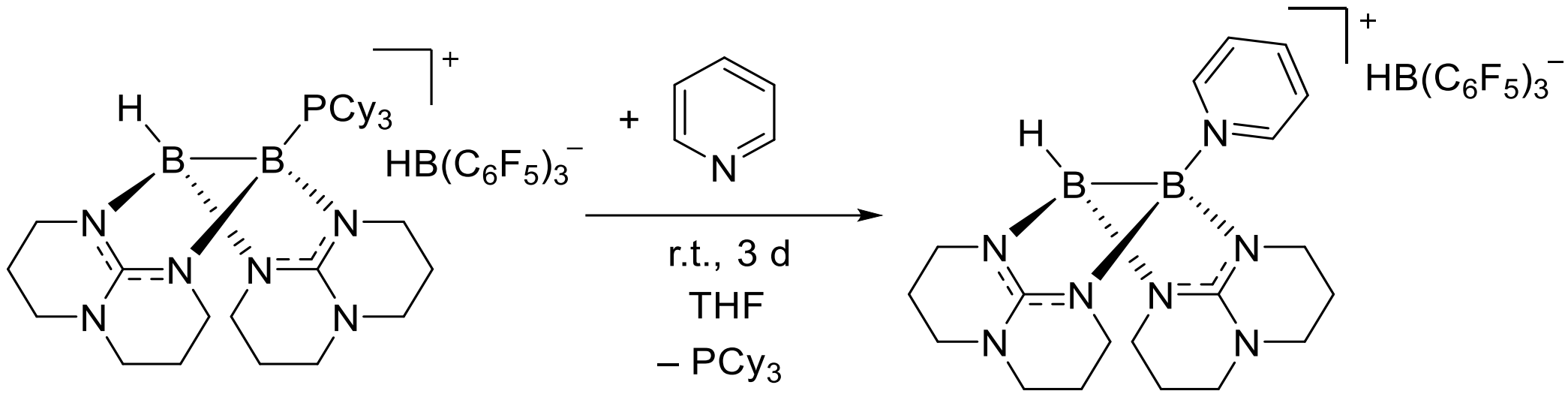


In a Schlenk flask, 103 mg of **DB2**[HB($C_6F_5$)$_3$] (94.3 µmol, 1.00 eq.) was dissolved in 6 mL tetrahydrofuran. Then 8.0 µL of pyridine (99.9 µmol, 1.06 eq.) was added dropwise to the vigorously stirred solution. After three days of stirring at room temperature the solvent was removed under reduced pressure. The resulting bright yellow residue was extracted with diethyl ether (3 x 1 mL). The combined extracts were layered with *n*-pentane and stored at –30 °C overnight during which a yellow precipitate formed. The solvent was removed *via* syringe and the crude product was washed with *n*-pentane (3 x 1 mL) and dried under vacuum to obtain **DB6**[HB($C_6F_5$)$_3$] in a yield of 48.6 mg (54.5 µmol, 59%) as a bright yellow solid.

**$^1$H NMR (600 MHz, $CD_2Cl_2$):**

$\delta$ = 8.31–8.29 (m, 2H, $H_{Py}$), 8.02 (tt, $^3J_{HH}$ = 8 Hz, $^4J_{HH}$ = 2 Hz,1H, $H_{Py}$), 7.71–7.69 (m, 2H, $H_{Py}$), 3.31–3.01 (m, 16H, $H_{hpp}$), 2.01–1.89 (m, 8H, $H_{hpp}$) ppm.

**$^{13}$C{$^1$H} NMR (151 MHz, $CD_2Cl_2$):**

$\delta$ = 158.0 ($C_{q\text{-}hpp}$), 143.4 ($C_{py}$), 140.2 ($C_{py}$), 127.2 ($C_{py}$), 47.6 ($C_{hpp}$), 47.4 ($C_{hpp}$), 45.9 ($C_{hpp}$), 45.0 ($C_{hpp}$), 23.0 ($C_{hpp}$), 22.6 ($C_{hpp}$) ppm.

**$^{11}$B NMR (193 MHz, $CD_2Cl_2$):**

$\delta$ = 5.36 (s, 1B), –2.77 (s, 1B), –25.47 (d, $^1J_{BH}$ = 91 Hz, 1B, $B_{HBCF}$) ppm.

**$^{11}$B{$^1$H} NMR (193 MHz, $CD_2Cl_2$):**

$\delta$ = 5.23 (s, 1B), –2.62 (s, 1B), –25.44 (s, 1B, $B_{HBCF}$) ppm.

**$^{19}$F NMR (377 MHz, $CD_2Cl_2$):**

$\delta$ = –134.00 (d, $^3J_{FF}$ = 34 Hz, 6F, $F_{HBCF}$), –164.83 (t, $^3J_{FF}$ = 20 Hz, 3F, $F_{HBCF}$), –167.62–167.78 (m, 6F, $F_{HBCF}$) ppm.

**Mass spectrum (HR-TOF-MALDI$^+$):**

m/z = 628.4213 ([M+$C_{17}H_{18}N_2$]$^+$, 100%).

**IR** (ATR): 2934 (w), 2860 (w), 2343 (w), 1642 (w), 1589 (m), 1561 (m), 1505 (m), 1461 (w), 1371 (w), 1319 (m), 1274 (m), 1185 (w), 1096 (m), 1046 (w), 998 (w), 962 (s), 906 (w), 842 (w), 756 (w), 725 (w), 697 (w), 659 (w), 583 (w), 567 (w), 533 (w) cm$^{-1}$.

**UV/Vis spectrum** ($CH_2Cl_2$): $\lambda(\varepsilon)$ = 341 (2497), 235 (9654) nm ($M^{-1}$ $cm^{-1}$).

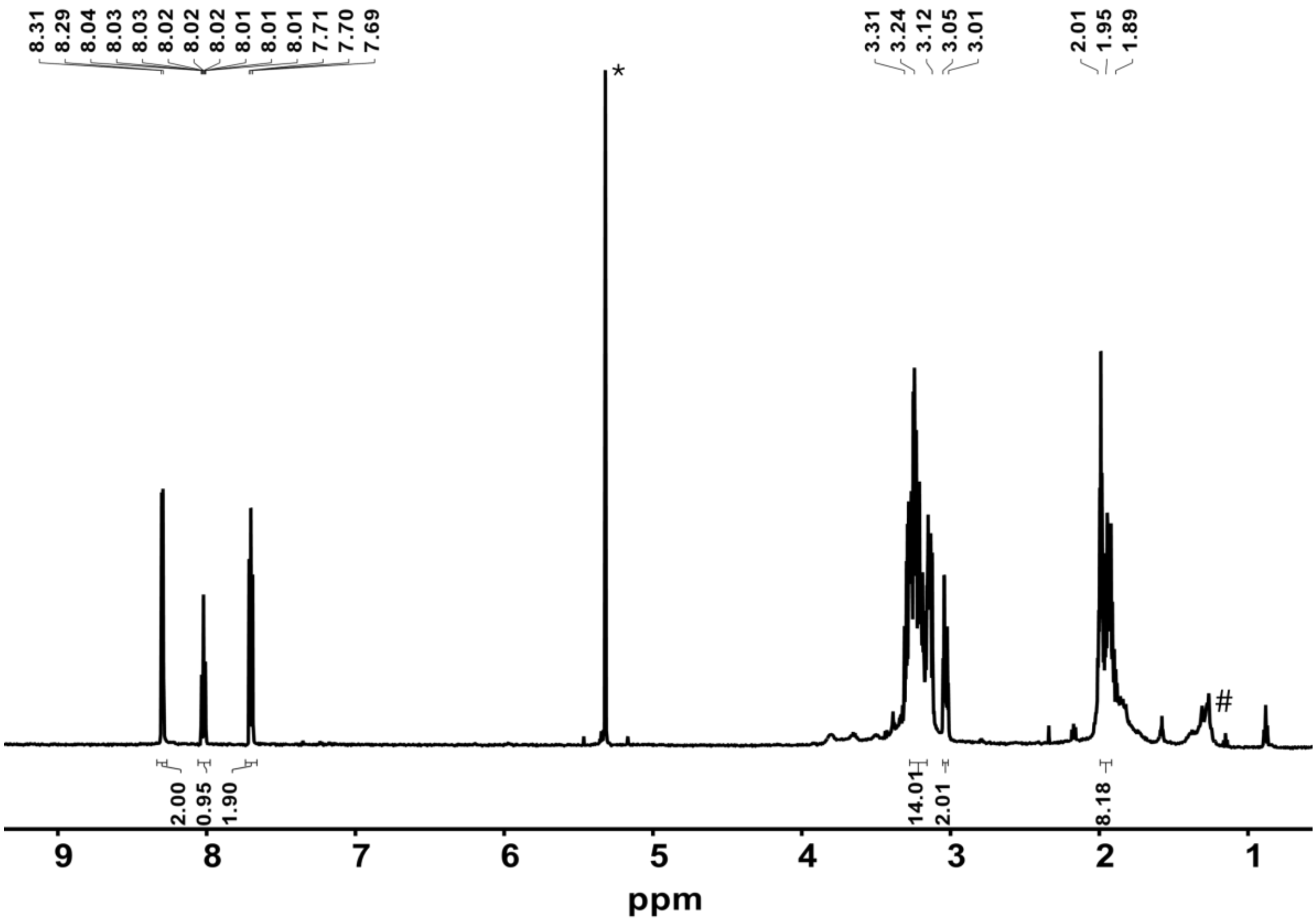


***Figure S43.*** *$^{1}$H NMR spectrum (600 MHz) of [(Py)(H){B(μ-hpp)}$_2$][HB($C_6F_5$)$_3$] in $CD_2Cl_2$. The asterisk * denotes a solvent signal. # denote residues of $PCy_3$.*

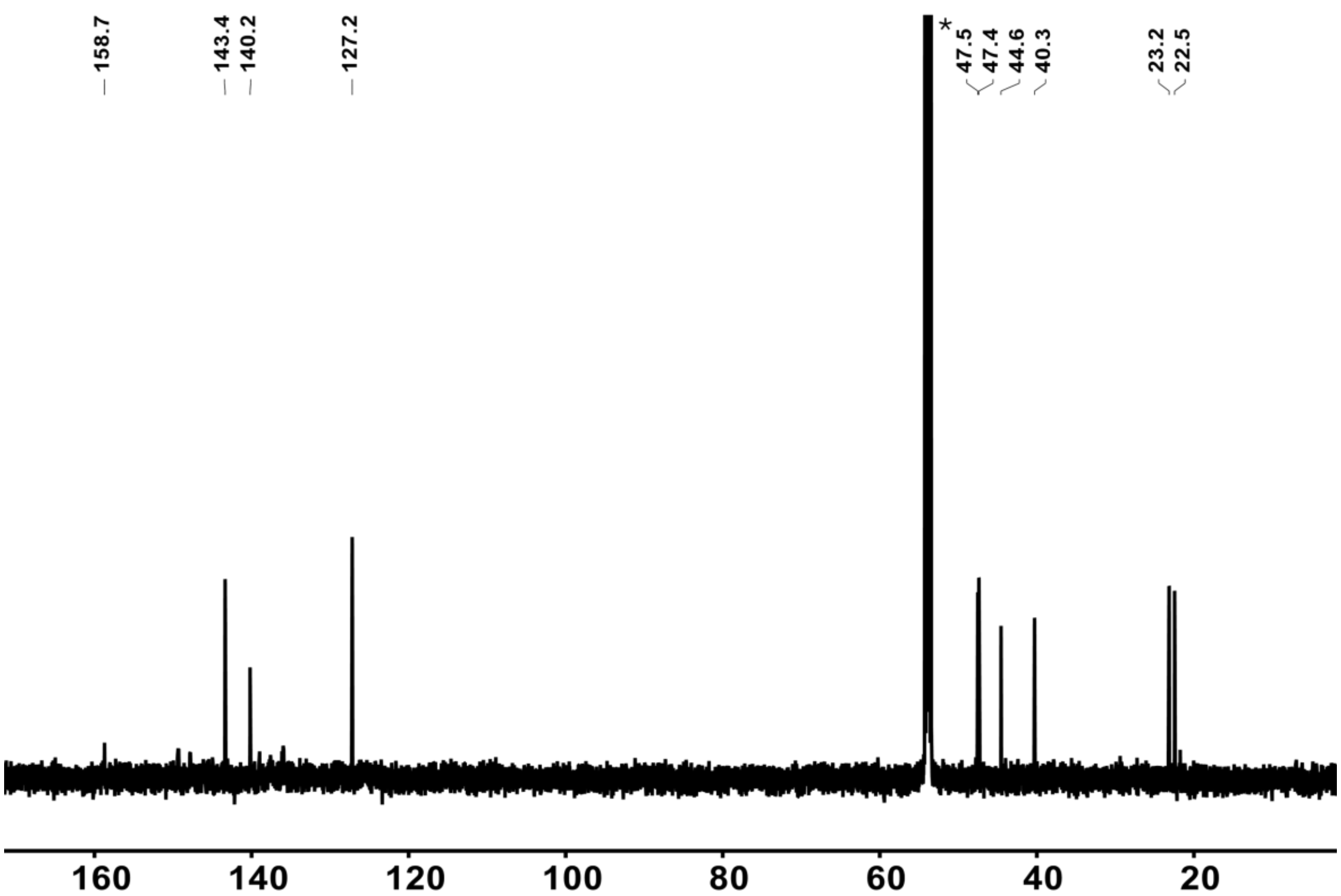


***Figure S44.*** *$^{13}$C{$^{1}$H} NMR spectrum (151 MHz) of [(Py)(H){B(μ-hpp)}$_2$][HB($C_6F_5$)$_3$] in $CD_2Cl_2$. The asterisk * denotes a solvent signal.*

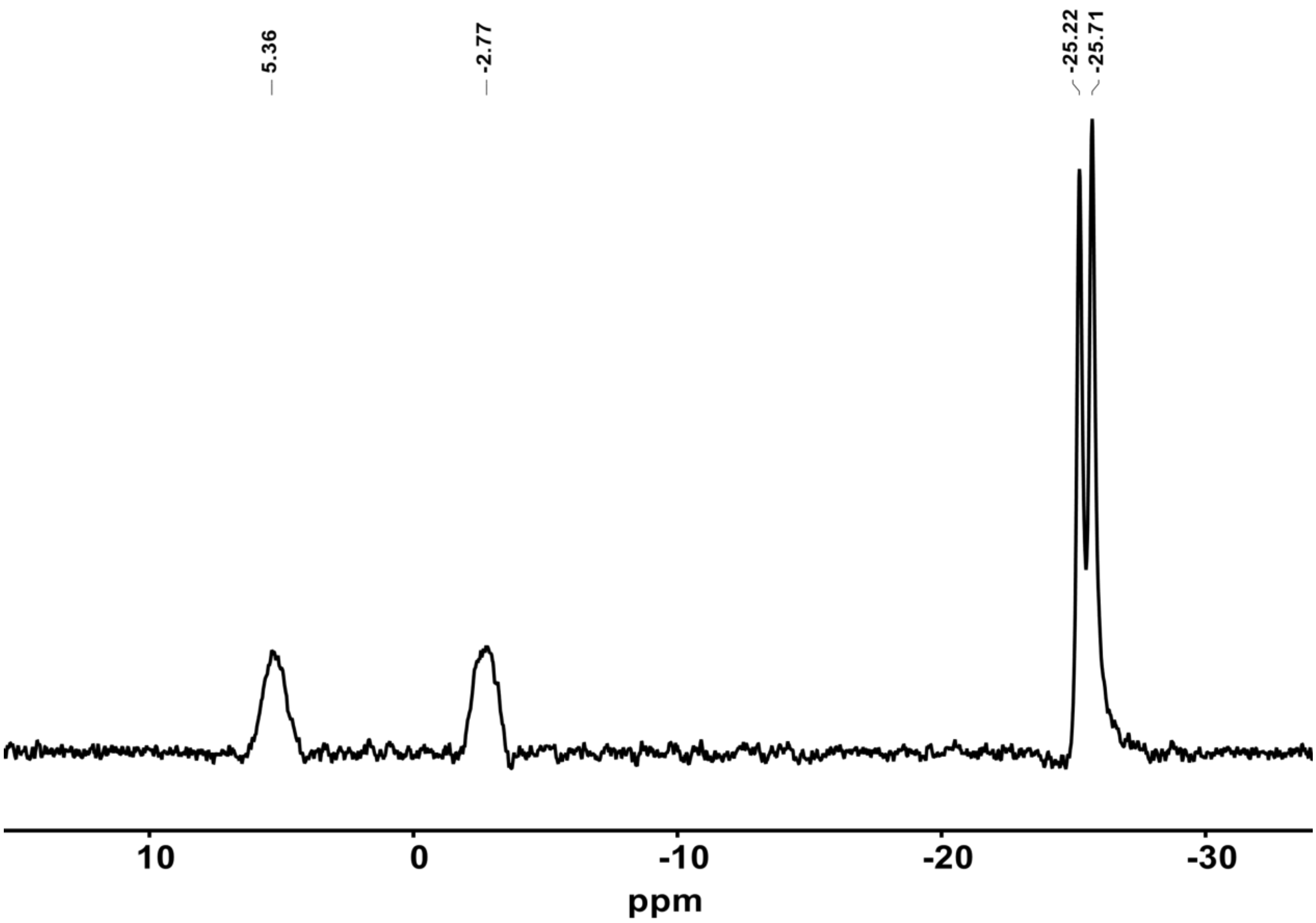


***Figure S45.*** *$^{11}$B NMR spectrum (193 MHz) of $[(Py)(H)\{B(\mu\text{-hpp})\}_2][HB(C_6F_5)_3]$ in $CD_2Cl_2$.*

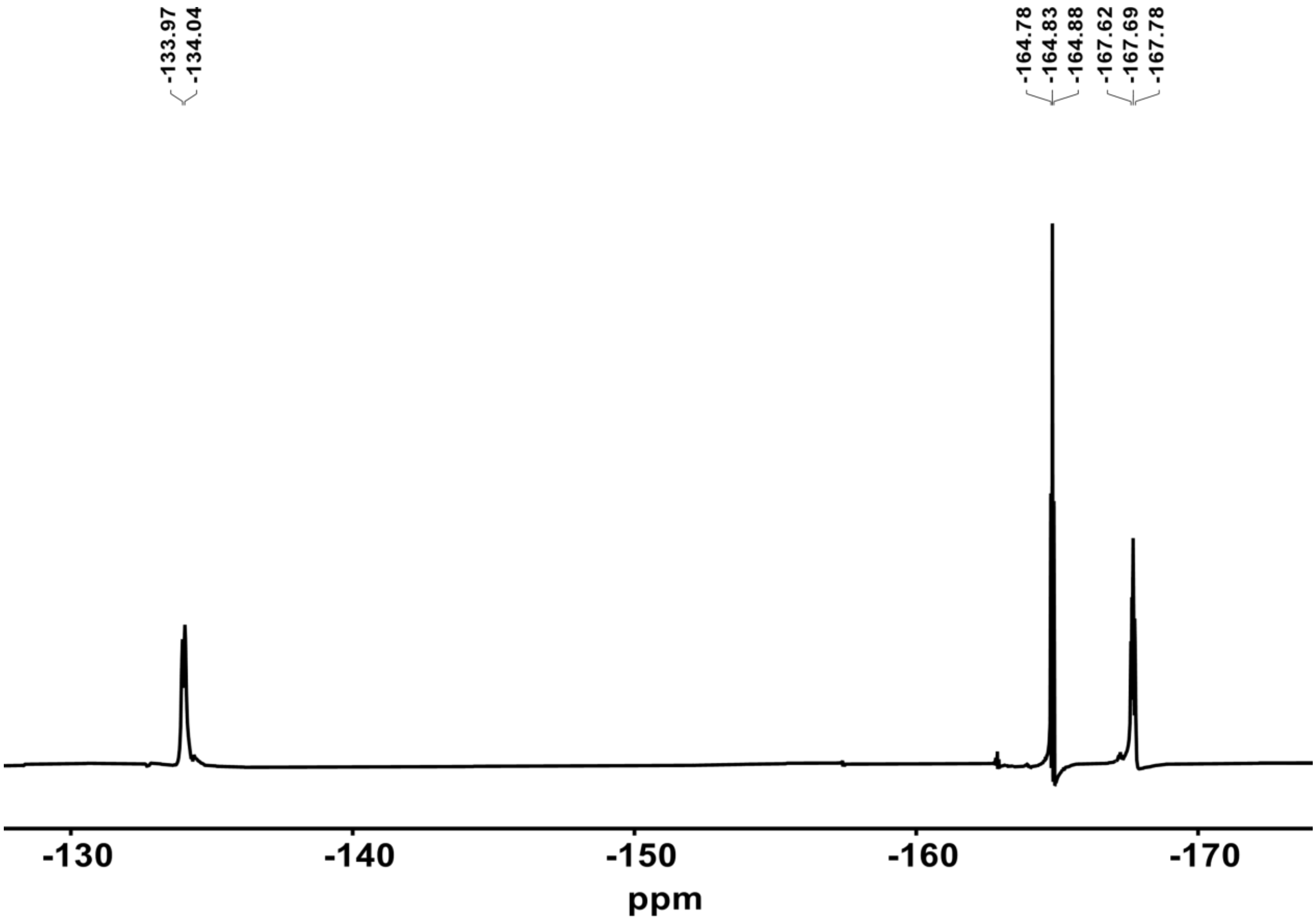


***Figure S46.*** *$^{19}$F NMR spectrum (377 MHz) of $[(Py)(H)\{B(\mu\text{-hpp})\}_2][HB(C_6F_5)_3]$ in $CD_2Cl_2$.*

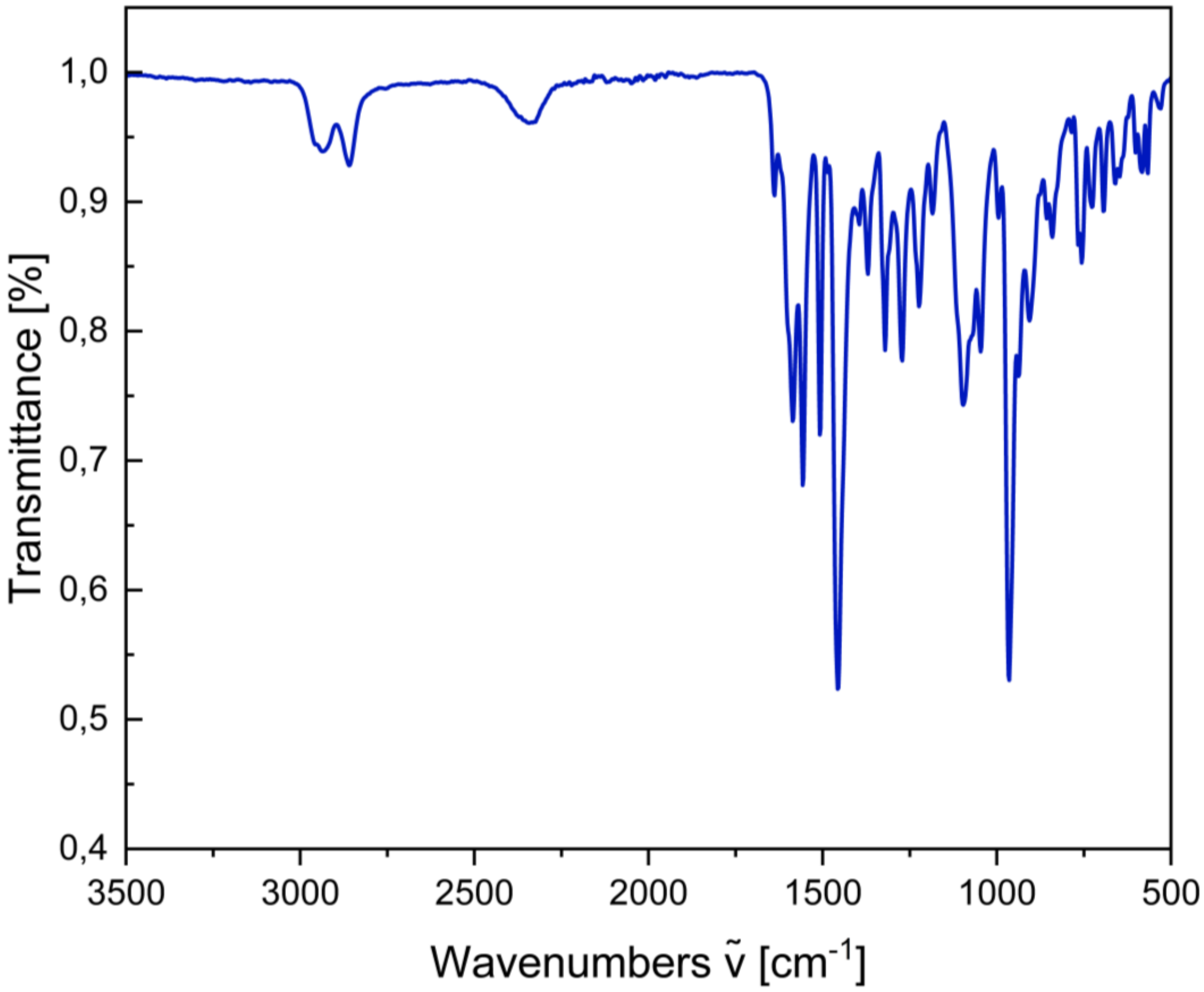


***Figure S47.*** *IR spectrum of [$HB_2$(hpp)$_2$(py)][HB($C_6F_5$)$_3$] as powder.*

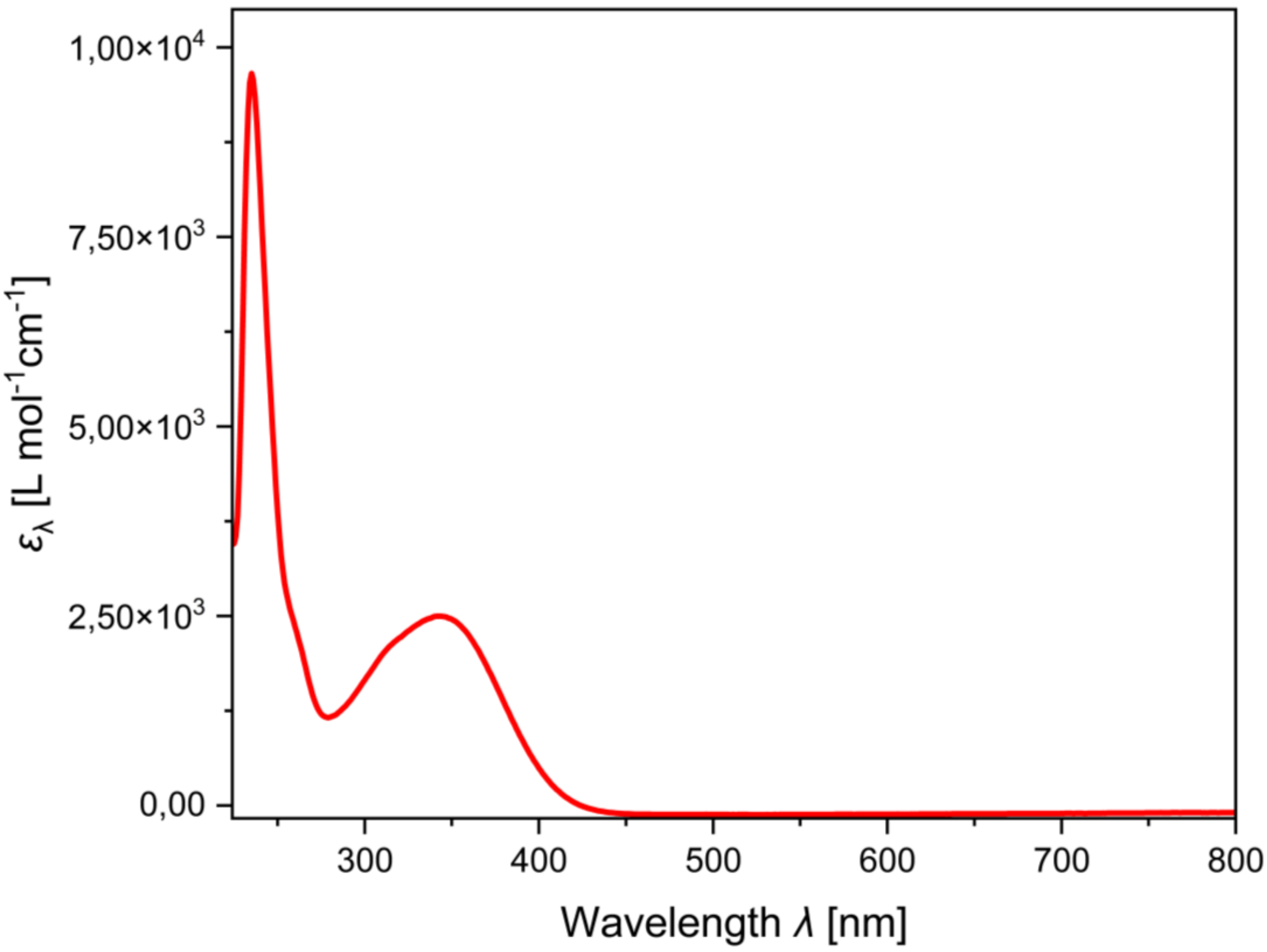


***Figure S48.*** *UV/Vis spectrum of [(Py)(H){B(μ-hpp)}$_2$][HB($C_6F_5$)$_3$] in $CH_2Cl_2$ (c = 9.49·10$^{-5}$ mol L$^{-1}$).*

## 2.3.3 [(DMAP)(H){B($\mu$-hpp)}$_2$][B(cat)$_2$] **DB5**[B(cat)$_2$]

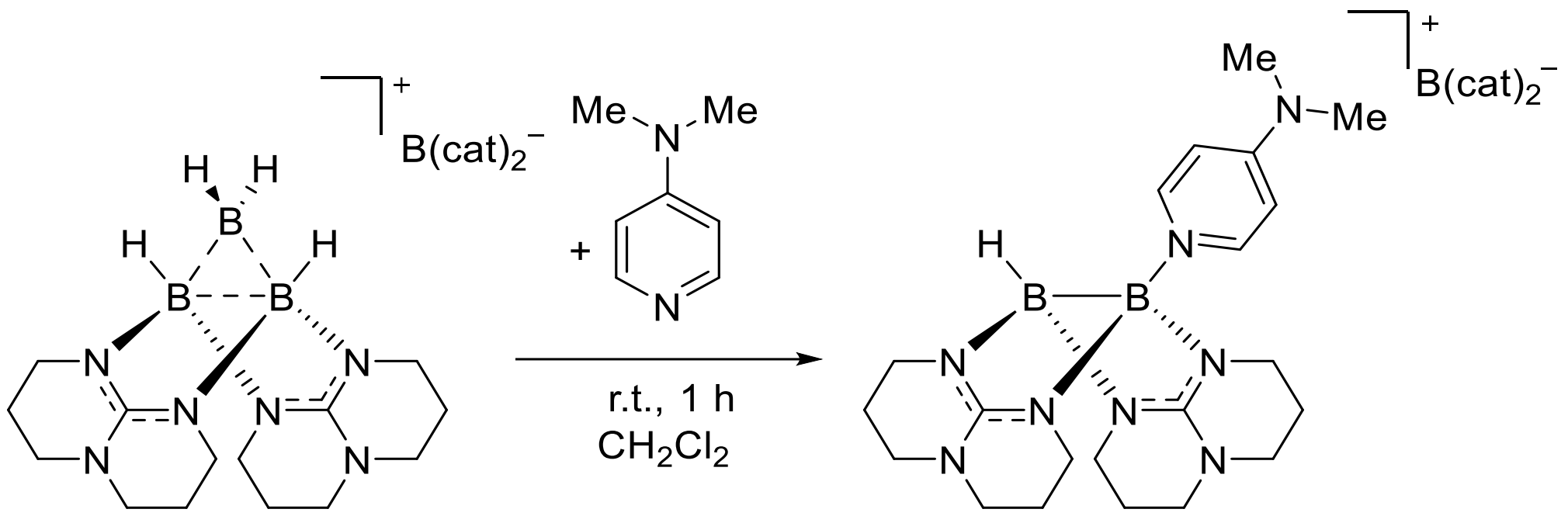


In a Schlenk flask, 30.0 mg of **TB2**[B(cat)$_2$] (55.6 µmol, 1.00 eq.) and 14.3 mg of 4-dimethylaminopyridine (116.8 µmol, 2.10 eq.) were dissolved in 2 mL dichloromethane. After one hour of stirring at room temperature the solvent was removed under reduced pressure. The colorless residue was washed with diethyl ether (24 x 2 mL) to remove 4-dimethylaminopyridine-borane formed as a byproduct and dried under vacuum to obtain compound **DB5**[B(cat)$_2$] (12.4 mg, 18.3 µmol, 33%) as a colorless solid.

**$^1$H NMR (600 MHz, $CD_2Cl_2$):**

$\delta$ = 7.72 (d, $^3J_{HH}$ = 6 Hz, 2H, $H_{DMAP}$), 6.65 (d, $^3J_{HH}$ = 6 Hz, 2H, $H_{DMAP}$), 6.58–6.52 (m, $H_{cat}$, $H_{DMAP\text{-}BH3}$), 3.30–3.00 (m, $H_{hpp}$), 3.08 (s, $H_{Me\text{-}DMAP}$), 1.97–1.85 (m, 8H, $H_{hpp}$) ppm.

**$^{13}C\{^1H\}$ NMR (151 MHz, $CD_2Cl_2$):**

$\delta$ = 158.5 ($C_{q\text{-}hpp}$), 155.4 ($C_{q\text{-}DMAP}$), 152.5 ($C_{cat\text{-}O}$), 142.5 ($C_{DMAP}$), 117.7 ($C_{cat\text{-}H}$), 108.4 ($C_{cat\text{-}H}$), 107.9 ($C_{DMAP}$), 47.6 ($C_{hpp}$), 47.4 ($C_{hpp}$), 44.7 ($C_{hpp}$), 40.3 ($C_{hpp}$), 39.8 ($C_{DMAP\text{-}Me}$), 23.1 ($C_{hpp}$), 22.6 ($C_{hpp}$) ppm.

**$^{11}B$ NMR (193 MHz, $CD_2Cl_2$):**

$\delta$ = 14.26 (s, 1B, $B_{cat}$), 4.16 (s, 1B), –3.54 (s, 1B) ppm.

**$^{11}B\{^1H\}$ NMR (193 MHz, $CD_2Cl_2$):**

$\delta$ = 14.25 (s, 1B, $B_{cat}$), 4.17 (s, 2B), –3.39 (s, 1B) ppm.

**Mass spectrum (HR-TOF-MALDI$^+$):**

m/z = 647.3623 ([M–H]$^+$, 100%).

**IR** (ATR): 2949 (w), 2853 (w), 2359 (w), 1636 (m), 1584 (m), 1561 (m), 1480 (m), 1392 (w), 1371 (w), 1305 (w), 1270 (w), 1234 (w), 1179 (m), 1091 (m), 1049 (s), 1007 (w), 913 (m), 843 (w), 830 (w), 819 (w), 796 (w), 751 (w), 736 (w), 723 (w), 701 (m), 590 (m), 549 (w), 538 (w), 523 (w) cm$^{-1}$.

**UV/Vis spectrum** ($CH_2Cl_2$): $\lambda(\varepsilon)$ = 306 (shoulder, 11898), 288 (19616), 232 (15279) nm (M$^{-1}$ cm$^{-1}$).

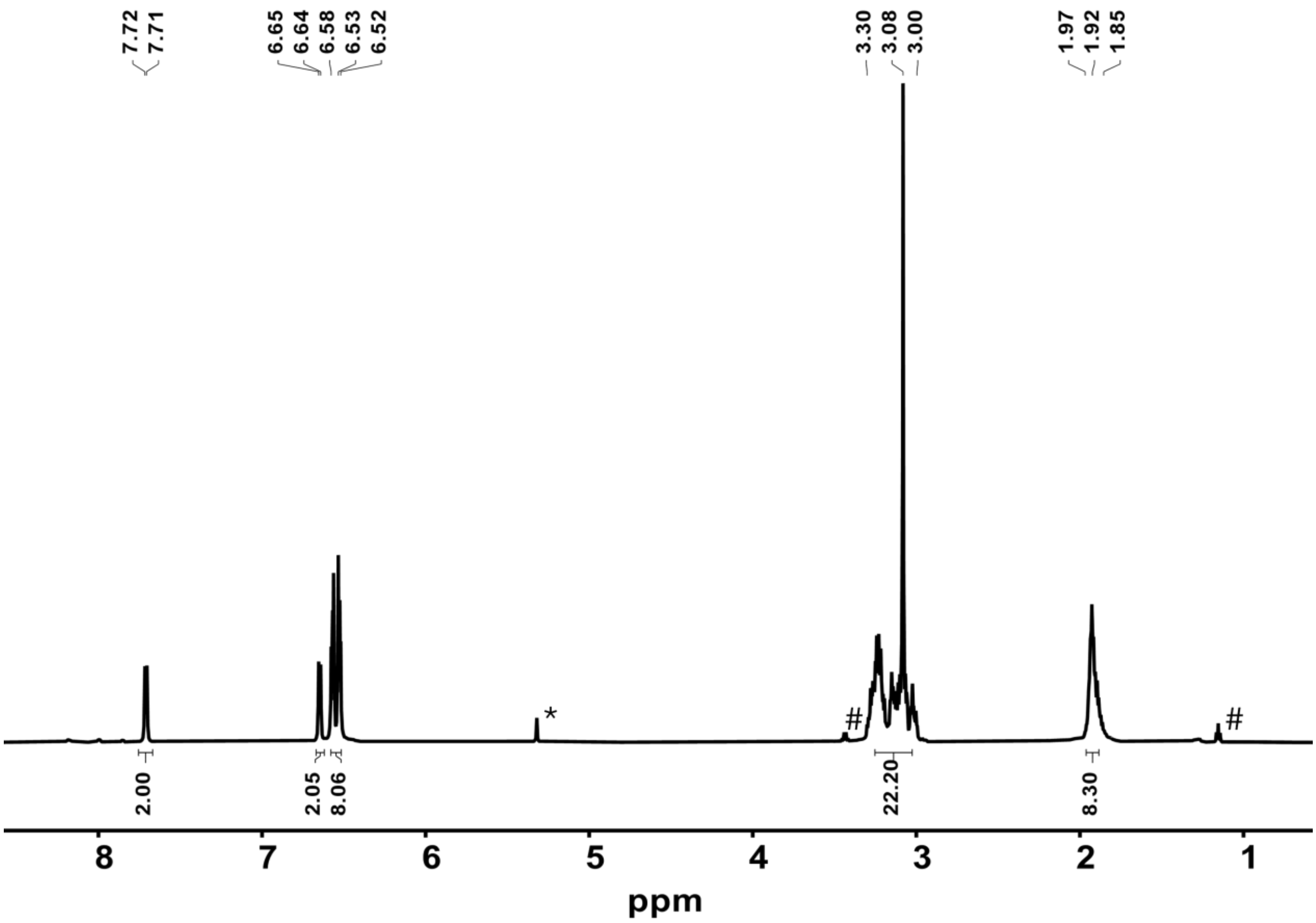


***Figure S49.*** *$^{1}H$ NMR spectrum (600 MHz) of $[HB_2(hpp)_2(DMAP)][B(cat)_2]$ in $CD_2Cl_2$. The asterisk * denotes a solvent signal. # denote traces of diethyl ether.*

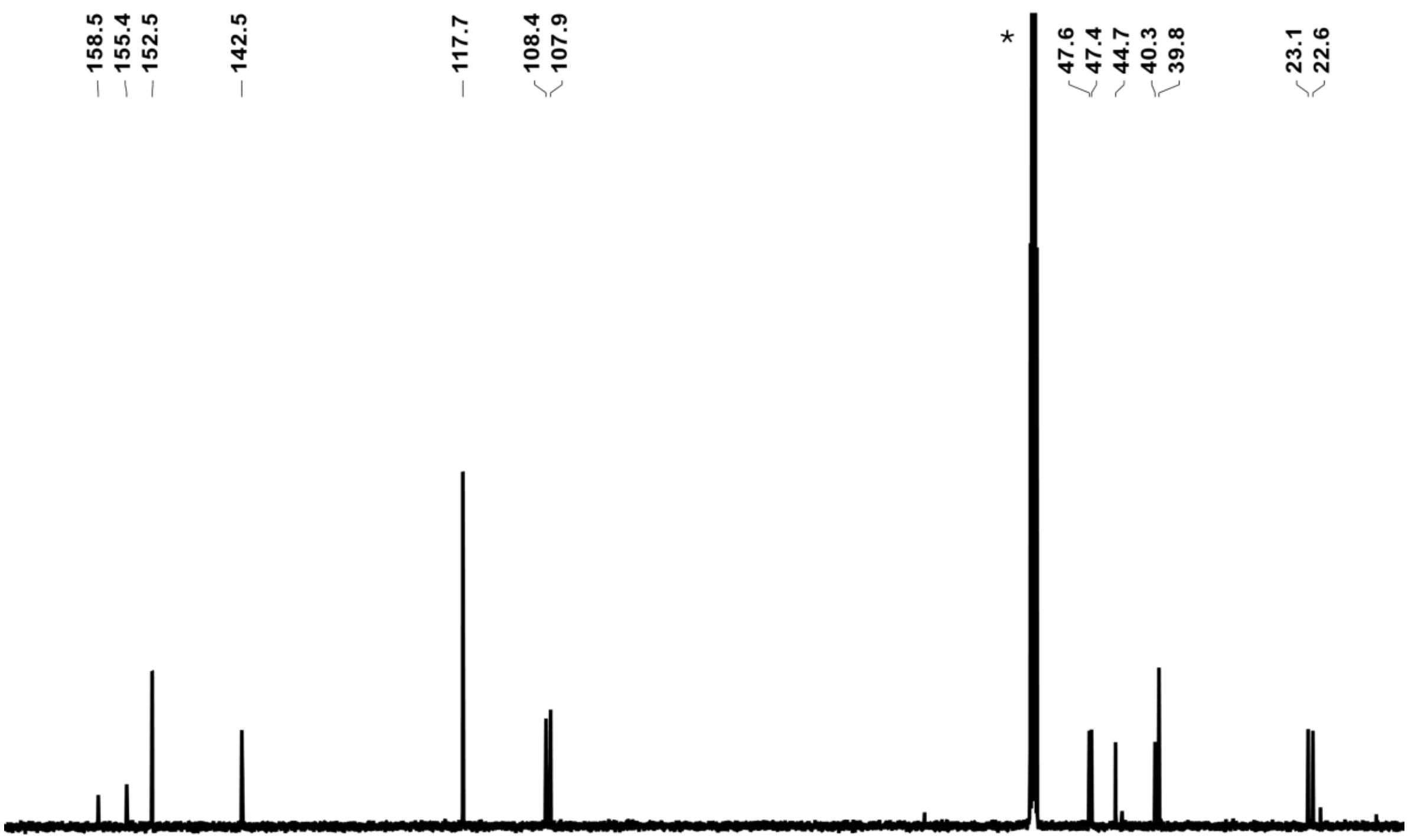


***Figure S50.*** *$^{13}C\{^{1}H\}$ NMR spectrum (151 MHz) of $[HB_2(hpp)_2(DMAP)][B(cat)_2]$ in $CD_2Cl_2$. The asterisk * denotes a solvent signal.*

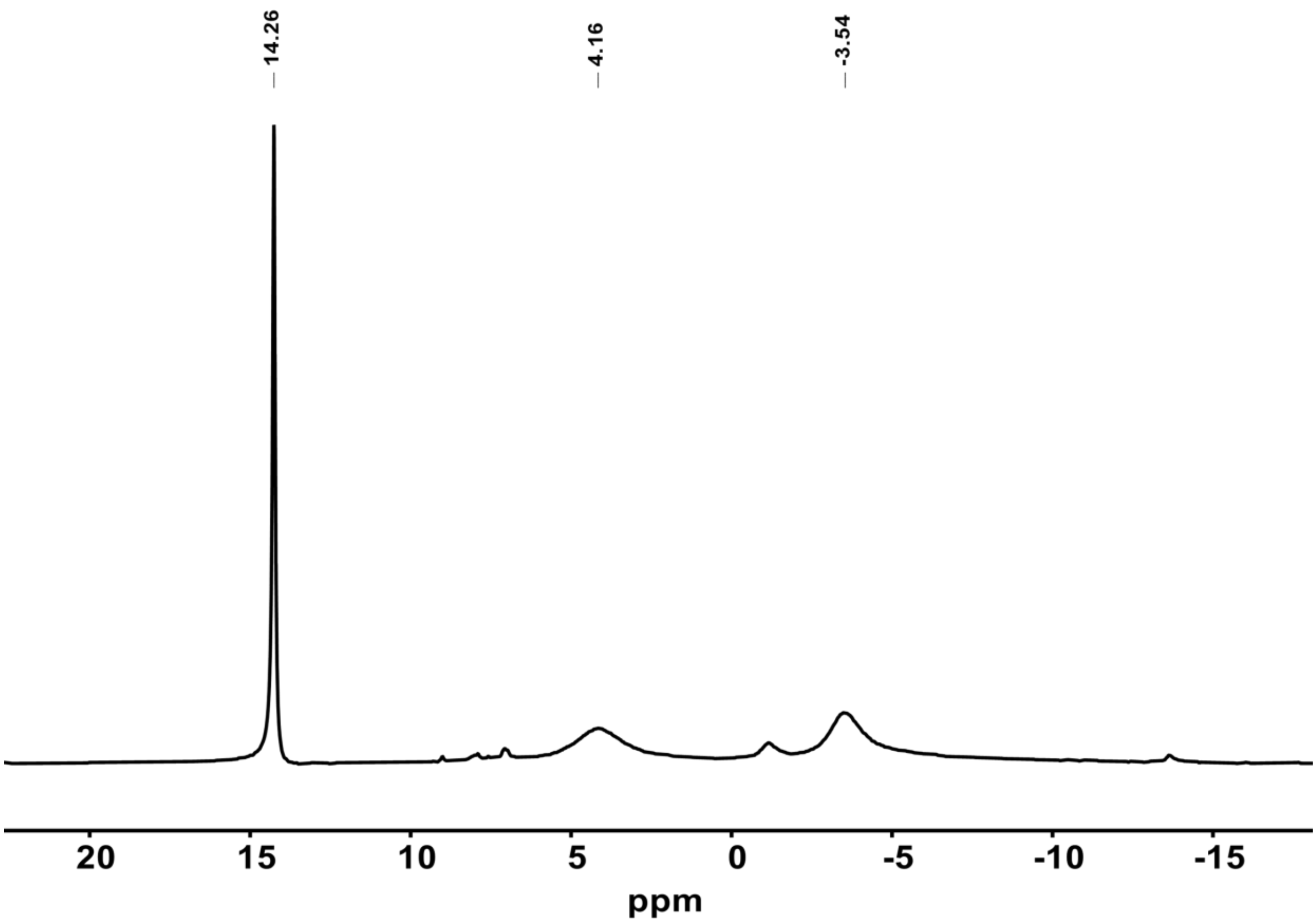


***Figure S51.*** *$^{11}B$ NMR spectrum (193 MHz) of [$HB_2(hpp)_2(DMAP)$][$B(cat)_2$] in $CD_2Cl_2$.*

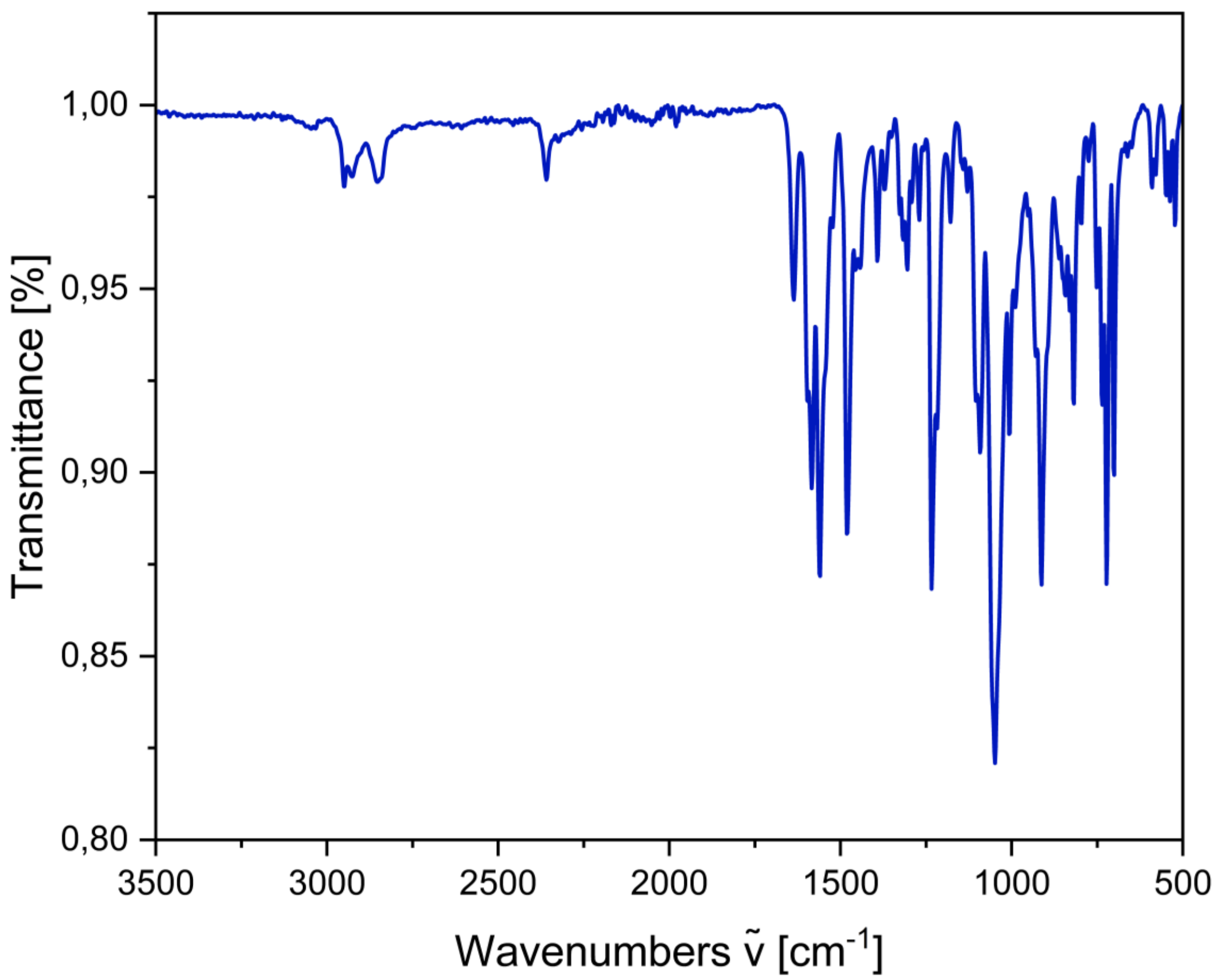


***Figure S52.*** *IR spectrum of [$HB_2(hpp)_2(DMAP)$][$B(cat)_2$] as powder.*

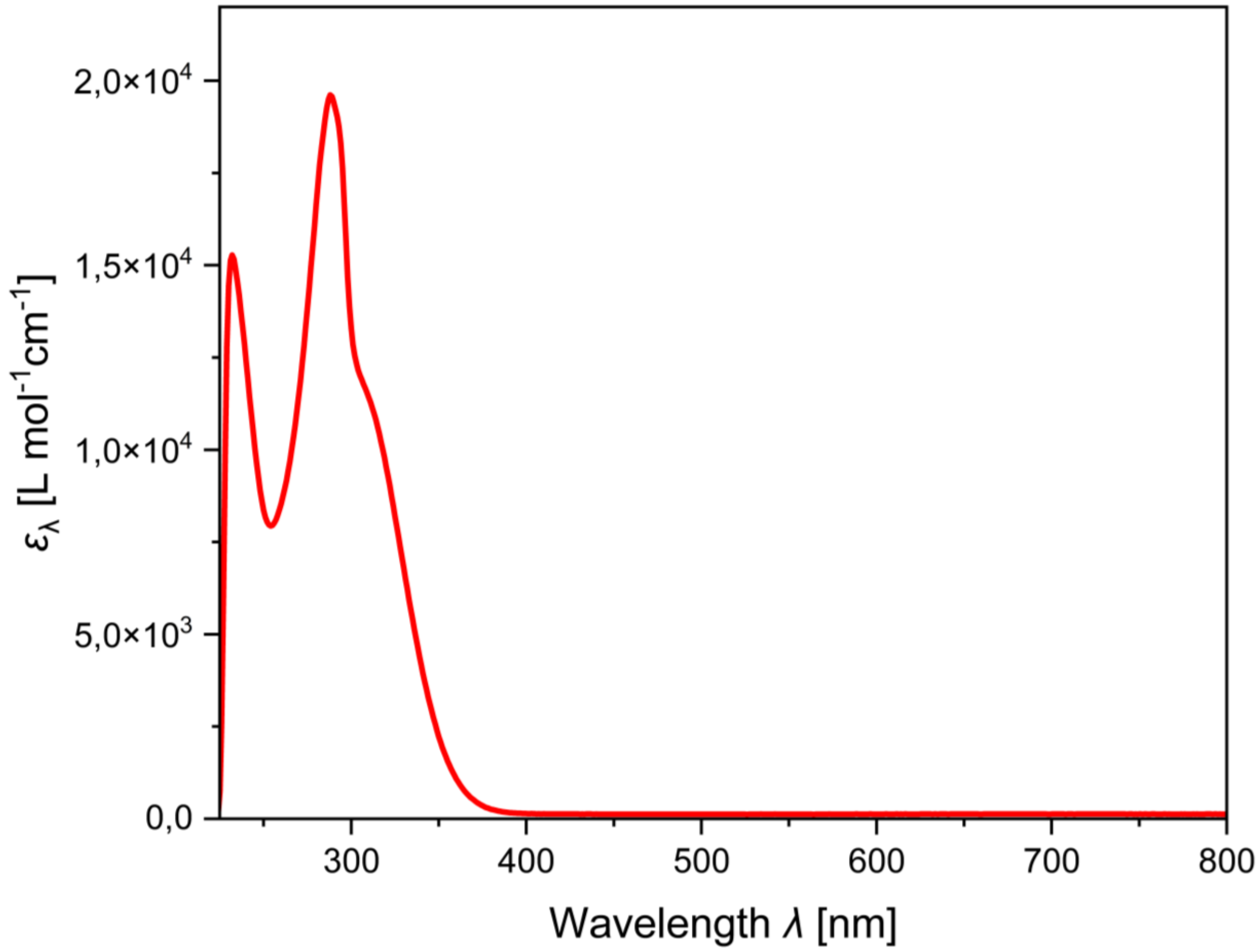


***Figure S53.*** *UV/Vis spectrum of [$HB_2$(hpp)$_2$(DMAP)][B(cat)$_2$] in $CH_2Cl_2$ (c = $4.62 \cdot 10^{-5}$ mol $L^{-1}$).*

## 2.3.4 [(Py)(H){B($\mu$-hpp)}$_2$][B(cat)$_2$] **DB6**[B(cat)$_2$]

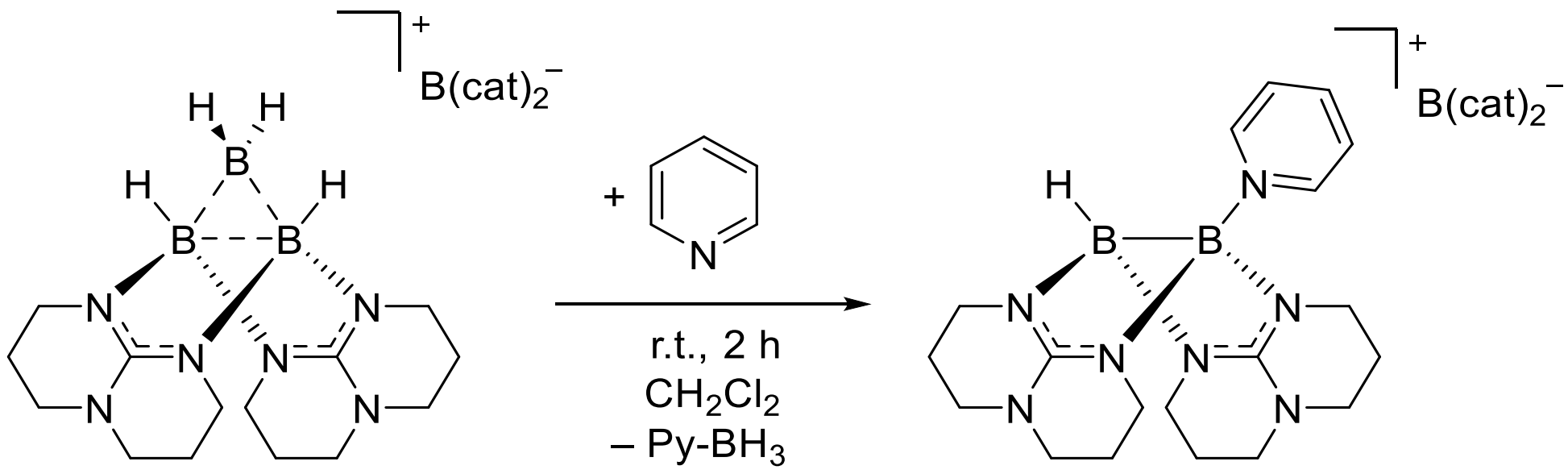


In a Schlenk flask, 30.0 mg of **TB2**[B(cat)$_2$] 55.6 µmol, 1.00 eq.) was dissolved in 3 mL dichloromethane. Then, 9.4 µL of pyridine (117 µmol, 1.10 eq.) was added dropwise to the stirred solution. After two hours of stirring at room temperature the solvent was removed under reduced pressure. The yellow residue was washed with diethyl ether (12 x 1 mL) to remove pyridine-borane formed as a byproduct and dried under vacuum to obtain compound **DB6**[B(cat)$_2$] (19.8 mg, 32.7 µmol, 59%) as a pale yellow solid.

**$^{1}$H NMR (600 MHz, CD$_2$Cl$_2$):**

$\delta$ = 8.28 (d, $^{3}J_{HH}$ = 5 Hz, 2H, $H_{Py}$), 7.98 (t, $^{3}J_{HH}$ = 8 Hz, 2H, $H_{Py}$), 7.68 (t, $^{3}J_{HH}$ = 7 Hz, 2H, $H_{Py}$), 6.58–6.51 (m, 8H, $H_{cat}$), 3.30–2.99 (m, 16H, $H_{hpp}$), 1.99–1.88 (m, 8H, $H_{hpp}$) ppm.

**$^{13}$C{$^{1}$H} NMR (151 MHz, CD$_2$Cl$_2$):**

$\delta$ = 158.7 ($C_{q\text{-}hpp}$), 152.5 ($C_{cat\text{-}O}$), 143.4 ($C_{py}$), 140.2 ($C_{py}$), 127.2 ($C_{py}$), 117.7 ($C_{cat\text{-}H}$), 108.4 ($C_{cat\text{-}H}$), 47.5 ($C_{hpp}$), 47.3 ($C_{hpp}$), 44.6 ($C_{hpp}$), 40.3 ($C_{hpp}$), 23.1 ($C_{hpp}$), 22.5 ($C_{hpp}$) ppm.

**$^{11}$B NMR (193 MHz, CD$_2$Cl$_2$):**

$\delta$ = 14.26 (s, 1B, $B_{cat}$), 5.22 (s, 1B), –2.60 (s, 1B) ppm.

**$^{11}$B{$^{1}$H} NMR (193 MHz, CD$_2$Cl$_2$):**

$\delta$ = 14.26 (s, 1B, $B_{cat}$), 5.19 (s, 1B), –2.64 (s, 1B) ppm.

**Mass spectrum (HR-TOF-MALDI$^+$):**

m/z = 604.3196 ([M–H]$^+$, 100%).

**IR** (ATR): 2960 (w), 2933 (w), 2856 (w), 2293 (w), 1585 (m), 1556 (m), 1481 (m), 1457 (w), 1395 (w), 1370 (w), 1353 (w), 1320 (w), 1275 (w), 1233 (m), 1183 (w), 1143 (w), 1094 (w), 1061 (s), 1005 (w), 936 (w), 907 (m), 857 (w), 842 (w), 795 (w), 774 (w), 730 (m), 699 (m), 579 (m), 550 (w), 536 (w) cm$^{-1}$.

**UV/Vis spectrum** (CH$_2$Cl$_2$): $\lambda(\varepsilon)$ = 341 (2079), 288 (6681), 237 (8178) nm (M$^{-1}$ cm$^{-1}$).

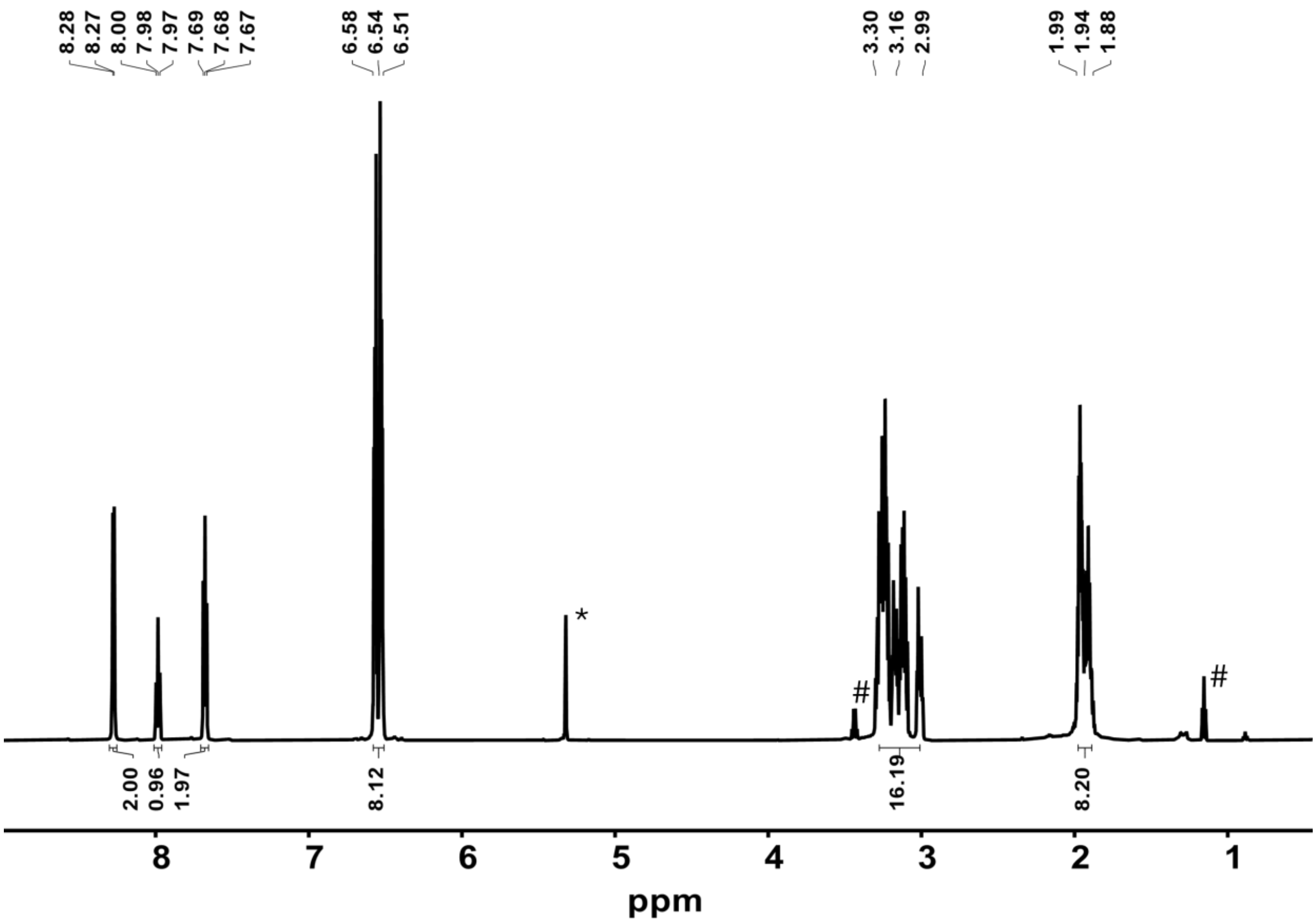


***Figure S54.*** *$^{1}$H NMR spectrum (600 MHz) of [HB₂(hpp)₂(py)][B(cat)₂] in CD₂Cl₂. The asterisk * denotes a solvent signal. # denote diethyl ether.*

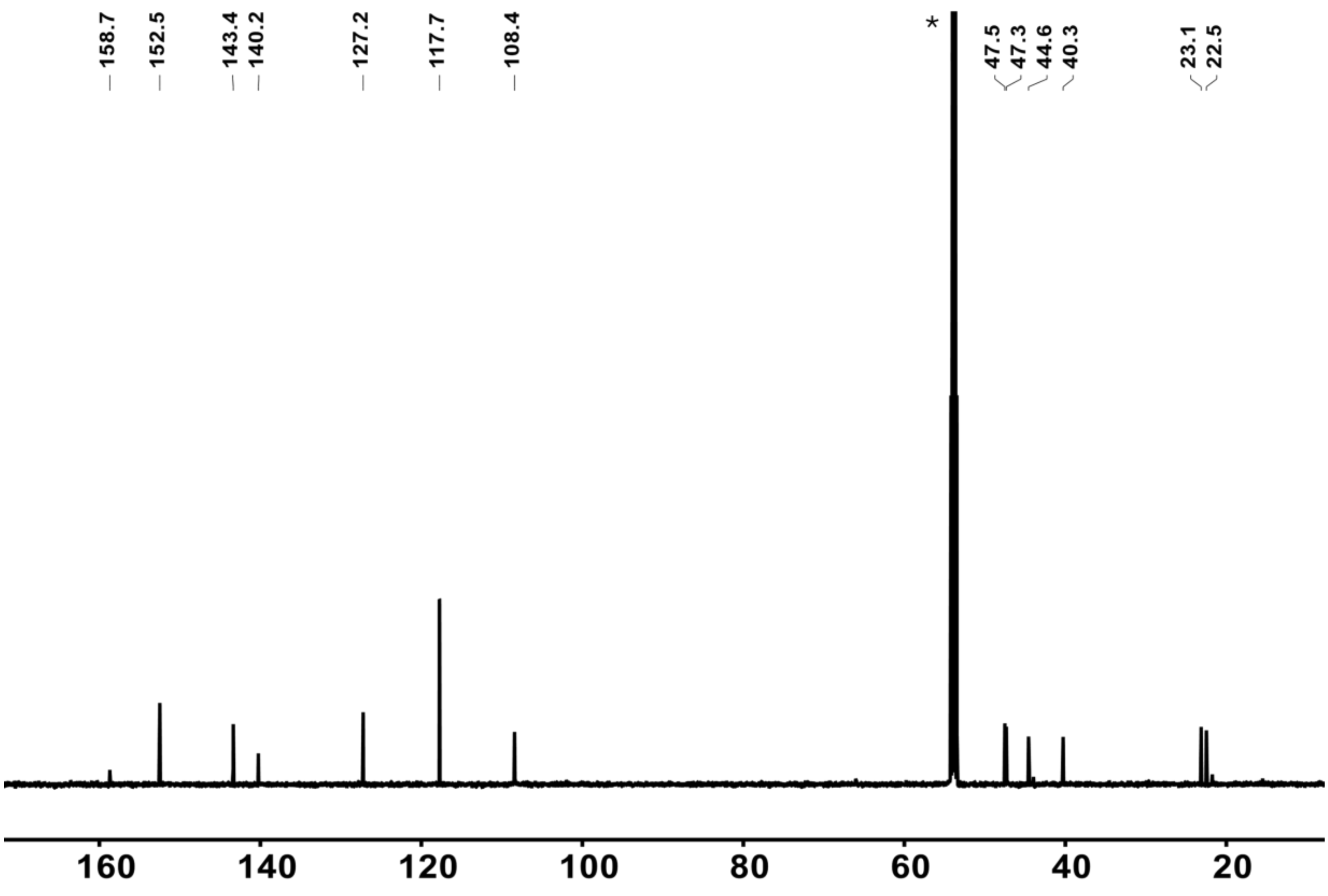


***Figure S55.*** *$^{13}C\{^{1}H\}$ NMR spectrum (151 MHz) of $[HB_2(hpp)_2(py)][B(cat)_2]$ in $CD_2Cl_2$. The asterisk * denotes a solvent signal.*

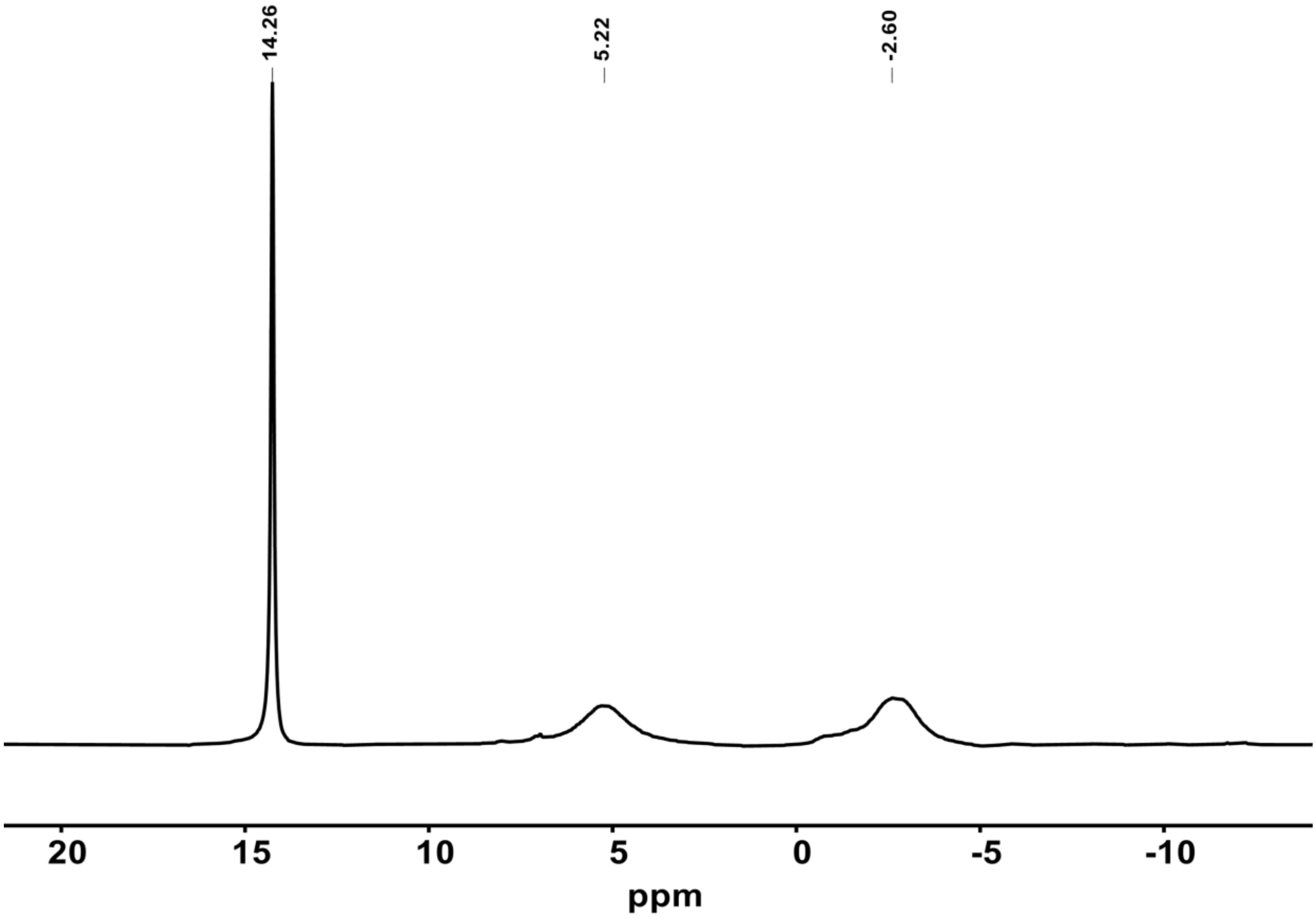


***Figure S56.*** *$^{11}$B NMR spectrum (193 MHz) of [$HB_2(hpp)_2(py)$][$B(cat)_2$] in $CD_2Cl_2$.*

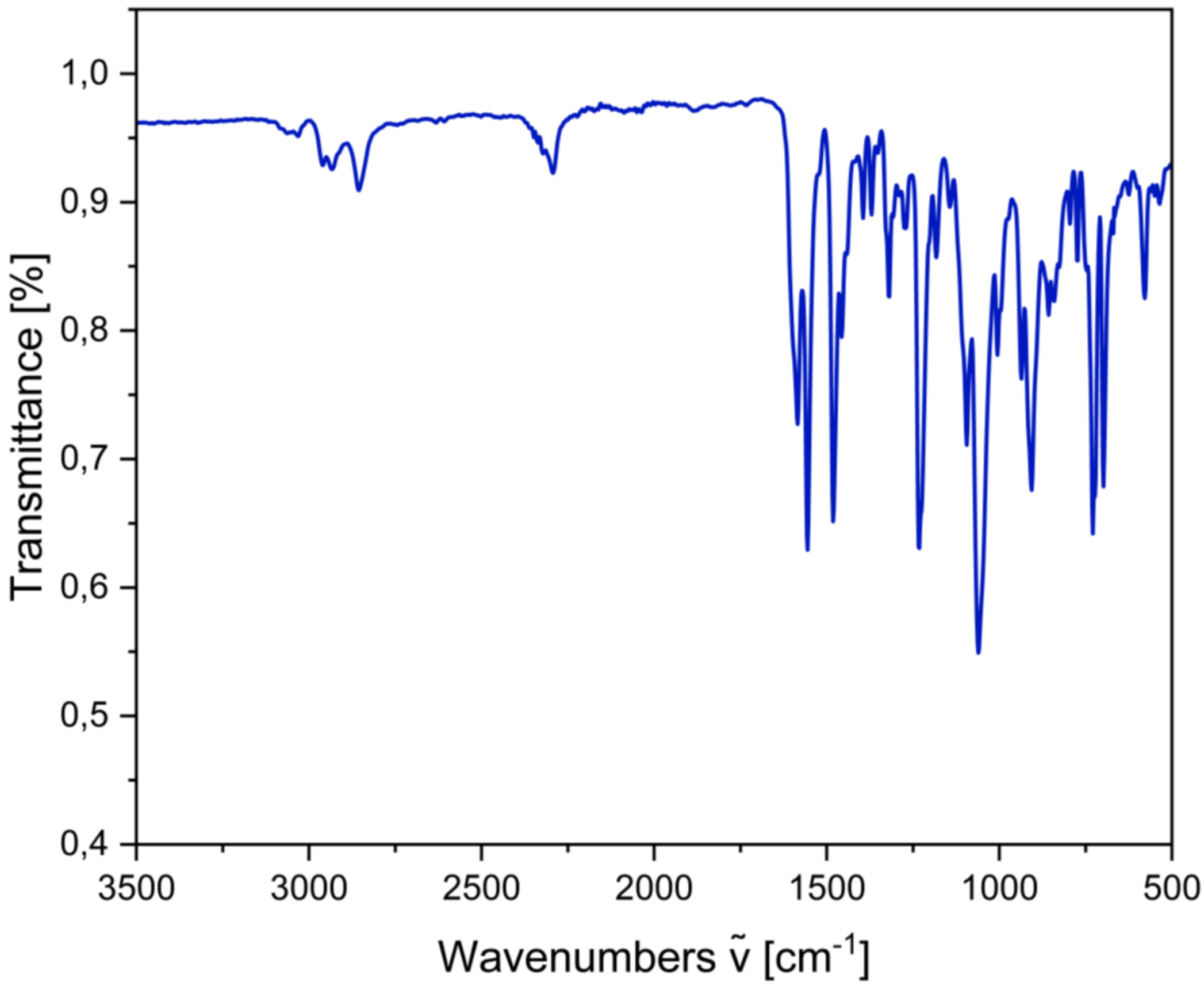


***Figure S57.*** *IR spectrum of [$HB_2(hpp)_2(py)$][$B(cat)_2$] as powder.*

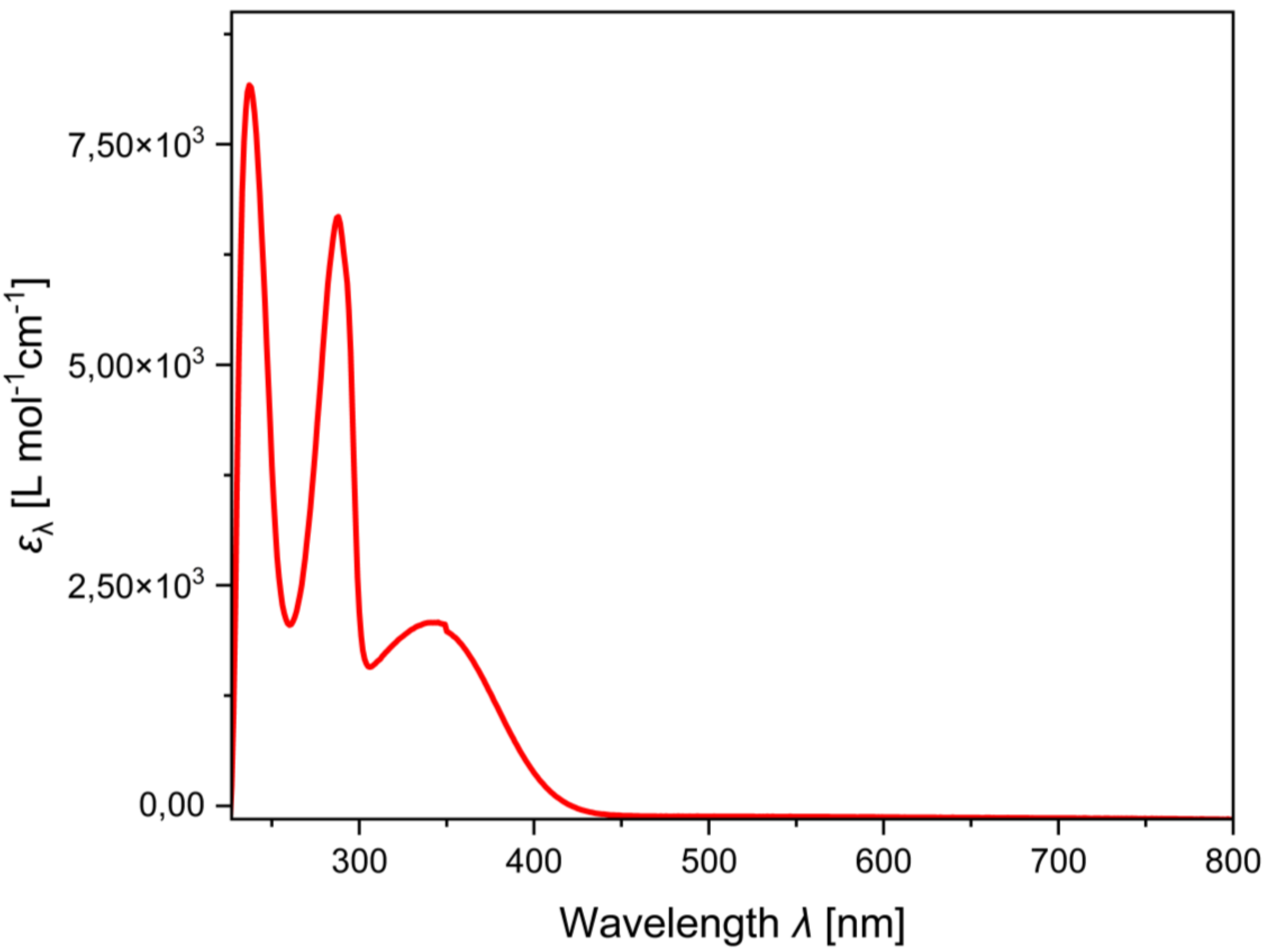


***Figure S58.*** *UV/Vis spectrum of [$HB_2$(hpp)$_2$(py)][B(cat)$_2$] in $CH_2Cl_2$ (c = 6.76·$10^{-5}$ mol $L^{-1}$).*

## 2.3.5 [($CF_3$-Py)(H){B($\mu$-hpp)}$_2$][B(cat)$_2$] **DB7**[B(cat)$_2$]

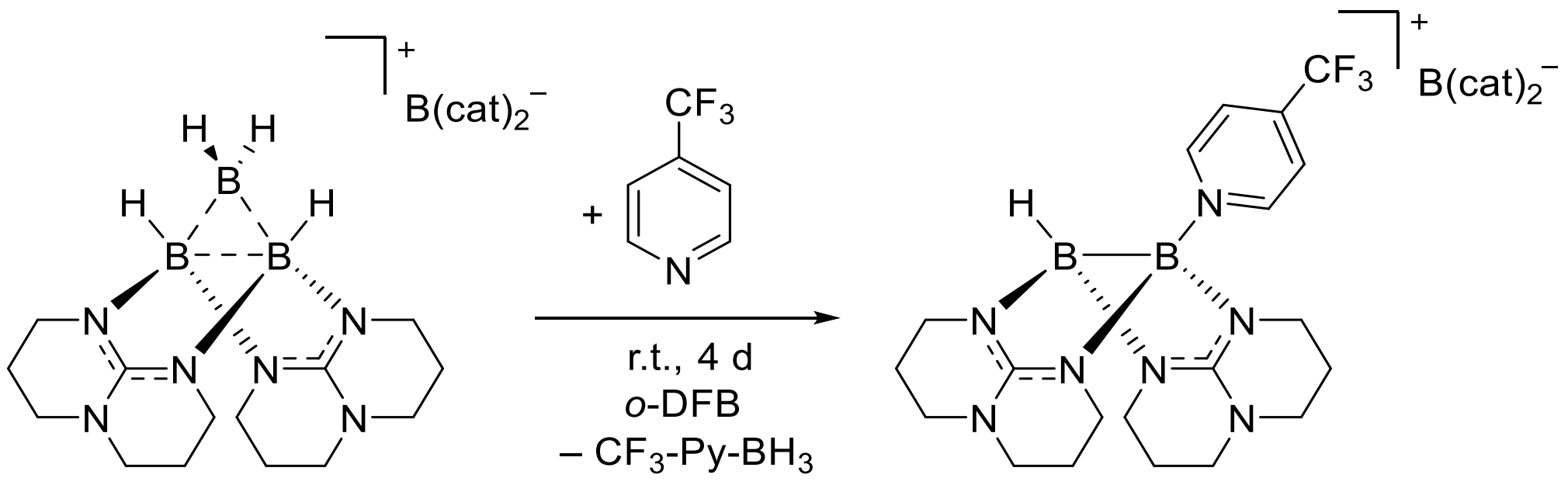

In a Schlenk flask, 30.0 mg of **TB2**[B(cat)$_2$] 55.6 µmol, 1.00 eq.) was dissolved in 3 mL *ortho*-difluorobenzene. Then, 13.0 µL of 4-trifluormethylpyridine (111 µmol, 2.00 eq.) was added dropwise to the stirred solution. After four days of stirring at room temperature the solvent was removed under reduced pressure. The orange residue was washed with diethyl ether (12 x 1 mL) to remove 4-trifluormethylpyridine-borane formed as a byproduct and dried under vacuum to obtain compound **DB7**[B(cat)$_2$] (17.4 mg, 25.9 µmol, 47%) as an orange solid.

**$^{1}$H NMR (600 MHz, $CD_2Cl_2$):**

$\delta$ = 8.52 (d, 2H, $^{3}J_{HH}$ = 8 Hz, $H_{CF3\text{-}Py}$), 7.89 (d, 2H, $^{3}J_{HH}$ = 8 Hz, 2H, $H_{CF3\text{-}Py}$), 6.57–6.51 (m, 8H, $H_{cat}$), 3.29–2.99 (m, 16H, $H_{hpp}$), 2.02–1.88 (m, 8H, $H_{hpp}$) ppm.

**$^{13}$C{$^{1}$H} NMR (151 MHz, $CD_2Cl_2$):**

$\delta$ = 158.7 ($C_{q\text{-}hpp}$), 152.4 ($C_{cat\text{-}O}$), 149.2 ($C_{CF3\text{-}Py}$), 144.8 ($C_{CF3\text{-}Py}$), 140.5 (q, $^{2}J_{CF}$ = 36 Hz, $C_{q\text{-}CF3\text{-}Py}$), 123.7 ($C_{CF3\text{-}Py}$), 122.2 (q, $^{1}J_{CF}$ = 274 Hz, $C_{CF3}$), 117.8 ($C_{cat\text{-}H}$), 108.5 ($C_{cat\text{-}H}$), 47.4 ($C_{hpp}$), 47.3 ($C_{hpp}$), 44.5 ($C_{hpp}$), 40.3 ($C_{hpp}$), 23.1 ($C_{hpp}$), 22.4 ($C_{hpp}$) ppm.

**$^{11}$B NMR (193 MHz, $CD_2Cl_2$):**

$\delta$ = 14.24 (s, 1B, $B_{cat}$), 5.62 (s, 1B), –2.09 (s, 1B) ppm.

**$^{11}$B{$^{1}$H} NMR (193 MHz, $CD_2Cl_2$):**

$\delta$ = 14.25 (s, 1B, $B_{cat}$), 5.53 (s, 1B), –2.23 (s, 1B) ppm.

**$^{19}$F NMR (377 MHz, $CD_2Cl_2$):**

$\delta$ = –65.2 (s, 1F, $F_{CF3}$) ppm.

**Mass spectrum (HR-TOF-MALDI$^{+}$):**

m/z = 448.2774 ([M+2H–B(cat)$_2$]$^{+}$, 100%), 673.3145 ([M]$^{+}$, 100%).

**IR** (ATR): 2934 (w), 2955 (w), 2323 (w),1586 (m), 1554 (m), 1481 (m), 1371 (w), 1321 (m), 1275 (w), 1231 (m), 1180 (w), 1142 (w), 1093 (m), 1048 (s), 1005 (w), 937 (w), 906 (m), 864 (w), 796 (w), 728 (m), 678 (m), 584 (w), 501 (w) cm$^{-1}$.

**UV/Vis spectrum** ($CH_2Cl_2$): $\lambda$(ε) = 384 (2963), 287 (11314), 236 (14237) nm (M$^{-1}$ cm$^{-1}$).

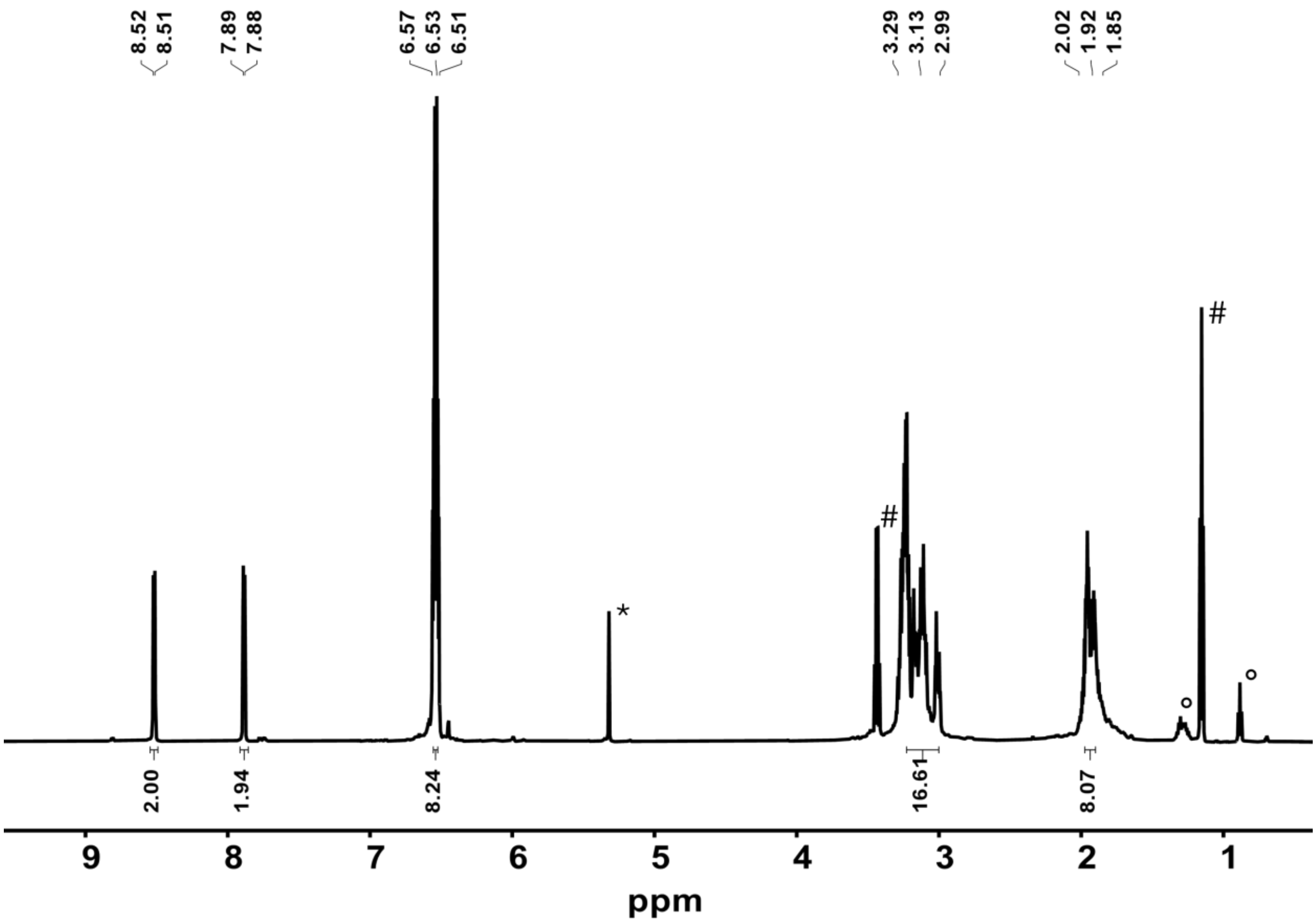


***Figure S59.*** *$^1H$ NMR spectrum (600 MHz) of $[HB_2(hpp)_2(CF_3\text{-}Py)][B(cat)_2]$ in $CD_2Cl_2$. The asterisk * denotes a solvent signal. # denote traces of diethyl ether.° denote n-pentane.*

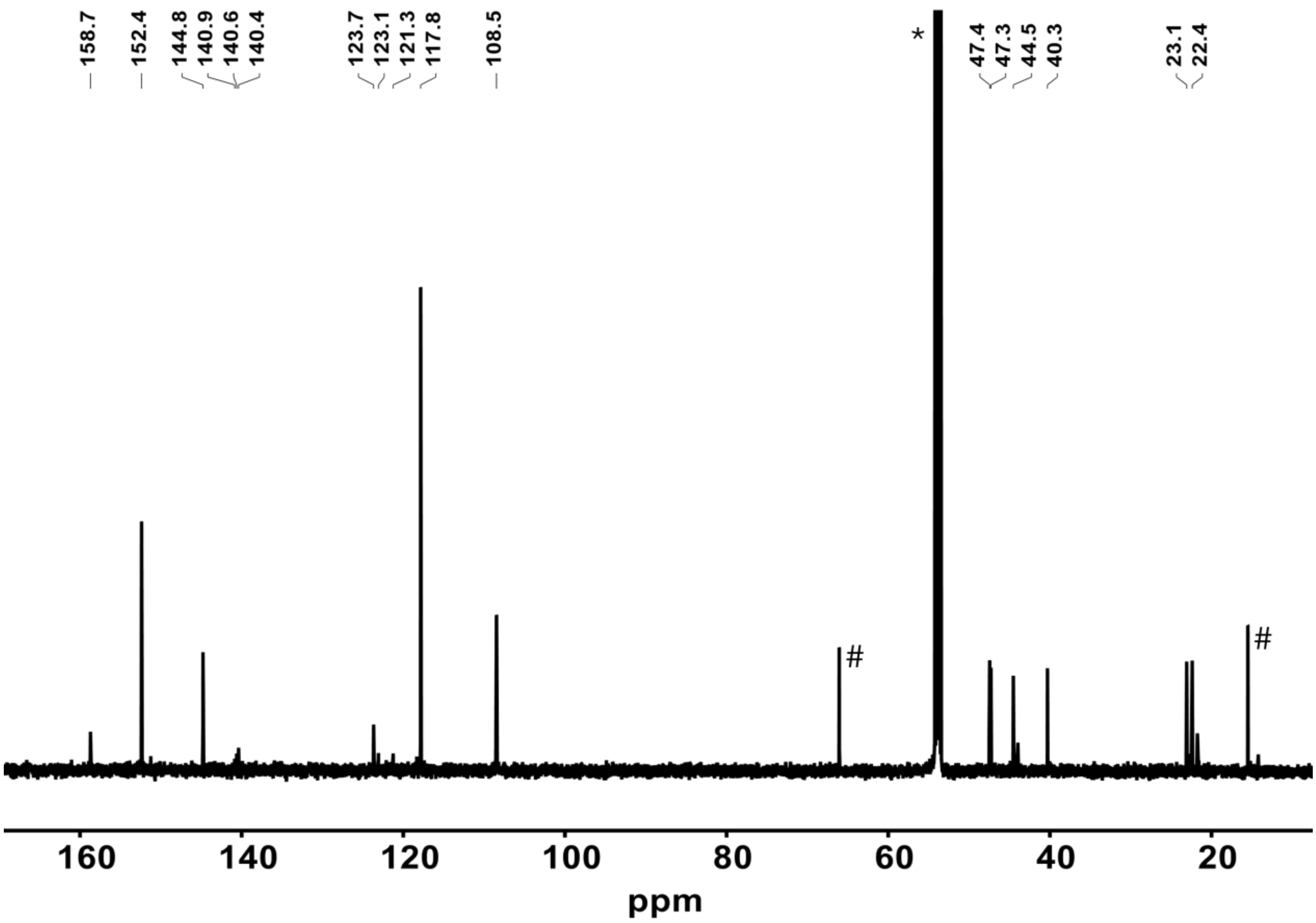


***Figure S60.*** *$^{13}C\{^1H\}$ NMR spectrum (150 MHz) of $[HB_2(hpp)_2(CF_3\text{-}Py)][B(cat)_2]$ in $CD_2Cl_2$. The asterisk * denotes a solvent signal. # denote diethyl ether.*

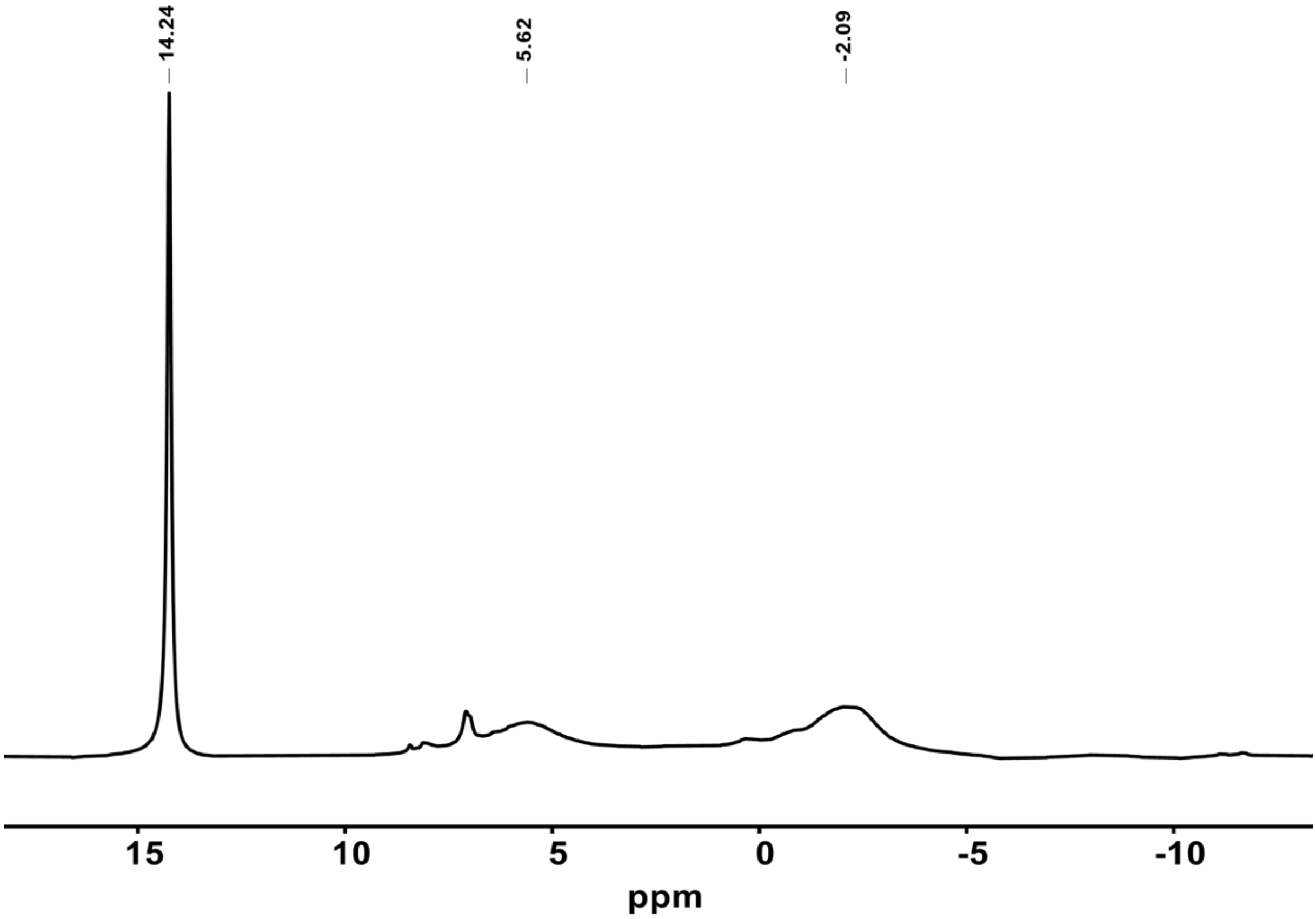


***Figure S61.*** *$^{11}B$ NMR spectrum (193 MHz) of $[HB_2(hpp)_2(CF_3\text{-}Py)][B(cat)_2]$ in $CD_2Cl_2$.*

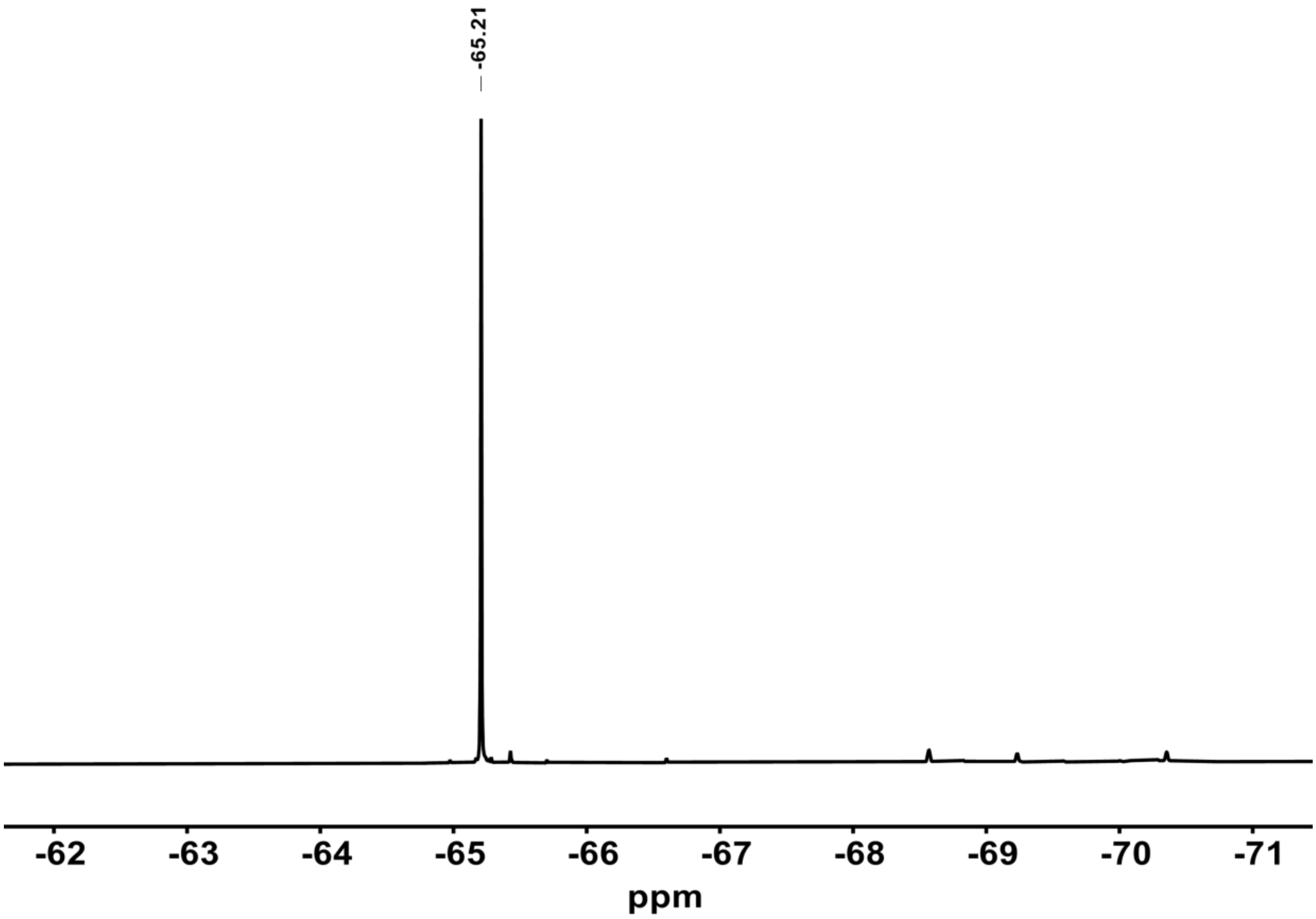


***Figure S62.*** *$^{19}F$ NMR spectrum (377 MHz) of $[HB_2(hpp)_2(CF_3\text{-}Py)][B(cat)_2]$ in $CD_2Cl_2$.*

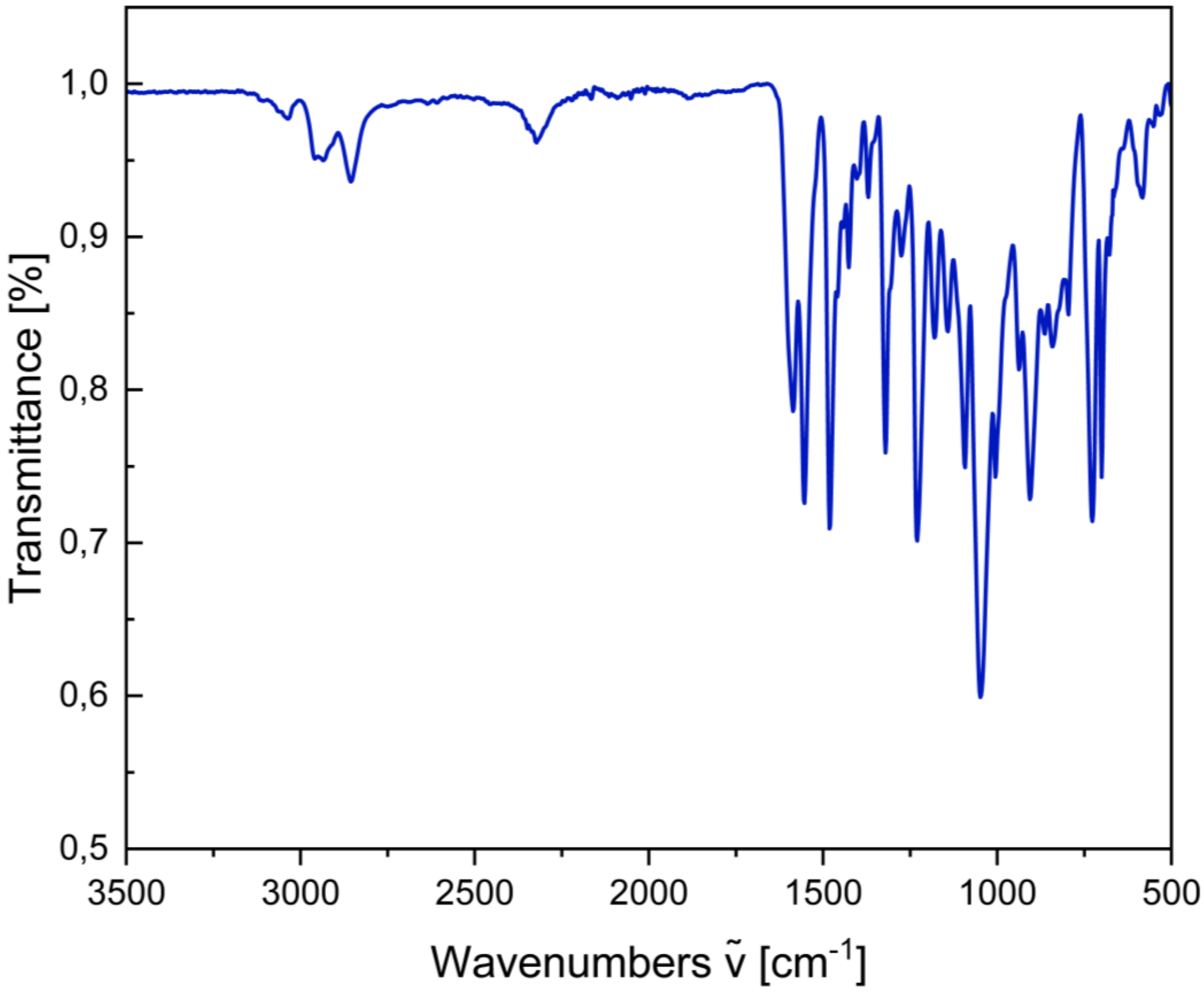


***Figure S63.*** *IR spectrum of [$HB_2(hpp)_2(CF_3$-Py)][$B(cat)_2$] as powder.*

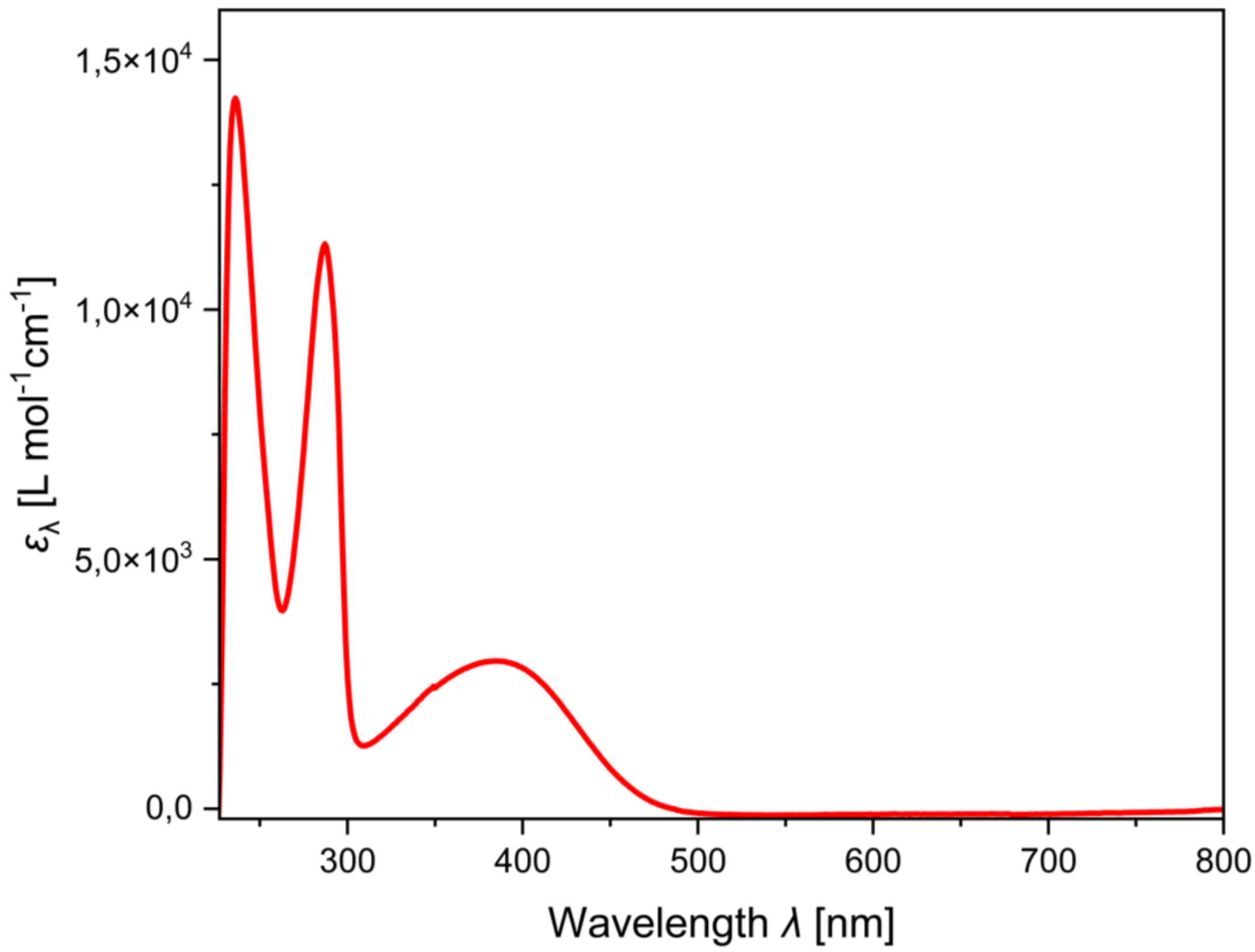


***Figure S64.*** *UV/Vis spectrum of [$HB_2(hpp)_2(CF_3$-Py)][$B(cat)_2$] in $CH_2Cl_2$ ($c = 4.29\cdot10^{-5}$ mol $L^{-1}$).*

***Table S4**. Calculated Gibbs free reaction energies $\Delta G_0$ for the reaction of different lewis bases with triborirane at the B3LYP+D3/def2-TZVP level.*

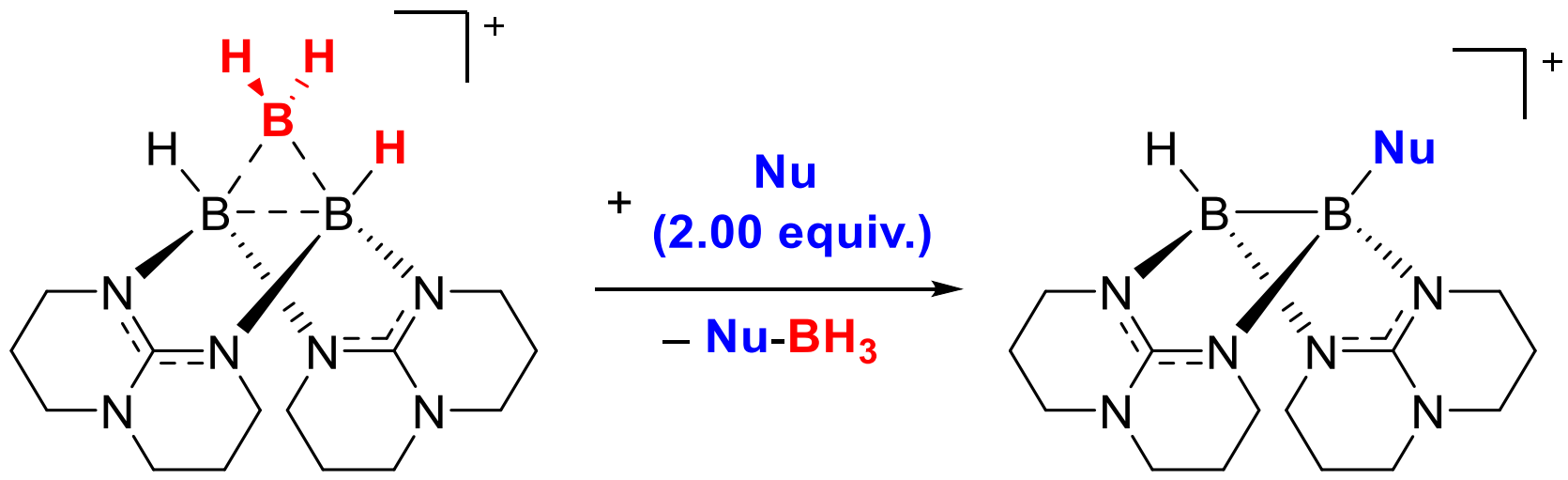


| **nucleophile** | **$\Delta G_0$ [kJ mol$^{-1}$]** |
|---|---|
| $NEt_3$ | 18.2 |
| $PCy_3$ | 16.6 |
| $NO_2$-Py | –35.0 |
| $CF_3$-Py | –39.3 |
| Bipy | –60.8 |
| Py | –62.5 |
| DMAP | –105.2 |

***Table S5**. Calculated Gibbs free reaction energies $\Delta G_0$ for the borane exchange reaction of triborirane at the B3LYP+D3/def2-TZVP level.*

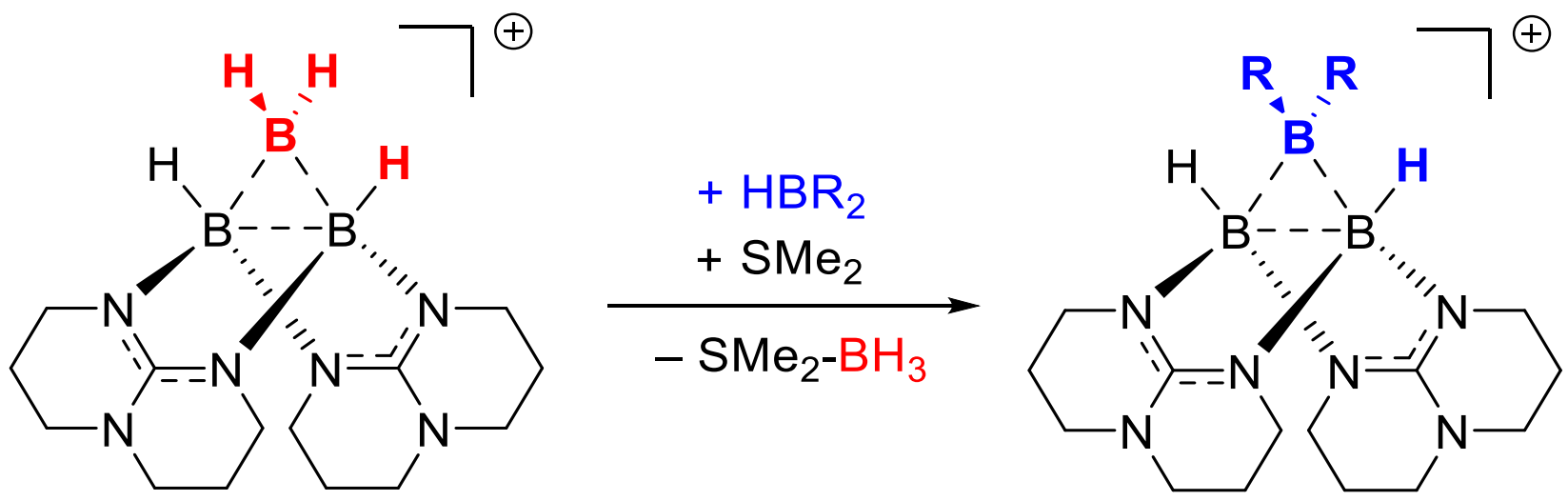


| **borane** | **$\Delta G_0$ [kJ mol$^{-1}$]** |
|---|---|
| HBpin | 33.9 |
| $HBMes_2$ | 30.6 |
| $HB(C_6F_5)_2$ | –11.2 |
| 9-BBN | –42.1 |
| $HBCy_2$ | –42.2 |
| HBcat | –52.6 |

a)

| | state | $\lambda_{calc}$ [nm] | $f$ | Main contribution |
|---|---|---|---|---|
| **DB5⁺** | $S_1$ | 266 | 0.636 | HOMO→ LUMO (95%) |
| **DB6⁺** | $S_1$ | 294 | 0.275 | HOMO→ LUMO (94%) |
| **DB7⁺** | $S_1$ | 323 | 0.320 | HOMO→ LUMO (93%) |

b)

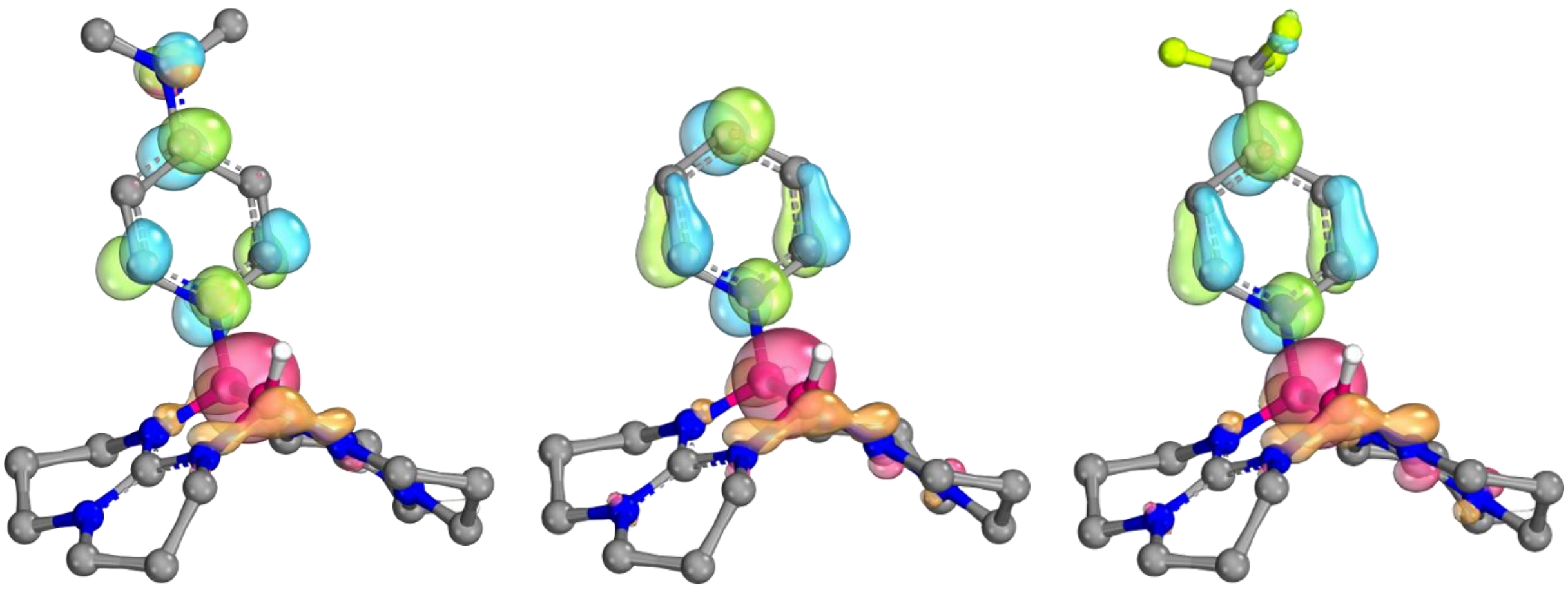

**DB5⁺** **DB6⁺** **DB7⁺**

c)

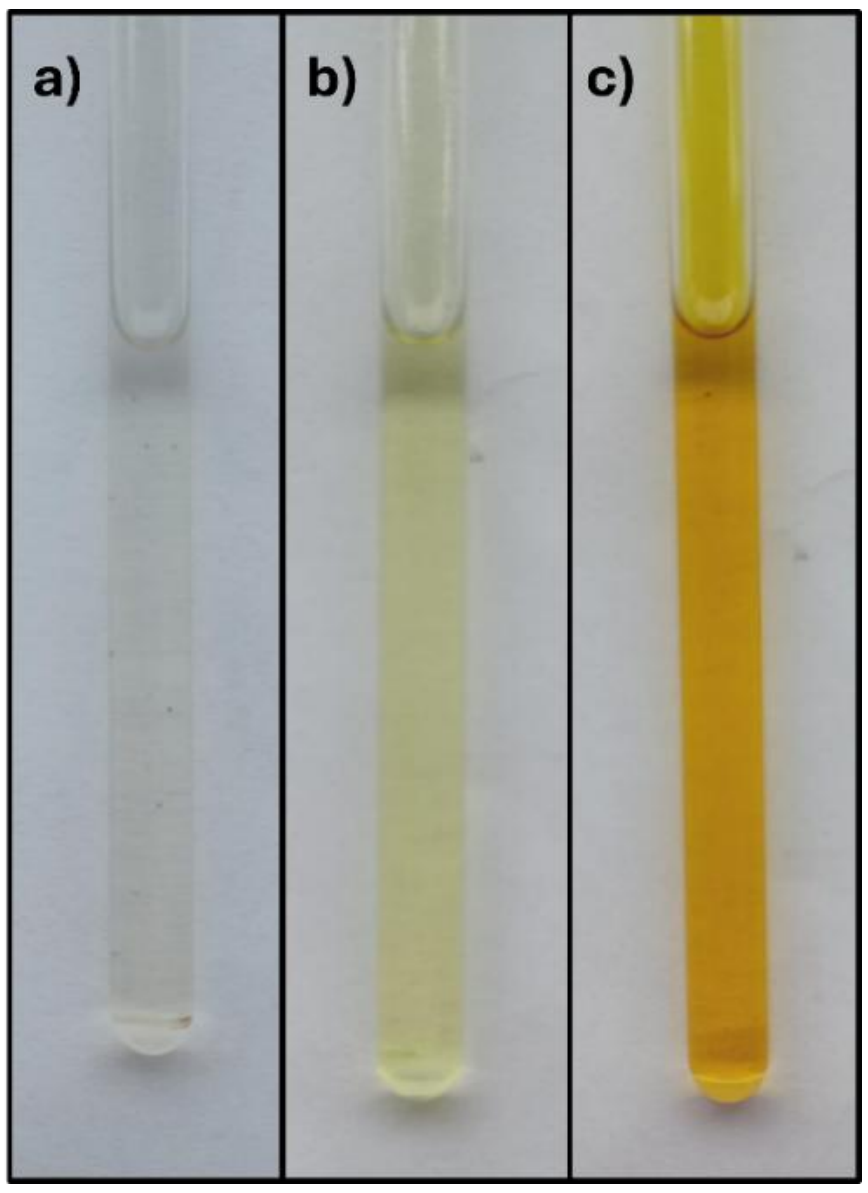


***Figure S65***. *a) Calculated TDDFT parameters of the $S_1$ excitation of **DB5⁺**, **DB6⁺** and **DB7⁺** obtained at the CAM-B3LYP-D3/def2-TZVP/CPCM(DCM) level of theory. b) Visualization of the frontier molecular orbitals for the three cationic diboranes **DB5⁺**, **DB6⁺** and **DB7⁺** calculated at the B3LYP+D3/def2-TZVP level of theory. HOMO (red-orange), LUMO (blue-green). c) color comparison of synthesized pyridine-base-substituted diborane(4) mono-adducts a) 4-dimethylaminopyridine, b) pyridine, c) 4-trifluormethylpyridine.*

# 2.4 Reference Spectra

## 2.4.1 Tricyclohexylphosphine $PCy_3$

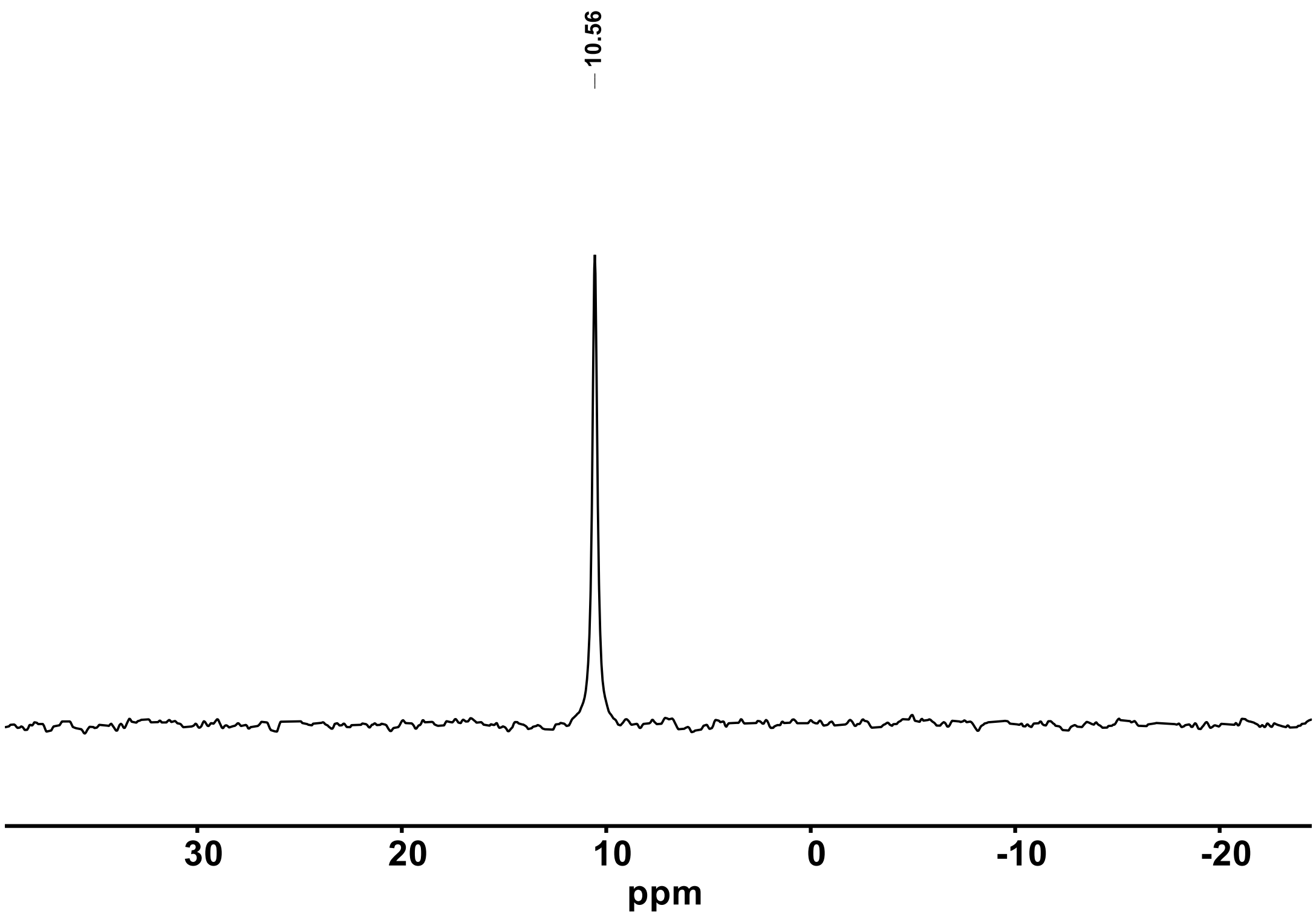


***Figure S66.*** *$^{31}P$ NMR spectrum (243 MHz) of $PCy_3$ in bromobenzene.*

## 2.4.2 1,2-bis(dicyclohexylphosphino)ethane (dcpe)

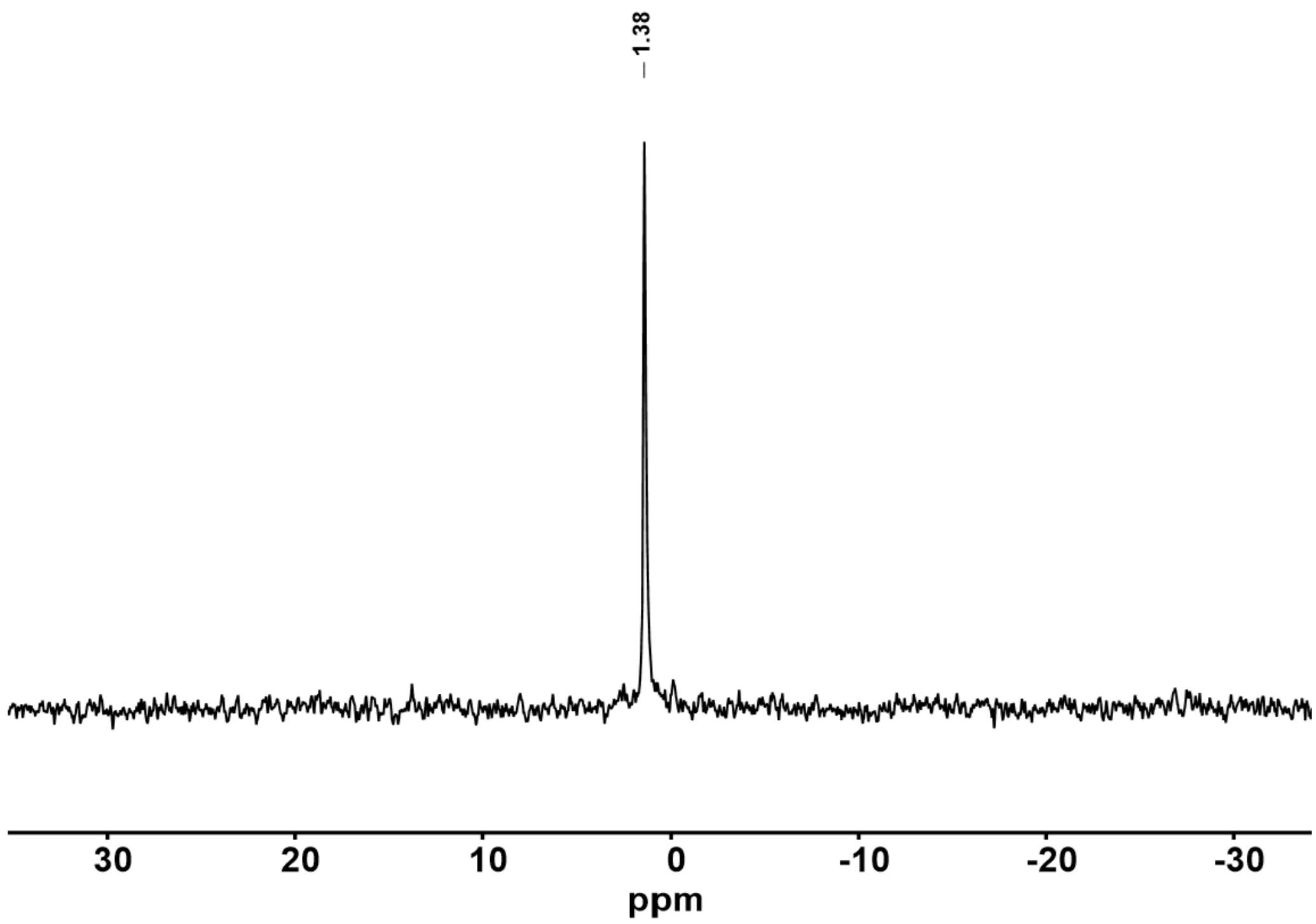


***Figure S67.*** *$^{31}P$ NMR spectrum (162 MHz) of dcpe in $CD_2Cl_2$.*

# 3 Details of the crystal structure determination

| **compound** | **DB3**[OTf]$_2$ | **TB2**[B($C_6F_5$)$_3$] |
|---|---|---|
| CCDC number | 2566210 | 2566211 |
| Empirical formula | $C_{43}H_{74}B_2Cl_2F_6N_6O_6P_2S_2$ | $C_{32}H_4B_4F_{15}N_6$ |
| Formula weight | 1103.66 | 825.85 |
| Temperature [K] | 100.00 | 100.00 |
| Crystal system | monoclinic | triclinic |
| Space group (number) | $P2_1/n$ | P-1 |
| *a* [Å] | 14.4672(7) | 12.4978(7) |
| *b* [Å] | 15.4841(8) | 12.9031(7) |
| *c* [Å] | 22.9936(10) | 13.2412(7) |
| α [°] | 90 | 63.357(2) |
| β [°] | 90.741(2) | 64.393(2) |
| γ [°] | 90 | 76.565(2) |
| Volume [Å$^3$] | 5150.4(4) | 1719.13(17) |
| *Z* | 4 | 2 |
| $\rho_{calc}$ [gcm$^{-3}$] | 1.423 | 1.595 |
| $\mu$ [mm$^{-1}$] | 0.343 | 0.152 |
| *F*(000) | 2328.0 | 836.0 |
| Crystal size [mm$^3$] | 0.58×0.18×0.1 | 0.46×0.31×0.1 |
| Radiation | Mo$K_\alpha$ (λ=0.71073 Å) | Mo$K_\alpha$ (λ=0.71073 Å) |
| 2θ range [°] | 3.854 to 55.968 | 3618 to 54 |
| Index ranges | $-19 \le h \le 19$, $-20 \le k \le 20$, $-30 \le l \le 30$ | $-15 \le h \le 15$, $-16 \le k \le 16$, $-16 \le l \le 16$ |
| Reflections collected | 128565 | 98261 |
| Independent reflections | 12364 $R_{int}$ = 0.0728 $R_{sigma}$ = 0.0333 | 7518 $R_{int}$ = 0.0624 $R_{sigma}$ = 0.0240 |
| Data / Restraints / Parameters | 12364/100/631 | 7518/0/534 |
| Goodness-of-fit on $F^2$ | 1.157 | 1.044 |
| Final *R* indexes [$I \geq 2\sigma(I)$] | $R_1$ = 0.0537 w$R_2$ = 0.1193 | $R_1$ = 0.0412 w$R_2$ = 0.1005 |
| Final *R* indexes [all data] | $R_1$ = 0.0606 w$R_2$ = 0.1193 | $R_1$ = 0.0484 w$R_2$ = 0.1054 |
| Largest peak/hole [eÅ$^{-3}$] | 0.46/-0.71 | 0.34/-0.46 |

| **compound** | **DB5**[B(cat)$_2$] | **DB7**[B(cat)$_2$] |
|---|---|---|
| CCDC number | 2566212 | 2566213 |
| Empirical formula | $C_{33.5}H_{43}B_3ClN_8O_4$ | $C_{35}H_{43}B_3Cl_6F_3N_7O_4$ |
| Formula weight | 690.64 | 927.89 |
| Temperature [K] | 100.00 | 100.00 |
| Crystal system | monoclinic | monoclinic |
| Space group (number) | P 2/c | $P2_1/c$ |
| *a* [Å] | 31.101(3) | 8.4756(7) |
| *b* [Å] | 14.3327(15) | 23.304(2) |
| *c* [Å] | 15.7079(15) | 21.0376(15) |
| α [°] | 90 | 90 |
| β [°] | 101.922(4) | 90.717(3) |
| γ [°] | 90 | 90 |
| Volume [Å$^3$] | 6850.9(11) | 4154.9(6) |
| *Z* | 8 | 4 |
| $\rho_{calc}$ [gcm$^{-3}$] | 1.339 | 1.483 |
| $\mu$ [mm$^{-1}$] | 0.163 | 0.475 |
| *F*(000) | 2920.0 | 1912.0 |
| Crystal size [mm$^3$] | 0.25×0.17×0.1 | 0.17×0.17×0.09 |
| Radiation | Mo$K_\alpha$ (λ=0.71073 Å) | Mo$K_\alpha$ (λ=0.71073 Å) |
| 2θ range [°] | 3.886 to 52 | 3872 to 52.998 |
| Index ranges | −38 ≤ h ≤ 38 −17 ≤ k ≤ 17 −19 ≤ l ≤ 19 | −10 ≤ h ≤ 10 −29 ≤ k ≤ 29 −26 ≤ l ≤ 26 |
| Reflections collected | 347917 | 177513 |
| Independent reflections | 13432 $R_{int}$ = 0.1355 $R_{sigma}$ = 0.0402 | 8608 $R_{int}$ = 0.1009 $R_{sigma}$ = 0.0320 |
| Data / Restraints / Parameters | 13432/0/898 | 8608/9/536 |
| Goodness-of-fit on $F^2$ | 1.172 | 1.177 |
| Final *R* indexes [$I≥2σ(I)$] | $R_1$ = 0.1085 w$R_2$ = 0.2757 | $R_1$ = 0.0713 w$R_2$ = 0.1862 |
| Final *R* indexes [all data] | $R_1$ = 0.1190 w$R_2$ = 0.2817 | $R_1$ = 0.0788 w$R_2$ = 0.1901 |
| Largest peak/hole [eÅ$^{-3}$] | 0.89/-0.48 | 0.55/-0.45 |

## [(dcpe){B(*μ*-hpp)}$_2$][OTf]$_2$ DB4[OTf]$_2$

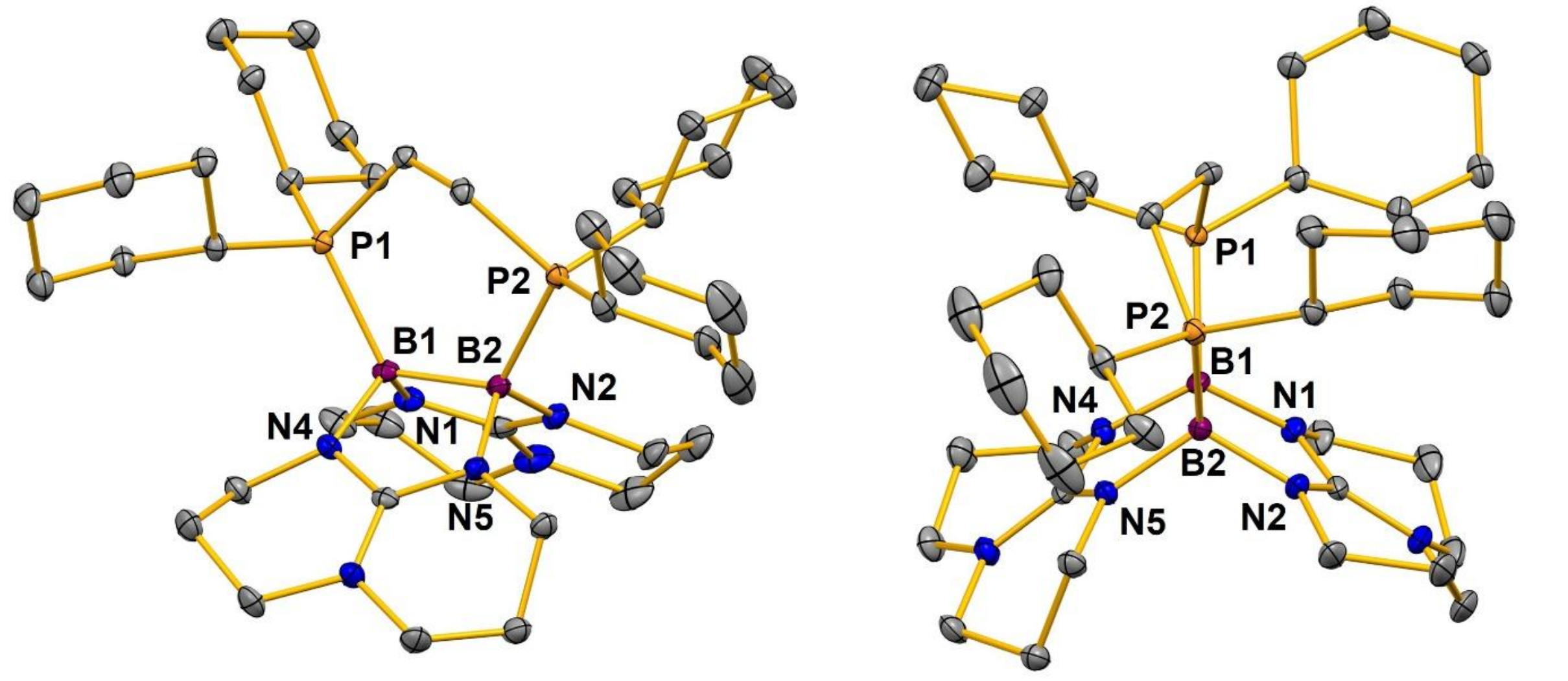


***Figure S68.*** *Illustration of the structure [(dcpe){B(μ-hpp)}$_2$][OTf]$_2$ in the solid state. Color code: B violet, C grey, N blue, P orange. Displacement ellipsoids drawn at the 50% probability level. Counterion, C−H hydrogen atoms and co-crystallized solvent molecules omitted. Selected bond lengths (Å) and angles (°):* B1−B2 1.753(3), B1−P1 2.020(2), B1−N1 1.539(3), B1−N4 1.539(3), B2−P2 2.002(2), B2−N2 1.537(3), B2−N5 1.548(3), N2−B2−N5 114.7(2).

## [(DMAP)(H){B(*μ*-hpp)}$_2$][B(cat)$_2$] DB5[B(cat)$_2$]

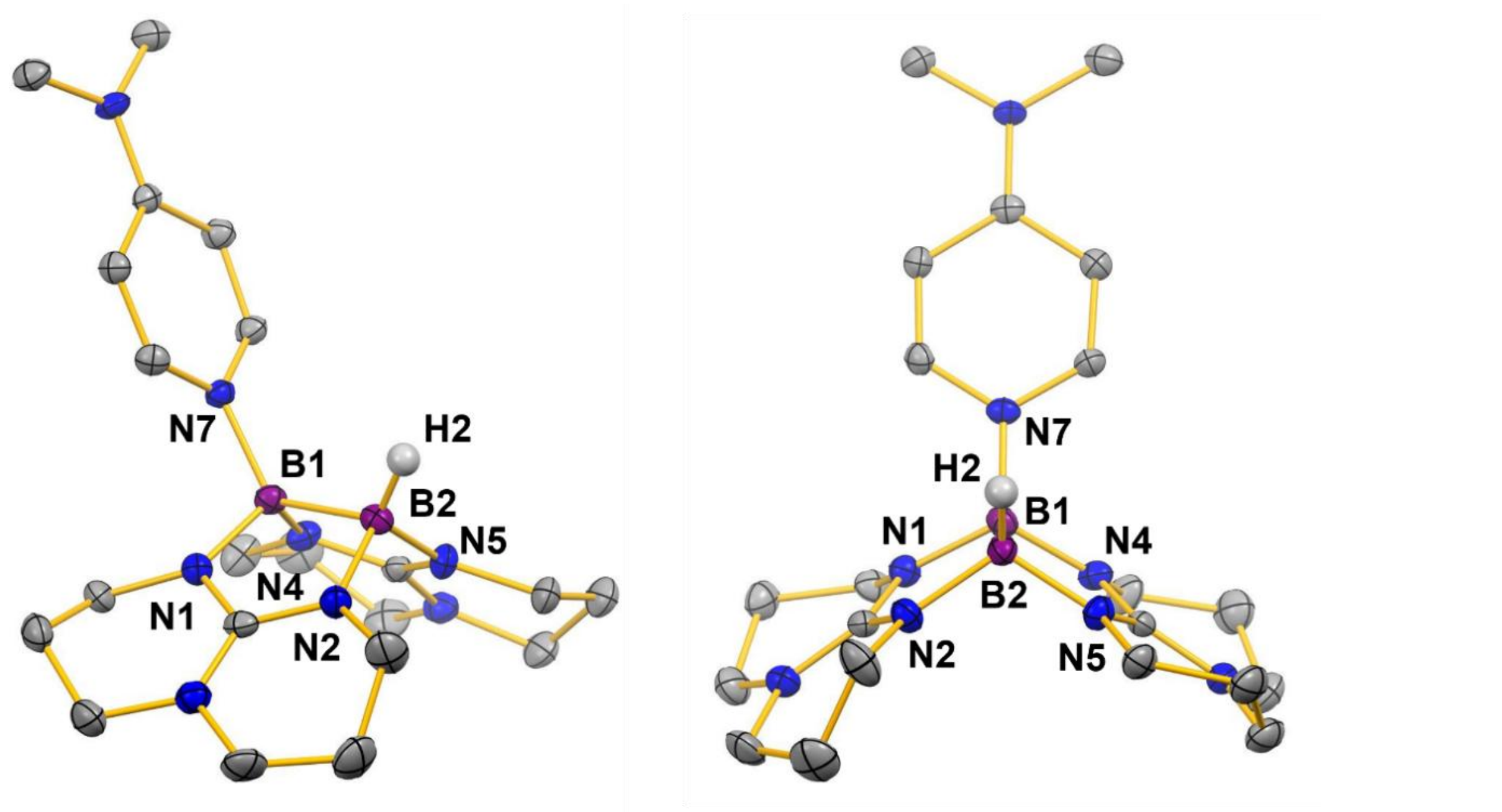


***Figure S69.*** *Illustration of the structure [(DMAP)(H){B(μ-hpp)}$_2$][B(cat)$_2$] in the solid state. Color code: B violet, C grey, N blue. Displacement ellipsoids drawn at the 50% probability level. Counterion, C−H hydrogen atoms and co-crystallized solvent molecules omitted. Selected bond lengths (Å) and angles (°):* B1−B2 1.741(9), B1−N1 1.544(7), B1−N4 1.533(8), B1−N7 1.594(7), B2−N2 1.565(7), B2−N5 1.557(7), N1−B1−N4 114.1(4), BBN DMAP 95.4(4).

## $[(CF_3\text{-Py})(H)\{B(\mu\text{-hpp})\}_2][B(cat)_2]$ DB7$[B(cat)_2]$

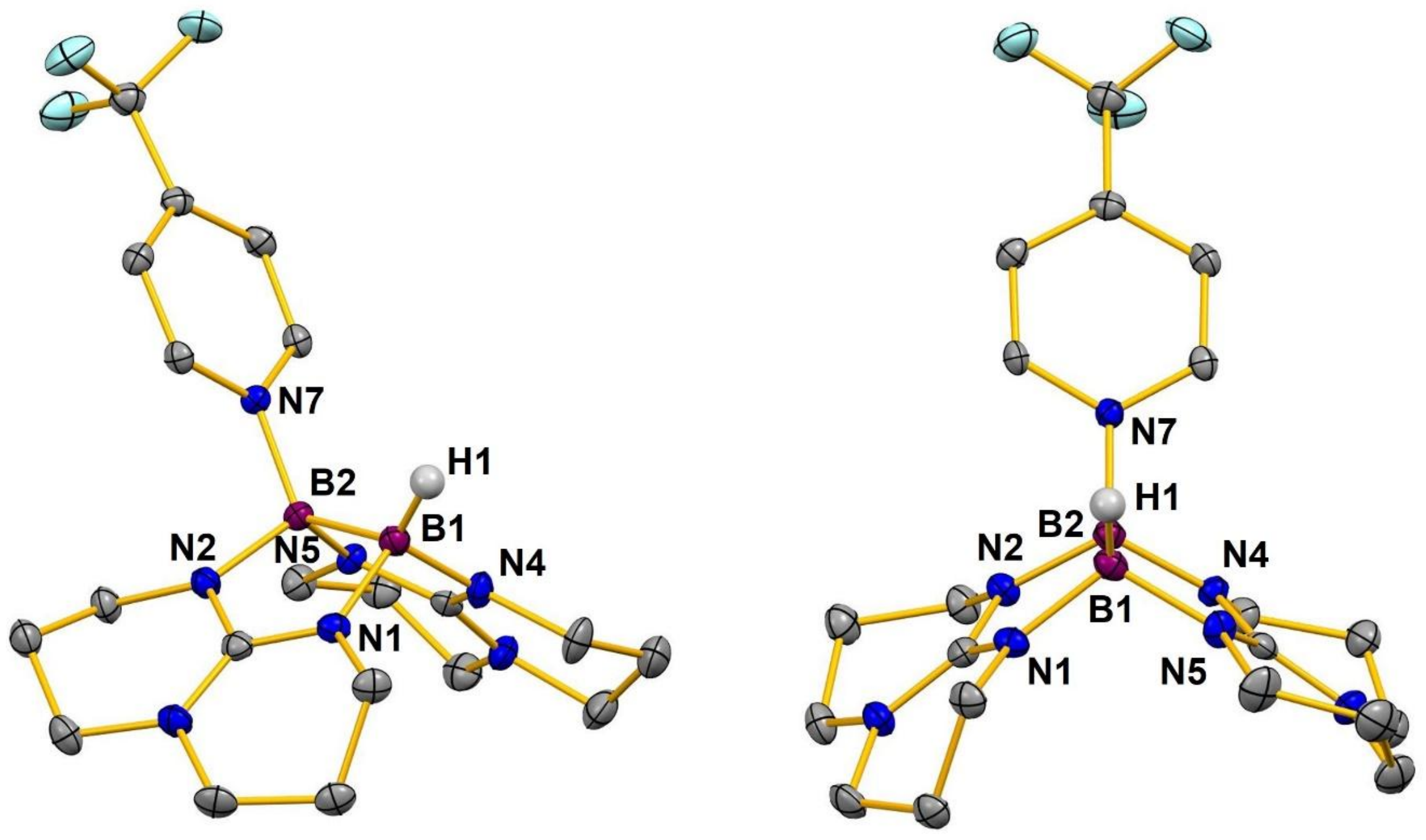


***Figure S70***. *Illustration of the structure [(CF$_3$-Py)(H){B(μ-hpp)}$_2$][B(cat)$_2$] in the solid state. Color code: B violet, C grey, N blue. Displacement ellipsoids drawn at the 50% probability level. Counterion, C−H hydrogen atoms and co-crystallized solvent molecules omitted. Selected bond lengths (Å) and angles (°):* B1−B2 1.737(6), B1−N1 1.562(5), B1−N4 1.563(5), B2−N2 1.545(5), B2−N5 1.541(5), B2−N7 1.605(5), N2−B2−N5 114.9(3), BBN CF$_3$-Py 95.9(5).

## [{HB(*μ*-hpp)}$_2$(*μ*-BH$_2$)][HB(C$_6$F$_5$)$_3$] TB2[HB(C$_6$F$_5$)$_3$]

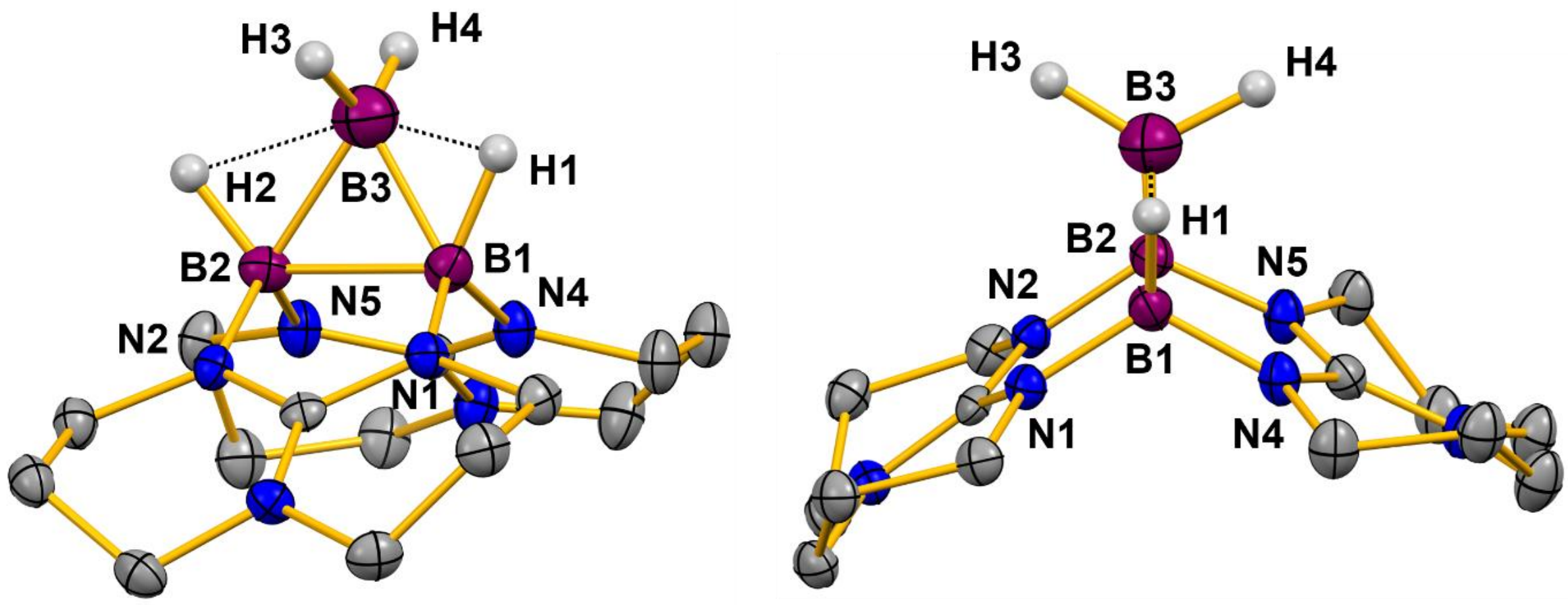


***Figure S71.*** *Illustration of the structure [{HB(μ-hpp)}$_2$(μ-BH$_2$)][HB(C$_6$F$_5$)$_3$] in the solid state. Color code: B violet, C grey, N blue. Displacement ellipsoids drawn at the 50% probability level. Counterion, C−H hydrogen atoms and co-crystallized solvent molecules omitted. Selected bond lengths (in Å) and angles (in °):* (B1−B2 1.927(3), B1−B3 1.715(3), B2−B3 1.861(3), B1−N1 1.503(3), B1−N4 1.501(3), B2−N2 1.520(3), B2−N5 1.517(3), B1−B2−B3 53.8(1), B1−B3−B2 65.0(1), B2−B1−B3 61.1(1), N2−B2−N5 116.9(2).

### [(PCy$_3$)(H){B($\mu$-hpp)}$_2$][HB(C$_6$F$_5$)$_3$] DB2[HB(C$_6$F$_5$)$_3$]

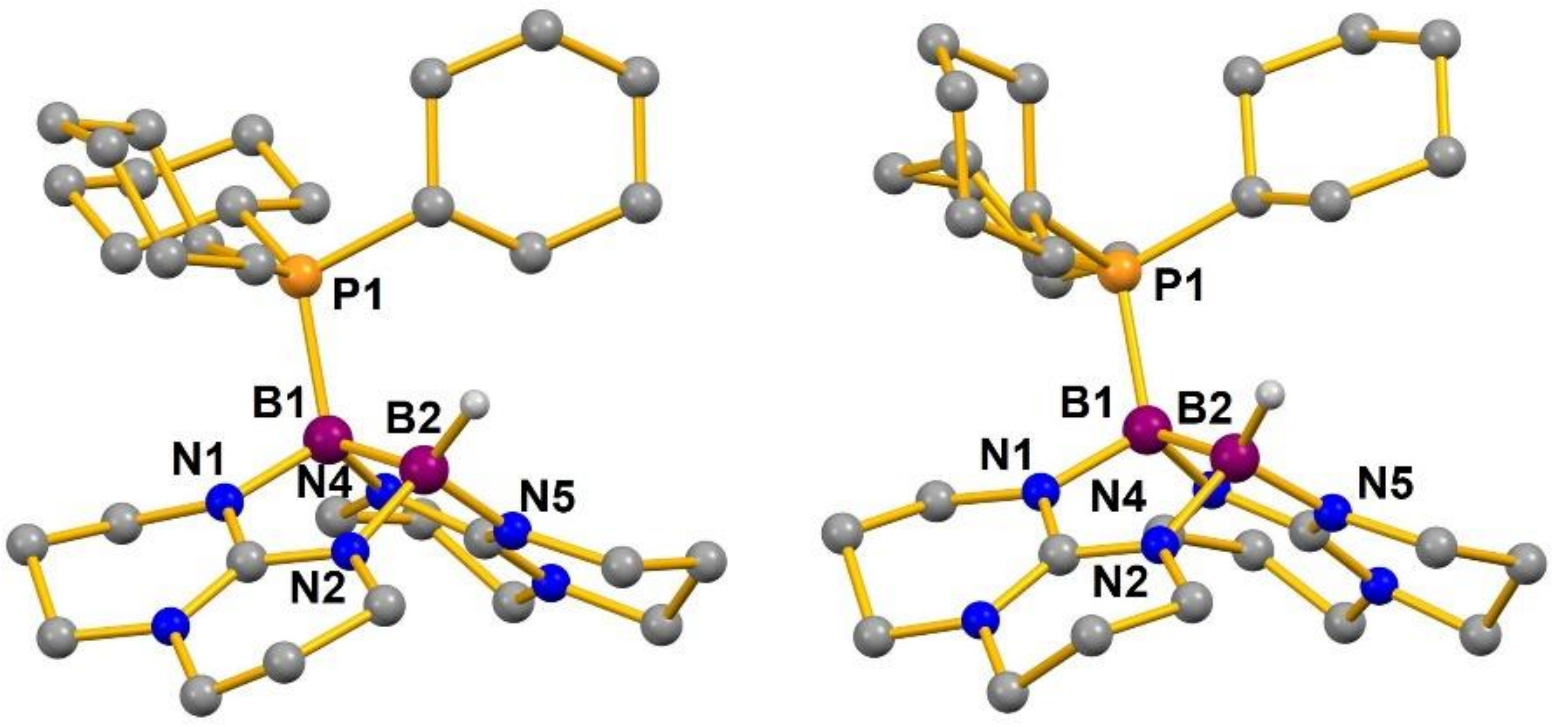


| | **DB2**[HB(C$_6$F$_5$)$_3$] exp.[xx] | **DB2**$^+$ DFT (gas phase) | **DB2**$^+$ DFT (CH$_2$Cl$_2$) |
|---|---|---|---|
| **B$^1$–B$^2$** | 1.749(4) | 1.744 | 1.745 |
| **B$^1$–N$^1$** | 1.528(3) | 1.555 | 1.553 |
| **B$^2$–N$^2$** | 1.561(4) | 1.567 | 1.565 |
| **B$^1$–P$^1$** | 2.004(3) | 1.997 | 1.995 |
| **∡(N$^1$B$^1$N$^4$)** | 115.0(2) | 113.7 | 113.8 |

***Figure S72.*** *Comparison of the structure [(PCy$_3$)(H){B(μ-hpp)}$_2$][HB(C$_6$F$_5$)$_3$] in solid state with the calculated minimum structure **DB2$^+$** (B3LYP+D3/def2-TZVP). Displacement ellipsoids drawn at the 50% probability level. C−H hydrogens and counterions are omitted. Color code: B violet, C grey, N blue, P orange.*

### [(dcpe){B($\mu$-hpp)}$_2$][OTf]$_2$ DB3[OTf]$_2$

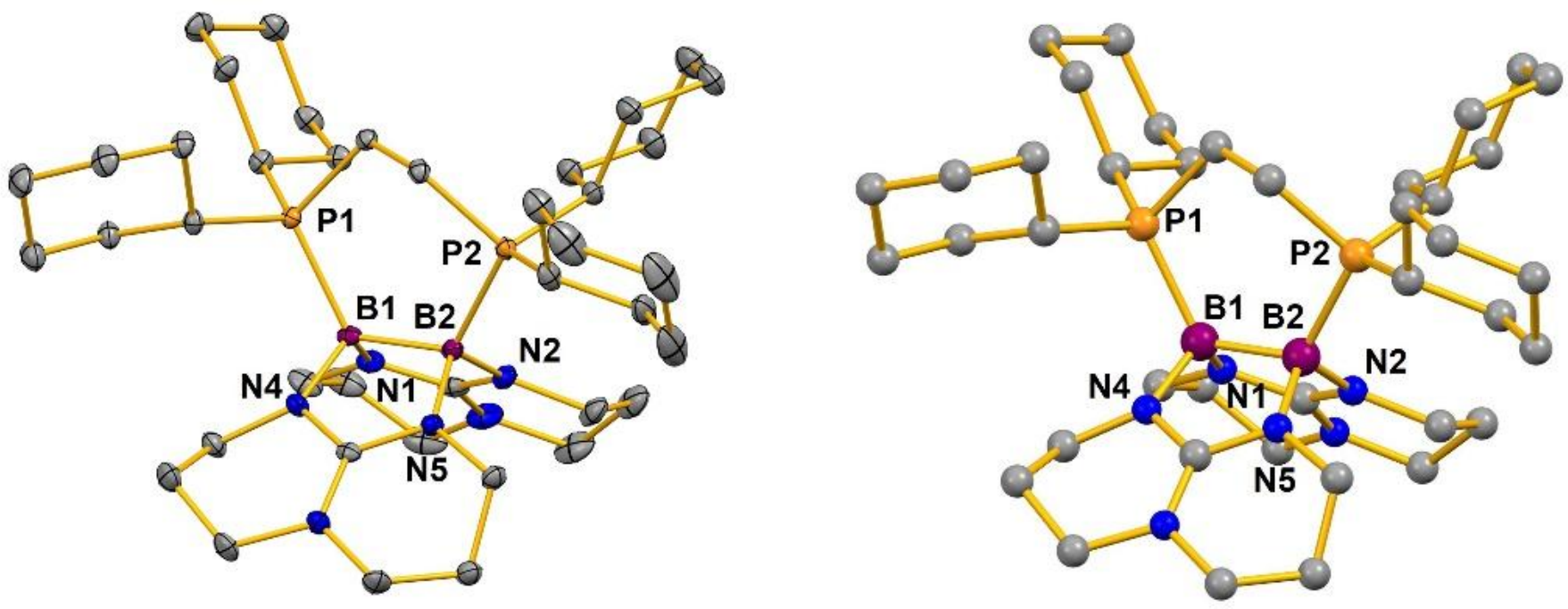


| | **DB3**[OTf]$_2$ exp. (this work) | **DB3**$^+$ DFT (gas phase) |
|---|---|---|
| **B$^1$–B$^2$** | 1.753(3) | 1.757 |
| **B$^1$–N$^1$** | 1.539(3) | 1.541 |
| **B$^2$–N$^2$** | 1.537(3) | 1.541 |
| **B$^1$–P$^1$** | 2.020(2) | 2.025 |
| **∡(N$^2$B$^2$N$^5$)** | 114.7(2) | 114.0 |

***Figure S73.*** *Comparison of the structure [(dcpe){B(μ-hpp)}$_2$][OTf]$_2$ in solid state with the calculated minimum structure **DB3$^+$** (B3LYP+D3/def2-TZVP). Displacement ellipsoids drawn at the 50% probability level. C−H hydrogens and counterions are omitted. Color code: B violet, C grey, N blue, P orange.*

## [{HB($\mu$-hpp)}$_2$($\mu$-BH$_2$)][HB(C$_6$F$_5$)$_3$] TB2[HB(C$_6$F$_5$)$_3$]

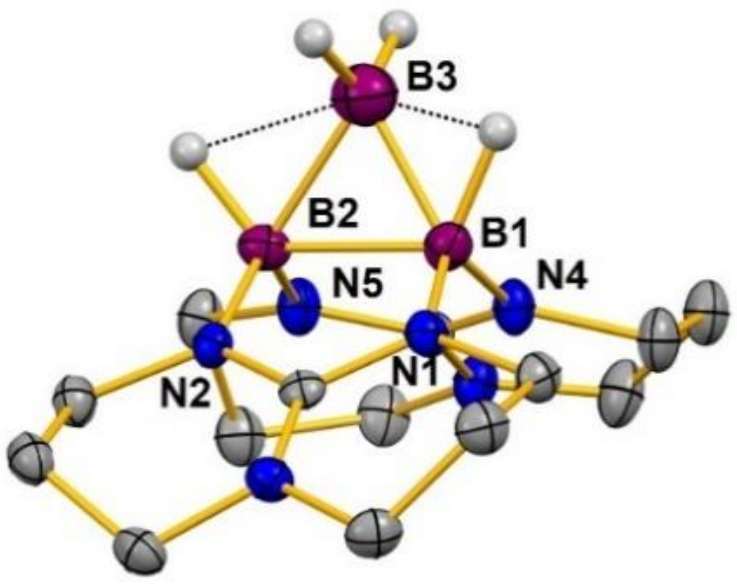


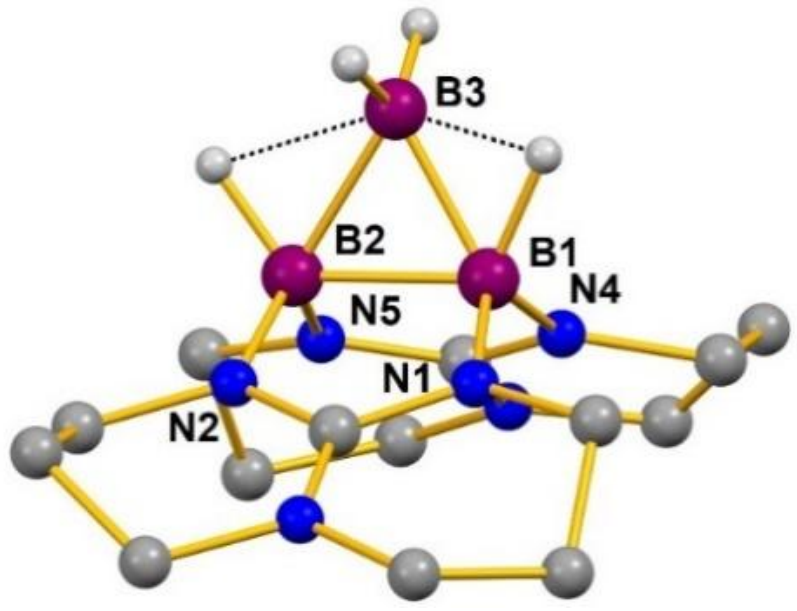


| | **TB2**[HB(C$_6$F$_5$)$_3$] exp. (this work) | **TB2**$^+$ DFT (gas phase) |
|---|---|---|
| **B$^1$–B$^2$** | 1.927(3) | 1.841 |
| **B$^1$–B$^3$** | 1.715(3) | 1.732 |
| **B$^2$–B$^3$** | 1.861(3) | 1.844 |
| **B$^1$–N$^1$** | 1.503(3) | 1.509 |
| **B$^2$–N$^2$** | 1.520(3) | 1.532 |
| **∡(B$^1$B$^2$B$^3$)** | 53.8(1) | 56.1 |
| **∡(B$^1$B$^3$B$^2$)** | 65.0(1) | 61.9 |
| **∡(B$^2$B$^1$B$^3$)** | 61.1(1) | 62.0 |
| **∡(N$^2$B$^2$N$^5$)** | 116.9(2) | 117.1 |

***Figure S74.*** *Comparison of the structure [{HB(μ-hpp)}$_2$(μ-BH$_2$)][HB(C$_6$F$_5$)$_3$] in solid state with the calculated minimum structure **TB2**$^+$ (B3LYP+D3/def2-TZVP). Displacement ellipsoids drawn at the 50% probability level. C−H hydrogens and counterions are omitted. Color code: B violet, C grey, N blue.*

## [(DMAP)(H){B(*μ*-hpp)}$_2$][B(cat)$_2$] DB5[B(cat)$_2$]

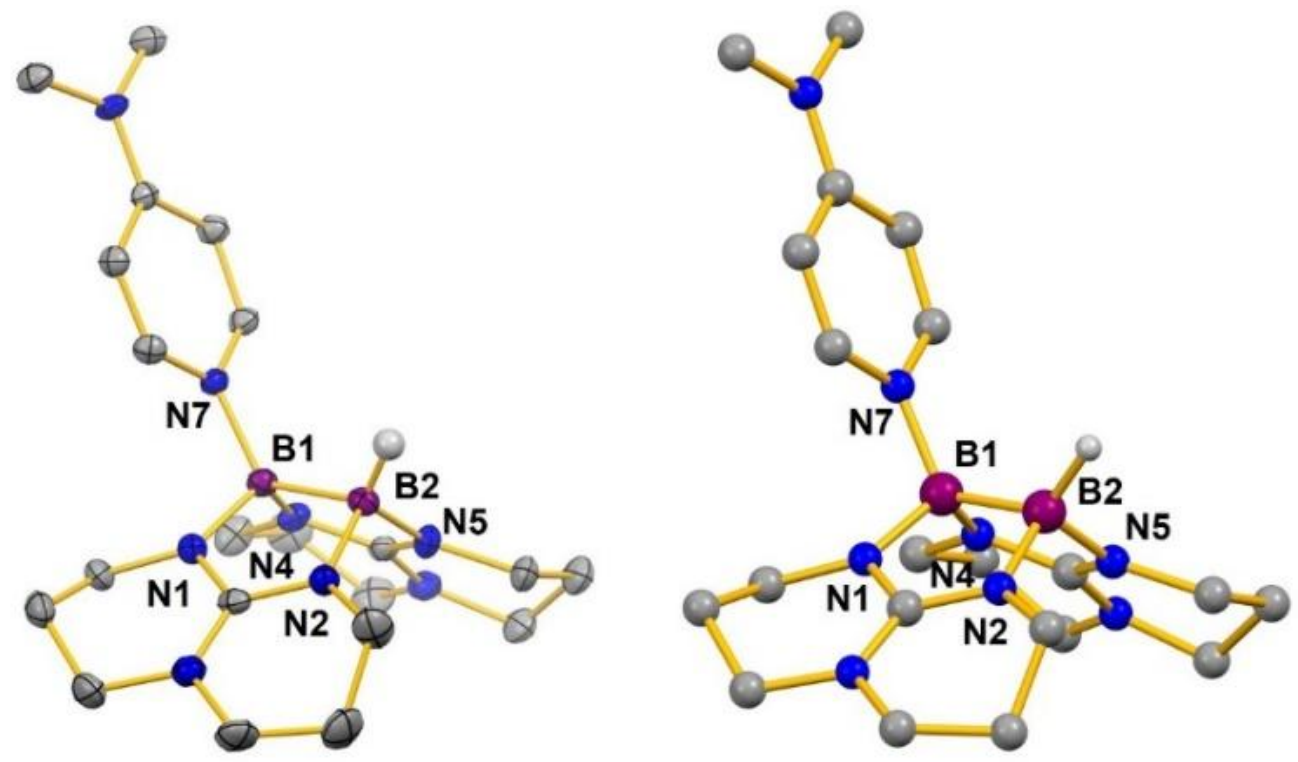


| | **DB5**[B(cat)$_2$] exp. (this work) | **DB5**$^+$ DFT (gas phase) | **DB5**$^+$ DFT ($CH_2Cl_2$) |
|---|---|---|---|
| **$B^1$–$N^2$** | 1.741(9) | 1.731 | 1.733 |
| **$B^1$–$N^1$** | 1.554(7) | 1.543 | 1.543 |
| **$B^2$–$N^2$** | 1.565(7) | 1.569 | 1.565 |
| **$B^1$–$N^7$** | 1.594(7) | 1.594 | 1.593 |
| **$\measuredangle(N^1B^1N^4)$** | 114.1(4) | 114.6 | 114.2 |

***Figure S75.*** *Comparison of the structure [(DMAP)(H){B(μ-hpp)}$_2$][B(cat)$_2$] in solid state with the calculated minimum structure **DB5$^+$** (B3LYP+D3/def2-TZVP). Displacement ellipsoids drawn at the 50% probability level. C−H hydrogens and counterions are omitted. Color code: B violet, C grey, N blue.*

## [(CF$_3$-Py)(H){B(*μ*-hpp)}$_2$][B(cat)$_2$] DB7[B(cat)$_2$]

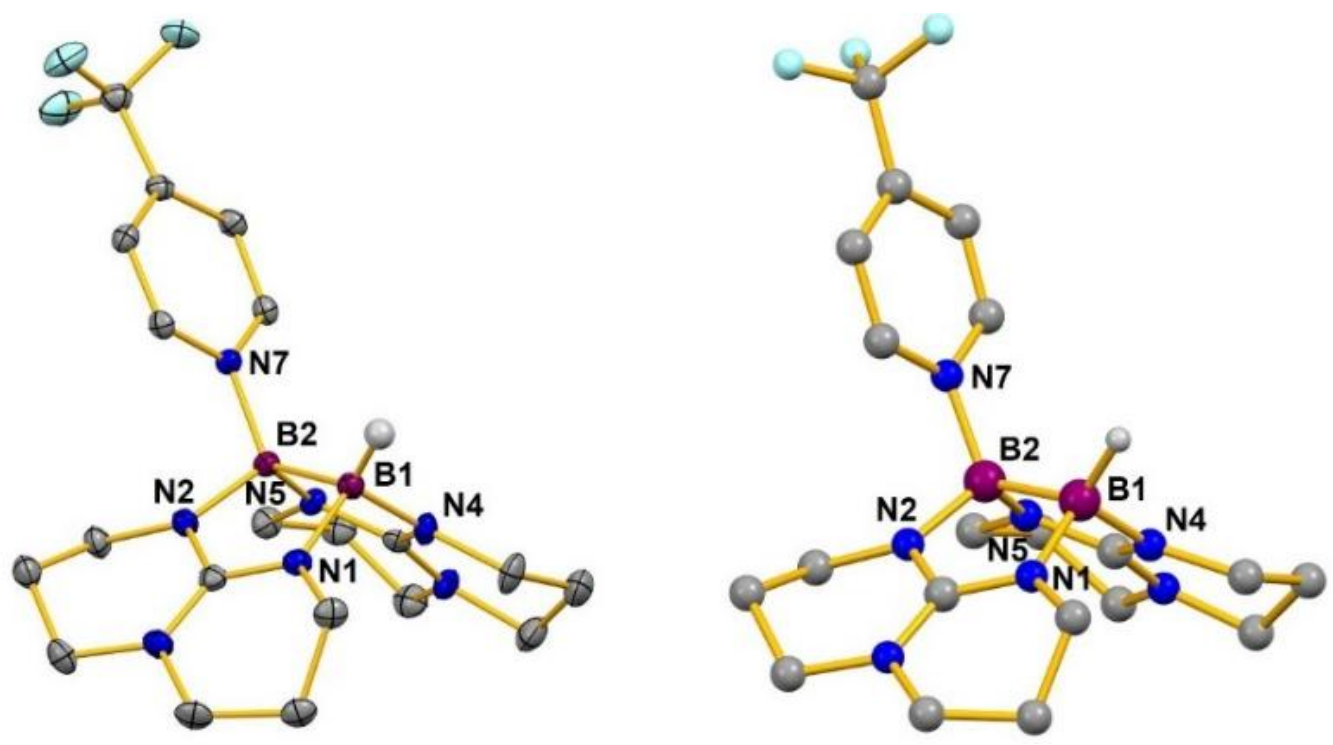


| | **DB7**[B(cat)$_2$] exp. (this work) | **DB7**$^+$ DFT (gas phase) | **DB5**$^+$ DFT ($CH_2Cl_2$) |
|---|---|---|---|
| **$B^1$–$B^2$** | 1.737(6) | 1.741 | 1.742 |
| **$B^1$–$N^1$** | 1.545(5) | 1.534 | 1.533 |
| **$B^2$–$N^2$** | 1.562(5) | 1.564 | 1.561 |
| **$B^1$–$N^7$** | 1.605(5) | 1.592 | 1.592 |
| **$\measuredangle(N^1B^1N^4)$** | 114.9(3) | 116.1 | 115.7 |

***Figure S76.*** *Comparison of the structure [(CF$_3$-Py)(H){B(μ-hpp)}$_2$][B(cat)$_2$] in solid state with the calculated minimum structure **DB7$^+$** (B3LYP+D3/def2-TZVP). Displacement ellipsoids drawn at the 50% probability level. C−H hydrogens and counterions are omitted. Color code: B violet, C grey, N blue, F light blue.*

# 4 Details of the quantum-chemical calculations

All quantum chemical (DFT) calculations were either performed with the 7.7 suite of the TURBOMOLE program[21] or the 6.1 suite of the ORCA program[22,23] using the B3LYP functional[24] (with dispersion correction D3)[25] together with the def2-TZVP basis set.[26] With TURBOMOLE, solvent effects were accounted using the COSMO continuum solvation model for dichloromethane (ε = 8.93) and bromobenzene (ε = 5.4), with COSMO files generated using COSMOprep. With ORCA, solvent effects were accounted using the CPCM (Conductor-like Polarizable Continuum Model) continuum solvation model with CPCM(DCM). TDDFT calculations were performed using the CAM-B3LYP functional instead.[27] The RI (resolution identity) approximation was applied for structure optimization. All minimum structures are stationary points on the energy potential surface as confirmed by frequency computations.

Hydrogen atoms that are not bonded to a boron atom are omitted for clarity in all structure visualizations. Color code: P orange, B purple, N blue, C grey, H white.

Calculated transition state structure.

**[{HB(*μ*-hpp)}$_2$(*μ*-BH$_2$)]**

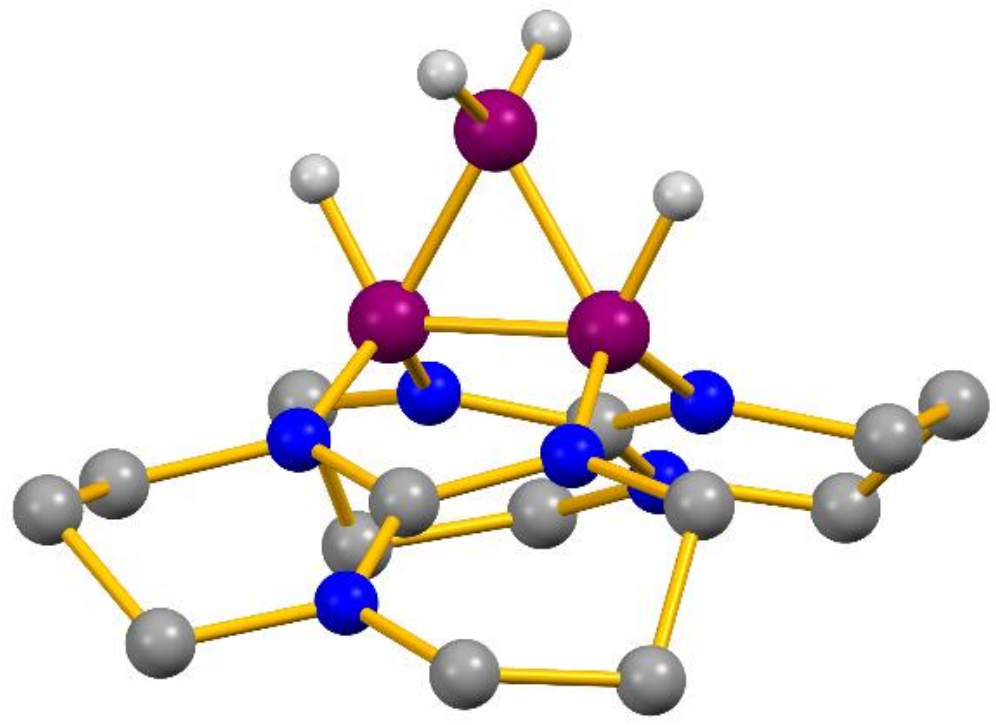

Energy: -953.220643045660
xyz, charge: 1, multiplicity: 1

Cartesian coordinates (in Å)

| | | | |
|---|---|---|---|
| N | 1.73476457104724 | -0.05442583320376 | -0.40189264237732 |
| N | 0.19204809035755 | -1.70981583367989 | -0.37377167588780 |
| N | 2.31855218723668 | -2.12885870478179 | 0.54300719688156 |
| N | -0.19204889136635 | 1.70981284427117 | -0.37377169934533 |
| N | -1.73476687791654 | 0.05442435315735 | -0.40188826427466 |
| N | -2.31855075429716 | 2.12885973736311 | 0.54300913264830 |
| C | 1.43781516416059 | -1.31861726184465 | -0.06018607611082 |
| C | 3.03757012712279 | 0.51949066153559 | -0.08648977875499 |
| H | 3.01566253857048 | 1.00274757673641 | 0.89725972630335 |
| H | 3.25304396230576 | 1.29073334150050 | -0.82604549840288 |
| C | 4.10282124473191 | -0.56533134911052 | -0.11677322704870 |
| H | 4.21474366265844 | -0.93320700538051 | -1.13891904330978 |
| H | 5.06531398054171 | -0.16504493723365 | 0.20142491999985 |
| C | 3.69843854451741 | -1.70657794911848 | 0.79975736459347 |
| H | 4.34225819534238 | -2.57234434604373 | 0.63704467373868 |
| H | 3.79732857717379 | -1.41725858024096 | 1.85131424053305 |
| C | 1.95771769508205 | -3.49139931825552 | 0.94349568669856 |
| H | 2.45927926590014 | -3.70005124157148 | 1.88993352757974 |
| H | 2.34566040847529 | -4.19883552589556 | 0.20318437624728 |

| | | | |
|---|---|---|---|
| C | 0.45361578430281 | -3.63915192308736 | 1.09383654375505 |
| H | 0.11599320221993 | -3.09711187931594 | 1.98026382449629 |
| H | 0.20272128606621 | -4.69084612610081 | 1.23092351980878 |
| C | -0.24264825494862 | -3.08140251547149 | -0.13682278868803 |
| H | -0.03051500880280 | -3.70248209314127 | -1.01449960474758 |
| H | -1.32347935305303 | -3.07820208027820 | 0.00216567757651 |
| C | -1.43781551968698 | 1.31861657225188 | -0.06018422366396 |
| C | 0.24265328279552 | 3.08139705716750 | -0.13681897299330 |
| H | 1.32348411991866 | 3.07819121475259 | 0.00217179066763 |
| H | -0.11599061484454 | 3.09710581407202 | 1.98026735679907 |
| C | -0.45361035030663 | 3.63914762185856 | 1.09383992635460 |
| H | 0.03052483961205 | 3.70247945679185 | -1.01449488817499 |
| H | -0.20271203053785 | 4.69084065452074 | 1.23092857021671 |
| C | -1.95771263241914 | 3.49139968594964 | 0.94349713393773 |
| H | -2.45927456049897 | 3.70005398646519 | 1.88993426314975 |
| H | -2.34565163348972 | 4.19883639548751 | 0.20318446367290 |
| C | -3.69843967148041 | 1.70658406731704 | 0.79975425449957 |
| H | -4.34225551880335 | 2.57235239300311 | 0.63703704657073 |
| H | -3.79733557660395 | 1.41726718731493 | 1.85131121354317 |
| C | -4.10282299381765 | 0.56533736462249 | -0.11677630017697 |
| H | -4.21473988509579 | 0.93321246304679 | -1.13892299894753 |
| H | -5.06531852911769 | 0.16505473828055 | 0.20141851836155 |
| C | -3.03757531046122 | -0.51948797279430 | -0.08648808811267 |
| H | -3.01567206507956 | -1.00274290586863 | 0.89726250224840 |
| H | -3.25305007401232 | -1.29073142784212 | -0.82604275821446 |
| H | 1.01634699211105 | 1.12146411484289 | -2.21644738351595 |
| H | -1.01634921823186 | -1.12146252484136 | -2.21644677925615 |
| B | -0.61789259231574 | -0.67058825611713 | -1.13306578629899 |
| B | 0.61788998807289 | 0.67058667307075 | -1.13307057956398 |
| H | -0.76443073606701 | 0.69362765615379 | -3.24847448993326 |
| H | 0.76441828373012 | -0.69363931130436 | -3.24847359657116 |
| B | -0.00000734079857 | -0.00000672901051 | -2.66102535323837 |

Calculated minimum structures.

**[{HB(*μ*-hpp)}$_2$(*μ*-BH$_2$)]**

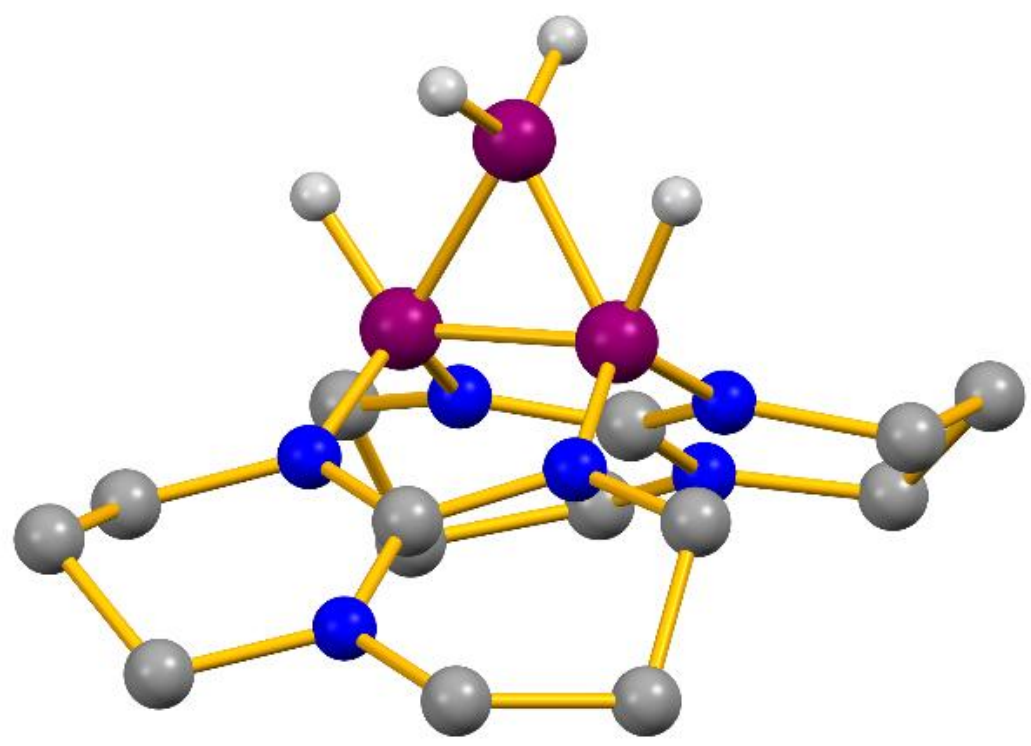

Energy: -953.220729658887
xyz, charge: 1, multiplicity: 1

Cartesian coordinates (in Å)

| | | | |
|---|---|---|---|
| N | 1.72561858903943 | -0.05847578046575 | -0.41577883926623 |

| | | | |
|---|---|---|---|
| N | 0.18774275199694 | -1.71720670726894 | -0.37285908063294 |
| N | 2.31692119679266 | -2.12398395575200 | 0.54247509517832 |
| N | -0.19506261069542 | 1.69971784439542 | -0.38703680856299 |
| N | -1.74198526889376 | 0.04843317407469 | -0.40098252610891 |
| N | -2.31280720242258 | 2.12571722342572 | 0.54526546937208 |
| C | 1.42950267025725 | -1.32372228563418 | -0.06434649905507 |
| C | 3.01877744075239 | 0.52838246013620 | -0.08530477230112 |
| H | 2.98508810115596 | 1.00440007831355 | 0.90169144329446 |
| H | 3.23442707892906 | 1.30649399372978 | -0.81788977643936 |
| C | 4.09294773711811 | -0.54753433878173 | -0.11575282503742 |
| H | 4.21071897466462 | -0.91057418387062 | -1.13889224017985 |
| H | 5.05114793137054 | -0.14061556125530 | 0.20693857390959 |
| C | 3.69498932076854 | -1.69456676311741 | 0.79643352085051 |
| H | 4.34360855918145 | -2.55602357567981 | 0.62949833917480 |
| H | 3.79452887879499 | -1.40869135619522 | 1.84891822986419 |
| C | 1.96332819574837 | -3.48471232048158 | 0.95636938138858 |
| H | 2.46199463135082 | -3.68010449667202 | 1.90724853584517 |
| H | 2.36018572848190 | -4.19628286181193 | 0.22493082173772 |
| C | 0.45947159224333 | -3.64156769874006 | 1.09981995378437 |
| H | 0.11381129828029 | -3.09843245111335 | 1.98249447876592 |
| H | 0.21493810324520 | -4.69434020272612 | 1.24015779690714 |
| C | -0.23454331722173 | -3.09296871391764 | -0.13609440497091 |
| H | -0.01209184806265 | -3.71245038478406 | -1.01223115080265 |
| H | -1.31593207218776 | -3.09912474415182 | -0.00394676186467 |
| C | -1.44212369947341 | 1.30897278657871 | -0.06411962996165 |
| C | 0.25376520745095 | 3.06573694914859 | -0.14278969637032 |
| H | 1.33462250706125 | 3.05226725662226 | -0.00330550632983 |
| H | -0.10349813962609 | 3.07716926288698 | 1.97484185232891 |
| C | -0.43752077872996 | 3.62425932689514 | 1.09016535397051 |
| H | 0.04711934567400 | 3.69361994196165 | -1.01692383591260 |
| H | -0.17877984845569 | 4.67370812365219 | 1.22970705655933 |
| C | -1.94244225300560 | 3.48716625099817 | 0.93955650587799 |
| H | -2.44238001367956 | 3.70306251983754 | 1.88513250163635 |
| H | -2.32469963828623 | 4.19527494000948 | 0.19680644776929 |
| C | -3.69360465232674 | 1.71068031295908 | 0.80967080032049 |
| H | -4.33272633888876 | 2.58147792935665 | 0.65585527809265 |
| H | -3.78736953362015 | 1.41579835353264 | 1.86009767426131 |
| C | -4.10885860812751 | 0.57752100094191 | -0.11174719319679 |
| H | -4.22157767609641 | 0.95234229402601 | -1.13132340570728 |
| H | -5.07307270643000 | 0.18172713480893 | 0.20688594782985 |
| C | -3.05114460200308 | -0.51466658190935 | -0.09079072188581 |
| H | -3.03239947624436 | -1.00570771415217 | 0.88909841529013 |
| H | -3.27054687937792 | -1.27860374443854 | -0.83634230601030 |
| H | 0.98278976085395 | 1.08474050971312 | -2.25381656927098 |
| H | -1.10580269169071 | -1.22034261145156 | -2.16356669486183 |
| B | -0.64832348816661 | -0.70432015873353 | -1.16155810250092 |
| B | 0.59820710914404 | 0.64863801182666 | -1.12856062743586 |
| H | -0.75267642385229 | 0.71369273445448 | -3.25230403617873 |
| H | 0.77678535050885 | -0.67470211505157 | -3.25869268633545 |
| B | 0.05894450670008 | 0.06844169387073 | -2.67366897682920 |

**[{HB($\mu$-hpp)}$_2$($\mu$-BH$_2$)]**

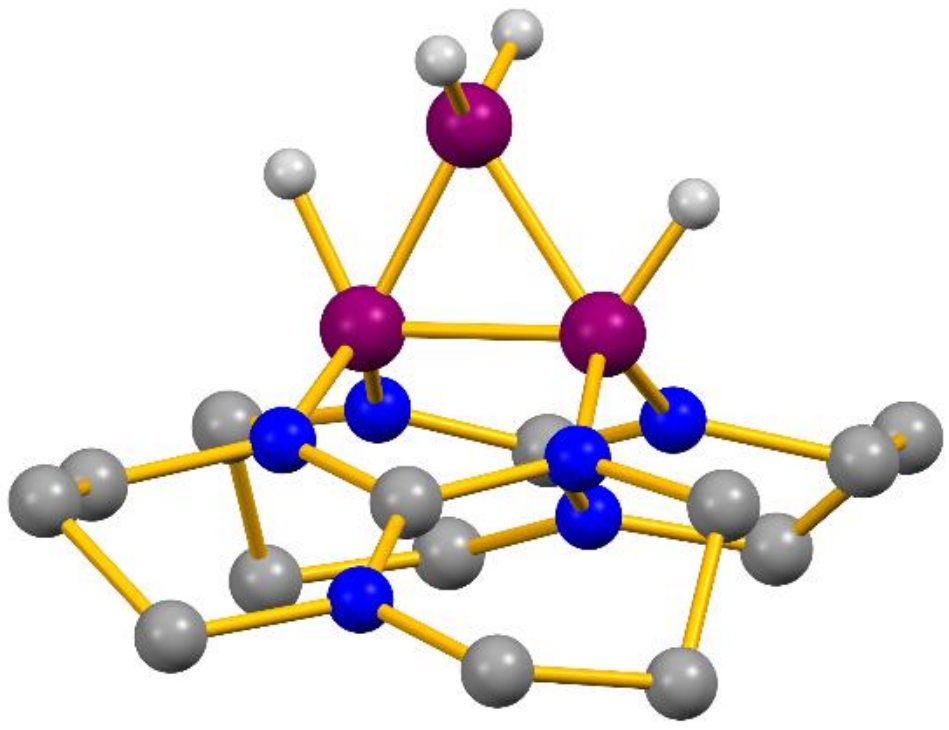

Energy: -953.220729667460
xyz, charge: 1, multiplicity: 1

Cartesian coordinates (in Å)

| | | | |
|---|---|---|---|
| N | 1.74200481688127 | -0.04842919814741 | -0.40101865048036 |
| N | 0.19505944556494 | -1.69969065076834 | -0.38702826750695 |
| N | 2.31280648015890 | -2.12570579604865 | 0.54525610024423 |
| N | -0.18775269346952 | 1.71719684065423 | -0.37285453937766 |
| N | -1.72562831056294 | 0.05845982921177 | -0.41577460104786 |
| N | -2.31692722990475 | 2.12396061723938 | 0.54250138150138 |
| C | 1.44212955851482 | -1.30895811269110 | -0.06413369677704 |
| C | 3.05117898091408 | 0.51465222599969 | -0.09087028023176 |
| H | 3.03245564682307 | 1.00570696053227 | 0.88901490779563 |
| H | 3.27057164676994 | 1.27860850746888 | -0.83640479199078 |
| C | 4.10888326013599 | -0.57754202398457 | -0.11179459607601 |
| H | 4.22160893020297 | -0.95237944127822 | -1.13136514554002 |
| H | 5.07309766532458 | -0.18174936478785 | 0.20683785001943 |
| C | 3.69361339089068 | -1.71068837085828 | 0.80963720666265 |
| H | 4.33271977648928 | -2.58149978448418 | 0.65584998019057 |
| H | 3.78737830180561 | -1.41574202921265 | 1.86004730563694 |
| C | 1.94243003003729 | -3.48713581747287 | 0.93961027043632 |
| H | 2.44237159035390 | -3.70300333201000 | 1.88518704253859 |
| H | 2.32464641223946 | -4.19525765689253 | 0.19685035795986 |
| C | 0.43750372907864 | -3.62421258400772 | 1.09020350856506 |
| H | 0.10346647272685 | -3.07709424854781 | 1.97485824843911 |
| H | 0.17875597155341 | -4.67365586370679 | 1.22977417256800 |
| C | -0.25376710619074 | -3.06570995769477 | -0.14277068450122 |
| H | -0.04709798054048 | -3.69360455567509 | -1.01689019165661 |
| H | -1.33462210963032 | -3.05223170590890 | -0.00328140478463 |
| C | -1.42951104402664 | 1.32370570426123 | -0.06433525330491 |
| C | 0.23454782710840 | 3.09295680209842 | -0.13606917800774 |
| H | 1.31592958825831 | 3.09909481506254 | -0.00388203619005 |
| H | -0.11382182693686 | 3.09843202788079 | 1.98254531190676 |
| C | -0.45947889430757 | 3.64153949184266 | 1.09985541160123 |
| H | 0.01205520258550 | 3.71246628387787 | -1.01217180420030 |
| H | -0.21495895899657 | 4.69432009980278 | 1.24016423703752 |
| C | -1.96333035155998 | 3.48468257420293 | 0.95639245633219 |
| H | -2.46197344369892 | 3.68005838678166 | 1.90729287413146 |
| H | -2.36025661463973 | 4.19628726875896 | 0.22502570002000 |

| | | | |
|---|---|---|---|
| C | -3.69499354836160 | 1.69452498593721 | 0.79645604385079 |
| H | -4.34361045148049 | 2.55599155821972 | 0.62955183605994 |
| H | -3.79454490673774 | 1.40862166277474 | 1.84892955053915 |
| C | -4.09294606463240 | 0.54754998615530 | -0.11580130156719 |
| H | -4.21065570475309 | 0.91063511993620 | -1.13893021634999 |
| H | -5.05117262123261 | 0.14064266231677 | 0.20682822666822 |
| C | -3.01879912746627 | -0.52839105521410 | -0.08532874083668 |
| H | -2.98513753927386 | -1.00442576507871 | 0.90165817832163 |
| H | -3.23445364430430 | -1.30647536025242 | -0.81794227548165 |
| H | 1.10581001022824 | 1.22034300206150 | -2.16357075868159 |
| H | -0.98282665444971 | -1.08470028496240 | -2.25383456428222 |
| B | -0.59821072855847 | -0.64863112277283 | -1.12857622035541 |
| B | 0.64832057804078 | 0.70432800555924 | -1.16156121610945 |
| H | -0.77663933481763 | 0.67472063050296 | -3.25880442032718 |
| H | 0.75267628762689 | -0.71383227990127 | -3.25221438053758 |
| B | -0.05892350978060 | -0.06846048678024 | -2.67368074282379 |

**[(dcpe){B(*μ*-hpp)}$_2$]$^{2+}$**

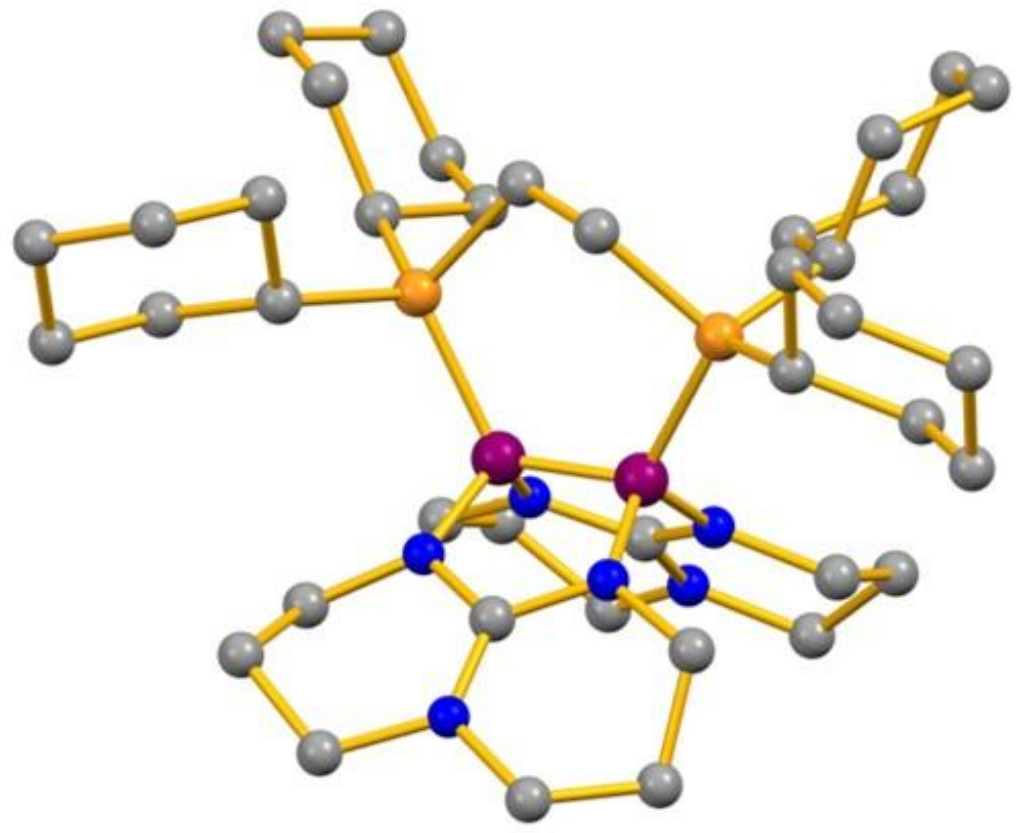

Energy: -2627.947426018899 H
xyz, charge: 2, multiplicity: 1

<u>Cartesian coordinates (in Å)</u>

| | | | |
|---|---|---|---|
| P | 7.15683017403170 | 12.52819937210898 | 16.94221485568410 |
| P | 9.52045683702722 | 11.31584193655950 | 14.39153415487916 |
| N | 5.54008165901166 | 10.67664362007006 | 15.23194221649136 |
| N | 7.01896242152076 | 9.89148212693560 | 13.69330085673806 |
| N | 4.72301918652786 | 9.91688116766609 | 13.15292279611808 |
| N | 6.95174935866474 | 9.59617287068017 | 17.10717461494596 |
| N | 8.46281483801985 | 8.84718311748166 | 15.57581721131968 |
| N | 7.87993121855893 | 7.45064519045903 | 17.38377819285444 |
| C | 5.73435106445999 | 10.14862386950834 | 14.00641152496792 |
| C | 4.18436871461716 | 10.82085568597808 | 15.76064694069727 |
| H | 4.18874581147220 | 11.62345267611240 | 16.49611451032684 |
| H | 3.88734171873830 | 9.90858281221021 | 16.28801483555918 |
| C | 3.18983652784300 | 11.11482735961307 | 14.65187352810855 |
| H | 2.17352187993241 | 11.10797729699124 | 15.04549325712647 |
| H | 3.37408277568262 | 12.10478291391225 | 14.23200065474433 |
| C | 3.32368525397560 | 10.06598598806461 | 13.56582011185516 |
| H | 2.93866505377513 | 9.09897876944528 | 13.90703397349353 |
| H | 2.75087858547588 | 10.35008059959161 | 12.68343472282008 |
| C | 4.95197614041245 | 9.27871651397134 | 11.85280780659309 |
| H | 4.20610827293925 | 9.67029639772752 | 11.16137743462472 |

| | | | |
|---|---|---|---|
| H | 4.78406437771836 | 8.19959783107441 | 11.93743413560830 |
| C | 6.35269479151635 | 9.56978962672383 | 11.35264895132826 |
| H | 6.43926754592958 | 10.62622664869065 | 11.09131283376228 |
| H | 6.55114727858066 | 8.98757335842356 | 10.45304293160810 |
| C | 7.35298413956248 | 9.22225309792349 | 12.43983504283286 |
| H | 7.38081086682595 | 8.13633581408753 | 12.58651659819995 |
| H | 8.35580056425842 | 9.52885045885439 | 12.14637390503892 |
| C | 7.77594944858860 | 8.60538862222703 | 16.70900001529896 |
| C | 6.00150965302181 | 9.29232309883211 | 18.17348356349903 |
| H | 5.16076141458399 | 8.70694665823649 | 17.78310315788815 |
| H | 5.59271674812335 | 10.22478746517115 | 18.54699127220088 |
| C | 6.69817144017430 | 8.51202014326545 | 19.27446582006662 |
| H | 7.50996701957746 | 9.11211611096309 | 19.68974855407723 |
| H | 6.00953148085337 | 8.27980314484125 | 20.08651285607105 |
| C | 7.24018799910563 | 7.22427185640349 | 18.68619060300524 |
| H | 7.98193694927869 | 6.77246390377279 | 19.34800941645549 |
| H | 6.43606427312042 | 6.49447267788706 | 18.55220172722497 |
| C | 8.58808505605830 | 6.29096188179205 | 16.83047281306381 |
| H | 8.01454216601729 | 5.40186737499544 | 17.09691229913355 |
| H | 9.56969936881952 | 6.20145001586280 | 17.30582815608273 |
| C | 8.72533674332134 | 6.41927469531739 | 15.32718498557150 |
| H | 7.74892448606895 | 6.30673502913498 | 14.84929188333674 |
| H | 9.37652046559800 | 5.63237197124638 | 14.94726025186700 |
| C | 9.30406576694160 | 7.78704561804783 | 15.01445607419904 |
| H | 10.31817715016366 | 7.84094719721473 | 15.42170027009117 |
| H | 9.37927398668448 | 7.94273642558782 | 13.94199147657176 |
| C | 7.56386225839450 | 12.30310423880594 | 18.74122898570088 |
| H | 7.84422137041575 | 11.24742193648777 | 18.79370928954943 |
| C | 6.33986967975313 | 12.51374629448720 | 19.65588456661313 |
| H | 6.06083752678629 | 13.56823496928471 | 19.65243582823840 |
| H | 5.46661700630486 | 11.96995955456273 | 19.29210961084694 |
| C | 6.66010943559618 | 12.09924084894951 | 21.09360927458572 |
| H | 5.78511121362023 | 12.26890626807348 | 21.72354151305246 |
| H | 6.86753290354823 | 11.02456279477829 | 21.12777714463760 |
| C | 7.86363890800074 | 12.87184605988629 | 21.63171218818441 |
| H | 7.60268419189651 | 13.93200416787548 | 21.71510626339610 |
| H | 8.10971499703257 | 12.53348635674294 | 22.63928895895588 |
| C | 9.07627237278142 | 12.71908784665545 | 20.71525870496290 |
| H | 9.41727737089279 | 11.67779477203842 | 20.73151129318573 |
| H | 9.90821545207263 | 13.32387147981270 | 21.08017779619905 |
| C | 8.75874059455978 | 13.11433322423621 | 19.26891768534110 |
| H | 9.64378586582396 | 12.95004630521069 | 18.65242104975151 |
| H | 8.53475830383527 | 14.18353526109660 | 19.22494295780710 |
| C | 5.85087299234870 | 13.82426323650573 | 16.79103141251113 |
| H | 5.00305895758352 | 13.42840184836293 | 17.35839368104570 |
| C | 6.23534029801423 | 15.18402752595046 | 17.40468367404564 |
| H | 6.59755738667285 | 15.07729187188968 | 18.42549771979374 |
| H | 7.05098337577546 | 15.62410702896164 | 16.82414515418625 |
| C | 4.47922715304987 | 16.31254357158911 | 15.96364746514130 |
| H | 3.58160842476713 | 16.93208660478677 | 15.98769330423393 |
| H | 5.21406367952349 | 16.85342250776677 | 15.35783251589294 |
| C | 4.17739645671695 | 14.96898357490904 | 15.29859559872925 |
| H | 3.86495881300082 | 15.12079175914589 | 14.26322342638001 |
| H | 3.34037018813123 | 14.48227240110883 | 15.81131869835358 |
| C | 5.39744826780459 | 14.04583248471169 | 15.33814884263093 |
| H | 6.20893134182072 | 14.52108695064662 | 14.78147144687954 |

| | | | |
|---|---|---|---|
| H | 5.19277612823278 | 13.10239217088913 | 14.83850222146784 |
| C | 8.60506329966372 | 13.34192468792632 | 16.16666812788969 |
| H | 8.23847082254565 | 13.86033895041085 | 15.28501379723448 |
| H | 8.94058814935628 | 14.11710182464514 | 16.85381467392540 |
| C | 9.78551615807078 | 12.44509351178068 | 15.80599197648291 |
| H | 10.07741923324783 | 11.81371684724432 | 16.64920691001261 |
| H | 10.64726737395792 | 13.07060883464925 | 15.57569307805424 |
| C | 11.14535568414963 | 10.43639402932536 | 14.22652484883125 |
| H | 11.04583834472180 | 9.62676888187299 | 14.95256744294539 |
| C | 12.40742253771980 | 11.21902829047037 | 14.63533920663113 |
| H | 12.56308293252047 | 12.07059037595538 | 13.97081948117402 |
| H | 12.30503626095944 | 11.61765055795933 | 15.64472758308596 |
| C | 13.63812847439767 | 10.30658701666747 | 14.58232789663683 |
| H | 14.52439954091143 | 10.88491961936184 | 14.84872727034092 |
| H | 13.53616215734550 | 9.52481745701377 | 15.34323169174984 |
| C | 13.81364593429665 | 9.66066954255928 | 13.20962608512246 |
| H | 14.03027633450964 | 10.43603184942761 | 12.46716938421284 |
| H | 14.67359536275543 | 8.98940406345275 | 13.21677782663686 |
| C | 12.55557675430389 | 8.90102224465072 | 12.79325909243590 |
| H | 12.40196516826693 | 8.04985877314939 | 13.46588022924025 |
| H | 12.67064042677858 | 8.48955667143466 | 11.78892012783117 |
| C | 5.03006886093896 | 16.13035619601433 | 17.37818822709336 |
| H | 4.24741821803245 | 15.72685906576507 | 18.02937870841970 |
| H | 5.31735819705981 | 17.09521303654379 | 17.79937720840261 |
| C | 11.32206825207665 | 9.80597735291064 | 12.83503699511282 |
| H | 10.43601300497548 | 9.23592888585679 | 12.54780470624035 |
| H | 11.43804583246806 | 10.59820271720470 | 12.09075836971348 |
| C | 9.24833491018241 | 12.30830480446913 | 12.85956328414638 |
| H | 9.32104111441842 | 11.54523750156887 | 12.07660680022389 |
| C | 7.85611937426959 | 12.95044057570619 | 12.73157616587826 |
| H | 7.07118230489145 | 12.23526844100757 | 12.96462187900332 |
| H | 7.76603696199160 | 13.76779489223394 | 13.44726998860562 |
| C | 7.66587005373070 | 13.53149962191834 | 11.32887269545179 |
| H | 7.70436344917084 | 12.72149379105353 | 10.59195730086356 |
| H | 6.67308664774294 | 13.97977699379122 | 11.25042060258328 |
| C | 8.74568254411540 | 14.56536560841340 | 11.01072532084854 |
| H | 8.62151060211266 | 15.42849155637277 | 11.67382880409241 |
| H | 8.62616248317131 | 14.93755835525267 | 9.99224023764945 |
| C | 10.14533203724433 | 13.98130877163327 | 11.19522182170144 |
| H | 10.90289796480830 | 14.75056013017764 | 11.03684782333636 |
| H | 10.32148884137299 | 13.21087251966120 | 10.43695881449990 |
| C | 10.33632184892759 | 13.35899021716087 | 12.58284896135675 |
| H | 11.32994562372563 | 12.91982608265660 | 12.64555231886805 |
| H | 10.28643442447309 | 14.14233503482859 | 13.34599714957932 |
| B | 6.83613137589766 | 10.73133732109635 | 16.06418642240674 |
| B | 7.98229253378542 | 10.14090956887084 | 14.87047059711513 |

**[(PCy3)(H){B(μ-hpp)}2]+**

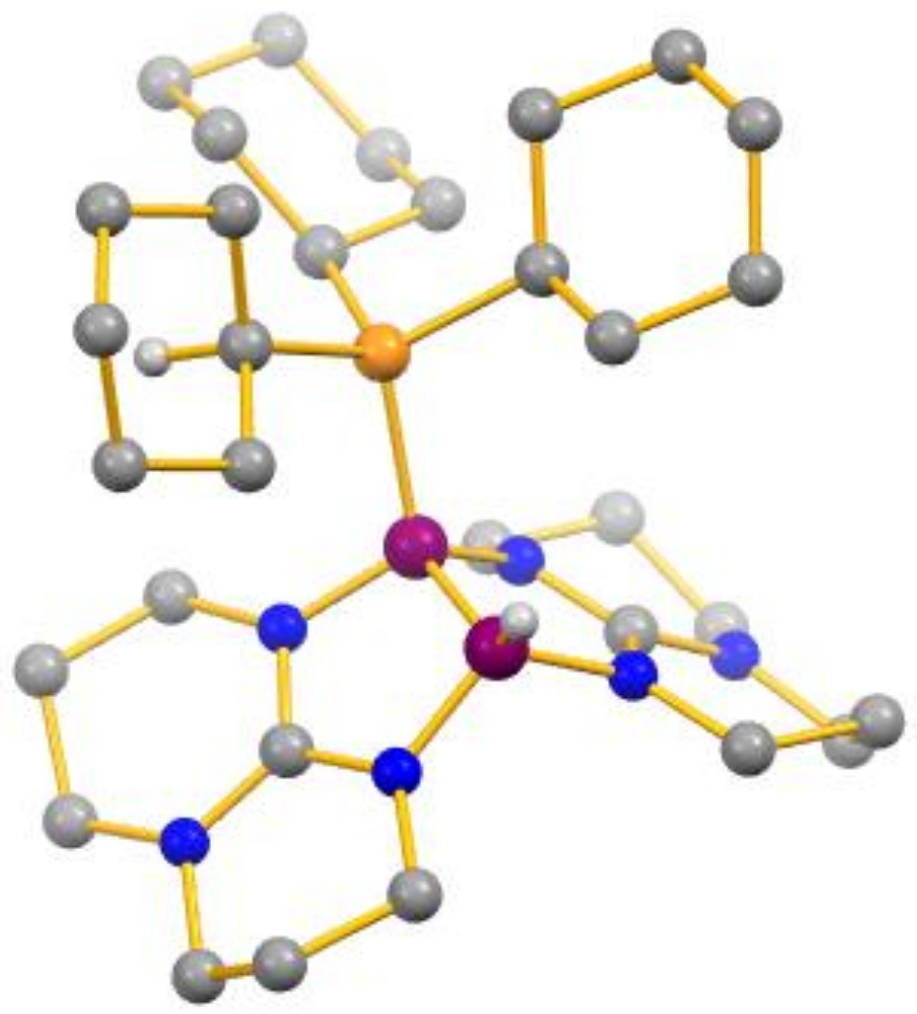

Energy: -1973.49900012992 H
xyz, charge: 1, multiplicity: 1

Cartesian coordinates (in Å)

| | | | |
|---|---|---|---|
| N | 8.5077482 | 14.8853512 | 5.2016475 |
| N | 7.9999533 | 12.7181295 | 5.6386768 |
| N | 6.9520290 | 13.7661646 | 3.8071550 |
| N | 8.9458237 | 15.4308898 | 7.7117803 |
| N | 8.0824493 | 13.3815139 | 8.1559087 |
| N | 7.6230565 | 15.1304639 | 9.6617050 |
| B | 9.4075820 | 14.6463817 | 6.4510478 |
| B | 8.9082430 | 13.0287354 | 6.8714015 |
| H | 9.6008539 | 12.0715922 | 7.1198035 |
| C | 7.7955859 | 13.7811731 | 4.8679293 |
| C | 8.0442038 | 16.1781587 | 4.7107486 |
| H | 8.8441368 | 16.9051740 | 4.8283070 |
| H | 7.2031614 | 16.5381437 | 5.3192810 |
| C | 7.6162698 | 16.0734023 | 3.2550109 |
| H | 8.4804441 | 15.8194911 | 2.6370700 |
| H | 7.2182292 | 17.0240832 | 2.8968502 |
| C | 6.5554512 | 14.9936683 | 3.1182937 |
| H | 5.5990485 | 15.3472108 | 3.5245440 |
| H | 6.3877457 | 14.7506399 | 2.0671856 |
| C | 7.3595218 | 11.4365115 | 5.3830917 |
| H | 6.4968127 | 11.3146179 | 6.0493944 |
| H | 8.0709829 | 10.6494791 | 5.6388582 |
| C | 6.9166299 | 11.3233641 | 3.9322497 |
| H | 6.2932650 | 10.4398589 | 3.7899425 |
| H | 7.7908545 | 11.2230656 | 3.2840491 |
| C | 6.1451400 | 12.5741561 | 3.5429170 |
| H | 5.9072221 | 12.5667365 | 2.4780848 |
| H | 5.1951899 | 12.6302837 | 4.0897346 |
| C | 8.1927011 | 14.6532701 | 8.5275286 |
| C | 9.0606895 | 16.8625852 | 7.9668847 |
| H | 8.2995387 | 17.4203108 | 7.4059986 |
| H | 10.0293761 | 17.2032928 | 7.6054380 |
| C | 8.9377178 | 17.1737465 | 9.4498966 |

| | | | |
|---|---|---|---|
| H | 9.7971315 | 16.7609066 | 9.9845946 |
| H | 8.9265862 | 18.2524121 | 9.6122424 |
| C | 7.6624944 | 16.5521588 | 9.9887144 |
| H | 7.6155829 | 16.6471012 | 11.0746695 |
| H | 6.7818467 | 17.0638495 | 9.5793080 |
| C | 6.7422192 | 14.2704737 | 10.4516779 |
| H | 6.7443879 | 14.6468983 | 11.4753864 |
| H | 5.7116599 | 14.3371595 | 10.0807666 |
| C | 7.2378330 | 12.8343182 | 10.4040545 |
| H | 6.5496865 | 12.1814669 | 10.9425262 |
| H | 8.2114543 | 12.7739795 | 10.8967134 |
| C | 7.3646361 | 12.3963171 | 8.9528790 |
| H | 7.9077400 | 11.4526256 | 8.8762688 |
| H | 6.3693592 | 12.2291269 | 8.5236396 |
| P | 11.3387990 | 14.9315022 | 6.0324418 |
| C | 12.3167771 | 14.7919940 | 7.6068242 |
| C | 11.7598254 | 13.7475485 | 8.5960448 |
| C | 13.8339467 | 14.5748873 | 7.4455987 |
| H | 12.1616857 | 15.7808466 | 8.0597768 |
| C | 12.4732943 | 13.8505060 | 9.9489155 |
| H | 11.8962722 | 12.7459115 | 8.1807300 |
| H | 10.6918189 | 13.8767790 | 8.7376704 |
| C | 14.5486255 | 14.6983315 | 8.7959023 |
| H | 14.0007985 | 13.5686257 | 7.0521259 |
| H | 14.2702893 | 15.2687655 | 6.7301778 |
| C | 13.9896552 | 13.7048316 | 9.8154656 |
| H | 12.0758655 | 13.0903722 | 10.6262118 |
| H | 12.2408728 | 14.8229143 | 10.4004477 |
| H | 15.6207289 | 14.5407084 | 8.6566485 |
| H | 14.4296919 | 15.7203823 | 9.1743584 |
| H | 14.4731540 | 13.8417333 | 10.7853373 |
| H | 14.2253238 | 12.6857298 | 9.4887549 |
| C | 11.6581847 | 16.6474076 | 5.3324570 |
| C | 12.9921670 | 17.3477142 | 5.6491737 |
| C | 11.3585176 | 16.7454636 | 3.8218007 |
| H | 10.8849486 | 17.2347860 | 5.8365439 |
| C | 12.9447320 | 18.8131122 | 5.1945653 |
| H | 13.8143742 | 16.8389523 | 5.1399508 |
| H | 13.2094851 | 17.3106906 | 6.7176854 |
| C | 11.2935102 | 18.2101933 | 3.3781765 |
| H | 12.1460409 | 16.2461334 | 3.2556229 |
| H | 10.4324662 | 16.2308917 | 3.5710966 |
| C | 12.5950815 | 18.9437224 | 3.7105981 |
| H | 13.9050231 | 19.2906257 | 5.4023223 |
| H | 12.1959036 | 19.3466339 | 5.7917802 |
| H | 11.0907376 | 18.2610161 | 2.3056602 |
| H | 10.4544751 | 18.7067118 | 3.8802075 |
| H | 12.5197309 | 19.9970394 | 3.4319429 |
| H | 13.4072980 | 18.5191240 | 3.1099436 |
| C | 11.9013227 | 13.7436112 | 4.7208148 |
| C | 13.3287239 | 13.9512686 | 4.1713347 |
| C | 11.7014347 | 12.2569151 | 5.0655658 |
| H | 11.1957521 | 13.9810487 | 3.9140989 |
| C | 13.5453939 | 13.0986036 | 2.9155975 |
| H | 14.0556686 | 13.6494698 | 4.9258068 |
| H | 13.5317033 | 14.9963491 | 3.9469618 |

| | | | |
|---|---|---|---|
| C | 11.9392413 | 11.3807156 | 3.8297430 |
| H | 12.4026259 | 11.9706699 | 5.8563503 |
| H | 10.7045324 | 12.0814664 | 5.4553006 |
| C | 13.3168123 | 11.6143835 | 3.2079202 |
| H | 14.5562500 | 13.2608287 | 2.5338553 |
| H | 12.8564619 | 13.4296173 | 2.1296423 |
| H | 11.8173755 | 10.3293843 | 4.1019004 |
| H | 11.1633945 | 11.6027198 | 3.0868631 |
| H | 13.4280437 | 11.0250125 | 2.2948750 |
| H | 14.0889665 | 11.2643166 | 3.9022422 |

**$PCy_3$**

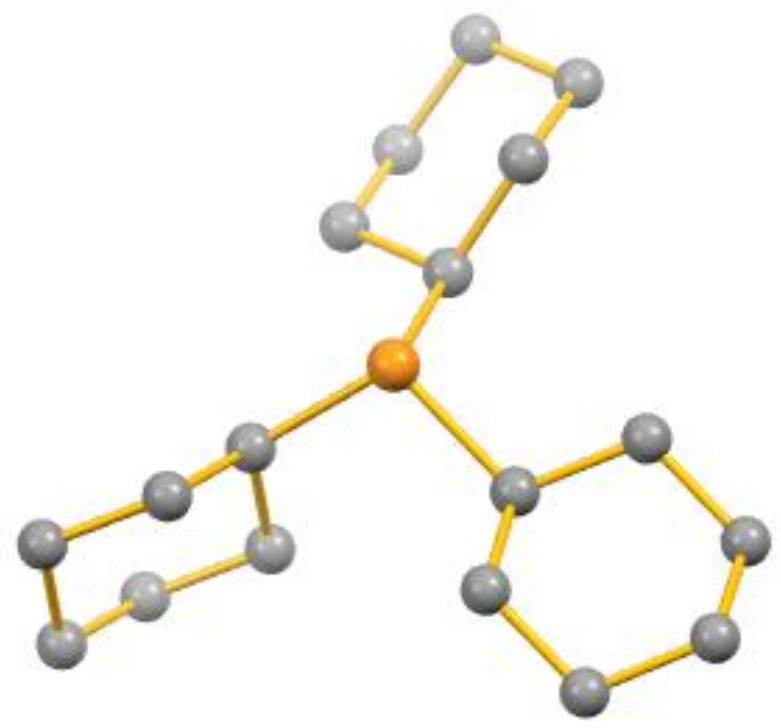

Energy: -1046.94011042941 H
xyz, charge: 0, multiplicity: 1

Cartesian coordinates (in Å)

| | | | |
|---|---|---|---|
| C | -1.1384790 | 4.1218434 | 1.3466911 |
| C | 0.1546507 | 3.4998171 | 1.9001761 |
| C | -0.9843502 | 5.6569577 | 1.3168620 |
| H | -1.9478608 | 3.8680080 | 2.0427191 |
| C | 0.5128074 | 4.0705269 | 3.2791084 |
| H | 0.9802884 | 3.6944097 | 1.2049313 |
| H | 0.0628970 | 2.4161990 | 1.9828674 |
| C | -0.6461285 | 6.2304660 | 2.6960267 |
| H | -0.1828499 | 5.9090430 | 0.6132540 |
| H | -1.8855318 | 6.1316889 | 0.9258796 |
| C | 0.6260189 | 5.5957628 | 3.2605898 |
| H | 1.4470100 | 3.6233026 | 3.6310439 |
| H | -0.2635713 | 3.7765588 | 3.9954771 |
| H | -0.5341902 | 7.3165200 | 2.6310900 |
| H | -1.4812160 | 6.0415957 | 3.3812729 |
| H | 0.8315674 | 5.9739031 | 4.2659053 |
| H | 1.4784579 | 5.8858677 | 2.6350514 |
| C | -1.0508469 | 1.7505916 | -0.4175254 |
| C | -1.7406867 | 0.7428279 | 0.5137901 |
| C | -0.9653745 | 1.1961111 | -1.8525051 |
| H | -0.0129457 | 1.8520085 | -0.0813669 |
| C | -1.0341617 | -0.6181084 | 0.4869695 |
| H | -2.7794436 | 0.6038188 | 0.1981292 |
| H | -1.7783401 | 1.1258584 | 1.5369973 |
| C | -0.2619090 | -0.1662113 | -1.8775719 |

| | | | |
|---|---|---|---|
| H | -1.9674969 | 1.0836882 | -2.2759477 |
| H | -0.4385107 | 1.9071773 | -2.4935028 |
| C | -0.9399421 | -1.1683012 | -0.9388300 |
| H | -1.5595117 | -1.3264503 | 1.1342084 |
| H | -0.0239245 | -0.5065555 | 0.8983955 |
| H | -0.2404379 | -0.5571668 | -2.8988040 |
| H | 0.7825155 | -0.0357477 | -1.5699072 |
| H | -0.4007908 | -2.1196662 | -0.9442132 |
| H | -1.9507877 | -1.3789934 | -1.3083333 |
| C | -3.5161654 | 3.3440906 | -0.1103034 |
| C | -4.1800722 | 4.6860159 | 0.2475018 |
| C | -4.1943792 | 2.7368699 | -1.3507036 |
| H | -3.6706475 | 2.6641695 | 0.7357361 |
| C | -5.6983788 | 4.5522836 | 0.4151010 |
| H | -3.9662849 | 5.4168552 | -0.5413985 |
| H | -3.7576759 | 5.0870068 | 1.1699381 |
| C | -5.7119039 | 2.6068986 | -1.1785081 |
| H | -3.9808114 | 3.3701918 | -2.2194797 |
| H | -3.7748728 | 1.7554476 | -1.5734055 |
| C | -6.3550376 | 3.9487092 | -0.8268020 |
| H | -6.1314766 | 5.5312705 | 0.6397152 |
| H | -5.9079542 | 3.9112780 | 1.2796346 |
| H | -6.1538097 | 2.1981191 | -2.0916814 |
| H | -5.9220608 | 1.8863838 | -0.3789968 |
| H | -7.4306921 | 3.8288073 | -0.6705419 |
| H | -6.2340472 | 4.6385524 | -1.6703910 |
| P | -1.6596463 | 3.5314737 | -0.3779837 |

**[HB($\mu$-hpp)$_2$B]$^+$**

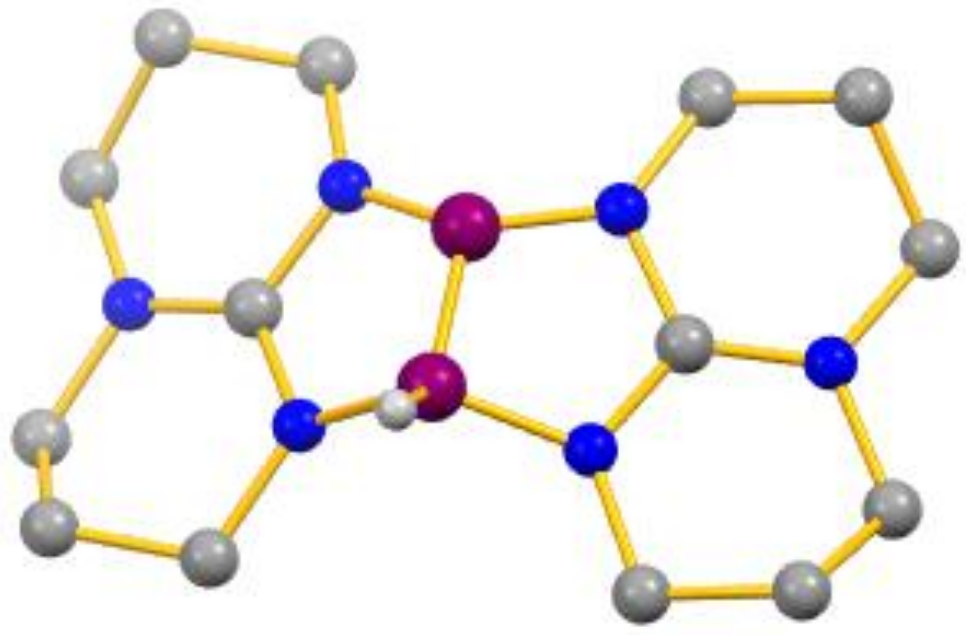

Energy: -926.4984612528 H
xyz, charge: 1, multiplicity: 1

Cartesian coordinates (in Å)

| | | | |
|---|---|---|---|
| N | 15.7112779 | 17.3951430 | 1.8780627 |
| N | 17.6817173 | 18.5283107 | 2.2387517 |
| N | 15.7688902 | 19.7521337 | 1.5950348 |
| N | 16.9711136 | 15.1083570 | 1.6731907 |
| N | 19.0088161 | 16.1193399 | 2.0234042 |
| N | 18.9609385 | 13.9574952 | 1.0772836 |
| B | 16.6622377 | 16.3858170 | 2.2531861 |
| B | 18.0493814 | 17.1008501 | 2.8029128 |
| H | 18.4283452 | 17.2073675 | 3.9444737 |
| C | 16.3933764 | 18.6081596 | 1.9074917 |
| C | 14.4109900 | 17.3092844 | 1.2097733 |
| H | 13.9025974 | 16.4166940 | 1.5706088 |
| H | 14.5452222 | 17.2036532 | 0.1270185 |
| C | 13.6035096 | 18.5579809 | 1.5253575 |
| H | 13.3989530 | 18.5953243 | 2.5975955 |
| H | 12.6469543 | 18.5403564 | 1.0022220 |
| C | 14.3866442 | 19.7903594 | 1.1024844 |
| H | 14.3997854 | 19.8813564 | 0.0101442 |
| H | 13.9199370 | 20.6934682 | 1.4983077 |
| C | 18.5015304 | 19.7313643 | 2.3941304 |
| H | 19.1472831 | 19.8346552 | 1.5160806 |
| H | 19.1493807 | 19.5775519 | 3.2577209 |
| C | 17.6466935 | 20.9776444 | 2.5745923 |
| H | 18.2600896 | 21.8747526 | 2.4857592 |
| H | 17.1970729 | 20.9794521 | 3.5702953 |
| C | 16.5431732 | 20.9972373 | 1.5286617 |
| H | 15.8529855 | 21.8217466 | 1.7071203 |
| H | 16.9558531 | 21.1230198 | 0.5214185 |
| C | 18.3588788 | 15.0402819 | 1.5885387 |
| C | 16.1505161 | 14.1523026 | 0.9267493 |
| H | 16.0533704 | 14.4679452 | -0.1184504 |
| H | 15.1543364 | 14.1447631 | 1.3660755 |
| C | 16.7890857 | 12.7751640 | 1.0083638 |
| H | 16.7904734 | 12.4378999 | 2.0472253 |
| H | 16.2211317 | 12.0521030 | 0.4222106 |
| C | 18.2136632 | 12.8430483 | 0.4821622 |
| H | 18.7510532 | 11.9229496 | 0.7159766 |
| H | 18.2157570 | 12.9543951 | -0.6083455 |

| | | | |
|---|---|---|---|
| C | 20.4179891 | 13.9631912 | 0.9004135 |
| H | 20.7532217 | 12.9261235 | 0.9125959 |
| H | 20.6699340 | 14.3810759 | -0.0807190 |
| C | 21.0728466 | 14.7580477 | 2.0191888 |
| H | 22.1493384 | 14.8143736 | 1.8552248 |
| H | 20.9074676 | 14.2437717 | 2.9687712 |
| C | 20.4715669 | 16.1551979 | 2.0747591 |
| H | 20.7550444 | 16.6626492 | 2.9973822 |
| H | 20.8394969 | 16.7629484 | 1.2417757 |

**Pyridine**

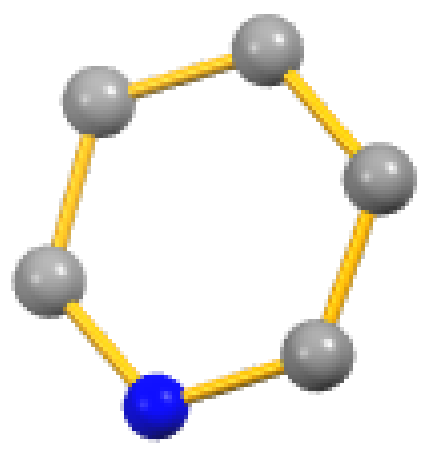

Energy: -248.2289514345 H
xyz, charge: 0, multiplicity: 1

Cartesian coordinates (in Å)

| | | | |
|---|---|---|---|
| C | -1.5006603 | -2.4157887 | 0.0012504 |
| C | -0.1116577 | -2.4036865 | 0.0017387 |
| C | 0.5415239 | -1.1749969 | 0.0010623 |
| C | -2.1747733 | -1.2011987 | 0.0002635 |
| H | -2.0461673 | -3.3514676 | 0.0016986 |
| H | 0.4581507 | -3.3240794 | 0.0025618 |
| H | 1.6261321 | -1.1314349 | 0.0013833 |
| H | -3.2564565 | -1.1592682 | -0.0000740 |
| N | -0.0940938 | -0.0024223 | -0.0000697 |
| C | -1.4276777 | -0.0272983 | -0.0003425 |
| H | -1.9244223 | 0.9378331 | -0.0011306 |

**Pyridine-$BH_3$**

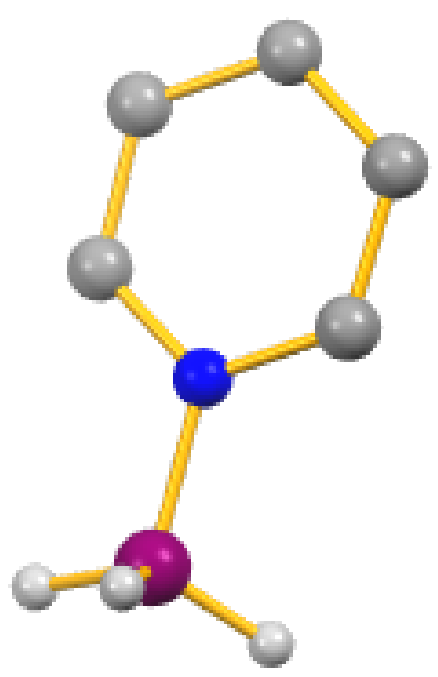

Energy: -274.8788238301 H
xyz, charge: 0, multiplicity: 1

Cartesian coordinates (in Å)

| | | | |
|---|---|---|---|
| C | -1.5045770 | -2.4212139 | 0.0051321 |
| C | -0.1141048 | -2.4082271 | 0.0014822 |
| C | 0.5466698 | -1.1944293 | -0.0075333 |
| C | -2.1797446 | -1.2091174 | -0.0010884 |
| H | -2.0480105 | -3.3570777 | 0.0151921 |
| H | 0.4579336 | -3.3256406 | 0.0085565 |
| H | 1.6246940 | -1.1192632 | -0.0072825 |
| H | -3.2599958 | -1.1649869 | 0.0038996 |
| C | -1.4505013 | -0.0309569 | -0.0103609 |
| H | -1.9195122 | 0.9420716 | -0.0125502 |
| H | 1.6258770 | 1.2409408 | 0.7250317 |
| H | 1.1355302 | 1.4382167 | -1.2114332 |
| H | -0.0530997 | 2.2445850 | 0.2043192 |
| B | 0.7236801 | 1.3614729 | -0.0728985 |
| N | -0.1102627 | -0.0230649 | -0.0157984 |

**[(Py)(H){B(*μ*-hpp)}$_2$]$^+$**

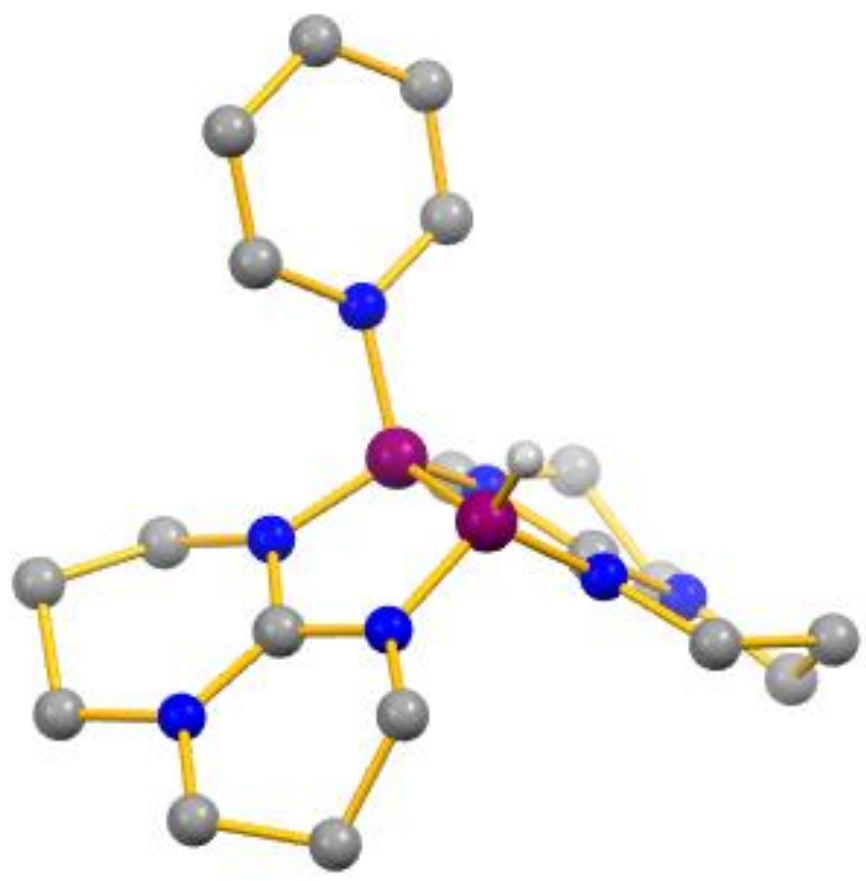

Energy: -1174.789616747 H
xyz, charge: +1, multiplicity: 1

Cartesian coordinates (in Å)

| | | | |
|---|---|---|---|
| N | 4.7578116 | 11.9798969 | 5.3627415 |
| N | 4.6224875 | 12.9285760 | 3.3060778 |
| N | 5.7662382 | 14.0875102 | 5.0071511 |
| N | 4.8406172 | 9.6267714 | 4.2615760 |
| N | 4.6774141 | 10.5674355 | 2.2044014 |
| N | 5.9406390 | 8.5902426 | 2.4395313 |
| B | 4.0126093 | 10.8774522 | 4.5943349 |
| B | 3.8368019 | 11.6031613 | 3.0268679 |
| C | 5.0656337 | 13.0258127 | 4.5564530 |
| C | 5.3423055 | 11.8957050 | 6.6935674 |
| H | 6.3448087 | 11.4504298 | 6.6570909 |
| H | 4.7222647 | 11.2320985 | 7.2991825 |
| C | 5.4142688 | 13.2833218 | 7.3159570 |
| H | 4.4018472 | 13.6724643 | 7.4531489 |
| H | 5.8913016 | 13.2483564 | 8.2961690 |
| C | 6.2014223 | 14.2067719 | 6.3996842 |

| | | | |
|---|---|---|---|
| H | 6.0718103 | 15.2481611 | 6.7040656 |
| H | 7.2730375 | 13.9834651 | 6.4609557 |
| C | 6.1488997 | 15.1867886 | 4.1153571 |
| H | 7.1623690 | 15.4957994 | 4.3824113 |
| H | 5.4888686 | 16.0416678 | 4.3000585 |
| C | 6.0852385 | 14.7677485 | 2.6546312 |
| H | 6.9158703 | 14.0947623 | 2.4268059 |
| H | 6.1859701 | 15.6483654 | 2.0191444 |
| C | 4.7697418 | 14.0485035 | 2.3877840 |
| H | 3.9257364 | 14.7416886 | 2.4901899 |
| H | 4.7381036 | 13.6588478 | 1.3711452 |
| C | 5.1754578 | 9.5845706 | 2.9477031 |
| C | 5.4830067 | 8.7372812 | 5.2208443 |
| H | 4.8118440 | 8.6092884 | 6.0730103 |
| H | 6.4049568 | 9.1884840 | 5.6090259 |
| C | 5.7962023 | 7.3939180 | 4.5795625 |
| H | 4.8653607 | 6.8754912 | 4.3349095 |
| H | 6.3595102 | 6.7607703 | 5.2662959 |
| C | 6.6012550 | 7.6186474 | 3.3096460 |
| H | 7.6137425 | 7.9632678 | 3.5558990 |
| H | 6.7045677 | 6.6867636 | 2.7513752 |
| C | 6.3658406 | 8.6487737 | 1.0403002 |
| H | 6.5669641 | 7.6279492 | 0.7128253 |
| H | 7.3034128 | 9.2118140 | 0.9526702 |
| C | 5.2752439 | 9.2824191 | 0.1905987 |
| H | 4.3981030 | 8.6307722 | 0.1871488 |
| H | 5.6172190 | 9.3854633 | -0.8399277 |
| C | 4.9069950 | 10.6416340 | 0.7678418 |
| H | 5.7058129 | 11.3645506 | 0.5611895 |
| H | 3.9972078 | 11.0283739 | 0.3055970 |
| H | 2.7845310 | 11.8319483 | 2.4749303 |
| C | 2.1284942 | 9.2500693 | 5.1819753 |
| C | 2.0447801 | 11.3484101 | 6.1611208 |
| C | 0.9186026 | 8.8993679 | 5.7478930 |
| H | 2.6789505 | 8.5830606 | 4.5360170 |
| C | 0.8326281 | 11.0608808 | 6.7568363 |
| H | 2.5295550 | 12.3062005 | 6.2725342 |
| C | 0.2523829 | 9.8148780 | 6.5532201 |
| H | 0.5074070 | 7.9190584 | 5.5516745 |
| H | 0.3525709 | 11.8117540 | 7.3686508 |
| H | -0.6976410 | 9.5655038 | 7.0065704 |
| N | 2.6920468 | 10.4570382 | 5.3852971 |

### ***N*,*N'*-Dimethylaminopyridine**

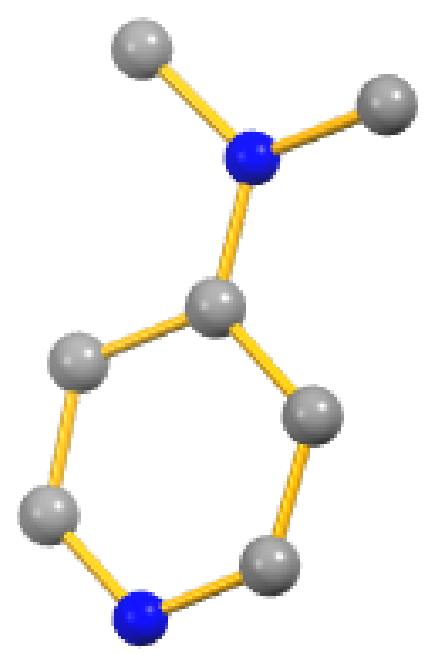

Energy: -382.1717538031 H
xyz, charge: 0, multiplicity: 1

<u>Cartesian coordinates (in Å)</u>

| | | | |
|---|---|---|---|
| C | -1.5576920 | -2.5850739 | 0.0387973 |
| C | -0.4407980 | -2.3619430 | -0.7922232 |
| C | 0.1636411 | -1.1156737 | -0.8024541 |
| C | -1.9841937 | -1.4772626 | 0.7994750 |
| H | -0.0418017 | -3.1397359 | -1.4259155 |
| H | 1.0249139 | -0.9527052 | -1.4437647 |
| H | -2.8378732 | -1.5370399 | 1.4578018 |
| N | -0.2333144 | -0.0687541 | -0.0756009 |
| C | -1.2967000 | -0.2785846 | 0.7035529 |
| H | -1.6304430 | 0.5693413 | 1.2945358 |
| C | -1.7977468 | -4.8666158 | -0.8012513 |
| H | -0.7421135 | -5.1231789 | -0.6792345 |
| H | -2.3812589 | -5.7572071 | -0.5799146 |
| H | -1.9637255 | -4.5998269 | -1.8523854 |
| C | -3.4156480 | -3.9391926 | 0.8652364 |
| H | -4.2200818 | -3.3039709 | 0.4740925 |
| H | -3.7487058 | -4.9735889 | 0.8246631 |
| H | -3.2609942 | -3.6815329 | 1.9163527 |
| N | -2.1871442 | -3.8021103 | 0.1049365 |

### **4-Dimethylaminopyridine-$BH_3$**

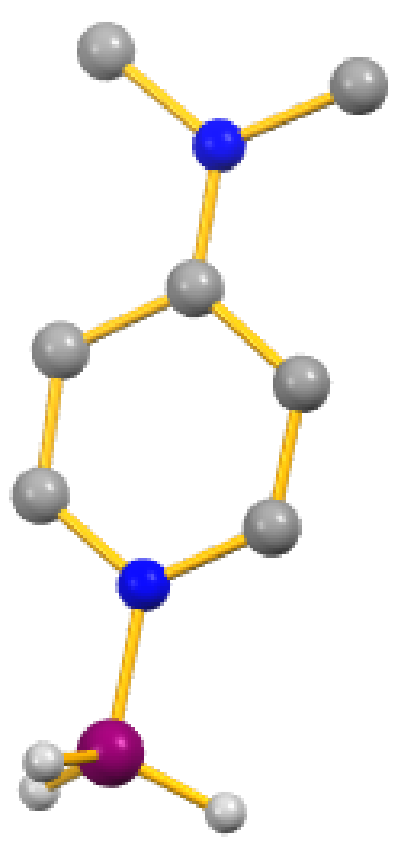

Energy: -408.8262630507 H
xyz, charge: 0, multiplicity: 1

Cartesian coordinates (in Å)

| | | | |
|---|---|---|---|
| C | -1.5768870 | -2.5592374 | 0.0052143 |
| C | -0.4359276 | -2.3500938 | 0.8105440 |
| C | 0.2074681 | -1.1333992 | 0.7936060 |
| C | -1.9818768 | -1.4565874 | -0.7820346 |
| H | -0.0455672 | -3.1237120 | 1.4527390 |
| H | 1.0808861 | -0.9511311 | 1.4032200 |
| H | -2.8434457 | -1.5082814 | -1.4288940 |
| C | -1.2758515 | -0.2787770 | -0.7327929 |
| H | -1.5691913 | 0.5774537 | -1.3238816 |
| C | -3.4188448 | -3.9111088 | -0.8540555 |
| H | -3.1767811 | -3.7689854 | -1.9118256 |
| H | -3.8076074 | -4.9182999 | -0.7310327 |
| H | -4.2122761 | -3.2068710 | -0.5851447 |
| C | -1.7949932 | -4.8490530 | 0.8195623 |
| H | -1.8167582 | -4.5891397 | 1.8824904 |
| H | -2.4511834 | -5.7018965 | 0.6684796 |
| H | -0.7766315 | -5.1556821 | 0.5614748 |
| N | -2.2464490 | -3.7439547 | -0.0103789 |
| H | 1.2811916 | 1.2739606 | -1.0217771 |
| H | -0.2110175 | 2.1758130 | -0.0213246 |
| H | 1.3138124 | 1.3205546 | 0.9928874 |
| B | 0.6227643 | 1.2959652 | -0.0013359 |
| N | -0.1898379 | -0.0992931 | 0.0372289 |

**[(DMAP)(H){B(*μ*-hpp)}$_2$]$^+$**

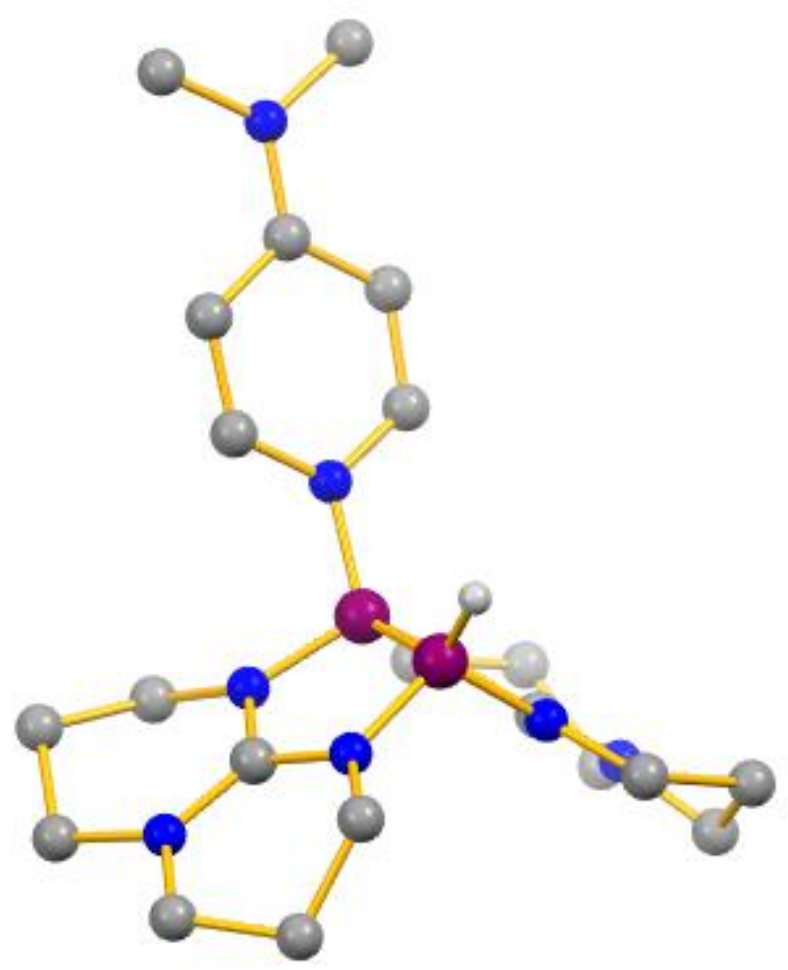

Energy: -1308.744568924 H
xyz, charge: +1, multiplicity: 1

Cartesian coordinates (in Å)

| | | | |
|---|---|---|---|
| N | 4.8662893 | 11.8266399 | 5.4785556 |
| N | 4.6100108 | 12.9567917 | 3.5282808 |
| N | 5.9249606 | 13.9243511 | 5.2265776 |
| N | 4.7807389 | 9.5808575 | 4.1769559 |

| | | | |
|---|---|---|---|
| N | 4.4943031 | 10.7039008 | 2.2279242 |
| N | 5.7050919 | 8.6796721 | 2.1923490 |
| B | 4.0114033 | 10.8195093 | 4.6811331 |
| B | 3.7517472 | 11.6839073 | 3.2040764 |
| C | 5.1499622 | 12.9276029 | 4.7435796 |
| C | 5.5455394 | 11.6089729 | 6.7462135 |
| H | 6.5271959 | 11.1410934 | 6.5941577 |
| H | 4.9500964 | 10.9114290 | 7.3380477 |
| C | 5.7131130 | 12.9332578 | 7.4787754 |
| H | 4.7276925 | 13.3378613 | 7.7248948 |
| H | 6.2595602 | 12.7986222 | 8.4132380 |
| C | 6.4633104 | 13.9101444 | 6.5869751 |
| H | 6.3927996 | 14.9245856 | 6.9874796 |
| H | 7.5283374 | 13.6517095 | 6.5495051 |
| C | 6.2824804 | 15.0835605 | 4.4047003 |
| H | 7.3250929 | 15.3345169 | 4.6156003 |
| H | 5.6735770 | 15.9414878 | 4.7122411 |
| C | 6.0908460 | 14.7974312 | 2.9232045 |
| H | 6.8737489 | 14.1192804 | 2.5737596 |
| H | 6.1784471 | 15.7268011 | 2.3589636 |
| C | 4.7309891 | 14.1482354 | 2.7026475 |
| H | 3.9264194 | 14.8584362 | 2.9318991 |
| H | 4.6046264 | 13.8527835 | 1.6618416 |
| C | 5.0132181 | 9.6468441 | 2.8442447 |
| C | 5.4634936 | 8.5943683 | 5.0021925 |
| H | 4.8546410 | 8.4099404 | 5.8898357 |
| H | 6.4266805 | 8.9851638 | 5.3552523 |
| C | 5.6853746 | 7.3049608 | 4.2260651 |
| H | 4.7226323 | 6.8342737 | 4.0096890 |
| H | 6.2782528 | 6.5994288 | 4.8099225 |
| C | 6.3999564 | 7.6215237 | 2.9219592 |
| H | 7.4380924 | 7.9192075 | 3.1195187 |
| H | 6.4345415 | 6.7398722 | 2.2794944 |
| C | 6.0341306 | 8.8562661 | 0.7782893 |
| H | 6.1814021 | 7.8653712 | 0.3462055 |
| H | 6.9801136 | 9.4027906 | 0.6733089 |
| C | 4.9071596 | 9.5913919 | 0.0695608 |
| H | 4.0120406 | 8.9645285 | 0.0727501 |
| H | 5.1786547 | 9.7791143 | -0.9700994 |
| C | 4.6234410 | 10.9009074 | 0.7915473 |
| H | 5.4278567 | 11.6195807 | 0.5899979 |
| H | 3.6957847 | 11.3507891 | 0.4333332 |
| H | 2.6760532 | 11.9996571 | 2.7453565 |
| C | 2.1500184 | 9.1752211 | 5.3356889 |
| C | 2.2101238 | 11.1721095 | 6.4774045 |
| C | 1.0041837 | 8.7878046 | 5.9791526 |
| H | 2.6068004 | 8.5485104 | 4.5840133 |
| C | 1.0669239 | 10.8663990 | 7.1676608 |
| H | 2.7137268 | 12.1160570 | 6.6235157 |
| C | 0.4031681 | 9.6337618 | 6.9447839 |
| H | 0.5757361 | 7.8324528 | 5.7211876 |
| H | 0.6893044 | 11.5916823 | 7.8706136 |
| N | 2.7745944 | 10.3481522 | 5.5702458 |
| C | -1.3203812 | 10.2073860 | 8.5700239 |
| H | -1.6126425 | 11.1507386 | 8.1005221 |
| H | -2.2101932 | 9.7509327 | 8.9928441 |

| | | | |
|---|---|---|---|
| H | -0.6289385 | 10.4223680 | 9.3889648 |
| C | -1.3855509 | 8.0214097 | 7.3219062 |
| H | -2.2633093 | 7.9282127 | 7.9541054 |
| H | -1.7094496 | 7.9607068 | 6.2792069 |
| H | -0.7249454 | 7.1766449 | 7.5338607 |
| N | -0.7262068 | 9.2923233 | 7.6016813 |
| H | 0.5074070 | 7.9190584 | 5.5516745 |
| H | 0.3525709 | 11.8117540 | 7.3686508 |
| H | -0.6976410 | 9.5655038 | 7.0065704 |
| N | 2.6920468 | 10.4570382 | 5.3852971 |

**4-Trifluormethylpyridine**

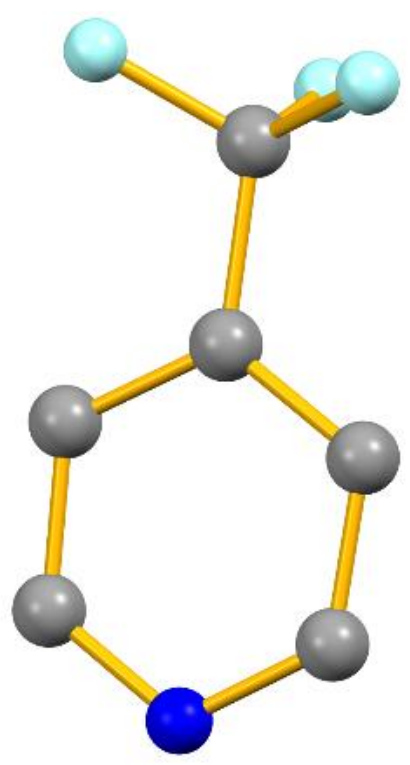

Energy: -585.3033403173 H
xyz, charge: 0, multiplicity: 1

Cartesian coordinates (in Å)

| | | | |
|---|---|---|---|
| C | -0.4488425 | -2.0229652 | 0.0085419 |
| C | 0.2946559 | -0.8480693 | -0.0132766 |
| C | -1.7786513 | 0.3483069 | -0.0118040 |
| C | -2.4236800 | -0.8836320 | 0.0097904 |
| H | 0.0531701 | -2.9845346 | 0.0146184 |
| H | 1.3753740 | -0.8769134 | -0.0288768 |
| H | -2.3445726 | 1.2694146 | -0.0262978 |
| H | -3.5074106 | -0.9301843 | 0.0167310 |
| C | 0.3645536 | 1.6648161 | -0.0022256 |
| F | -0.3275618 | 2.6585745 | -0.5919390 |
| F | 0.6187805 | 2.0584790 | 1.2659096 |
| F | 1.5526222 | 1.5706650 | -0.6304676 |
| C | -0.3903465 | 0.3597547 | -0.0245526 |
| N | -1.7814927 | -2.0514918 | 0.0203326 |

**4-Trifluormethylpyridine-$BH_3$**

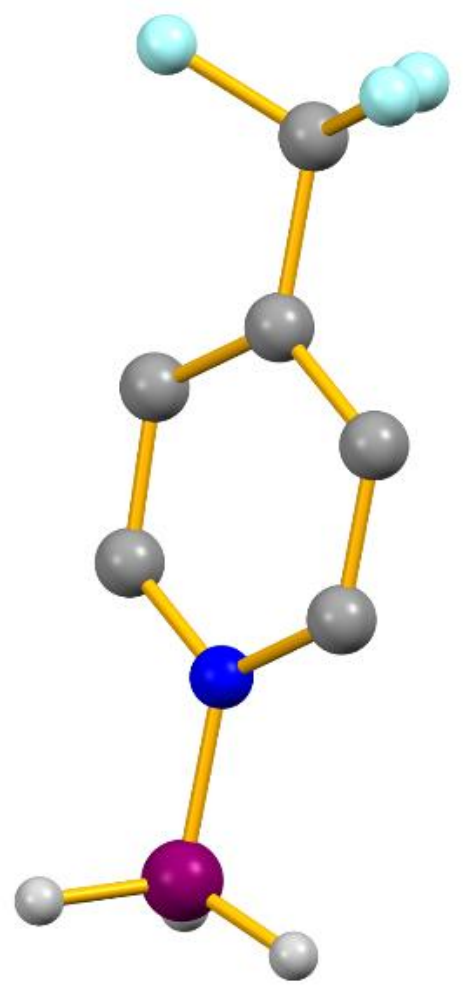

Energy: -611.95007110591 H
xyz, charge: 0, multiplicity: 1

Cartesian coordinates (in Å)

| | | | |
|---|---|---|---|
| C | -0.4420862 | -2.0143148 | 0.0117216 |
| C | 0.2862791 | -0.8425662 | -0.0091231 |
| C | -1.7817336 | 0.3625619 | -0.0174571 |
| C | -2.4424513 | -0.8555624 | 0.0033548 |
| H | 0.0276882 | -2.9871953 | 0.0238090 |
| H | 1.3662177 | -0.8770999 | -0.0191842 |
| H | -2.3470769 | 1.2828541 | -0.0328831 |
| H | -3.5199481 | -0.9272853 | 0.0088314 |
| C | 0.3736162 | 1.6686336 | -0.0023802 |
| F | -0.3576932 | 2.6938763 | -0.4720771 |
| F | 0.7476500 | 1.9830544 | 1.2553053 |
| F | 1.4944274 | 1.5898414 | -0.7433511 |
| C | -0.3960346 | 0.3697980 | -0.0242676 |
| H | -3.7385574 | -3.1832770 | 0.1800663 |
| H | -2.3383828 | -3.9201049 | -1.0752976 |
| H | -2.0764360 | -4.0856844 | 0.9052973 |
| N | -1.7856976 | -2.0222044 | 0.0168948 |
| B | -2.5703232 | -3.4360348 | 0.0115272 |

**[($CF_3$-Py)(H){B($\mu$-hpp)}$_2$]$^+$**

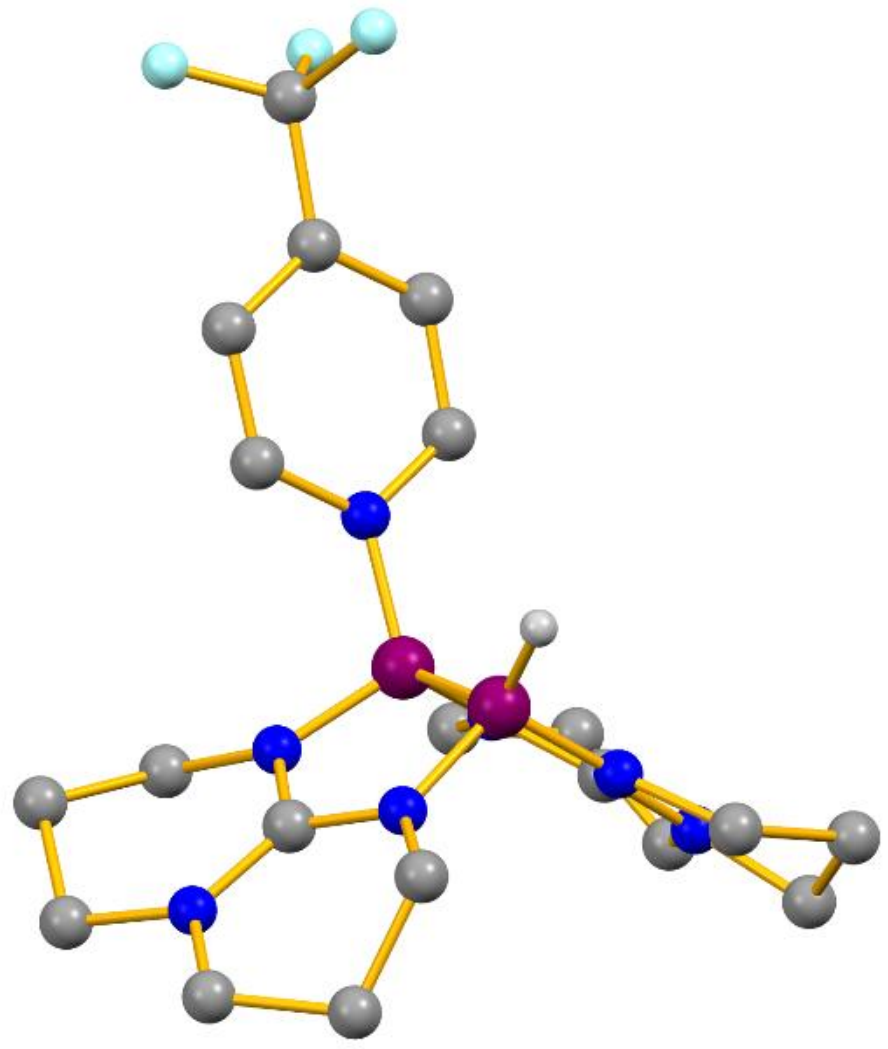

Energy: -1511.856918045 H
xyz, charge: +1, multiplicity: 1

Cartesian coordinates (in Å)

| | | | |
|---|---|---|---|
| N | 4.8143986 | 11.9565758 | 5.4060861 |
| N | 4.5942343 | 12.9162963 | 3.3617997 |
| N | 5.7827681 | 14.0790771 | 5.0287093 |
| N | 4.8859980 | 9.6101367 | 4.2842966 |
| N | 4.6346815 | 10.5624199 | 2.2411863 |
| N | 5.9259745 | 8.5975848 | 2.4143628 |
| B | 4.0621954 | 10.8489397 | 4.6576068 |
| B | 3.8165668 | 11.5845334 | 3.0993963 |
| C | 5.0808388 | 13.0121765 | 4.5960515 |
| C | 5.4480906 | 11.8702916 | 6.7147309 |
| H | 6.4534030 | 11.4368835 | 6.6385214 |
| H | 4.8582469 | 11.1954947 | 7.3380806 |
| C | 5.5262737 | 13.2547150 | 7.3433859 |
| H | 4.5151752 | 13.6308329 | 7.5209820 |
| H | 6.0394404 | 13.2189228 | 8.3050580 |
| C | 6.2679625 | 14.1938698 | 6.4054544 |
| H | 6.1372121 | 15.2313817 | 6.7218645 |
| H | 7.3435459 | 13.9831821 | 6.4248356 |
| C | 6.1175287 | 15.1901805 | 4.1314647 |
| H | 7.1355568 | 15.5113501 | 4.3641298 |
| H | 5.4526839 | 16.0338991 | 4.3468113 |
| C | 6.0067915 | 14.7801979 | 2.6710137 |
| H | 6.8381435 | 14.1210684 | 2.4081466 |
| H | 6.0718196 | 15.6665024 | 2.0388651 |
| C | 4.6927458 | 14.0443099 | 2.4459245 |
| H | 3.8430405 | 14.7241039 | 2.5833873 |
| H | 4.6305511 | 13.6606037 | 1.4284885 |
| C | 5.1712892 | 9.5796774 | 2.9574538 |
| C | 5.5741192 | 8.7209950 | 5.2125795 |
| H | 4.9379470 | 8.5809273 | 6.0894321 |
| H | 6.5059111 | 9.1789724 | 5.5674470 |

| | | | |
|---|---|---|---|
| C | 5.8742483 | 7.3848842 | 4.5502681 |
| H | 4.9395185 | 6.8589139 | 4.3388503 |
| H | 6.4695708 | 6.7528460 | 5.2103775 |
| C | 6.6271683 | 7.6247378 | 3.2514786 |
| H | 7.6457507 | 7.9757296 | 3.4598677 |
| H | 6.7152398 | 6.6975991 | 2.6829973 |
| C | 6.2926911 | 8.6664810 | 0.9986205 |
| H | 6.4892318 | 7.6490715 | 0.6583152 |
| H | 7.2207120 | 9.2383753 | 0.8760555 |
| C | 5.1623117 | 9.2938240 | 0.1976762 |
| H | 4.2919471 | 8.6337820 | 0.2260324 |
| H | 5.4609909 | 9.4054473 | -0.8452672 |
| C | 4.8049437 | 10.6466323 | 0.7962614 |
| H | 5.5877202 | 11.3783707 | 0.5620430 |
| H | 3.8734019 | 11.0266014 | 0.3735959 |
| H | 2.7388123 | 11.8018108 | 2.5952185 |
| C | 2.2089105 | 9.2028028 | 5.2785697 |
| C | 2.1336916 | 11.2938844 | 6.2747903 |
| C | 1.0183751 | 8.8380742 | 5.8699563 |
| H | 2.7487871 | 8.5429605 | 4.6170379 |
| C | 0.9380543 | 10.9951005 | 6.8980277 |
| H | 2.6134673 | 12.2540597 | 6.3852161 |
| C | 0.3679167 | 9.7460855 | 6.6978013 |
| H | 0.6078375 | 7.8549740 | 5.6873915 |
| H | 0.4642265 | 11.7301730 | 7.5320740 |
| N | 2.7677302 | 10.4144675 | 5.4762165 |
| C | -0.9540453 | 9.3706500 | 7.3312820 |
| F | -0.9036853 | 8.1284587 | 7.8428706 |
| F | -1.9379003 | 9.3927440 | 6.4141324 |
| F | -1.2898980 | 10.2144789 | 8.3176606 |

**Triethylamine**

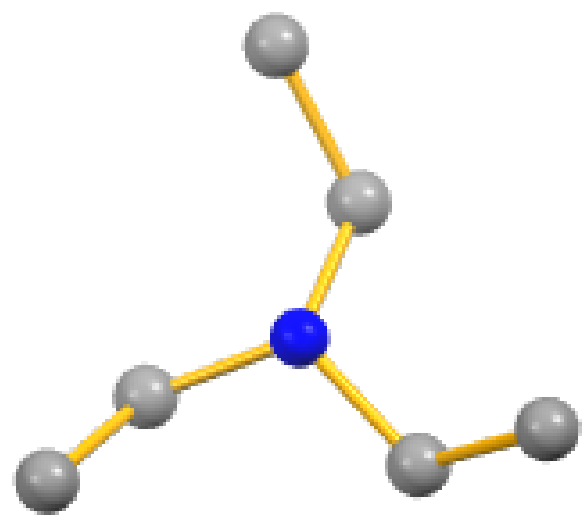

Energy: -292.3336613868 H
xyz, charge: 0, multiplicity: 1

Cartesian coordinates (in Å)

| | | | |
|---|---|---|---|
| N | 2.4438897 | -0.5362720 | -1.2478406 |
| C | 2.7400575 | -1.9432705 | -0.9727876 |
| H | 2.6873095 | -2.4918134 | -1.9142328 |
| H | 3.7758585 | -2.0636338 | -0.6068271 |
| C | 1.7599970 | -2.5798208 | 0.0075770 |
| H | 0.7427835 | -2.5151731 | -0.3822421 |
| H | 2.0084774 | -3.6321188 | 0.1651265 |

| | | | |
|---|---|---|---|
| H | 1.7772537 | -2.0887289 | 0.9821782 |
| C | 2.8903768 | 0.3444376 | -0.1669926 |
| H | 2.7638482 | -0.1861274 | 0.7776947 |
| H | 3.9709831 | 0.5586894 | -0.2545158 |
| C | 2.1011567 | 1.6477299 | -0.0934932 |
| H | 1.0406682 | 1.4372762 | 0.0544811 |
| H | 2.4559714 | 2.2613730 | 0.7380148 |
| H | 2.2011979 | 2.2400023 | -1.0048793 |
| C | 2.9499226 | -0.1094591 | -2.5535497 |
| H | 2.9896073 | 0.9806376 | -2.5624157 |
| H | 3.9888902 | -0.4541774 | -2.7053579 |
| C | 2.0710552 | -0.5659771 | -3.7137121 |
| H | 2.4765719 | -0.2067919 | -4.6626229 |
| H | 2.0051471 | -1.6536917 | -3.7753127 |
| H | 1.0590751 | -0.1751145 | -3.5954981 |

**Triethylamine-BH$_3$**

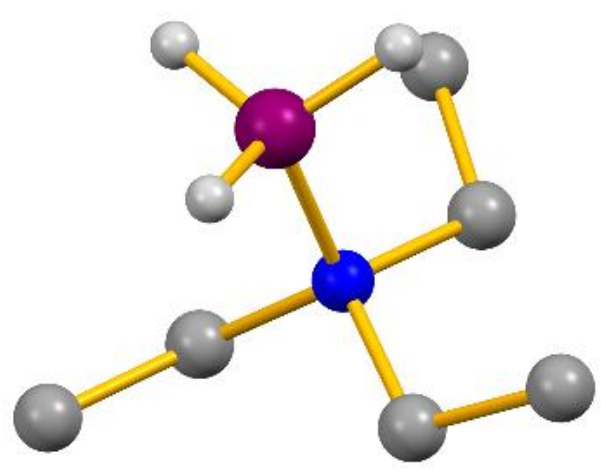

Energy: -318.9835576410 H
xyz, charge: 0, multiplicity: 1

Cartesian coordinates (in Å)

| | | | |
|---|---|---|---|
| C | 2.5092436 | -1.9636557 | -1.0705752 |
| H | 2.1314572 | -2.4997342 | -1.9360438 |
| H | 3.6044920 | -1.9865540 | -1.1074695 |
| C | 2.0096893 | -2.6622147 | 0.1854582 |
| H | 0.9254418 | -2.6085392 | 0.2567611 |
| H | 2.3006723 | -3.7129678 | 0.1266683 |
| H | 2.4429639 | -2.2558825 | 1.0990719 |
| C | 2.6295310 | 0.2724564 | -0.0945405 |
| H | 2.2110743 | -0.1627574 | 0.8079666 |
| H | 3.7147803 | 0.1197939 | -0.0745572 |
| C | 2.3071630 | 1.7594393 | -0.1075680 |
| H | 1.2339297 | 1.9256394 | -0.1712761 |
| H | 2.6690776 | 2.1920802 | 0.8273716 |
| H | 2.7949476 | 2.2933992 | -0.9227174 |
| C | 2.6759677 | -0.0051771 | -2.5211812 |
| H | 2.3801516 | 1.0375292 | -2.5873215 |
| H | 3.7664369 | -0.0436683 | -2.4172115 |
| C | 2.2434215 | -0.7113506 | -3.7976690 |
| H | 2.6604912 | -0.1635502 | -4.6451593 |
| H | 2.6089263 | -1.7359685 | -3.8617376 |
| H | 1.1596387 | -0.7183917 | -3.8918697 |
| H | 0.0248492 | -1.3868595 | -1.9023596 |
| H | 0.0408768 | -0.4284551 | -0.1372521 |

| | | | |
|---|---|---|---|
| H | 0.1548609 | 0.6167538 | -1.8485317 |
| B | 0.4154104 | -0.4220384 | -1.2869839 |
| N | 2.0769540 | -0.5309545 | -1.2428447 |

**[(NEt$_3$)(H){B($\mu$-hpp)}$_2$]$^+$**

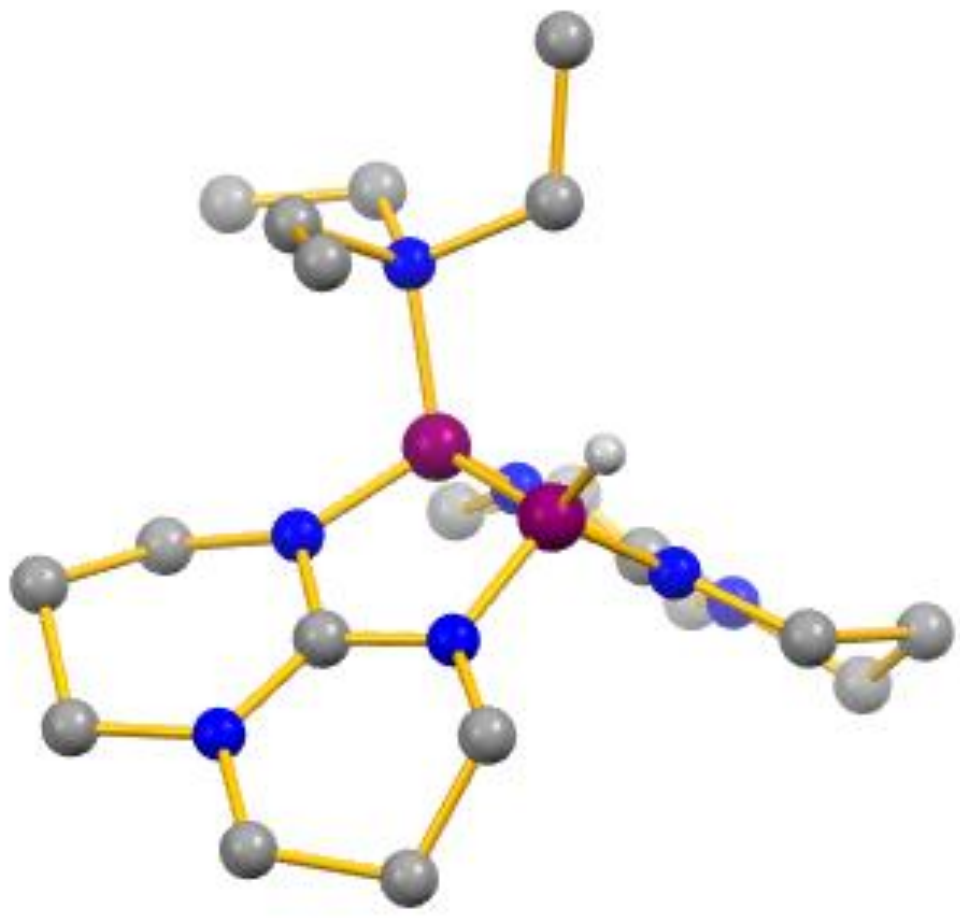

Energy: -1218.876287313 H
xyz, charge: +1, multiplicity: 1

Cartesian coordinates (in Å)

| | | | |
|---|---|---|---|
| N | 4.7980911 | 12.1356242 | 5.2305381 |
| N | 4.7193809 | 12.8577379 | 3.0714761 |
| N | 5.7668976 | 14.2274222 | 4.6641866 |
| N | 5.0311515 | 9.7076279 | 4.3111394 |
| N | 4.7443786 | 10.4149487 | 2.1667070 |
| N | 6.0973202 | 8.5286773 | 2.5491033 |
| B | 4.1076276 | 10.9279750 | 4.5451559 |
| B | 3.9369562 | 11.5188383 | 2.9223507 |
| C | 5.1062361 | 13.0987526 | 4.3203273 |
| C | 5.4047532 | 12.2498935 | 6.5521785 |
| H | 6.4437965 | 11.8942685 | 6.5435752 |
| H | 4.8586180 | 11.6176515 | 7.2407830 |
| C | 5.3540757 | 13.6934759 | 7.0302826 |
| H | 4.3105133 | 14.0121216 | 7.0961881 |
| H | 5.7966741 | 13.7922596 | 8.0223046 |
| C | 6.1053440 | 14.5684990 | 6.0457259 |
| H | 5.8659905 | 15.6235730 | 6.2029136 |
| H | 7.1872804 | 14.4591241 | 6.1834831 |
| C | 6.1579135 | 15.2265740 | 3.6642819 |
| H | 7.1530974 | 15.5911057 | 3.9308835 |
| H | 5.4725689 | 16.0794261 | 3.7286773 |
| C | 6.1564878 | 14.6483273 | 2.2592368 |
| H | 7.0072753 | 13.9738028 | 2.1333257 |
| H | 6.2601250 | 15.4553798 | 1.5330294 |
| C | 4.8652903 | 13.8744111 | 2.0409141 |
| H | 4.0041698 | 14.5543905 | 2.0478148 |
| H | 4.8662977 | 13.3751522 | 1.0730131 |
| C | 5.3085884 | 9.5381697 | 2.9879189 |
| C | 5.9558016 | 9.0950448 | 5.2629453 |

| | | | |
|---|---|---|---|
| H | 5.5164206 | 9.1225391 | 6.2542467 |
| H | 6.8891419 | 9.6716248 | 5.3179083 |
| C | 6.2564777 | 7.6625801 | 4.8522365 |
| H | 5.3309633 | 7.0813020 | 4.8654974 |
| H | 6.9559047 | 7.1936995 | 5.5460082 |
| C | 6.8517829 | 7.6622772 | 3.4549292 |
| H | 7.8955724 | 7.9987827 | 3.4891374 |
| H | 6.8529083 | 6.6540371 | 3.0355067 |
| C | 6.4473854 | 8.4480609 | 1.1294233 |
| H | 6.6768584 | 7.4054140 | 0.9058696 |
| H | 7.3546486 | 9.0321767 | 0.9312931 |
| C | 5.2908491 | 8.9404267 | 0.2750845 |
| H | 4.4423606 | 8.2615715 | 0.3917175 |
| H | 5.5754343 | 8.9391436 | -0.7776943 |
| C | 4.8998707 | 10.3409779 | 0.7207389 |
| H | 5.6618162 | 11.0607999 | 0.3975495 |
| H | 3.9563588 | 10.6490270 | 0.2672353 |
| H | 2.9043308 | 11.7341001 | 2.3236290 |
| N | 2.6768027 | 10.4122472 | 5.3417433 |
| C | 1.9689986 | 11.5831076 | 5.9746788 |
| H | 1.1256604 | 11.1900884 | 6.5462278 |
| H | 2.6634097 | 12.0176192 | 6.6877565 |
| C | 2.8146499 | 9.3051042 | 6.3722583 |
| H | 1.8152785 | 8.9069627 | 6.5305323 |
| H | 3.3954758 | 8.5216617 | 5.8896606 |
| C | 1.8278150 | 9.8051556 | 4.2278425 |
| H | 1.7919980 | 10.5452691 | 3.4405069 |
| H | 2.4129568 | 8.9691329 | 3.8446258 |
| C | 1.4897420 | 12.6668822 | 5.0217937 |
| H | 2.3030618 | 13.0799039 | 4.4317485 |
| H | 0.7194829 | 12.3135614 | 4.3381382 |
| H | 1.0541067 | 13.4700808 | 5.6187827 |
| C | 0.4127783 | 9.3554713 | 4.5744121 |
| H | -0.1892550 | 10.1560691 | 5.0043274 |
| H | -0.0685694 | 9.0622494 | 3.6396673 |
| H | 0.3651170 | 8.4933481 | 5.2383537 |
| C | 3.3806163 | 9.6221430 | 7.7478671 |
| H | 3.2873229 | 8.7163551 | 8.3500265 |
| H | 4.4335560 | 9.8883637 | 7.7399810 |
| H | 2.8263849 | 10.4054756 | 8.2631770 |

**4-Nitropyridine**

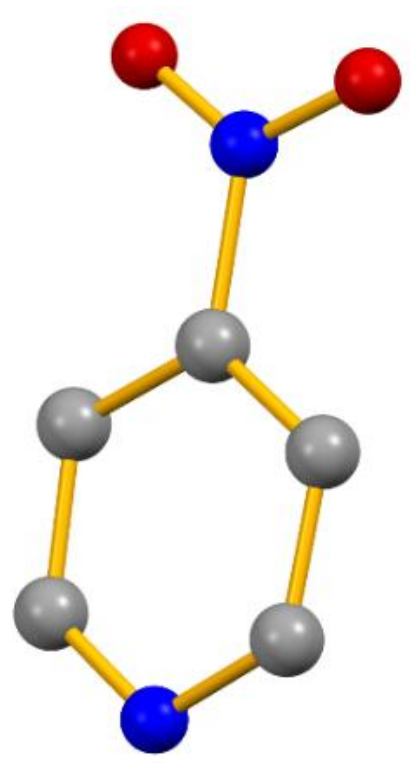

Energy: -452.7342095920
xyz, charge: 0, multiplicity: 1

<u>Cartesian coordinates (in Å)</u>

| | | | |
|---|---|---|---|
| C | -1.5134444 | -2.4412190 | 0.0010957 |
| C | -0.1406820 | -2.4516019 | -0.1728592 |
| C | 0.5055956 | -1.2194316 | -0.1640925 |
| C | -2.2000733 | -1.2524666 | 0.1748239 |
| H | 0.3951930 | -3.3789095 | -0.3083560 |
| H | 1.5807639 | -1.1710308 | -0.2960193 |
| H | -3.2710177 | -1.2444151 | 0.3105577 |
| N | -0.1249058 | -0.0564455 | 0.0006121 |
| C | -1.4475125 | -0.0821700 | 0.1655459 |
| H | -1.9360659 | 0.8768388 | 0.2972741 |
| N | -2.2606236 | -3.7246620 | 0.0013589 |
| O | -3.4683990 | -3.6701138 | 0.1583191 |
| O | -1.6169772 | -4.7480695 | -0.1553884 |

**4-Nitropyridine-BH$_3$**

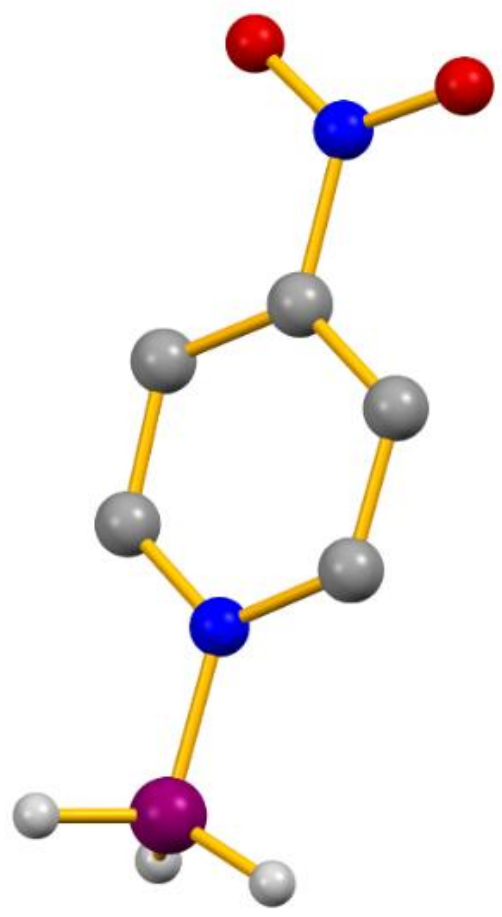

Energy: -479.3794435236 H
xyz, charge: 0, multiplicity: 1

<u>Cartesian coordinates (in Å)</u>

| | | | |
|---|---|---|---|
| C | -1.5071992 | -2.4346880 | -0.0009471 |
| C | -0.1332121 | -2.4515367 | -0.1459962 |
| C | 0.5301505 | -1.2350921 | -0.1368872 |
| C | -2.1925138 | -1.2389006 | 0.1481316 |
| H | 0.4032911 | -3.3807269 | -0.2615074 |
| H | 1.6021354 | -1.1640808 | -0.2438180 |
| H | -3.2654412 | -1.2233321 | 0.2622596 |
| C | -1.4536713 | -0.0730662 | 0.1463316 |
| H | -1.9114905 | 0.8990378 | 0.2582645 |
| N | -2.2596239 | -3.7134319 | -0.0037355 |
| H | 0.2480630 | 1.9323702 | -0.9672684 |
| H | 0.3490578 | 1.8894175 | 1.0303733 |
| H | 1.8469020 | 1.0802084 | -0.0680131 |
| B | 0.6710625 | 1.3388824 | 0.0010859 |
| N | -0.1166350 | -0.0698172 | 0.0055902 |

| | | | |
|---|---|---|---|
| O | -1.6146004 | -4.7385461 | -0.1350341 |
| O | -3.4692931 | -3.6466519 | 0.1253809 |

**[(NO$_2$-Py)(H){B($\mu$-hpp)}$_2$]$^+$**

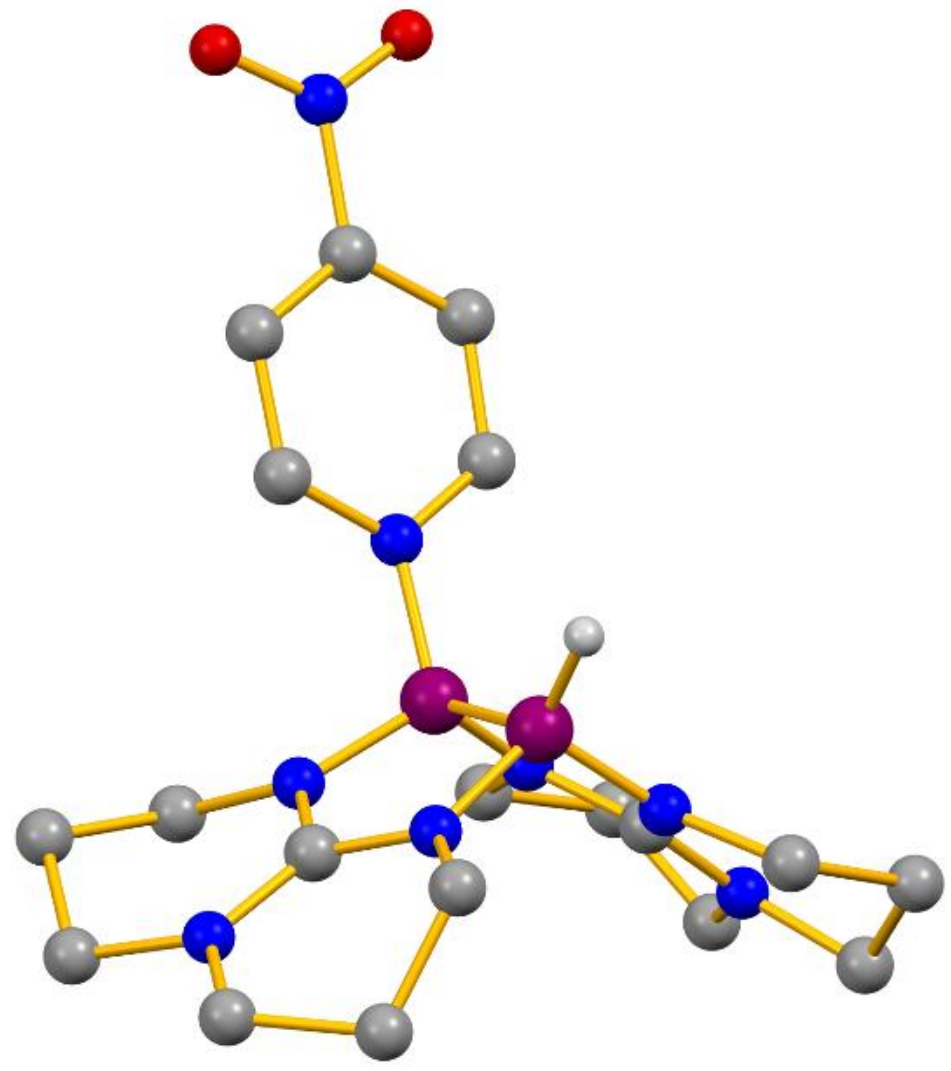

Energy: -1379.284310422 H
xyz, charge: +1, multiplicity: 1

Cartesian coordinates (in Å)

| | | | |
|---|---|---|---|
| N | 4.8036733 | 11.9486920 | 5.4143854 |
| N | 4.5913425 | 12.9137231 | 3.3720460 |
| N | 5.7736648 | 14.0717159 | 5.0461548 |
| N | 4.8739845 | 9.6040678 | 4.2866478 |
| N | 4.6310562 | 10.5620375 | 2.2455458 |
| N | 5.9175433 | 8.5944595 | 2.4176851 |
| B | 4.0551959 | 10.8427384 | 4.6636092 |
| B | 3.8149026 | 11.5858480 | 3.0996459 |
| C | 5.0739483 | 13.0066594 | 4.6083576 |
| C | 5.4297522 | 11.8587998 | 6.7270992 |
| H | 6.4341166 | 11.4228266 | 6.6555272 |
| H | 4.8344395 | 11.1847434 | 7.3460526 |
| C | 5.5074345 | 13.2421863 | 7.3580097 |
| H | 4.4963707 | 13.6198655 | 7.5323539 |
| H | 6.0163813 | 13.2036199 | 8.3217476 |
| C | 6.2546519 | 14.1819271 | 6.4251307 |
| H | 6.1248399 | 15.2188468 | 6.7434482 |
| H | 7.3296013 | 13.9689028 | 6.4471914 |
| C | 6.1099638 | 15.1864629 | 4.1533780 |
| H | 7.1270130 | 15.5074474 | 4.3900616 |
| H | 5.4436838 | 16.0285032 | 4.3702713 |
| C | 6.0035880 | 14.7813623 | 2.6912789 |
| H | 6.8364286 | 14.1242755 | 2.4281558 |
| H | 6.0690951 | 15.6699107 | 2.0624187 |
| C | 4.6909262 | 14.0451283 | 2.4595484 |
| H | 3.8398731 | 14.7229843 | 2.5970987 |
| H | 4.6324311 | 13.6646380 | 1.4407141 |

| | | | |
|---|---|---|---|
| C | 5.1638047 | 9.5759337 | 2.9605709 |
| C | 5.5537649 | 8.7083392 | 5.2155931 |
| H | 4.9119905 | 8.5670098 | 6.0881675 |
| H | 6.4850416 | 9.1617194 | 5.5771729 |
| C | 5.8519807 | 7.3738938 | 4.5490945 |
| H | 4.9164438 | 6.8517988 | 4.3318561 |
| H | 6.4421112 | 6.7374099 | 5.2094979 |
| C | 6.6114788 | 7.6155721 | 3.2544758 |
| H | 7.6303558 | 7.9621366 | 3.4679622 |
| H | 6.6980965 | 6.6902534 | 2.6830119 |
| C | 6.2855808 | 8.6638652 | 1.0019276 |
| H | 6.4794425 | 7.6463012 | 0.6608405 |
| H | 7.2152390 | 9.2332139 | 0.8812264 |
| C | 5.1574492 | 9.2950719 | 0.2008624 |
| H | 4.2854065 | 8.6372159 | 0.2272830 |
| H | 5.4574044 | 9.4074028 | -0.8415830 |
| C | 4.8028730 | 10.6481860 | 0.8003076 |
| H | 5.5876450 | 11.3783167 | 0.5684134 |
| H | 3.8726278 | 11.0307918 | 0.3772604 |
| H | 2.7360202 | 11.8030658 | 2.5994553 |
| C | 2.1830050 | 9.2123233 | 5.2425686 |
| C | 2.1118182 | 11.3044476 | 6.2495406 |
| C | 0.9771237 | 8.8537764 | 5.8081756 |
| H | 2.7275202 | 8.5503400 | 4.5873100 |
| C | 0.9039372 | 11.0193763 | 6.8513166 |
| H | 2.6002987 | 12.2585622 | 6.3709742 |
| C | 0.3406921 | 9.7754497 | 6.6221262 |
| H | 0.5402694 | 7.8841052 | 5.6225231 |
| H | 0.4098960 | 11.7434094 | 7.4816681 |
| N | 2.7501295 | 10.4184462 | 5.4562681 |
| N | -0.9552420 | 9.4274114 | 7.2539683 |
| O | -1.4644780 | 10.2728252 | 7.9664841 |
| O | -1.4063953 | 8.3232049 | 7.0118448 |

**4,4-Bipyridine**

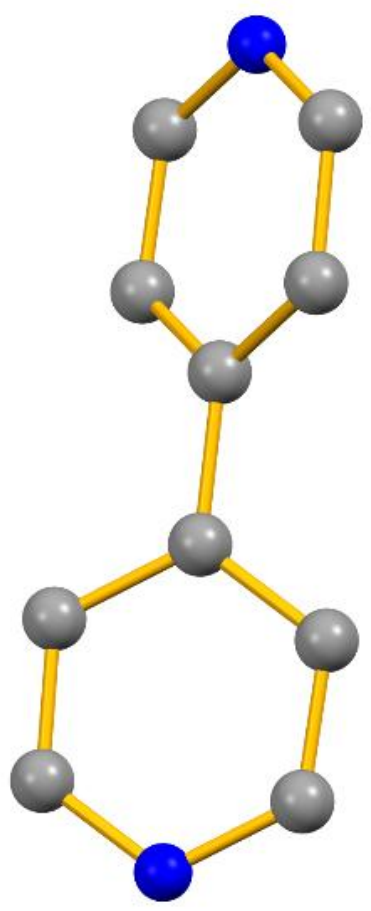

Energy: -495.2707055053 H
xyz, charge: 0, multiplicity: 1

Cartesian coordinates (in Å)

| | | | |
|---|---|---|---|
| C | -1.3490214 | -1.4777739 | 0.0005281 |
| C | -0.0093295 | -1.4078216 | 0.3887604 |
| C | 0.6403052 | -0.1800680 | 0.3729740 |
| C | -1.2191893 | 0.8936062 | -0.3733364 |
| C | -1.9580268 | -0.2826432 | -0.3882238 |
| H | 0.5174092 | -2.2924103 | 0.7225928 |
| H | 1.6788732 | -0.1115182 | 0.6804532 |
| H | -1.6788949 | 1.8273220 | -0.6810998 |
| H | -2.9875836 | -0.2686441 | -0.7217166 |
| N | 0.0593649 | 0.9607115 | -0.0004001 |
| C | -2.0887155 | -2.7584976 | 0.0013237 |
| C | -3.4283481 | -2.8280626 | 0.3898404 |
| C | -1.4797129 | -3.9540483 | -0.3861291 |
| C | -4.0779176 | -4.0558580 | 0.3756055 |
| H | -3.9550567 | -1.9430952 | 0.7227189 |
| C | -2.2184893 | -5.1303281 | -0.3697226 |
| H | -0.4502022 | -3.9684175 | -0.7197459 |
| H | -5.1164420 | -4.1241054 | 0.6832951 |
| H | -1.7587703 | -6.0643807 | -0.6764447 |
| N | -3.4969781 | -5.1970526 | 0.0034816 |

**4,4-Bipyridine-$BH_3$**

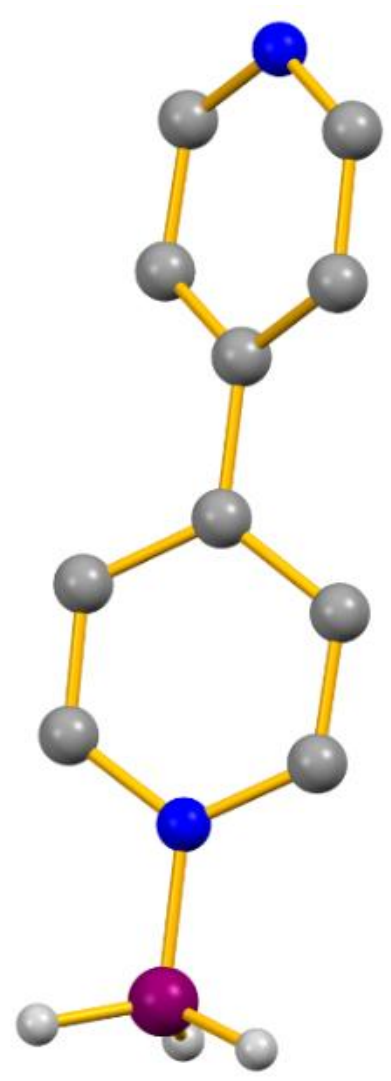

Energy: -521.9200482754 H
xyz, charge: 0, multiplicity: 1

Cartesian coordinates (in Å)

| | | | |
|---|---|---|---|
| C | -1.3502993 | -1.4861937 | -0.0085822 |
| C | 0.0017110 | -1.4210346 | 0.3378714 |
| C | 0.6628129 | -0.2086780 | 0.3164818 |
| C | -1.2432287 | 0.8902267 | -0.3708390 |
| C | -1.9678970 | -0.2852359 | -0.3676568 |
| H | 0.5397307 | -2.3071646 | 0.6445294 |
| H | 1.7061047 | -0.1169458 | 0.5818372 |

| | | | |
|---|---|---|---|
| H | -1.6814539 | 1.8360810 | -0.6548305 |
| H | -3.0049222 | -0.2636409 | -0.6723898 |
| C | -2.0907579 | -2.7644516 | 0.0006502 |
| C | -3.4225175 | -2.8309303 | 0.4156014 |
| C | -1.4890637 | -3.9578379 | -0.4041565 |
| C | -4.0734210 | -4.0582704 | 0.4099448 |
| H | -3.9416154 | -1.9476371 | 0.7644101 |
| C | -2.2300203 | -5.1327979 | -0.3787351 |
| H | -0.4671645 | -3.9732813 | -0.7602379 |
| H | -5.1051332 | -4.1272216 | 0.7387457 |
| H | -1.7790528 | -6.0661505 | -0.6993464 |
| N | -3.5003079 | -5.1972555 | 0.0205857 |
| H | 0.3402335 | 3.0472564 | -0.8242315 |
| H | 0.6685391 | 2.7675858 | 1.1400019 |
| H | 2.0090885 | 2.0860132 | -0.1959660 |
| B | 0.8465454 | 2.3417620 | 0.0173954 |
| N | 0.0536177 | 0.9350899 | -0.0300596 |

**[(Bipy)(H){B(*μ*-hpp)}$_2$]$^+$**

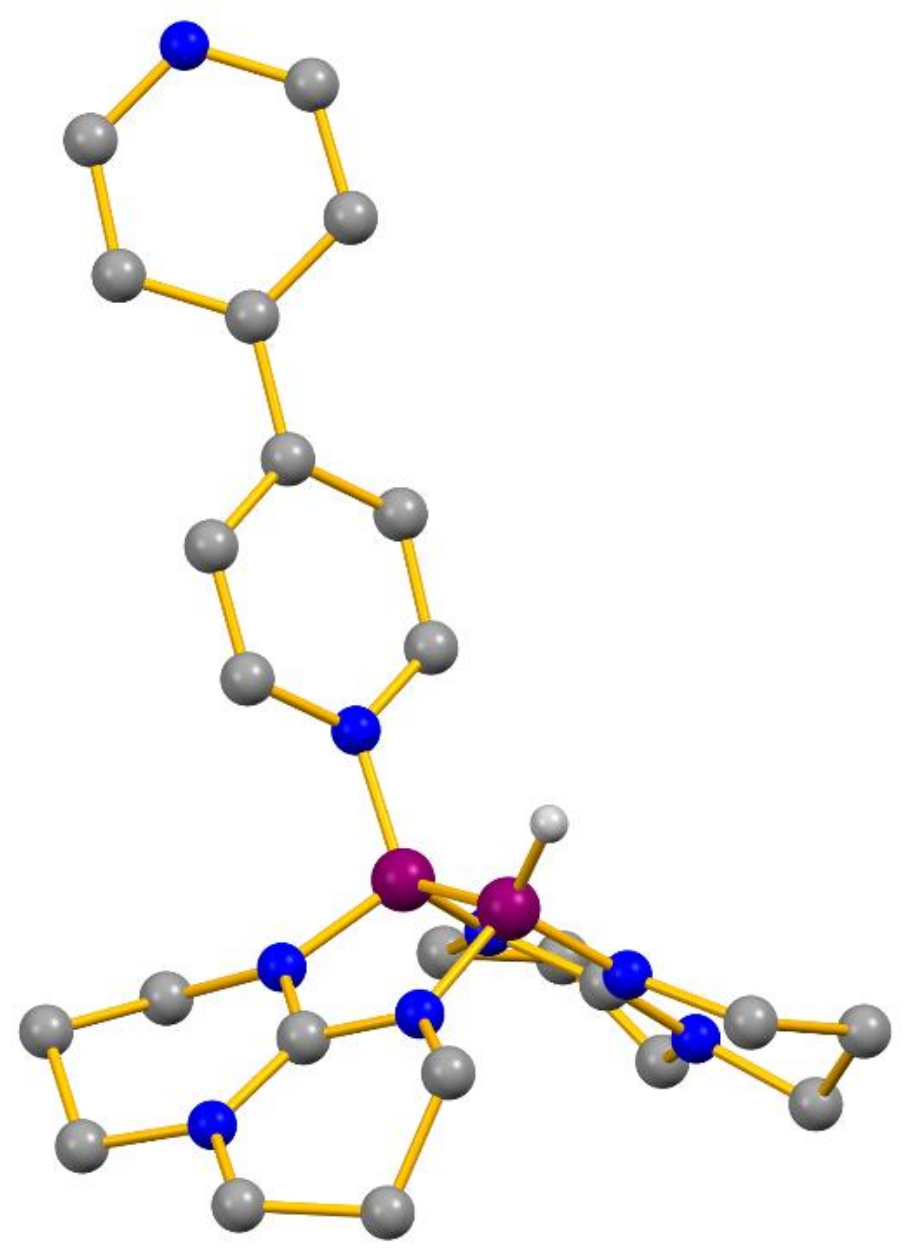

Energy: -1421.830539614 H
xyz, charge: +1, multiplicity: 1

Cartesian coordinates (in Å)

| | | | |
|---|---|---|---|
| N | 4.8672682 | 11.9169516 | 5.4322865 |
| N | 4.5580254 | 12.9102683 | 3.4160165 |
| N | 5.8049913 | 14.0530061 | 5.0544678 |
| N | 4.9108764 | 9.5924440 | 4.2691473 |
| N | 4.5722560 | 10.5773149 | 2.2546261 |
| N | 5.8881130 | 8.6225424 | 2.3434895 |
| B | 4.0859073 | 10.8169185 | 4.6959955 |
| B | 3.7779549 | 11.5760212 | 3.1641580 |
| C | 5.0927988 | 12.9874531 | 4.6315547 |
| C | 5.5513720 | 11.8143043 | 6.7132254 |
| H | 6.5568587 | 11.3912229 | 6.5913775 |

| | | | |
|---|---|---|---|
| H | 4.9913582 | 11.1237455 | 7.3466084 |
| C | 5.6430983 | 13.1883737 | 7.3625740 |
| H | 4.6364502 | 13.5527691 | 7.5843985 |
| H | 6.1930505 | 13.1409003 | 8.3032895 |
| C | 6.3411704 | 14.1492278 | 6.4131058 |
| H | 6.2145949 | 15.1803152 | 6.7519053 |
| H | 7.4184671 | 13.9473164 | 6.3887956 |
| C | 6.0969542 | 15.1811067 | 4.1644218 |
| H | 7.1217027 | 15.5052065 | 4.3614327 |
| H | 5.4365244 | 16.0171504 | 4.4203981 |
| C | 5.9298574 | 14.7958915 | 2.7025648 |
| H | 6.7537553 | 14.1467348 | 2.3950086 |
| H | 5.9641859 | 15.6934963 | 2.0839553 |
| C | 4.6122901 | 14.0548840 | 2.5183191 |
| H | 3.7650366 | 14.7270131 | 2.7024979 |
| H | 4.5104774 | 13.6893614 | 1.4973557 |
| C | 5.1448453 | 9.5881675 | 2.9332294 |
| C | 5.6414119 | 8.6943787 | 5.1543379 |
| H | 5.0395277 | 8.5320655 | 6.0512230 |
| H | 6.5813046 | 9.1557518 | 5.4831174 |
| C | 5.9311543 | 7.3735219 | 4.4576351 |
| H | 4.9947791 | 6.8410630 | 4.2713085 |
| H | 6.5576563 | 6.7367095 | 5.0835612 |
| C | 6.6323349 | 7.6453694 | 3.1363559 |
| H | 7.6536766 | 8.0059056 | 3.3137575 |
| H | 6.7112582 | 6.7292914 | 2.5487074 |
| C | 6.2045583 | 8.7236387 | 0.9182230 |
| H | 6.4010952 | 7.7155984 | 0.5507471 |
| H | 7.1210355 | 9.3093869 | 0.7742783 |
| C | 5.0397998 | 9.3528005 | 0.1696880 |
| H | 4.1783237 | 8.6821752 | 0.2164845 |
| H | 5.2998835 | 9.4875021 | -0.8809162 |
| C | 4.6893376 | 10.6897197 | 0.8070116 |
| H | 5.4550399 | 11.4347885 | 0.5579370 |
| H | 3.7390981 | 11.0668040 | 0.4251499 |
| H | 2.6825490 | 11.7964417 | 2.6993773 |
| C | 2.2843353 | 9.1376438 | 5.3908865 |
| C | 2.2341755 | 11.2051334 | 6.4202171 |
| C | 1.1307152 | 8.7504949 | 6.0362280 |
| H | 2.7965903 | 8.4897524 | 4.6957571 |
| C | 1.0812295 | 10.8777384 | 7.0989193 |
| H | 2.6981154 | 12.1750119 | 6.5154732 |
| C | 0.4892999 | 9.6221779 | 6.9229669 |
| H | 0.7435742 | 7.7568782 | 5.8597360 |
| H | 0.6292183 | 11.6170062 | 7.7450490 |
| N | 2.8429077 | 10.3501078 | 5.5750761 |
| C | -0.7472686 | 9.2402892 | 7.6299429 |
| C | -0.9941647 | 9.6606243 | 8.9388734 |
| C | -1.7175683 | 8.4445621 | 7.0165668 |
| C | -2.1726401 | 9.2689273 | 9.5624295 |
| H | -0.2730544 | 10.2583376 | 9.4812353 |
| C | -2.8673257 | 8.1165588 | 7.7249163 |
| H | -1.6025103 | 8.1079361 | 5.9944351 |
| H | -2.3768635 | 9.5760081 | 10.5825302 |
| H | -3.6373671 | 7.5093103 | 7.2614285 |
| N | -3.0998807 | 8.5131070 | 8.9755841 |

# 5 References


[1] A. Wagner, S. Litters, J. Elias, E. Kaifer, H.-J. Himmel, *Chem. Eur. J.* **2014**, *20*, 12514–12527.

[2] O. Ciobanu, P. Roquette, S. Leingang, H. Wadepohl, J. Mautz, H.-J. Himmel, *Eur. J. Inorg. Chem.* **2007**, 4530–4534.

[3] J. Horn, A. Widera, S. Litters, E. Kaifer, H.-J. Himmel, *Dalton Trans.* **2018**, *47*, 2009–2017.

[4] D. Vogler, L. Haas, N. Wolf, E. Kaifer, H.-J. Himmel, *Chem. Commun.* **2022**, *58*, 693–696.

[5] A. Widera, D. Vogler, H. Wadepohl, E. Kaifer, H.-J. Himmel, *Angew. Chem. Int. Ed.* **2018***,* *57*, 11456–11459.

[6] J. Hu, D. Zhang, F. W. Harris, *J. Org. Chem*. **2005**, *70*, 707–708.

[7] SAINT (APEX 3/4/6) Bruker AXS GmbH, Karlsruhe, Germany **2016/2021/2025**.

[8] G. M. Sheldrick, SADABS, Bruker AXS GmbH, Karlsruhe, Germany **2004-2014**.

[9] L. Krause, R. Herbst-Irmer, G. M. Sheldrick, D. Stalke, *J. Appl. Cryst*. **2015**, *48*, 3–10.

[10] G. M. Sheldrick, SHELXT, Program for Crystal Structure Solution, University of Göttingen, Germany **2014-2018**.

[11] G. M. Sheldrick, *Acta Cryst.* **2015**, *C71*, 3–8.

[12] G. M. Sheldrick, SHELXL-20xx, University of Göttingen and Bruker AXS GmbH, Karlsruhe, Germany **2012-2018**.

[13] W. Robinson, G. M. Sheldrick in: N. W. Isaaks, M. R. Taylor (eds.) „Crystallographic Computing 4“, Ch. 22, IUCr and Oxford University Press, Oxford, UK, **1988.**

[14] G. M. Sheldrick, *Acta Cryst*. **2008**, *A64*, 112–122.

[15] O. V. Dolomanov, L. J. Bourhis, R. J. Gildea, J. A. K. Howard, H. Puschmann, *J. Appl. Cryst.* **2009**, *42*, 339–341.

[16] A. Thorn, B. Dittrich, G. M. Sheldrick, *Acta Cryst.* **2012**, *A68*, 448–451.

[17] P. v. d. Sluis, A. L. Spek, *Acta Cryst.* **1990**, *A46*, 194–201.

[18] A. L. Spek*, Acta Cryst.* **2015**, *C71*, 9–18.

[19] A. L. Spek, PLATON, Utrecht University, The Netherlands; b) A. L. Spek, *J. Appl. Cryst.* **2003**, *36*, 7–13.

[20] C. F. Macrae, I. Sovago, S. J. Cottrell, P. T. A. Galek, P. McCabe, E. Pidcock, M. Platings, G. P. Shields, J. S. Stevens, M. Towler and P. A. Wood, *J. Appl. Cryst.* **2020***,* *53*, 226–235.

[21] TURBOMOLE V7.7 **2023**, a development of University of Karlsruhe and Forschungszentrum Karlsruhe GmbH, **1989-2007**, TURBOMOLE GmbH, since **2007**; available from http://www.turbomole.org.
[22] F. Neese, *WIREs Comput. Mol. Sci.* **2012***, 2*, 73–78.
[23] F. Neese, *WIREs Comput. Mol. Sci.* **2025***, 15*, 2, e70019.
[24] A. D. Becke, *J. Chem. Phys.* **1993***, 98*, 5648.
[25] S. Grimme, J. Antony, S. Ehrlich, H. Krieg, *J. Chem. Phys.* **2010***, 132*, 154104.
[26] A. Schäfer, H. Horn, R. Ahlrichs, *J. Chem. Phys.* **1992***, 97*, 2571–2577.
[27] T. Yanai, D. P. Tew, N. C. Handy, *Chem. Phys. Lett.* **2004**, *393*, 51–57.
